# Genome-wide identification of whole ATP-binding cassette (ABC) transporters in the intertidal copepod *Tigriopus japonicus*

**DOI:** 10.1186/1471-2164-15-651

**Published:** 2014-08-05

**Authors:** Chang-Bum Jeong, Bo-Mi Kim, Jae-Seong Lee, Jae-Sung Rhee

**Affiliations:** Department of Chemistry, College of Natural Sciences, Hanyang University, Seoul, 133-791 South Korea; Department of Biological Sciences, College of Science, Sungkyunkwan University, Suwon, 440-746 South Korea; Department of Marine Science, College of Natural Sciences, Incheon National University, Incheon, 406-772 South Korea

**Keywords:** ATP-binding cassette transporter, Copepod, *Tigriopus japonicus*, Genome, Development

## Abstract

**Backgrounds:**

The ATP-binding cassette (ABC) transporter superfamily is one of the largest transporter gene families and is observed in all animal taxa. Although a large set of transcriptomic data was recently assembled for several species of crustaceans, identification and annotation of the large ABC transporter gene family have been very challenging.

**Results:**

In the intertidal copepod *Tigriopus japonicus*, 46 putative ABC transporters were identified using *in silico* analysis, and their full-length cDNA sequences were characterized. Phylogenetic analysis revealed that the 46 *T. japonicus* ABC transporters are classified into eight subfamilies (A-H) that include all the members of all ABC subfamilies, consisting of five ABCA, five ABCB, 17 ABCC, three ABCD, one ABCE, three ABCF, seven ABCG, and five ABCH subfamilies. Of them, unique isotypic expansion of two clades of ABCC1 proteins was observed. Real-time RT-PCR-based heatmap analysis revealed that most *T. japonicus ABC* genes showed temporal transcriptional expression during copepod development. The overall transcriptional profile demonstrated that half of all *T. japonicus ABC* genes were strongly associated with at least one developmental stage. Of them, transcripts *TJ-ABCH_88708* and *TJ-ABCE1* were highly expressed during all developmental stages.

**Conclusions:**

The whole set of *T. japonicus ABC* genes and their phylogenetic relationships will provide a better understanding of the comparative evolution of essential gene family resources in arthropods, including the crustacean copepods.

**Electronic supplementary material:**

The online version of this article (doi:10.1186/1471-2164-15-651) contains supplementary material, which is available to authorized users.

## Background

The ATP-binding cassette (ABC) transporters constitute one of the largest families of ubiquitous membrane proteins [1]. These proteins are present in all organisms from prokaryotes to eukaryotes (e.g., plants, fungi, yeast, and animals) and use ATP hydrolysis to transport diverse substrates (e.g., amino acids, peptides, vitamins, sugars, lipids, sterols, hormones, endogenous metabolites, inorganic anions, drugs, and metal ions) in and out of cells across biological membranes [2]. ABC transporters share highly conserved structural domains known as nucleotide binding domains (NBDs, also referred to as ATP-binding cassettes) and are classified according to the presence of three sequence motifs: Walker A, Walker B, and Walker C [3]. Substrate specificity depends on the transmembrane domains (TMDs), while the ability to bind and hydrolyze ATP is achieved by the NBDs, which are related to energy production for substrate translocation or non-transport processes in a cell. These domains form either homo- or heterodimers (half transporters: one TMD and one NBD, full transporter: two TMDs and two NBDs), and they are located on a single polypeptide chain. Within the NBDs, several architectural motifs, such as the Walker motifs and ABC signature, can be used as unique characteristics for investigating ABC transporters [4].

To date, several ABC transporter superfamilies have been characterized from diverse animal taxa, and there are ongoing investigations into the potential roles of each subfamily. Previously, 71 discrete ABC transporters were identified in the genome of the bacterium *Escherichia coli* strain K-12 [5], while the human genome contains only 48 ABC transporters in seven subfamilies (designated A-G) [1]. Interestingly, the eighth subfamily H was first identified in the *Drosophila melanogaster* genome [1] and has been subsequently identified in several lower taxa such as *Caenorhabditis elegans*[6], *Daphnia pulex*[7], *Anopheles gambiae*[8, 9], *Apis mellifera*[9, 10], *Bombyx mori*[9], *Tetranychus urticae*[11], and *Tribilium castaneum*[12]. However, while one member of the H subfamily has been found in the zebrafish *Danio rerio*[13], there is currently no evidence of the H subfamily in mammals. In fact, the role of ABC transporters is not restricted to ATP-dependent active transport as several subfamily members function as receptors or ion channels. For example, the members of the E and F subfamilies are not transporters, as they possess two NBDs, but lack TMDs. Thus, the suggested roles of both subfamilies include transcription, translation, or ribosome assembly [14, 15].

In invertebrates, the importance of ABC transporters has been emphasized due to their potential roles in a wide range of molecular and biochemical metabolisms. Regardless of genomic technology, the study of invertebrate ABC transporters has mainly focused on the identification and functional characterization of P-glycoprotein (P-gp) and other *ABC* genes that are potentially involved in multidrug resistance and/or xenobiotic elimination. During the last decade, genomic analysis has significantly aided our understanding of the diversity and functions of invertebrate ABC superfamilies. Moreover, recent state-of-the-art sequencing technologies have provided unique opportunities for comparative analysis of the entire ABC superfamily and allowed easy access to the differentially expressed profiles in a species. Previously, Dean *et al.*[1] identified 56 ABC transporters in the *D. melanogaster* genome and compared them to those of human ABC transporters. Sheps *et al.*[6] reported 60 *C. elegans* ABC transporters and suggested gene duplication and loss events in the *C. elegans* genome based on orthologous gene comparison from analogous genes. In the water flea *D. pulex*, Sturm *et al.*[7] suggested that 64 *Daphnia* ABC transporters most closely resembled those of *D. melanogaster* based on phylogenetic relationship. In insects, Liu *et al.*[9] identified 51 ABC transporters in the silkworm *B. mori* and showed their spatial transcriptional expressions in multiple larval tissues. Broehan *et al.*[13] annotated 73 ABC transporters in the genome of the flour beetle *T. castaneum* that have several physiological functions in developmental stages. Recently, Dermauw *et al.*[11] investigated 103 ABC transporters in the genome of the polyphagous spider mite *T. urticae,* which has the largest number of ABC subfamilies among metazoan species. However, only a few ABC proteins have been functionally characterized in other invertebrates, including copepods.

Copepods are widely distributed and ecologically important invertebrates in aquatic ecosystems. *T. japonicus* has been identified as a significant copepod species for assessing impacts of environmental changes [16]. In the whole genome and RNA-Seq databases of *T. japonicus*[17], we identified multiple ABC transporters exhibiting high similarities with those of other invertebrates. Thus, we were able to characterize all of the ABC transporters in *T. japonicus*. Although there is limited information on copepod *ABC* gene repositories, Tribble *et al.*[18] initially showed P-gp-mediated tolerance of parasitic sea lice (*Lepeophtheirus salmonis*) against antibiotic treatment from commercial sea cage salmon farms. However, extensive *ABC* gene mining and functional characterization have not yet been reported in copepods. In this study, we identified and characterized 46 putative *T. japonicus* ABC transporters at the genome- and transcriptome levels. In addition, we investigated their temporal transcriptional levels using real-time RT-PCR-based heatmap analysis for different developmental stages.

## Results and discussion

### Identification and annotation of the *T. japonicus*ABC transporters

**Figure 1:**
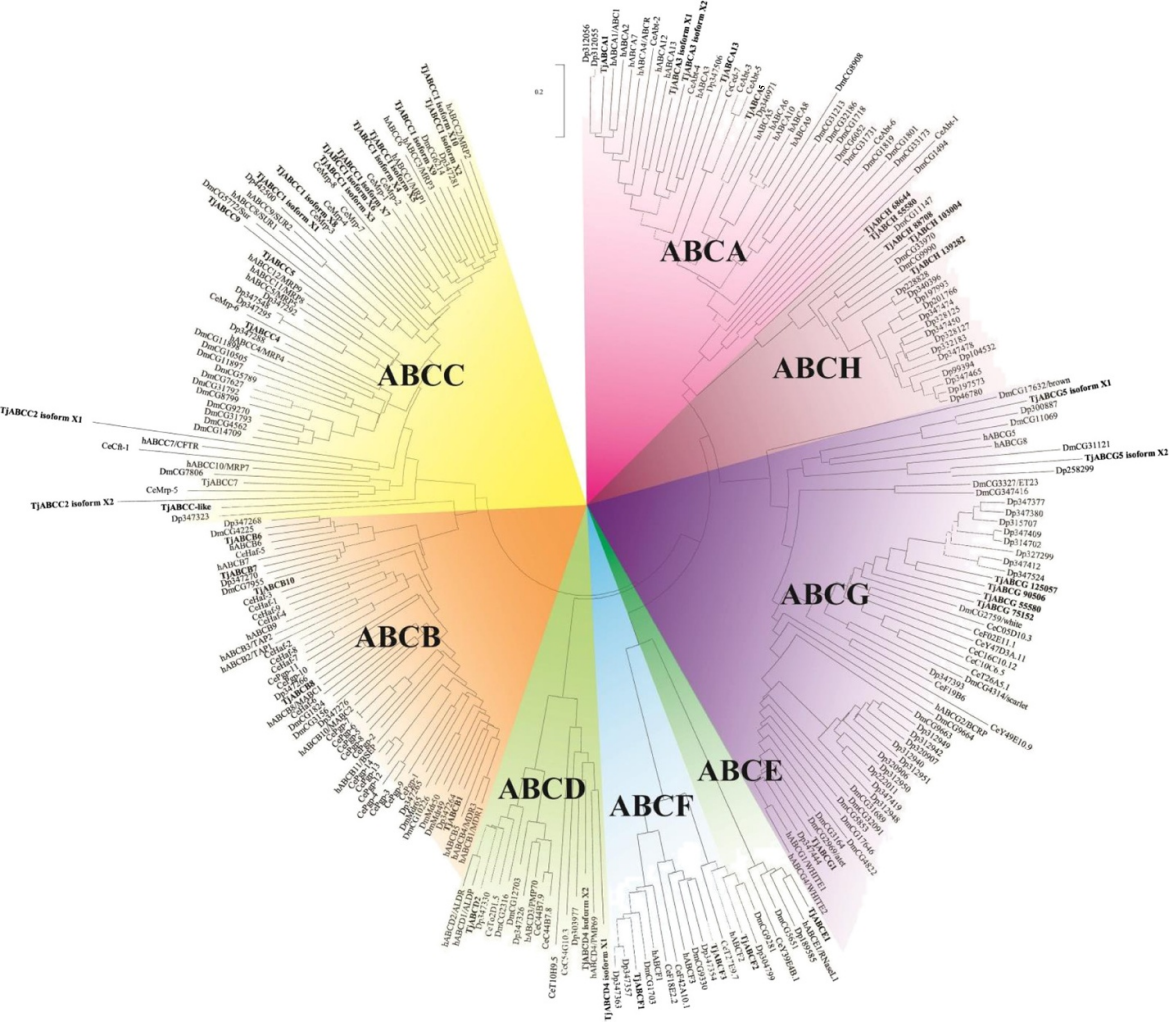
**Phylogenetic analysis of 46** ***T. japonicus*** **ABC transporters using the Bayesian method.** Numbers at branch nodes represent the confidence level of posterior probability.

In this study, we identified 46 ABC transporters in the copepod *T. japonicus* whole genome and RNA-Seq databases. All NBD-containing reads were examined by BLAST search to the non-redundant (NR) database of NCBI. To annotate each subfamily, full-length *ABC* sequences were obtained from *in silico* analysis and/or the RACE method, and entire *T. japonicus ABC* genes were subsequently registered in GenBank (Table 1, Additional file 1). Thus, this is the first report on the cloning and characterization of the entire ABC superfamily in copepods. Full-length ABC proteins were ranged in size from 3124 amino acids (ABCA13) to 603 amino acids (ABCD4 isoform X1). In our preliminary phylogenetic analysis (Figure 1), 46 *T. japonicus ABC* genes were separated into eight subfamilies named A to H, which were confirmed by Sturm *et al.*[7]. To analyze the evolutionary placement of the 46 *T. japonicus ABC* genes, in-depth phylogenetic analyses were conducted for each subfamily in comparison to those of yeast (*S. cerevisiae*), worm (*C. elegans*), fruit fly (*D. melanogaster*), and human (*H. sapiens*) based on the criteria of Sturm *et al.*[7] (Additional files 2, 3, 4, 5, 6, 7 and 8). As discussed below, this kind of in-depth phylogenetic analysis allows evaluation of the evolutionary distance of each *T. japonicus* ABC transporter across the different families.

**Table 1:** **Information on 46 ABC transporters identified in the** ***T. japonicus*** **genome**

Gene	Length (AA)	Accession no.	Matched gene	Matched species	E-value	Real-time RT-PCR primer (5′ → 3′)
ABCA1	2428	KF906264	ABCA1 (XP003747270)	*Metaseiulus occidentalis*	0	F: CGGCAGGTCTTATGAGTTTC R: CATTGTAAGTTTGGATTTGGG
ABCA3 isoform X1	1541	KF906265	ABCA3 (EKC30762)	*Crassostrea gigas*	0	F: TGATTCTGTGCCCTACTACTTG R: GAGATGGGTGATTGGTGAAG
ABCA3 isoform X2	1808	KF906266	ABCA3 (XP001851801)	*Culex quinquefasciatus*	0	F: TAGTTATGACACGGAGGTTGC R: TGAATAGTTGGTATGAACAGGG
ABCA5	1781	KF906267	ABCA5 (XP001607492)	*Nasonia vitripennis*	0	F: GACAGCAATCAGATGGAGGA R: CTTTCTTCCATTCCTCTGATTC
ABCA13	3124	KF906268	ABCA13 (EFN62269)	*Camponotus floridanus*	0	F: AGGTTTGTCTGAGGATGCTG R: GTATTTTGGGTCAATGTGCC
ABCB1 (Full transporter)	1361	KF906269	ABCB1 (AFS49708)	*Tigriopus japonicus*	0	F: GTGATGATTATTCTCTTTGGTGAC R: ATTGATTGCTGGAGTGTCGT
ABCB6 (Half transporter)	827	KF906270	ABCB6 (XP003485185)	*Bombus impatiens*	0	F: CCTTATCAAATGCTTGGGTC R: AGAATCCAAGTTGAATACACCC
ABCB7 (Half transporter)	692	KF906271	ABCB7 (XP001813375)	*Tribolium castaneum*	0	F: AGCCTAAAGTCCAGAATAAAGTG R: CAAACTGAGTCCGTTCAAGATA
ABCB8 (Half transporter)	676	KF906272	ABCB8 (EKC24099)	*Crassostrea gigas*	0	F: TTATTCAAGGCTTTCCAGACA R: GAATGGTCGGATTTTTGAGTA
ABCB10 (Half transporter)	665	KF906273	ABCB10 (XP005102782)	*Aplysia californica*	0	F: ACTTCGGCAGGATTTATTTG R: GTTGCGTGTCTGATGAAAGTC
ABCC1 isoform X1	1493	KF906274	ABCC1 (XP003243122)	*Acyrthosiphon pisum*	0	F : ACGGGAAGTATCATCAATCG R: GATGACAATGAGGACGGATG
ABCC1 isoform X2	1515	KF906275	ABCC1 (XP001604021)	*Nasonia vitripennis*	0	F: TTATCCTTCCAGTTATTGACCTT R: AGCAGAGAGCACACACATAGG
ABCC1 isoform X3	1497	KF906276	ABCC1 (XP003426122)	*Nasonia vitripennis*	0	F: TCATACTCAGTTACTATCCTCTT R: AAGGTCAGCAAGGATGGATA
ABCC1 isoform X4	1513	KF906277	ABCC1 (XP005176239)	*Musca domestica*	0	F: GGGGAAACTGTGAATCTTATGT R: TAGGAAAGCCAAGGACAAGA
ABCC1 isoform X5	1509	KF906278	ABCC1 (XP001604021)	*Nasonia vitripennis*	0	F: GTGTCAATCGTAACATTCCGT R: GTTTTCACGAAGAGGAGGATT
ABCC1 isoform X6	1476	KF906279	ABCC1 (XP003243122)	*Acyrthosiphon pisum*	0	F: CAGTGCCACAGTTTCTACCAT R: ACAACTTCAGACTCTTCCGATAG
ABCC1 isoform X7	1533	KF906280	ABCC1 (XP001604021)	*Nasonia vitripennis*	0	F: TGATGAAGAGGCTATGATTGG R: AGAGGAGAAACGAGATAACGC
ABCC1 isoform X8	1436	KF906281	ABCC1 (XP006119649)	*Pelodiscus sinensis*	0	F: TCAAGAACAAGGACGAGAGG R: CTTCAATGTTTCGGTATCCC
ABCC1 isoform X9	1611	KF906282	ABCC1 (XP003426121)	*Nasonia vitripennis*	0	F: TCTCTTGGTTTACGGGTTTG R: GAAACACGAAGCGACTCATC
ABCC1 isoform X10	1523	KF906283	ABCC1 (XP001604021)	*Nasonia vitripennis*	0	F: CAACGAGAGTCCAAACGAAC R: GGAAACAAGTGGTGAAATCG
ABCC2 isoform X1	1346	KF906285	ABCC1 (XP004770550)	*Mustela putorius furo*	7E-116	F: AGTATTCTCTCTCCGCATTCTC R: TGCCAATAAGGAGAGTGTAATG
ABCC2 isoform X2	1380	KF906286	ABCC1 (XP004580304)	*Ochotona princeps*	0	F: ATTAGCCAGTAAGAAGTTGAAGG R: GTCGTTCCCAAACATTCATC
ABCC4	1386	KF906287	ABCC4 (XP002939329)	*Xenopus tropicalis*	0	F: AGTATTCCTAAGTTGGTTTGTGG R: ACGGATGATGAGTGAAGGTG
ABCC5	1422	KF906288	ABCC5 (XP005051248)	*Ficedula albicollis*	0	F: TCAACAACCCTACACTTCCTG R: GCAGACAAGAGCGATGATTT
ABCC7	1496	KF906289	ABCC7 (ELT97351)	*Acromyrmex echinatior*	0	F: CAAATGACCCTCAACGAACT R: AGATACCGATGGAGAAAAACC
ABCC9	1949	KF906290	ABCC9 (XP003698789)	*Apis florea*	4E-135	F: TTGGGTCTGTTATCATTCTGG R: ACGAGTTGAAGCAAGGTGAG
ABCC-like	1296	KF906284	ABCC1 (XP001341895)	*Danio rerio*	0	F: GAAAAACGATAAGGCTGGTG R: CTATCCCAACTTTCTGACCG
ABCD2	736	KF906291	ABCD2 (XP001943381)	*Acyrthosiphon pisum*	0	F: GATGACGAAGAGACGACAATG R: GTCGTTGAAGCCTTTCTATGA
ABCD4 isoform X1	603	KF906292	ABCD4 (XP004699087)	*Echinops telfairi*	4E-153	F: AATGGAACTGGTCTGATGTGA R: ACAAGGACAAGGCTGAAGTG
ABCD4 isoform X2	608	KF906293	ABCD4 (XP005021486)	*Anas platyrhynchos*	7E-169	F: ATCAGTTACTACACCTATTCGGC R: CACGGGCGACATAAGTAGTT
ABCE1	611	KF906294	ABCE1 (XP003690922)	*Apis florea*	0	F: ACGAACCCTCTGCTTATTTG R: GCTCCACGATAAAACCTGTC
ABCF1	820	KF906295	ABCF1 (XP004531105)	*Ceratitis capitata*	0	F: CGAGAGTGAAGATGAAGACCA R: ATTTCTTGCCTTTCTTGTCCT
ABCF2	642	KF906296	ABCF2 (XP003403104)	*Bombus impatiens*	0	F: GATTTGGAGGCTTGTGTCTG R: TCCAGATGAATGATGTTGCTAC
ABCF3	711	KF906297	ABCF3 (XP004923267)	*Bombyx mori*	0	F: AATGTTGCTCAAGAAGATGGA R: GGCTTTGGCATCTTTTTTAC
ABCG_125057	687	KF906298	ABCG (XP003489743)	*Bombus impatiens*	0	F: TTATCGGCACACTCACTCCT R: ACACTATCCACACGAGCCAT
ABCG_90506	659	KF906299	ABCG (EFX83517)	*Daphnia pulex*	0	F: GGAAACGGTAATGAATCTGG R: GGATTCTTGATAACAGATAACCAG
ABCG_75152	651	KF906300	ABCG (XP_003398687)	*Bombus terrestris*	0	F: GTTACTAAAGAACCTCTCATTGC R: CCTTCTTGATTATTTTCTTGTCC
ABCG_105304	656	KF906301	ABCG (NP001034521)	*Tribolium castaneum*	0	F: ATGTATTCACACGGGAGACG R: TACACAGCACGACAAATCCA
ABCG1	767	KF906302	ABCG1 (EGI70628)	*Acromyrmex echinatior*	0	F: TGAGACAAACTTGAAAGAGAAAC R: ACACCTTCGGAGTTGTATGC
ABCG5 isoform X1	656	KF906303	ABCG5 (XP003748075)	*Metaseiulus occidentalis*	1E-170	F: ATTACCAACATCTCTCAAACACC R: TCCACTTAGCGACCTCCTTA
ABCG5 isoform X2	822	KF906304	ABCG5 (XP003694147)	*Apis florea*	2E-141	F: CTATCCATCCACCAACTTACG R: CAAGGATAGTCAATGAAGGCA
ABCH_139282	768	KF906305	ABCH (EFX78467)	*Daphnia pulex*	0	F: TGAGAAAGTTGCCTCTGGTC R: GATGGCTTGAATGCTGTAGTC
ABCH_88708	788	KF906306	ABCH (EFX78468)	*Daphnia pulex*	0	F: CAAACACAGCCATCTCCTTC R: GTAGATGGCACCTTTTGGAA
ABCH_103004	778	KF906307	ABCH (EFX78468)	*Daphnia pulex*	0	F: GTTGTGTTTGGTGTTCCTCC R: TTGAGTGGCATCTTGTTGAGT
ABCH_55580	723	KF906308	ABCH (EFX78468)	*Daphnia pulex*	0	F: ATCTATCAACTGCCCACTGC R: TTCCCATCTCCTCAGTGTTG
ABCH_68644	714	KF906309	ABCH (EFX71377)	*Daphnia pulex*	4E-88	F: TCCTCGGTTATTGATTCTCG R: CAAATAGTGTGTGGTGATGAGG

**Figure 2:**
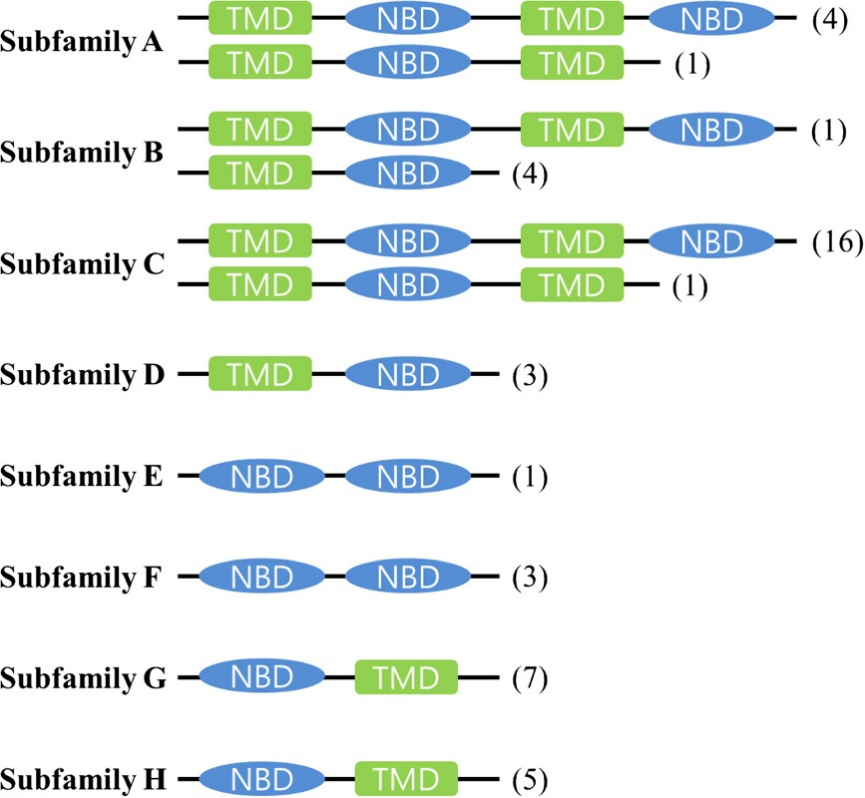
**Conserved domain analysis of 46** ***T. japonicus*** **ABC transporters.** ABC in an ellipse means ATP-binding cassette (referred to as nucleotide binding domains; NBDs), and TM in a rectangle represents transmembrane domains (TMDs). Numbers in brackets refer to the number of ABC proteins in each subfamily.

**Figure 3:**
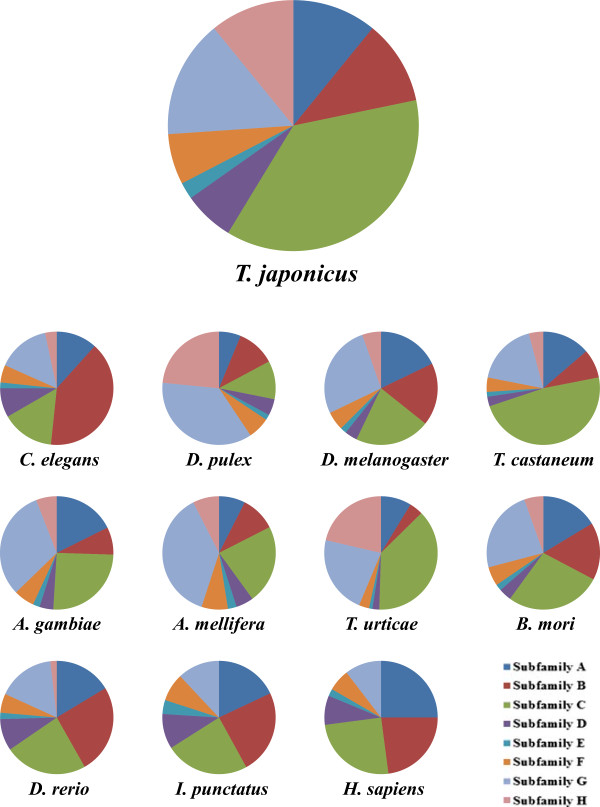
**Comparison of the number of genes in each subfamily of ABC transporters between** ***T. japonicus*** **and other animals.**

All *T. japonicus* ABC transporters possessed one or two conserved NBDs in their amino acid sequences (Figure 2). Of them, there were 21 full transporters (46%) and 19 half transporters (41%). Particularly, the ABCA, ABCB, and ABCC subfamilies contained full transporters, while the ABCB, ABCD, ABCG, and ABCH subfamilies possessed half transporters. However, the ABCE and ABCF subfamilies did not contain TMDs. In *T. japonicus*, the vast majority of ABC transporters are in the ABCC subfamily (37%), followed by the ABCG subfamily (15%) (Figure 3, Table 2). The composition rate and percentage rank of *T. japonicus* ABC transporters were similar to those of *T. castaneum*, *T. urticae*, and *B. mori*, which have large numbers of ABCs in the ABCC (48, 38, and 27%) and ABCG (18, 22, and 24%) subfamilies, respectively. Overall, the compositions of all ABC transporters showed different distributions for each animal subfamily analyzed in this study (Figure 3, Table 2). However, interestingly, similar percentage compositions were observed in the subfamilies of the insect ABCC and ABCG subfamilies, while high numbers of ABC proteins were observed in the ABCB and ABCC families of two fish species and humans (Figure 3), suggesting that the lineage-specific diversity of ABC-related pathways has accumulated over evolution in a wide range of animal taxa. It also demonstrates that each animal has evolved a unique function for ABC transporters in response to different environmental and ecological conditions. Incomplete genomic sequence databases of certain animals affects our knowledge of the annotation of ABC transporters and comparative composition analysis; only draft genome information is available for several animals including insects [9].

**Table 2:** **Number of ABC transporters in different animals and the composition in each ABC subfamily**

	***C. elegans*** [6]	***T. japonicus***	***D. pulex*** [7]	***D. melanogaster*** [1]	***T. castaneum*** [12]	***A. gambiae*** [8, 9]	***A. mellifera*** [9, 10]	***T. urticae*** [11]	***B. mori*** [9]	***D. rerio*** [13]	***I. punctatus*** [57]	***H. sapiens*** [1]
A	7	**5**	4	10	10	9	3	9	9	9	9	12
B	24	**5**	7	10	6	5	5	4	9	14	12	11
C	9	**17**	7	12	35	13	9	39	15	13	12	12
D	5	**3**	3	2	2	2	2	2	2	5	5	4
E	1	**1**	1	1	1	1	1	1	1	1	2	1
F	3	**3**	4	3	3	3	3	3	3	3	4	3
G	9	**7**	24	15	13	16	15	23	13	9	6	5
H	2	**5**	15	3	3	3	3	22	3	1	0	0
Total	60	**46**	65	56	73	52	41	103	55	55	50	48

### ABCA

Five ABCA proteins were identified in *T. japonicus* RNA-Seq and genome databases and were classified with phylogenetic analysis to provide their annotations (Additional file 2, Table 1). To date, the ABCA subfamily has been characterized by NBD(s), a conserved regulatory domain with multiple phosphorylation sites, and a large extracellular loop between the first two transmembrane (TM) helices in the TMD [19]. In *T. japonicus*, there are 4 ABCA full transporters (two TMDs and two NBDs) and one single NBD-containing ABCA protein (two TMDs and one NBD; ABCA3 isoform X1) (Figure 2). The human ABCA subfamily has full transporters, but no ABCA protein has been identified in yeast [1, 20]. The water flea *D. pulex* contains four ABCA full transporters [7]. The ABCA subfamily in the silkworm consists of two full transporters, one half transporter, and three single NBD-containing incomplete ABC proteins [9]. Thus, in animals, ABCA proteins show great domain variation among species due to duplication and insertion and/or deletion via evolution.

Regarding the potential function of the ABCA subfamily, there is still little information on the function of ABCA proteins in invertebrates. Mammalian ABCA proteins are involved in control of the cellular lipid transport processes and have several potential roles in lipoprotein biogenesis, lung surfactant production, retinal integrity, and skin lipid barrier formation [21]. In invertebrates, Broehan *et al.*[12] used the flour beetle *T. castaneum* to perform an RNAi screen for *TcABCA-9A* and *TcABCA-9B* and found ~30% stage-specific mortality with severe defects in the development of wings and elytra, leaving the functional role unknown in invertebrate ABCA proteins. Comparison of domains and other components among arthropods is necessary to better understand the “gain/loss of gene function” scenario of the ABCA subfamily from the evolutionary perspective of crustaceans, including copepod and *Daphnia* to insects and vertebrates.

### ABCB

The ABCB subfamily is divided into a group of full transporters (two TMDs and two NBDs) and a group of half transporters (one TMD and one NBD). In *T. japonicus*, five ABCB proteins were identified, and each had distinct clades with regard to phylogenetic distance (Additional file 3, Table 1). The *T. japonicus* ABCB subfamily consisted of one full transporter (ABCB1) and four half transporters (Figure 2). The TJ-ABCB1 protein, a P-glycoprotein (P-gp), is most well-characterized member of the mammalian multidrug transport process. As shown in Additional file 3, the *TJ-ABCB1* gene was clearly clustered with other *ABCB1/P-gp/MDR1* genes in phylogenetic analysis, suggesting that the TJ-ABCB1 protein is involved in biochemical defense against diverse molecules including xenobiotics, as suggested in other invertebrates. While we identified only a single *P-gp* gene in *T. japonicus,* Sturm et al. [7] reported three ABCB full transporters (*mdr49*, *mdr50*, and *mdr65*) and two full transporters in the fruit fly *D. melanogaster* and the water flea *D. pulex*, respectively. The silkworm *B. mori* also has multiple types of *ABCB1/P-gp/MDR1* genes [9].

The *T. japonicus* half transporters (TJ-ABCB6, TJ-ABCB7, TJ-ABCB8, and TJ-ABCB10 proteins) form a robust clade (Additional file 3), indicating that these proteins have clear orthologous relationships with those of the fruit fly and human, as shown in a previous study with *D. pulex*[7]. This also suggests that the four *T. japonicus* half transporter proteins have similar roles to the ABCB half transporter proteins of arthropods and humans. In humans, these ABCB transporter proteins are mitochondrial transporters with roles in porphyrin transport, Fe-S cluster assembly, and modulation of mitochondrial reactive oxygen species [22–25]. Since these processes are common to all eukaryotic systems, conservation of these proteins in *T. japonicus* is to be expected. These proteins may have additional roles because the ABCB half transporter in *D. melanogaster,* CG4225, is orthologous to TJ-ABCB6 and has been shown to confer tolerance to cadmium [26].

### ABCC

The ABCC subfamily consists of the chloride channel cystic fibrosis transmembrane conductance regulator (CFTR), membrane-bound sulfonylurea receptors (SUR1 and SUR2), and several multidrug resistance-associated proteins (MRPs) that are involved in transporting drugs, ions, toxins, and endogenous compounds [1, 27, 28]. In *T. japonicus*, we identified 17 ABCC transporters consisting of 16 full transporters (two TMDs and two NBDs) and one short form transporter (two TMDs and one NBD) (Figure 2, Additional file 4, Table 1). The *D. pulex* and *D. melanogaster* ABCC subfamilies contained seven full transporters as in the human ABCC subfamily [7], while other insects such as *A. gambiae*, *A. mellifera*, *B. mori*, and *T. castaneum* showed different numbers of full, half, and incomplete ABCC transporters [9]. In the *T. japonicus* ABCC subfamily, 10 TJ-ABCC proteins were identified as ABCC1 isoforms, suggesting that the TJ-ABCC1 subfamily is highly divergent based on adaptation to environmental stress. All TJ-ABCC1 isoforms were clustered in a clade with human “long” MRPs and *D. melanogaster* CG6214, as shown in Additional file 4.

One of the ABCC subfamilies, TJ-ABCC4, was clustered with human ABCC4 (MRP4), which is involved in removal of a wide range of endogenous and xenobiotic organic anionic compounds from the cell with a potential function in extracellular signaling pathways [29]. In addition, the TJ-ABCC9 protein clustered with human SURs (ABCC8 and ABCC9) and the fruit fly SUR protein (CG5772), indicating that TJ-ABCC9 is a SUR homolog.

### ABCD

Peroxisomes are involved in a variety of metabolic processes including β-oxidation of fatty acids. The ABCD subfamily members are peroxisomal transporters, and they actively participate in the transport of fatty acids and/or acyl-CoA with different substrate specificities in mammalian peroxisomes [30]. To date, in diverse animal taxa, all ABCD subfamilies are known to be half transporters and dimerize primarily as homodimers to function as transporters at the cellular level. In *T. japonicus*, we annotated three TJ-ABCD transporters including one ABCD2 and two isoforms of ABCD4 (Figure 2, Additional file 5, Table 1). There is no information on function in the invertebrate ABCD subfamily, but in humans, they have roles in fatty acid metabolism and pathological processes of neurodegenerative disease [31]. Based on phylogenetic analysis, each TJ-ABCD transporter showed a clear homologous relationship to that of humans, indicating that such genes may share similar functions across the animal kingdom. To date, copepod fatty acids and their metabolites have been intensively studied as a useful food source for fish larvae. Interestingly, copepods accumulate wax esters in their oil sac for energy reservation and buoyancy, and the high contents of fatty acids are a primary food source for fish, sea birds, and whales [32, 33]. These wax esters are used for gonadal development in female copepods and for physical activity in males [33]. Teerawanichpan and Qiu [34] identified three fatty acyl-CoA reductases that are responsible for *de novo* synthesis of fatty alcohol moieties of wax esters in the marine copepod *Calanus finmarchicus*. Therefore, general roles and/or specific functions of the ABCD subfamily may be highlighted in copepods, as their nutritional aspects are very important in aquaculture.

### ABCE and ABCF

The subfamilies of ABCE and ABCF contain a pair of linked NBDs and no TMD segment, which is commonly found in other members of the subfamily. Therefore, both subfamilies are known to play a biological role that does not include transmembrane functions. This unique structural character was also observed in both ABCE and ABCF subfamilies of *T. japonicus* (Figure 2). Phylogenetic analysis revealed that all TJ-ABCE and ABCF transporters formed a unique cluster similar to those of insect and human (Additional file 6), suggesting that they have conserved roles during evolution. In the case of the *T. japonicus* ABCE subfamily, a single transporter (TJ-ABCE1) was identified, as shown in other ABC superfamilies of diverse animal taxa (Table 2). The ABCE transporters are believed to be involved in inhibition of ribonuclease L, translation initiation, ribosome biosynthesis, tumor cell proliferation, and anti-apoptosis [35, 36]. In invertebrates, the ABCE transporter has not been well characterized, but a recent report showed that RNAi-mediated knockdown of a flour beetle *ABCE* gene (*TcABCE-3A*) caused defects in pupation, resulting in significant mortality in penultimate larvae of the flour beetle *T. castaneum*[12]. Moreover, eukaryotic ABCE1 contains a unique structural organization in the N-terminal region with eight conserved cysteines, which are predicted to coordinate diamagnetic iron-sulfur clusters (Fe-S)^2^[37]. Iron-sulfur clusters constitute an ancient prosthetic group that is found in diverse proteins from all living animals, and they are essential for the enzymatic function involved in electron transport for a variety of cellular processes. In the TJ-ABCE1 protein, there are two essential iron-sulfur clusters with different electronic environments, one ferredoxin-like (CPX_24_CX_2_CX_2_C; Cys at positions 4–7) and one unique ABCE1-type cluster (CXPX_2_CX_3_CX_3_CP; Cys at positions 1, 2, 3, and 8), suggesting that the TJ-ABCE1 protein is evolutionary conserved across the animal kingdom and is essential for copepod life. Thus, in copepods, ABCE1 has an obvious role in electron transport as well as potential roles in a wide range of activities including electron transport in respiratory chain complexes, regulatory sensing, photosynthesis, DNA repair, protein stability, and nucleic acid binding and modification [37–39].

In the case of the ABCF subfamily, three ABCF proteins were annotated and showed well-supported sister clades (Additional file 6, Table 2). To date, in most eukaryotes, including insects, three ABCF proteins have been identified [9, 10, 16], suggesting that ABCF subfamilies are highly conserved during evolution, as confirmed in *T. japonicus*. In yeast and human, ABCF proteins participate in gene regulation systems and ribosome assembly [14, 40], while RNAi-mediated knockdown of *TcABCF-2A* induced 100% mortality in penultimate larvae of the flour beetle *T. castaneum*[12]. Based on the conserved domain structure, high similarity in amino acids, and distinct separation in phylogenetic analysis of ABCF subfamilies within animal taxa, we suggest that TJ-ABCF transporters have a potential role in translational regulation and/or cell viability.

### ABCG

In the copepod *T. japonicus*, seven ABCG transporters were identified (Table 1). All *T. japonicus* ABCG proteins are half transporters that possess a reverse domain architecture (NBD-TMD), as shown in most metazoan species (Figure 2), while fungi and plants have additional full transporters [41, 42]. As described in the ABCC subfamily, arthropods including *T. japonicus* contain a high composition of the ABCG subfamily, while humans and two species of fish have a variety of subfamilies in the ABCA, ABCB, and ABCC subfamilies (Figure 3). These different subfamily patterns in several taxa suggest that extensive gene duplication is lineage-specific for different ABC subfamilies within arthropods and vertebrates (e.g., mammals, fish). In arthropods, this hypothesis was confirmed by comparison of the localization of paralogous or orthologous genes on chromosomes and with phylogenetic analysis [7, 9–11]. For example, in the water flea *D. pulex,* Sturm *et al.*[7] showed the presence of pseudogenes of the ABCG subfamily by comparing expressed sequence tags (ESTs) with the gene loci of each putative ABCG proteins through *in silico* analysis.

Among invertebrates, the functions of several ABCG proteins were first characterized in *Drosophila* as pigment precursor transporters (*brown*, *scarlet*, and *white* genes) into pigment cells [43, 44]. In silkworm, Kômoto *et al.*[45] reported that a single-base deletion in exon 2 and a premature stop codon at the 5′ end of exon 3 of a silkworm homolog (*Bmwh3*) of the *D. melanogaster white* gene caused white eyes, white eggs, and translucent larval skin in the silkworm *w-3*^*oe*^ mutant. In phylogenetic analysis, four *T. japonicus* ABCG proteins showed an orthologous relationship with nine *Daphnia* ABCG proteins and the *Drosophila* white protein, while no orthologous clone in *T. japonicus* was found for *Drosophila brown* or *scarlet* proteins (Additional file 7). A functional study with orthologs of the *Drosophila* white protein is necessary for a better understanding of copepod sensing mechanisms, as copepods have a single eyespot sensing illumination intensity, although we were not able to identify additional transporters for eye pigment transfer in *T. japonicus*. In addition, TJ-ABCG1 was in a clade with human ABCG1/ABCG4 and the *Drosophila* atet protein, which is expressed in the trachea [46]. In humans, ABCG1 plays a role in controlling sterol homeostasis [47, 48], while several ABCG full transporters of the Indian-rock oyster *S. forskali* and the white shrimp *Litopenaeus vannamei* are involved in fungicide resistance and detoxification [49–51]. In *D. melanogaster*, the CG3327 transporter (also called E23, early gene at 23) is believed to be induced by 20-OH ecdysone (20E), potentially through a 20E regulation mechanism [52], but the orthologous sequence was not identified in *T. japonicus*. Liu *et al.*[9] showed that 20E treatment regulated mRNA expression of the midgut-specific silkworm *ABCG* genes, suggesting that midgut-specific *ABCG* genes are closely related to 20E. Future studies should focus on examining similar or compensatory function with identification of the transporter gene for 20E regulation in *T. japonicus*, as ecdysone has a conserved role in crustaceans [53]. In addition, there is molecular and biochemical evidence for an ecdysone receptor and its putative involvement in the ecdysone-triggered metabolism of *T. japonicus*[54].

### ABCH

The ABCH proteins are an interesting ABC subfamily that is only annotated in arthropods and zebrafish [7, 9, 10, 12, 13, 55] and has not yet been identified in fungi, plants, *C. elegans*, or mammals [1, 6, 41, 42]. There are controversial reports on the presence of the ABCH subfamily in fish. After annotation of the ABCH proteins in zebrafish, Popovic *et al.*[56] showed a potential sequence of the ABCH subfamily in the green spotted pufferfish *Tetraodon nigroviridis.* However, Liu *et al.*[57] recently reported that the ABCH subfamily was identified in zebrafish but not in other fish such as catfish, medaka, fugu, stickleback, tetraodon, tilapia, cod, or coelacanths. Therefore, further analysis on evolutionary deletion or insertion of ABCH proteins may be helpful to clarify the situation in fish, especially since the ABCH subfamily was not identified in coelacanths, the most primitive fish. Regardless of the absence of the ABCH subfamily in fish aside from zebrafish, five ABCH proteins were identified as half transporters in *T. japonicus* (Figure 2, Table 1). Phylogenetic analysis showed that *T. japonicus* is a distinct clade of the ABCH subfamily (Additional file 8), indicating that this subfamily experienced lineage-specific accumulation by gene duplication, as suggested in the polyphagous spider mite [11]. However, Liu *et al.*[9] mentioned that insect ABCH subfamilies originated from a common ancestor based on the phylogenetic relationship, while this has not been confirmed in copepods or cladocerans. In fact, of the eight ABC subfamilies, ABCH proteins in the subfamily consist of many genes, as shown in *D. pulex* and *T. japonicus*, and these are located in different clades. As of yet, the physiological function of invertebrate ABCH proteins remains unclear, but several valuable reports have highlighted potential roles in insects. In *Drosophila* ABCH proteins, Dermauw *et al.*[10] summarized differentially expressed profiles of the *CG9990* gene in different tissues and its potential role in survival using an RNAi-aided gene silencing screening method. Broehan *et al.*[12] showed using RNAi-mediated knockdown of the *TCABCH-9C* gene in *T. castaneum* that one of the ABCH proteins is a lipid transporter required for the maintenance of the waterproof barrier of the epicuticle.

### Transcriptional expression of all ABC transporters in developmental stages

**Figure 4:**
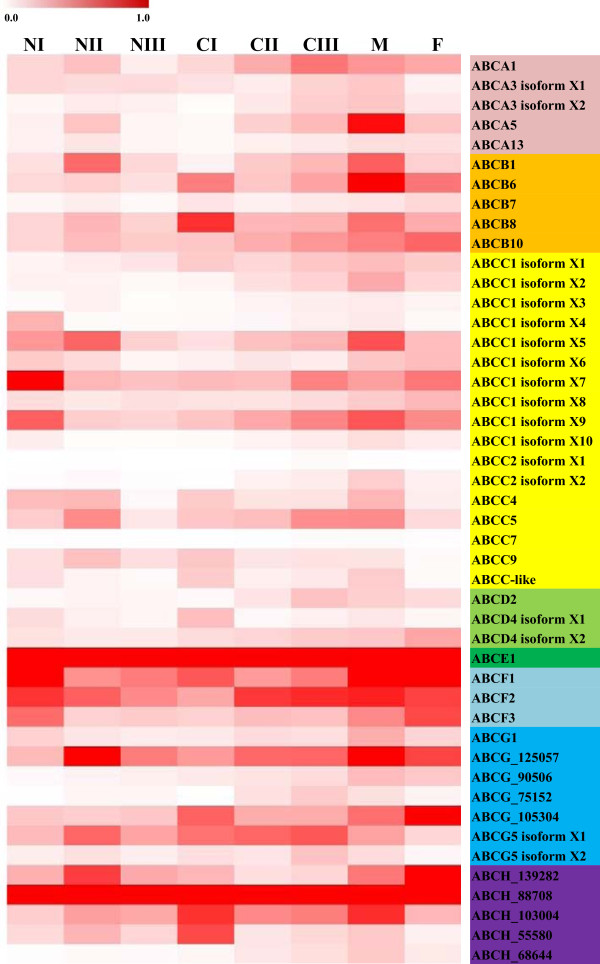
**Temporal transcriptional expressions of 46** ***T. japonicus*** **ABC transporters in different developmental stages (N, nauplius; C, copepodid; M, male; F, female).**

In *T. japonicus*, we measured temporal transcriptional expression of all ABC transporters during developmental stages using a real-time RT-PCR (Figure 4). Developmental stages and the morphological phenotype in each stage are shown in Additional file 9. Overall, expression intensity was high in the ABCE to ABCH subfamilies (Figure 4, Additional file 10). Interestingly, *ABC* genes of these four subfamilies are translated into short amino acid sequences and are composed of a unique domain structure, as described in each section. They are likely involved in cellular and biological processes other than transport. Therefore, we predict that these subfamilies individually play a role in the molecular physiology of different developmental stages of *T. japonicus*. Regarding stage-preferential transcript profile (Additional file 11), dramatic transcriptional change was not observed, but relatively low transcript intensities were observed in the NIII stage. The NIII stage (collecting naupliar stages 5 and 6) is important for molting prior to the copepodid stages. Diverse physiological and motional changes have been observed during the molting stage in copepods [58–60]. The naupliar stages are more sensitive to diverse environmental conditions including toxicants than are copepodid and adult stages [61–63]. Therefore, we concluded that several physiological metabolisms are highly activated in this stage, although this needs to be verified in future studies.

In all developmental stages of *T. japonicus*, the *TJ-ABCH_88708* and *TJ-ABCE1* genes are highly expressed (Additional file 10). However, in invertebrate development, the physiological or molecular function of ABCH remains generally unknown. In *D. melanogaster*, Zhang *et al.*[64] showed that one ABCH subfamily gene (*CG9990*) is involved in mortality, demonstrated by RNAi-mediated silencing. In *T. castaneum*, Broehan *et al.*[12] reported that injection of *TcABCH-9C* gene-specific dsRNA arrested development and caused 100% mortality with morphological changes in the cuticle such as desiccation and shrinkage during the quiescent stage. Moreover, *TcABCH-9C* gene knock-down reduced the number of laying eggs in adult *T. castaneum* and also caused failure to hatch for all eggs. Cuticles are essential in copepods and provide an important interface against the environment as a skeletal structure. The cuticle can also act as a defense barrier against detrimental conditions such as pathogens, osmotic shock, and a number of environmental toxicants [65]. Therefore, it is likely that the *TJ-ABCH_88708* gene is involved in development-related metabolism such as a lipid transporter to the cuticle.

ABCCs showed differential expression patterns in developmental stages, and isoforms of ABCC1 and ABCC2 also showed unique transcriptional profiles (Figure 4). While their roles in the detoxification process have been noted, as ABCCs/MRPs share substrates for the ABCB1/P-gp/MDR1 protein and eliminate xenobiotics or metabolites by transporting glucuronate, sulfate, and glutathione (GSH) conjugates [50, 66–68], several studies have suggested their participation in early cell division of embryos, development, oocyte maturation, and general physiology [10, 69, 70]. Based on the unique divergence of ABCCs in *T. japonicus*, we suggest that each isoform evolved as a new transporter for adaptation and/or speciation, even though pseudogenes should be confirmed with genomic structure comparison and functional characterization.

Three *T. japonicus* ABCDs showed slight changes in entire developmental stages (Figure 4). Since these ABCDs are homologous to those of mammal and insect transporters, similar functions such as roles in fatty acid metabolism can be inferred. Subsequently, modulation of ABCD transporters may be directly related to a variation in wax content and composition in different developmental stages of copepods [71].

Regarding the highly expressed *ABCE1* gene in *T. japonicus*, similar transcriptional profiles were observed in several insects, but there is limited information on the function of the ABCE subfamily in invertebrates. Liu *et al.*[9] reported that the silkworm *ABCE* gene (*BmABC010129*) was highly expressed in the EST information. In the polyphagous spider mite *T. urticae*, one of the ABCE subfamily members (*tetur30g01400* gene) showed very high expression in all developmental stages [11], while the *TcABCE-3A* transcripts in the flour beetle were highly abundant throughout all developmental stages [12]. In eukaryotes, the ABCE protein generally acts as a catalyst in the initiation and termination of translation and ribosome recycling, as described in the above ABCE section. Thus, we assume that ubiquitous TJ-ABCE1 plays a fundamental role in developmental processes in *T. japonicus*. In *T. castaneum*, injection of *TCABCE-3A*-specific dsRNA into penultimate larvae caused a lethal phenotype with 100% mortality, and the injection of *TCABCE-3A*-specific dsRNA into pre-pupae led to defects in pupation and 100% mortality [12]. Taken together, our results suggest that the ABCE transporter is essential for normal development of *T. japonicus*, as ABCE subfamilies are highly evolutionarily conserved with regard to both gene structure and role over all animal taxa [37, 72].

## Conclusion

Only a few genomes have been sequenced in crustaceans, resulting in a lack of comparative genomic information, particularly for large gene families. In this study, we conducted a genome-wide analysis of the entire ABC transporters in the intertidal copepod *T. japonicus* and annotated and characterized 46 *ABC* genes as a first report in copepods. Our analysis provides new insight into the diversity of the entire ABC subfamily in copepods compared with all arthropods. We anticipate that a functional study in the near future will elucidate the molecular and physiological functions of each ABC transporter in this species. The descriptive study of the *T. japonicus* ABC superfamily by genome sequencing, a genome-wide omics approach, and *in silico* analysis will reveal the evolutionary effects of genome duplication, particularly using the gene superfamily in *T. japonicus*.

## Methods

### *T. japonicus*culture and maintenance

The copepod *T. japonicus* was originally collected from a single rockpool at Haeundae beach (35°9′29.57″N, 129°9′36.60″E) in Busan (South Korea) in 2003; since then, we have continuously cultured them in a laboratory (the number of generation times ≈ 285; Sungkyunkwan University, Suwon, South Korea) with filtered artificial sea water (TetraMarine Salt Pro, Tetra™, Cincinnatti, OH, USA) adjusted to 25°C and a photoperiod of 12 h:12 h light/dark with a salinity of 30 practical salinity units (psu). The copepods were fed green algae, *Chlorella* sp. (approximately 6 × 10^4^ cells/ml). Identification of *T. japonicus* was based on morphological characteristics and the sequence identity of the universal barcode marker, the mitochondrial DNA *CO1* gene.

### Retrieval and annotation of whole ABC transporters

A *T. japonicus* genomic DNA database was constructed as shown in our previous study [17]. Briefly, we mechanically sheared the genomic DNA of *T. japonicus* into fragments and created a genomic DNA library according to the manufacturer’s instruction (Roche Applied Science, Genome Sequencer System). Sequencing (by GS-FLX, Solexa, and Solexa mate pair), assembly, gene annotation, and GO analysis were performed at the NICEM, Seoul National University (Seoul, South Korea). Finally, we obtained a scaffold number of 60,979 (scaffold length 174,022,895 bp; average read length 2,854 bp; N50 = 6,335 bp) covering approximately 174 Mb of *T. japonicus* genomic DNA. For RNA seq, total RNAs of *T. japonicus* were sequenced using an RNA-seq platform (Illumina, CA, US; 59,983 assembled ESTs; total length 78.3 Mb; N50 = 2,319 as of Feb. 10, 2014) and then assembled with NGS Cell (Ver. 4.06 beta 67189, CLC Bio, MA, USA) and Velvet (EMBL-EBI, UK) [17] software. To obtain the sequence information of all ABC transporters in *T. japonicus* genomic DNA and transcriptome databases, the obtained contigs and clones after assembly as well as genomic clones were subjected to BLAST analysis to the non-redundant (NR) database at GenBank (ftp://ftp.ncbi.nlm.nih.gov/blast/db/FASTA/nr.gz). To confirm exon/intron boundary and start/stop codons, genomic structure was mutuallycompared between the genomic clone and transcript for each gene. Some ABC transporter sequences were subjected to 5′- and 3′-RACEs according to the manufacturer’s protocol (Invitrogen, Carlsbad, CA), as incomplete transcript information was observed in the 5′ and/or 3′ terminal region. Annotation and nomenclature of all *T. japonicus ABC* genes were completed based on amino acid sequence similarities in terms of *in silico* domain analysis compared to ABC superfamilies of other animals. All gene information was registered to the GenBank database, and the accession numbers of each gene are appended in Table 1.

### Phylogenetic analysis

To analyze the evolutionary placement of the copepod *T. japonicus ABC* clusters on the phylogenetic tree, we aligned them with those of other species at the level of the deduced amino acid sequence by ClustalX 1.83. Selection of the ABC superfamily from a representative animal in each animal taxon followed the criteria of Sturm *et al.*[7]. Entire *ABC* gene sets of yeast (*Saccharomyces cerevisiae*), worm (*Caenorhabditis elegans*), fruit fly (*Drosophila melanogaster*), and human (*Homo sapiens*) were obtained from the GenBank database. Gaps and sets with missing data were excluded from the analysis. The generated data matrix was converted to the nexus format and was analyzed with the Mr. Bayes v3.1.2 program using the general time-reversible (GTR) model. A total of 1,000,000 generations were conducted, and the sampling frequency was assigned as every 100 generations. After analysis, the first 10,000 generations were deleted as the burn-in process, and the consensus tree was constructed and then visualized with Tree View software of PHYLIP.

### Total RNA extraction and single-strand cDNA synthesis

The morphological characteristics and developmental stages of the copepod *T. japonicus* were analyzed based on the criteria of Raisuddin *et al.*[16] (Additional file 9). Whole bodies (≈500 individuals for each developmental stage of nauplius and copepodid; ≈ 300 adult individuals) were homogenized in three volumes of TRIZOL® reagent (Molecular Research Center, Inc.) with a tissue grinder and stored at −80°C until use. Total RNA was extracted according to manufacturers’ instructions and stored at −80°C until use. DNA digestion was performed using DNase I (Sigma, St. Louis, Mo). Total RNA was quantified by absorption of light at 230, 260, and 280 nm (A230/260, A260/280) using a spectrophotometer (Ultrospec 2100 pro, Amersham Bioscience). To verify no genomic DNA contamination, we loaded total RNA in a 1% agarose gel that contained ethidium bromide (EtBr) and visualized it on a UV transilluminator (Wealtec Corp., NV, USA). Subsequently, we loaded total RNAs in a 1% formaldehyde/agarose gel with EtBr staining in order to verify the total RNA quality and verified the 18/28S ribosomal RNAs integrity. After RNA quality was determined, single-stranded cDNA was synthesized from 2 μg of total RNA of each sample using oligo (dT)_20_ primer for reverse transcription in 20 μl reactions (SuperScript™ III RT kit, Invitrogen, Carlsbad, CA).

### Real-time reverse transcriptase-polymerase chain reaction (real-time RT-PCR)

Transcriptional levels of *T. japonicus ABC* genes in each developmental stage were validated using real-time RT-PCR. Primers for each gene were designed after comparing the exon/intron boundary to genomic DNA using GENRUNNER software (Hastings Software, Inc. NY, USA) and were confirmed by the Primer 3 program (Whitehead Institute/MIT Center for Genome Research). To determine the amplicon identity, all the PCR products were cloned into the pCR2.1 TA vector and sequenced with an ABI 3700 DNA analyzer (Bionics Co., Seoul, South Korea). Optimized conditions were transferred according to the following CFX96™ real-time PCR protocol (Bio-Rad, Hercules, CA, USA). A no template control (NTC) reaction was included in every run for each primer pair in order to exclude DNA contamination in buffers/solutions and to assess primer dimers. Also, genomic DNA contamination was tested by the inclusion of controls that omitted the reverse transcriptase enzyme from the cDNA synthesis reaction (no RT controls). To set an appropriate reference gene for the real-time RT-PCR as a preliminary experiment, reliability of 9 reference candidates was validated using intra- and inter-laboratory validation procedures in a multiplex PCR condition (tubulin α; glyceraldehyde 3-phosphate dehydrogenase, GAPDH; β-actin; DNA-directed RNA polymerase II subunit RPB2, POLR2B; glucose-6-phosphate dehydrogenase, G6PD; hypoxanthine phosphoribosyltransferase 1, HPRT1; TATA box binding protein, TBP; elongation factor 1 α, EF1α; 18S ribosomal RNA, 18S rRNA) at different developmental stages of *T. japonicus*. As a result, the *EF1α* gene showed the most stable expression pattern in all multiplex plates (data not shown). All real-time RT-PCR experiments were carried out in unskirted low 96-well clear plates (Bio-Rad, Hercules, CA, USA). A total of 2 μg of total RNA was used to synthesize cDNA for real-time RT-PCR. In each reaction, 0.2 μM of each forward and reverse primer for each *ABC* gene was employed (Table 1). Reaction conditions to detect specific PCR products were as follows: 94°C/4 min; 35 cycles of 94°C/30 sec, 55°C/30 sec, 72°C/30 sec; and 72°C/10 min. SYBR® Green (Molecular Probe Inc., Invitrogen). To confirm the amplification of specific products, cycles were continued to determine the melting curve under the following conditions: 95°C/1 min, 55°C/1 min, and 80 cycles of 55°C/10 s with 0.5°C increase per cycle. All PCR products were sequenced at Bionics Co. (Seoul, South Korea). SYBR Green (Molecular Probes Inc., Invitrogen) was used to detect specific amplified products. Amplification and detection of SYBR Green-labeled products were performed using the CFX96 real-time PCR system (Bio-Rad, Hercules, CA, USA). Data from triplicate experiments were expressed relative to expression of the internal control *EF1α* gene that was used to normalize for any difference in reverse transcriptase efficiency. Each transcriptional level was determined by the 2^-ΔΔC^t method [73]. For the time-course experiment, hierarchical clustering analysis was employed in order to prepare a heat map using MeV v.7.4 software (Dana-Farber Cancer Institute, MA, USA).

## Electronic supplementary material

Additional file 1: **Complementary DNA (cDNA) sequence for 46 full-length** ***ABC*** **transcripts.** (DOCX 73 KB)

Additional file 2: **Phylogenetic analysis of** ***T. japonicus*** **ABCA subfamily with those of other species using Bayesian method.** Numbers at branch nodes represent the confidence level of posterior probability. (PPTX 85 KB)

Additional file 3: **Phylogenetic analysis of** ***T. japonicus*** **ABCB subfamily with those of other species using Bayesian method.** Numbers at branch nodes represent the confidence level of posterior probability. (PPTX 95 KB)

Additional file 4: **Phylogenetic analysis of** ***T. japonicus*** **ABCC subfamily with those of other species using Bayesian method.** Numbers at branch nodes represent the confidence level of posterior probability. (PPTX 93 KB)

Additional file 5: **Phylogenetic analysis of** ***T. japonicus*** **ABCD and ABCE subfamilies with those of other species using Bayesian method.** Numbers at branch nodes represent the confidence level of posterior probability. (PPTX 66 KB)

Additional file 6: **Phylogenetic analysis of** ***T. japonicus*** **ABCF subfamilies with those of other species using Bayesian method.** Numbers at branch nodes represent the confidence level of posterior probability. (PPTX 82 KB)

Additional file 7: **Phylogenetic analysis of** ***T. japonicus*** **ABCG subfamilies with those of other species using Bayesian method.** Numbers at branch nodes represent the confidence level of posterior probability. (PPTX 101 KB)

Additional file 8: **Phylogenetic analysis of** ***T. japonicus*** **ABCH subfamilies with those of other species using Bayesian method.** Numbers at branch nodes represent the confidence level of posterior probability. (PPTX 67 KB)

Additional file 9: **Developmental stages of the intertidal hapacticoid copepod,** ***T. japonicus*** **.** Stages 1–6 are nauplius (N) stages and five stages in the second row represent copepodite (C) stages. Figure was modified from our previous publication (Seo et al., 2006). (PPTX 997 KB)

Additional file 10: **Result of gene-specific hierarchical clustering analysis with temporal transcriptional expressions of** ***T. japonicus*** **46 ABC transporters in different developmental stages (N, nauplius; C, copepodid; M, male; F, female).** (PPTX 193 KB)

Additional file 11: **Result of developmental stage-specific hierarchical clustering analysis with temporal transcriptional expressions of** ***T. japonicus*** **46 ABC transporters (N, nauplius; C, copepodid; M, male; F, female).** (PPTX 488 KB)

## Abbreviations

ABC: ATP-binding cassette
CFTR: Cystic fibrosis transmembrane conductance regulator
ESTs: Expressed sequence tags
GSH: Glutathione
MDR: Multidrug resistance protein
MRP: Multidrug resistance-associated protein
NBD: Nucleotide binding domain
NR: Non-redundant
P-gp: P-glycoprotein
psu: Practical salinity units
SUR: Sulfonylurea receptor
TAP: Transporters associated with antigen processing
TBT: Tributyltin
TMD: Transmembrane domains.
