# Supplementary material for: Genome-wide identification of whole ATP-binding cassette (ABC) transporters in the intertidal copepod Tigriopus japonicus
Source: BMC Genomics. 2014 Aug 5;15(1):651. doi: 10.1186/1471-2164-15-651 (PMC4247197; doi:10.1186/1471-2164-15-651)
Supplement: Supplementary file 1 — Additional file 1: Complementary DNA (cDNA) sequence for 46 full-length ABC transcripts. (DOCX 73 KB) [file 12864_2014_6676_MOESM1_ESM.docx]

>ABCA1

**ATG**AATTTCCTTCTCCAATTGAGGCTTCTCATCTGGAAGAACCTCATCCTACGCAAACGGAGCTGGGTTCGCTTGCTCATCGAAGTTCTTTGGCCGTTGTTTCTATTCATGATCCTCGTTTTGGTGCGATTGAGAGGATTACGGAAATACCATCACCAATGCTATTTTGACGAAAAGGCCATGCCTTCGGCAGGTCTTATGAGTTTCGCTCAAAGCTTCATTTGCACCTTCAATAACACGTGTCACATGGAAAATCGAGATGATTATACCCAAATCCAAACTTACAATGACTCTTTTATATCAGAACTTGTGAGAGACGTGGAGGTGGCTTTGCAAAGAAATTTCGACGAGGAACGTGTCGTTGCAGTGAGCGAGATCATCAAGGACCTTTACACTATTCAACAGTTGGGGGTGAAAGTGGGAAACTCGTTCAATAATGGGGAAGGTGTTCCAGGTCTTACTGTGGATCAAGTGAGTCAACTCATTAAGGATCCCAATGCCTTTGTCTTTGGGTTGCTTTCATGGGCCAACGAAAAGAACATCCCCCTCCAACCGAAGGTCATCCAAGGCCTCATGGCTGCCACGTTAAGGCCAGAAAACGTCTCGCCAACAGAACTAATCAATATGGTCACAGATCCAAGGACCGCGATATGCGATCCCGAACTCGTGAGTCGATTCATCGACGCCAAGAACTTGACCAATGAAGAAGTTCGGGAGAGCTTGAGACCGTTTTGCAATCTTAGTGTTGAAGATGCTTTGGAACTTTTGAATTTAGTTCAAGAGAACATTGATTGGACCCAGGTTCAAGACGAGGTGGATGAGTTTGCAATTGGCAATGCCAACGGACAACTCCATTTCAACGATTGGAAGCTCCTCATTCAAGTCAGTGAAGATGTCAGACGGGATTTGCTTGAAAAAGAGTCAATTAAAAAGGTCATCGGCGATGTCTCCAACTTTATGAAGGAGCTCACCGATCAAATGACAATGGAAGTGAACAACGAAACCTTGAGCAACAACTCTTTATTCACACGAAACATCTCGGAAAGTGGAAGATTGGCCTTTGCTGCCTTTCAACGTGTGGTTTGTGGTCGAAACATGTCCTCAGACCAACTTTTCAGTGATGGAGGGGCTGCGACAAGGTTCGACCAACTCCGTGATCAATTGAAGGAGAAGGATGAAGAAGTATTTTACTTGTATGACAACTCAACCAGTAAGGAATGTAACGATTTTATGAAGACCATGGAGGAAAATCGCCTCACCGGGTTTTTATGGGATCAAATGAAACCGTTCATACGAGGGAAAATCTTGTTTAGTCCGGACACGCCCGCTACCCGGAAATTGATGTCGTTGGTCAATGCCACGTTCAGTCCAATTGAGGATGTCCGAAGAGTCACTCAAATGTGGATCGACACTTACTCTGACCGAGTCCGAGGATTCTTTTTGGACAAAGAAAACCAAGAGTTTCTTAAGGAACAATTTACATCGGAGGAGCCTGGAAGTTTGCTTGATCGAGTATTGAACAATAATATCACCATCGCTTTAAACCAAGAAGGCACATCGGAACTTGTGGACCCAGAGGAGCTCAAACTGACCCTTCAAGAATATTTCTCTGGCAATTACTCGGAACGATGGGAAGAGACCTTCGACACGATCGATCGGGTAGCCAAAAATGTCAGCACATACCTTTCGTGCTTCGATTTCAACAAGTTCGTTGGGTTCAATTCCGAGCACGAGTTGGAAAAAGAAGGCATTAATCTGATTGGTGACAATAAACTATGGGCTGGTATTGTGTTCCCTGATTTTCCCATTTCAACCGACTCTGAGAACTTTCCGAAATTCATCAAGTACAAGATCCGCATGGATTCGGACAAAGTGGATACAACTCGATACATCGAAGACCGATTAGCTCGACCCGGACCTCGGAGACGCCCGGGAATCGATCTCAAGTATCTTTATTTCGGGTTTGCTTACCTCCAAGATATGATGGAACATTCGATCATCTCAGTTCAAAGTGGTCGGAACAAATCTGAATTGCCCGGGATCACATTGCAGCAAATGCCGTACCCTTGCTACATTGAAGACCGCTTCGTGATTGCCATTGCTCGAACCTTCCCTCTATTCATGACCTTGGCTTGGGTTTATTCCGCCTCCATGATCATTAAATCTATTGTCTACGAGAAGGAACAACGACTAAAAGAAACGATGAGGGTCATGGGCTTGGGCAATGCGGTTCATTGGGTATCCTGGTTTGTCGACAGCTTTCTCATCATGTGTTTCTCAGCTTGTCTCCTGACCTTGATTCTCAAATATGGAGACATCTTGTCGAAATCAGATCCAACCGTGATTTTGGCATTCATGCTTTCCTACACAATATCCACCATTATGCTCTGTTTTTTCATTACAACCTTATTCTCAAGAGCAAATGTGGCTGCTGCAGCCGGGGGCATCATCTTCTTTTGCTTCTATCTCCCCTATTCCTTCTTGGTGGTCTGGGAGGAATTTCTCAATCCGAACTTCAAAATCGCTTCCTGCTTGATTAGTAATATTGCCTTTGGCTTTGGTTGCGCATACTTCTCACATTATGAAGAGTCTGGAATCGGTGCTCAATGGGATAACATTTGGACTAGCCCTCTTCTCGAGGACAAGTTTTCTATGGGTGGAGCCATGTTAATGCTCCTCTTGGACTCGGTAATCTACGGATTCTTGACCTGGTATACCGAAGCCGTTTTCCCAGGTCAATATGGAGTGCCGAAACCGTGGTACTTCTTCCTAACCAAAAGCTACTGGATCGAAGATGCGTTAAGTTCATTGAATGATACCTCTTCGCTCAATGGAAGTCTATCCGATGCTGAATTGGGAGAGCACAGCAAGAACATCGAAGTTGAACCCAAACATCTGCCTTTGGGCGTGTCGGTCAAGCACTTATCAAAGGTTTATGCCAATGGAAAAGTGGCAGTGGAAGACTTGAGTTTGAACTTTTACGAAGGTCAAATCACATCGTTCTTGGGACACAACGGTGCTGGCAAAACGACAACAATCTCTATCTTGACTGGATTGTTTCCTCCTTCGGCTGGAACGGCATTCATCAACGGCATGGACATTCGAAAAGACATGGACTCGATTAGGAAATCGTTGGGAACTTGTCCGCAGCACAACGTCCTTTTTGATCATTTGACGGTATTTGAGCATTTGTGGTTTTACGCTCGAATCAAGGGGCGTGATCCCGAAGATATCTACAAAGAAGCCGACAACATGATCAAGGATCTTGCTCTTCCCCATAAACGTGATGAGCTTTCGATCAATTTGTCTGGAGGCATGCAACGCAAGCTTAGCATTGCATCGGCATTTGTTGGAGGATCCAAAGTGGTGGTGTTGGATGAACCAACGGCTGGAGTGGATCCATATTCAAGACGAGCCATTTGGGATCTGCTCATCAAATACAAGGAAAATCGAACAATCATCTTGACCACCCACTTCATGGACGAGGCCGACCTTCTCGGTGACCGAATTGCCATCATCAATAATGGAAAACTAATTTGCTTTGGCTCATCGCTCTTCCTTCGCGCCAAGTTTGGCAACGGATACTACTTGACTTTGGTCCGAGACGACAGTACTGATGACGACGAAGAGGAAAGAGTGGAGCCTCTTTTAGAACTTGAGGAGCCTGAAGGCGCAAGTCCAAAGACCAACAATGGAGACCTTCTGATGATTGATAACGATGACGAGCATCCTGCAGATGAACATGATGAGGATGAAGGCGACGACGATGTTACCGTTCAAATTCATGATCTTAATGAGGTCCGAATGCGTCGAAGACCCAATCCATCATCAGGATCGGAGAGTATTGAGAATGACATCATCATCGACGACGAGGGCGTGTCAGATATTCATAGGGTTAATGGGATCATCATCAACCCGTCCAAGGCCAATGAATATGCCTACAACTATCCACACACAAAATTTATCCAGAAATGGGTGCCCAACGCCCGGTTATTGGAGCAAATTGGGTCCGAGATCATCTATATCCTTCCCACAAACGACCCTCGGGTTGTCAAGAAATTTGACAGACTCTTCACTGAACTGGATCGCTACATGCCAAAGTTAAAAATCAAGAGTTATGGTCTCTCAGATACAACTCTGGAGGAGATCTTCCTCAAAGTCGCAAGTAACGGGAACGATGGCGAAATTGTCAGACATTTAGATGAGAATTCAACCGATGTCGGAATGGTGGCCAATAGAGTTCGGTCCGCATCGGAAAGCTCCGGGATTCGCCATTTCTTCAGCAACAAAAAGAAACAACACGCCGCTTTATCCGAAGAAGAAGCCAATCAAACGACAAGTCCGACATTTTCTAAAAATTCCAAAGTGACTCTCAATGGCGTGTATGAAGCAGTCTCACAAGCTGACGAACCACGAGTTGATTCAGCGCCCAAACCAAAAATGCAAAACCCAGTGAAACCTCCAGACAATGATGGACCCACAACACTCTTTTGGAAACACATCAAGGCTTTGGAAATTAAACGATTCCACCACTCGAAACGGAACAAGAAAGGCATGCTTTGCGAGGTAATCCTACCCGCCGCATTTGTGTGCTTGGCCATGCTTTTTACATTGGTATTACCGCCTTTGGAGGAACAAAAACCCTTGGAGATCCACCCGTGGATCTATCCATCATCCGGTGAATACTCTCTACAGACATTCTATTCAAACTCACATCCCAGGGGTGATTGGCCCCGACGATATGAACAGCAATTGCTCTCGAAAACGGGCATGGGCATCAAATGCGTTGTCACAAACGATATGGAAAGTTTTGAAGATAAGACTTGTTTAGAGTCTGGTCCAGATTTGTCAAACTTCACCTATGCTCCGGGTGTGTTGGAAAACATTGAACTAAAGGAATGTTCTTGTGCCATTGGGACCCAACAATGCCCGGCAAATGCAGCAGGGATTCCTCCTCCATCTGTGATTATGCCATCATTTGACGTGCTTTATAACCTTACGGGAAGAAATGTTAGCGACTACCTCATGAAAACTAGAGTTCAATCATATAAACGACGTTATGGCGGGCTTGAATTTGGGGTGAGGAACCCTTTGGTTAACCGGAACATCACCCAACTTGAAGAAATCACCGATAGATGGCTAGCCGCAACCAACAATGGCGAGTCAAGATTAAACCCTACAGTCCGTGATGTCGTTTTTGGTAACCTCGAAGATACGGCCCTGAGTTCAAGTCAATTCGACAATGTCCGGATTTGGTTCAATAACAAGGGTTGGGCAGCATCTGTGGCTTACATGAATTCGGCAAACAATATCATTTTAAGAGCAAGTATTGCAGCCCAGCAGGAGAATTTGGATGATATGAGCTTTGATATGGAGTTCAAGGATGCCTCGGAGTATGGCATGGCCGTCATAAATCACCCGATGAATTTCACCAAGGAGCAACTTGACACGGAGATCATTCGACAGATTGGGATCTCGTTGTTGCATGCTATTTGTGTGATCTTTGCCATGAGTTTCGTGCCCGCCTCGTTCGTGATCTTCCACATTGATGAGCGGGTAACCAAGGTGAAGCATTTGCAATTCGTGTCTGGAGTCCAGCCCATTACCTATTGGATCTCTGCATTTCTCTGGGACATTGCCATGTTCATCTTCTCGGCATCGTTGTGTGTCTTGATCTTTTTGGCTTTCGACGCACAAGCTTACGTTTCCGAAGATAACTTCCCATCTCTTCTGATCCTCATGTTCCTTTACGGGTTCTCCTCGATACCTTTGATGTATCCAGCCAGTTTCCTATTCTCGGTGCCTAGTTCGGCCTTTGTGACCTTATCGTGCACGAACCTTTTCATTGGCATTATCACGACCGTGACCACCTTTGTGTTGGAGAATTTCGAGGACGACGAACTTCAATACATTGGCTCGATTCTCCGCGAGGTGTTCCTCATCTTTCCCCATTATTGTCTGGGTCGAGGTCTCATGGACATGGCCACCGAGATGAATATCAACTTGATTGTCCAGAAATTTGGATACATATCTGTGCGGAACAGGTTCTCGTGGAGCTTCTTAGGCAAATACATGATCTCTATGACCCTTCAAGGGACTCTCTTCTTTGTATTGACCTTGTTCATTGAATACAAGGTCTGGACATGGCGACCGAATTTCTGGCAGAAGCAAACAGAAACCGACGAATTCGACGAATCAGACGAAGACGAAGATGTTATTCGAGAGCGAAATAGGATTTTGAGCAAGAACAATCCCAATGATATGCTGGCAGTCAAATCGTTGCTGAAGCGTTATAGCAAAACTGGAAAACCGGCCGTGAACCGATTGACCTTTGGTGTTCGACGAGGTGAATGTTTCGGACTTCTCGGCGTGAATGGCGCTGGAAAGACCACTACGTTTAAAATGCTCACTGGTGATACTGAAGCGAATGGAGGCGATGCTCTAGTCAACGGAAACAGCATCCTGACCCAATTACAGAAAGTCCGGAAAAGCTTGGGCTACTGCCCTCAATTTGACGCGCTCAACCCTTTGCTGACTGGGCGAGAACATTTGCGATTATACGCTAGATTACGAGGATTAGATGAGGATTCGGTCAAGAAGGTTGCAGATTGGGGCCTTAAGAAACTGGGTCTTGTCGCTTACTCTGATCGATGCGCTGGAACCTATTCGGGAGGAAATAAGCGCAAGCTATCGACAGCCATCGCTCTTGTGGGAAATCCATCCATTGTCTTCTTGGACGAACCCACCACAGGAATGGATCCTGGTGCCCGGAGGTTCTTATGGAACACCATCTTGGAAATGATCCGTGGTGGCCAATCGGTGGTGCTCACGTCTCATTCTATGGAAGAATGCGAAGCTCTCTGCACCCGTTTGGGAATCATGGTGAATGGTCAATTCCAATGTCTGGGATCGGCTCAACACCTCAAGAACCGCTTTGGATCCGGCTACACCTTGACTCTTCGGAGCAATGGCGAGGCCGAGGCTATTCCTACATTGAAGGAGCTAGTGTCTAAGACATTCCCATTGGCTGAACTCAAAGAGGAGCACTATAATCAGTTGCAATACCAACTACCTCTAAAACACACGAAACTGCCCCTGGTCTTCAGAGAGATGGAAAAGGCCAAAGCCAGTTCTAAAGAGTCCTTCTTAGAGGACTACTCGATCACACAAACAACATTAGACGAGGTTTTTATTCGCTTTGCAAGTAAGCAGACCGAAATGGTCGAAGATATTGCTGCCAATGAGGCACTCCATGAAGAATCTCATCCGAGTGAACTGAAGGTG**TAA**

>ABCA3 isoform X1

**ATG**GCGGGGACTAGCAACAACTTTCTCCGGAAGTTCTTCATCCTTCAATGGAAGAATCTTCTTTTGAAATATCGCCATTGGATCCTCACGGTTTGCGAGATCATCATTCCCACGTTGCTCTTCATGGGCATTGTGGCCATTCGAGTGAAGGGAGGGGAAGACATTACGCCCACGCCTCGTCCAGCCGTGGTTAATGATTCTGTGCCCTACTACTTGGCCTATTGTGAAACCTTGTTCAAGCACACCACCAATGGCTCTTTGGCCAATCGTACTCTAACCTTCACCAATCACCCATCTCCGAAAGGACCCCCCAAAATGGAGGAACACAACGACGTGTCTCAATTGGCTCATGACATCATGGGCAGAGTGGAGATGGTGGTGGCCGAGGTTTTGTGGCCTATTTGCCGATCCTCCTTGGGCATCTTAAATCTCACCGACACTGGTCACGGATCATTATTTATCGAGTACCGTGACAGTGAAGATGACATCTTGAAGGAAAGTGAACAATATCAGGCCAATATGGATCTTTATAACGGCGAGTTCTACGGTGGAGTCGTGTTTCCGCCTCATTTCGACTATGGTCCAGATGATCACATTCCCGACCTGCACTACATCATGCGATTTGAGCCCGAGTATTCCAACTTGGAGACCAACCTCGTGTTTCCCATCTTTCAGCTCTCAGGTCCAGGTTATGCAGCGTCCACGTACGACGAGATTGTCAAGTTCCAAAACCTGATCGATCTAGCATATATAGAAATTGTGACGAAGCAACAACAACTTTTACCCAACACAGCCCTGGATCTGCTTCGCCTCCCATTTATGTCAAGTGAGAGGATCATGCCTTATCCAGCTTACTTGGAAAATAATGTGGCCATCTTCCTTGGCTTCATGCTCCCCATGTTCACGATCTTGAGTTTTTGCTTCATCGTCCCGCCCTTAATGAAGCGAATTGTCCACGAAAAACAAACTGGAGTGAAGGAACTGATGAAACTGATGGGATTGCCCAGTTGGATGCATTGGGTCTCCTGGTTCCTGAACGCCCTTCTCACGTCGCTCATTACGATCCTGATCATTGTTCTTTTGGTATGTGTGGAATGGAAGCCAGAAACGGGAAGGGTGTTCGACTACTCGGATCCATTTCTGATCTTTATCTTCTTCCTCATGTATGCCATGCCTCTTATTTTGATGCTCTTCGCCATTTCCACCTTCTTCGACAGTCCTAATTTGGCCTTGGCCACTGGCGTGATTGTTCATGTACTGACCTTCATTGGCCCATCCACCAGCATCAACGACGAAGTCTACATGACCATGGCTTTTGGGTCCAAATTGGCTTTGGCCATCATGCCTAATGTGGGACTGTGGTGGGGCATCAAAATCCTTTCTATCGAGGAAGGAAAAGGCTCGGGTCTCCAATGGAACACGCTCTTTGACAGGCCTGAACCTTCGGATCCAATGACGATGGGTGTGGTATGGGCTATGCTCCTGGCGGATATGCTCATCTATGGCATTATCATTTGGTACGTGGATGCCATATTTCCTGGCAAATATGGCGTGGCCAAGAAATGGTACTTTCCGTTTCAAATGAGCTATTGGCTGCCTGGTCAGATAAAAGATAGCACTTTGGATCATGCGAGAGCTGCTAAGGATGACCATCCATCCAATGATATGATGGAAGATGAGCCTCAGGGCAATCCAGGGATCCAAGTGAGCAATCTTATGAAGATATTTCGATCCCTCACAGGAGCTCCATTCACTGCCGTTAACGGAGTCAGTTTTAGCGCCTTCAGCGGTCAAATCACGGCACTCTTGGGTCACAATGGGGCAGGAAAGACGACCACTATGTCGGTGCTCACGGGCATGTATTCTATGACAAGTGGACAAGCAACTATTTTTGGTTGTGACATTGTCAGTCAAATGGACAAAATTCGGCAACATTTGGGTCTCTGTCCTCAGCATAACATGCTTTTCACCGACTTGAACGTCAGGGAGCACTTGCTCTTTTTCGGAATGTTGAAAGGTCTGTCTCGAAAGGAGGCAAATTTGGCAGCCATGAAATATATCAAGCTGCTTAATTTGGATACCAAGACCAATGAATTATCTAGCAATCTCTCGGGTGGAATGAAGAGGAAGGTGAATTTGGGCATTGCTCTTATCGGAGATTCTAAAGTTGTCATGTTAGATGAACCAACTTCGGGAATGGATCCAGAAGCCCGACGAGGAATTTGGGACCTCTTGCAAGCGGAAAAGAAAAACCGCACAATTCTGCTCACCACTCATTTCATGGAAGAAGCCGATGTCTTAGGGGATCGAATTTTGATCATGAGTGCAGGACAAGTCAAGTGTTCCGGTTCACCCATGTTCTTAAAGCGGCGATTCGGCAATGGGTATACATTAACCATGTCCCGAGGACCGAAATGCAACGTCACAGCCACCACGGAATTCATCCGCAATTACATTGAGCACGCTGAGCTCAAAGGACAAGCTAGTGGCGAACTCATGTTCCAGCTCCCAAACGACCAATCTCATAAATTTCCAGAGCTCTTTGGAGCTTTAAATCACGACAAGGATCAGTTGGATGTGCTCAATTTCGGCCTGTCCGTGACCACCATGGAAGATGTCTTTCTGAAGGTGGGTCAAATGACTGAGTACACGGATGTGGCCTTCGAAAGCATCGATCTTGATTTGAACGACAAGGACGACCTGGGATCACCATCATCATCGTCATCACAAATATCATCATCCACGCGGAGTTTGGCGAGAAAAGCAAGACTTACCGGTCGTGCATTGCAACGGCGAGAATTGGGTGGACTGCTCAAGAAACGAGTCATTTACACTTGGCGAAGAAAAATCCTGTACATAATCATGATGCTGATTCCGTCGTTATACGGTCTGATTTGTCAAATTGTGGTTAACGACACCAAATCTCCCTTGGCCAAGTTCAAGCCACGCGAATTCGACTTGTCCAATTATCCTGAGCCTATTGTTTTCGTCTCAGGAGAAAAGGGCAACCAAGAAGCTCAGGCCTTGGCAGAAACATTCAAGACCTTGATTTTTTCCGGAGCAGAGATTATTGAAACCGACAACTTGACTCAGACGATATTACAAGGAGGGGAAGCTAACATTGCCGAATATCGCGGTAGATACATTCTCGCCGCAGATTTCAAGACAAAGCCAGAACTATGTTTCGATGATGATTGCCCCTCGATCAAATCCAAGATCTTGGCCTTGGACTCTGTGTATAATTCGGTACCCAACCATGCCAGACCTTTGGCCAAGAACCTGTTGAGCAACACGTTATTGTCTTACTTAGAGAGGGACTCCAGTCAGACGCACTCGATTACAACGGGCTCCCATCCTTTGCCCCTCAATCTAAAGTGGCGCTTTGAAATCCTGTCCCAGGAGTTCATCTCCCCATACGTGTTTGCATACGGGGTCGCTTTGCCCCAAGGCATGGGTGTGCTCATTGCGGCTTTTGTCATATTCCCCCTTTTGGAACGGGTCTCCGGGGCAAAACAAGTTCAACTCATGACAGGATTGCACCCGGGCATATTTTGGCTTTCCAACCTCTTGTGGGACTTTCTGCTGTACATCGTGGCTATGATAATCATGATGGCGATCATCTTTGGTCTGGACCAAGAGCAAACATTTCTCGCTGGCGAAGCAGCAGGCGTGACTTTGCTCTTAATCGGACTCTTTGGGTTGGGATCAGTTCCGATGGCTTACGTATTCAGTCACACGTGTGAGTCTGCCGCCTCAGGATTTGCTCTGCTCATCATTCTGAACGTTTTATCCGGGGCGATCGGTCCCACCGGAGTTTGGATCCTCCGATTCTTTGGCGACTACAATGACACCATTGGTCTAGTCATTGCTTCTGATGTTATTCGTTATATTTTCACCCTCTTTCCGGCCTTTCCCATGTCAAGAGCAATAATGGCATTGGTTCAAATCCAAGAGAAGAACAATTTGTGCGAGGTTGGGATCAAAAGAGACACGTTAACAGCTTGGTGTACCATGTTCACGAATCGACCAGAGACTCTAATTCCAGAAGCCAATAGGAACTACGCCCAATGTTGTGAAGCTCCTTACGTCGATCCCAGCATTGCCATTTGTGGTCAGACGTTTCCTTTCGGTCCAATTGAAATTACGGCCCCTCCCTGTCATACCAAGGAGACAGTGTACACGTGGAGCTCATTGACCGGAATCAACATTGATCTCCTCATTCTGACGGCTAACTTGGTCTTTTACTTTGGATTACTTGTGGCTTTGGAGTCGGGCTTTCTAACGAAGCTCAACCAAGCTTGGCAACAATTCCTGGCCAGAAGGCACCAAAAACCAAGTGACGAAAATGACCCACTAGACGAGGACGTGGCAATGGAACAACATCGCATGCACAGTTTGCTCGACCAGTCGTCCGTCCAAAAGGATGCCTTGATGGTTCACCGATTGAAGAAGAACTACGGCGGGTTCACGGCCGTGAATGGCCTAAGTTTTGGCGTTCATCACGGCGAATGCTTTGGCCTGCTCGGCATTAACGGGGCCGGCAAAACCACCACCTTCAAGTTCGTACTTCTCCATGAGACCATGTTTTTCTTTTCTAGCCCTTTGGTGTTGGATGAGACATTA**TGA**

>ABCA3 isoform X2

**ATG**AAGCTGGCCGTGGTCTTGGGCAAAAATTTCGTGTTGAAGGGCAGACATTGGATCCAAACCGCGTTAGAGATCGTGGTACCCTGTCTCCTATTCATAGCATTAGTAATCATCCGATCTGCCTTAAAAGTGGATCCTAATAATGAAGATCCCGGAGCAAGAGCGTCACAGTATACAAGTTTTTTGGACGATCGCGCCGGGATGCAGACATTTTGTTGGTCGCTTTATGGTACCACTGGCGGAATTGTGTATGTTGCCCCAAATAGTTCCGAATATGCTCGGAAGTTCGTGGAACAGTTCAACGCTGCTGGCTTCAAATACTGCCAAGAGTCCAGATGGACCTATGAAGAGGATTATGTTGTCGCGGAGTTGATTGAATCAGAAGACGACATAGTGAAAATGTACCAAGAATACGTGTCTGACTACAATGTCAGAAAGAAAATTGGCCAAGATGATGATCCAAAACCCCCCCAAGTTTATGGTGGGATCGTTTTTGAGCCTAACCAGTCCTTTGAAAGTTCCCATTTGAAGTATAAAATCAGGATCGGATACAGAAATGATTACAACGATGAGGCCCAAACACAATACCTCTTTGATCCCTGGATGAAATATAATTACTACGGTCCATCCGACGCCATTTATAGTTATGACACGGAGGTTGCCATTCAAAGCATTGTCGACTTACTCTATTTACAAGGCCGACTTCATGAGGAAATCTTCATCCCTGTTCATACCAACTATTCAGATGGTAACGTCAAACTTTCCATCTCTGATCAAGGGTTTGCGGTTCCAAGCCGATCTACCAATTTTCTGGCCTCAGTTTTGGATTACATTATGCCGATCTTCTCAGTGATTTCGTTCATGTTTGTCGTTCCTCCATTGCTGAAGAGGATTGTTCAGGAAAAAGAAAGTGGAGTCAAGGAATTCATGAAGATGATGGGATTGGCAGGTTACATGAACTGGCTGGCGTGGTTCATAACGGCCTTTGCTTCTTGTCTTATAACAAACCTAATCATTTTGTTTCTCCTGGGCGTAAATTTCTCGTTGGGACCTGTCATCCAATATACCAATTTGTTTATCATCTTTATCTCGCTAACACTTTATAGTGTAGCTCTGATTTTCATGCTCTTCGCGATCTCCTCTTTGTTCAACAATGCTAACCTGGCTTTGGTGGCGGGGATTTTGCTCCATTTGGTTTCGTTCTTCTTCCCCTACGGATTGATGAGCCAAAGTGACCAGTTCTACGTGTCCGTTAGTTTAGGGGCAAAGTTGGCATTGTGCTTGTTGCCCAATGTCGGCTTGTGGCTGACCTTCAAGGGAGTTTTGGGAGCCGAGTCCGCGAGTGCCGGCTTTACCTTCAGTGATATCAATGAAGCTCTCATTCCTGGAGATAACATCACAATGCTTTATATCTGGCTGATGTTTCTGGCTTCGTCTGTGCTCTATGGTGCCATCATCTGGTATGTGGACACCATCAAACCAGGTCCTTTTGGTCAAGCCAAACCACCCTACTTCCCTTTCATGAAATCATATTGGATTATGGAGAAAGCAAAAGAAGTGGATTCCAAATCAATTGATATGGACGAATACGGTGCTGTTTGGGAACCAGAACCAGAGGGACAACCAGGGGTTCAAGTCAGAGGGTTGAAGAAGGTCTTCAAGAGACCGGGAAGCGCCCCCTTTGTGGCAGTCAAGAACGTGCGATTTTCGGCCTATCCTGACCAAATTATGGCTCTGTTAGGTCACAATGGAGCTGGCAAGACAACTACCATGTCCATTTTAACAGGTTTGTATTCAAGTTCCGGGGGGACAGCTTTGGTGAATGGCTTGAACATCAACACAAGCATGACAAAAATCCGGGGAGACCTAGGTCTTTGTCCACAACACAATATGCTCTTTGCAAGTTTGACGGTGAAGGAACATTTGATCTTTTTCGGGATGTTGAAAGGGATGTCGTGGGAACAGGCTAGACGAGAGTCGACAATTGTCGCTGATCGCGTAATGCTCTCTGAAAAGATAACCAAAATGTCAACAGCTCTGTCTGGAGGCATGAAGCGTAAACTCCACTTGGGCATAGCCTTGATGGGAGACTCCAAAGTGGTTCTTCTGGATGAACCCACTTCAGGGATGGACCCAGAGGCACGACGAGAAATTTGGGACCTTCTCCAAGACATGAAACAAGGCCGGACAGTCATTCTTACAACCCATTTCATGGAGGAAGCGGACGTCCTGGGTGACCGCATTGCCATTATGGCCGATGGCCAGGTTCAATGCTACGGCTCATCTCTCTTCCTCAAAAGAGCATATGGAAGTGGATACCGCTTGACCATGACCAAGAAGGCCGCCTGCGATGCGTCCAAGGTCAAGCGTGCTGTGCGAGAGCACATACCTGGCGCAGTGACCCTTAGCAATGTCTCAGGGGAGCTCGCCATGAGCTTACCCAGCGATAGCGAGGATAAATTCACAGATTTGTTGAAAGGTTTGACTGATGCCAAAGCCACATTGGGCATTGCCAACTTTGGTTTAAGCGTGACCACCCTTGAAGACGTATTCTTAAAAGTTGGAAGCAAAGTCGAGGATACTGAAACCCTTAACAAAGAGGAAACGAAGGGGAAATCCGTCCAAAGATCAGCTTCAATTGAGAGTGGACACAAAGTTGGAAAGTTACCGGAGAAACGGGAAGATCTGGTCTCAGGTTTCAAGCTGTGGTTGCTTCAGATGAAAGGACTTATCACGAAACGAATAATTCAGACCAAGCGAAACTGGATGATGTACCTGTTAATGGGATTGGTACCCATGATTATGGCAATTCTCTCCGGAATCGTCGTCAACCTCGTGTCGGATTTGGGTGGCATCTCAGACTCTAAGCCTCGTGTAATGACCTTCAGAGACTATCAAGAGGACAGCACTGTTTCCCTGTTAAATCGAAATTTCACTACCCAATATGCCAATGAATCCTATGAAGTGTTCAAAAACTTTTTGTCAATCTTCCCCAAAACTAAGGTTATTGAGGTGAAGGATTTTGACAACTACATCTTGGAAAACGCCGTGGACGAGGGAATGGAAAGATATGGCCGGAAGTACGTCCTCGGGTTTTCAACTCACGAGTTCTCGAATGCATTTTCACCAGAGACGTCCTTCACTACAACAGAATTGAACGCCCATTTCAATGTGGTGCCGTTACATGCTCGACCAATGGCCAAGAACTTGTTAGCCAATATGTTAAACAAAATGGTAGTCAATCCTGATGCCTCTCTAGACGACATCCAAGTGACATATGAGCCTATTGTTGTTAAGAATGATTGGGAGCGCTTTTGGTATGGCGATATAATGTTCTACCCCAGTGTGTTGGTCTACTCCATTCTACTCTCCGTATCTTGGATTCTATTCTTTGGCACCTTCATCATCTTCCCCATTCGGGAACGACTCTCGCAAGCCAAGCAGGTTCAAATCATGGCGGGTGTGAATCCATTCACGTTTTGGCTGGGTAATTTGGTGTGGGATTTCCTGATCACTTTGATCGTCACAGTTTTGGTTTTGGTACTACTTGGTGTATTGGACGCGAGGAACCTCTTTATTGAGCCCCAAGTTTGGGGTGGAATGGCTGTTGTTCACATTCTCTACGGGTTGAGCGGCTTACTCACGGCTTATGCGTTCAGCTTTGCCACAAAGTCGGCACCCGCAGCTTTTGCCTTCTATGTTCTCGTGGCCTTGGTTTGTGGCGTGGTTATCCCGAATGGTGTTTGGTTTATGAGTCTCGTAGACTACGAAGTCATAGACCAGGCGGACAATTTAGGAGCCAATATGACTGGGCCGGATTTGGGTCTGATTAGTGACTGTTTACGTTACTCCATTGGCTTTTTCTCAGCCATGCCCATGACAAGAGCTATCATGGCCATCACCCAGGTCTCCGAAGAAAATGGCCGATGCTTGAACAACATCCCTTCGGATATATTGAACAATATTTGCCAAACTTTCCTGGACAATCCGGCGTTGTTGGACCCAAATAATGGATACCAACTCGACTTGAACGTGGCTTCTTGTTGCGATGAAAAGTTCGTAGCCGATCCGGCCGTAGCCATTTGCAACACCACTCTCACTTACAACAATGCTAATGGCGAGACCGTGACTTACCTAACGCCTCCTTGTCCAATCCGCCAAACTTTGTTCTCCTTCGACAGAGTCAAAGGGATCAACATTGATTTGCTCTATTTGGCCCTGGACTCGGTTGTTTTGTTGCTCCTGCTCTATGGCCTCGAATCTGGTTATCTTCAAAGGGGATATGCCCAAAAGTGCAATTGGATTTGCCGCTCCAAGCCAGTTTTTGAGGCTGATCCCATTTTGGACGAGGATGTGATTGCTGAGCAAGAGGAGGCTATTCAAGCACTTAGAGACCCCGAATCGGTCGCCCTGATTGTCAACAACCTGACCAAGAACTTTGGCAAGTTTAGGGCTGTTAGAGGACTCAGCTTCACTATCAAACATGGTGAATGCTTTGGTTTCTTGGGTGTAAATGGAGCTGGGAAAACGACCTCGTTTCGCATGCTGACCGGTGATGAGTACATGACAGAGGGTCAAAGCATTCTTTATGGACAAGACTTGGGCTCAAAGCGTCGGAAATACTTGCGACAAATCGGGTATTGTCCCCAATTCGATTCCATCATAGATGTACTCACGGGCAGGGAAATTTTGAATTTGTTCGCTCACATTCGTGGAGTGCCTTTGAGAAGAATGAAGGAGGAAGTAGATAAGTGGATCGAGTTCGTCGGCTTGAAACAGTATGCAAATCGGAAATGCGGCCAATATAGTGGTGGCAACAAGAGAAAACTCAATGTGGCTCAAGCCTTAGTTGGTGATCCACCCATCATTTTCTTGGATGAACCGAGCACAGGGGTTGATCCTGTGGCACGGCGGAAACTTTGGGACGCTATCACTTCCATCAAGAGTCGAGGCCAATCTGTGGTTTTGACCTCCCACAGCATGGAAGAATGTGAGGCTTTGTGCGATCGGATTTCCATCATGGTCCAAGGACAATTCCGGTGCATGGGTGGCCCACAACATTTGAAGAACAAGTATGGTCAAGGATTCACTGTGATCATGAAGATGAACCAGTCTCATCCCACATATGAAAGTGACACTGCCAAAGAGAACATCAAGGGCTTCATGATGGAAAAATTTCCTTCAATTCTGATCAAAGATGAACATAAGGATTACATTCATTTCCACATTCCGGATGTTCAGACTCCGTGGTACCTACTGTTCCAATCGATGCAAGAAGCCAAACTGAAGTTTCCATTTGTTCAGGATTACTCACTCAATGAGACCTCTTTGGAGGACGTTTTCCTTCTCTTTGCAAGAGGTAGAGACCAAGATGATTCTCGAACCCAAGTTAACGAATTGGGAACGGTTAATGAGGCCTTTGAA**TGA**

>ABCA2

**ATG**GACCGTGGGACCCGGCGAGATGCCAGCTTCTATCAACAGTTCCGGGCCACTTTGATCCGGAACTTGTTGAGGAAGAAGCGAGCCCTTCGTCACACACTTCGGGAAATTTTGGCTCCCATCTACTTTCTGGCCATCATTATCATCTTGAAACTGGCCATTCCCGAGCCCTACTACGCTCCAGTGGATCAACCTGATCAACCCGTCGAAGTGTTTAGTTTTTACCCATTCGTCAATTTCACCCAGCGACCAGATCTGATAATAATCCCCTCGGAGGTGAAAGACCACCCATTTTTGTTGCAACTACAAGACAATTTATATTTACAATTCGCGGATTTTAACTTCCTGGATTCGGAAGAAGACTTGGAAAACCTATTTGACCAAGATCCCAATCAAATCTATGCGGCCATCATCTTTGATGACACCTCTTTGGAAACGCTGAATTATAAAATTCGATTCTCATCACAAGATCCAATGAATTCCCTTCCGGCTTCGTACTCCAAATTTGCTTCCAACAAGGAATGTCGAGTGAAAGGCAAGACTTCCGACCATCATGAGGGAAGTAGTTTTCCAGCATTTTTTCCAATCCAGCGAAGAGTGCTCGGAACCTTGGGTAAAAAAATGAGCCGTCAAATTAGATCGGTGGAAAAGGGCCAGGTTCCAAGTACAGAAAGATCGGAATATCCCGATTTCTTTGGTTTTCCACCTCCTTGTAGTGTTGGTACATATTACTGGTCTGGGTTTTCGACCATCCAGGCTGTCATTGATAACGAATGGATGAAGTTCCAATTGGGCGACCAAGGTGACTTCAAGTTTCCGAAAATTGCTCTTCAAATGCTCCCAAAACATGAATTCACGGGCACGGCTGCCGTGGTTTTGAGGTCTATTATTCCGTTATATTTGGTCATCTCTCTGTCCCAATTCATCACACCCATGTTGATTGTGGTGGTGGATGAGAAGGAAAAGAAAATCAAGGAAAGCATGAAGATGGTCGGACTTCGGGATTCAGTCTTTTGGCTCTCATGGTTCGCCGTGTATTCTGTAATGGTCCTCATTATCGCCCTCATTGGATCCATTCTGGTCTACTTCGTTGTGATCAAAAGCACCCAGTTTTGGGTGCTTTTCGTGCTCATGTTCGAGTTCGGGTTGTCCATGATCATGTTCGCATTCATGCTTACATCCCTATTTAGCAAGGCAAAACCTGCGGGAATTGTGGGCGGATTGTCCACAATGCTGCTGTCGTGTTTGTATTACCTTCAGGTTTTCCTTTCGGGTTCTCCCGAGCCCATTTTCTGGTTATTGGGCTTGCTGAGTCCCACTGCTTTCGCCATGGGGGTAGACAAGTTGATGTACTTTGATTTGGAGTCAAAACAGTTGGATCTTTGGGATCCTGCTGACAGTATTCCCGTGGCGGGGGTCATGATTATGTTGGCTGTGGACATTGTTTTGTATTTGCTTATCGCATTCTACTTGGACAACGTTGTTCCCACCGAGTATGGAACCCGTCGGAAGCCTTGGTATTTCCTTCAACCTTCATTCTGGAGGAAGTCTGTCAACGCCCTCTGTCCAAGGAAATCTCAAGTGAATGATCCCGATCGGATGGCGAAACTGCGCAGTTTGGTCAATGAATCCAATTTTAATGGACCAGATTTTGAAGCCATTCCCTCCTCCATGAAAGGTCGTGAGGCCATCGTGATCAATGAGCTCAAAAAGACCTTCAAATCCATGGGAAGTGCCCCCGTTCATGCGGTCAAAGACATCAGTCTCAAGATTTATCCGGGAGAGATTACAGCCATTTTAGGTCACAATGGTGCCGGTAAAAGTACCTTGTTCAACATGCTCACGGGGATGACCTCGATCACTTCGGGTTCGGCCGATATTTTTGGATTTGATGTGACTGACAGCAATCAGATGGAGGAAATCCGGAAGATGACGGGTATTTGCCCCCAGCATGATGTTCTCTTTGACGAGTTGACCCCGAGGGAACATTTGACGTTTTTCGCCAGAATCAGAGGAATGGAAGAAAGCGAAATTCCTCAAGAAGTGGATAGCATCCTTAAAGATACAAATTTGGAAGAAAAAGCGGATTCCGTGGCCTTGAATTTATCCGGTGGTCAAAAGCGGAAATTGTCTGTTGGGATTGCCTTAATTGGAGATCCCAAGATCATTTTCCTGGATGAACCGACGGCTGGGGTTGATCCGTATTCTCGAAGACATCTATGGTCAGTTCTGAAAGAGAGAAAAGAAGGCAAGGTGATTCTCTTGACAACCCACTTCATGGATGAAGCGGATATTTTGGCAGATAGAAAAGCCATTGTGTCAGATGGAAAAGTGCGGTGTTACGGGTCCTCTTTGTTCCTCAAGAATAAATTTGGAGTGGGCTATCACTTGACTTTGGTGCTGGAACCTAGTGGGAACATTTTGGGAATTCGAGAGCTCATTAAGCGTTTCATTCCGGATGCCGAACAAAATCGTCTTTTTGGGCGCGAACTCTCGTTTATTTTACCCAGGGATGATGTGGACAAATTTCCTGACCTGTTTTCGGCCATTGAATCAGATATTGAAAAAAATCACTTGGGAATCAGTAGCTACGGTGTTTCCATGACCACTTTGGAAGAGATCTTCTTGAAGCTGGGAGAAGAGGAAAAGGCCAAAAAGGAAGTTGAAGAACTGAAGAAATTAGGCGAAGAAACGGAGGGCATGATTTCCAATGGAAATGTTGGCAACTTGAATGGAACGGTCAAACCTACTCAGCACATGGCCAGTAATCAAAGCAATAAGGAAATGGGCGGATTCTCATTCGAAGCTGTGCCAACCGAGAAATCCAAGTGGCAAATGTTTTGGGCTTTAGCTTGGGTTCGATTTGTTCGGAAATACCGAGAACCTGTGGCTGTACTTGTTCAGATTGTCTTGCCCGTGGTTTATCTGGTCTTGGGTATTTGGCTCACCACTCTTACTTCCGTTGTTCCACCAGAAGAGGTCCCAATTACAATTTCATCAGACATGTATGAAAAAGATTACCCCGAGACTCCAAATTCTGCCTTGTTTTCTATATGGAGTCCGCAAGGAACTGACTTAACTGACATGGTTAGCAGGTTAAATGGATCCACCTTGAAGTTACCAGATTCTACTCAATTCAGTGATCTTTTGACGACAATGCAAATGGGTGCTTTTAAGGTGAACCAATTCAATGAGAATGCCAAGTCCATAGACCTTACAGCTTTTGTCAACTTTACCAGTCTTCATTCCGTACCAATCATGATCAACCAAATCAGTAACATCTTAGCCGATCTCCATAACCTCGACCCCATCACAACTATTTCTCATAACTTCAAACACACTTCAATGGCTGGCGACTTTGACTTTGGGTCTTTTGGCGGGACTATCTTCATTGGTTTCACATACGTTCTTATTCCAATCGGATTGGCCTTGGAGCTGATTGAAGACAGGGAGATACGAGCCAAAAACCAGTTGAGAGTGAATGGATTAACCTTTGGCATTTATTATGGCTCCTTTTATCTTGTTTTGGGAGGAATGATGTTGGCAGTCTTAGCGGTTTTGTTGGGGCTGGTCTGGGCTTTTCAAATTGCTCCTCTCACCTTGATTCCGGCTTTCGTAACCTTGGCTCTACTGTATCTTATCTACACCCCGGCTGCTCTTCTTTTCTGCTCTGCCATCAGCTACATGTTTGACAAGGTGGAGAATGGACAGTTCCTTTTCCCCATTGCCTCCTACGTTGGATTCATTCCTTACATTGCTGTCTCGTTATTGGACATGTTTCAGGTGGCTGATGGATTGGTGGGCAAGATTTGTCACGTGGTTTTCGCCTTCTTGAGCCCTATCTATATTCCATTTGGAGCTGTGTATTACATCAATCGGCAGTTTATCTTCAACCTTTGTGGCATCCTGACTGATTGTTCGGAGATCACCTTTGGCAACTACATGGGACAATATGAAATCTACACCCTATTCATTGCCTGCTTCTTCCACATCATTGTCTGGTTTATTGTTCTTAAAGTGGTTGATGTGGTAAAAGATGGAGGAAGCATCAAAGAAGGATTTTCCTTTTTAACGCGAAATAAAGAAAAATACAAGAAGTTGTACGAAGACCGGGAAAATGTGGATATGATCGAAGATGAAGATGTGGATGTTCGTCGTGAGCGTGAAGAAATCAACCAATATTTTGCCCAAAACGGTGAAAATCGCGTTGTAGCCGTTCAAGGTCTCCGTAAAGAATTCACTACCAACGAGGGCAAAGCTGAAGAAACGTCCATTAGCAAAGATTCAAAAAAGAAAGAAAGCAATAAACTAAAAGTTGCCGTTCGAAACATGACCATGGGTGTTCATCAAGGCGAGGTTTTTGGTCTTTTGGGGCACAATGGGGCCGGAAAGACCACCACCATGAGAATCATCACTGCCGAAGAGGCTTCCACGGCTGGAAACGTCAAAATTGGAAAACATAATATCCTCTCGAACGCTTCTCCAGGCTTCGATATGTTGGGATATTGTCCTCAGTTTGATGCTGTTTGGAAAACCATCACGATTCGAGAGCACTTGCAAGTGTACGCCGCCATTCGAGGTGTGGCCAAGCCCAATATTGACCGCCTAGCTAATCAATTCATGGAAGGACTCCAAATTGAAGAGCACTCCAAGAAGTACGCCAAGAATTGCTCAGGAGGAACGAAACGGAAACTGAGCTACGCAATGGCCATGTTGGGCGATCCCAAGATCGTTCTGCTCGATGAACCCAGTACAGGAATGGATCCTCAATCGAAACGATTTGTTTGGGACACCATATTGGCTAGCTTTAAGAATGACCGGGGTGCCATATTAACGACTCATTCAATGGAAGAGGCTGATGCTTTGTGCACAAGAGTTGGAATCATGGTCAAAGGAGCACTCAGGTGCCTAGGGTCTACTCAACATTTGAAGAATCGCTATGGAAGTGGCTACATGCTGGAGGCCAAGTTAAAACATGAAGGTGGACCAGAAGAGAACCTGGATCAACAATGGACCACTTTGGAAGAGGAAATCCTCCACGAGTTTCCAGGCGCTGTACTCCAAGAAAGATTCGCCGATCGACGAACTTATTCTATTCCACAATCAGCATTCTCATCTCTGGCCTCGGCTTTCAAGGCCTTGGAACATTTGAAATCCATTCATGACGTCGAAGAGTACAGTTTTGGCCAGACTACATTGGAACAAGTGTTCCTGGAATTTGCCAAGCAACAGGAAGCTGCCGATGAAGATGCACAAGAGGAAGAATTGGACCATTTAAATGATATGGGGATCTACCAAAGGCGTGTCAGTAGAACGGGAAGTGTCCTTCCTGTAACCGAGCTG**TGA**

>ABCA13

**ATG**ACAGCCTCCTATTGGGCTCAAATCAAGCTCAATCTGTGGAAGAGCTGGCTTGTGAGGAAAAGGAAGCCAGTTCTATGGACTTTTGAGGTTTTGAGTCCGCTTATCATCGTATCCATAGTTGCTCTCATGCACTTGGGATGGGGGCCGTCACAACTCGATACATGCTACTTTCGCTCTCGTGCTAATCCCTCAGCGGGAAATTTGCCATATTTGCAATCTAGTCTGTGTAATATCATTAATCGATGTCGACCTGAGGATACCTTCGAAGATGTTCCTACTTACCTTGGATCCAAGCTACCCGACTTAGACCAATATGGATCACCCATATTCTATGATGACAATATTCTAGCAGGAATAGAAAGTCTAGAAGTAATGGTCAAGATCTTGGATGGGGCTGCTGAAACGTTCAACACCACTTTTTGGCAAACTGTTCTTGATGACCAGTCTAAAGTGAGAGAATGGTTCAATGAACCTGAGAGAATCGCAGACTTCCTAGAGACTCAATCCCAAACTATGGATCAAGACTTGGCTGATTTTTTCCTCAATTCAACTCTCAGACTCTCCACCGTGATCAATCAAATTGATTTGCTAGGAGCTCAAACCACTGCGTGCAATTCCGAGTTGTTGTCAAAATATATCATTACTCACGATGCATTGGCCATTCAAGAGTTTTCAGATGGATTGTGTAATTTGTCTGACGATCAAGTGGTGGACTTCATTGAAGTGCTTCATCATAACCTGGATTTGGACAACATCTTCCGGACCATCGGGCATCATGTTGAGCTCTTGATCAACTATGATGTGTATGCTGGGCTTCGTGATCTGGCGACAGTAGAAGGTTCCATTGTCAATATCACCGACATTGTGGGACACGTGGAGCCTATCTTTCATTTCGATTTTTGGATGGAGGAATTTACCAAGCTGATTAGATCAATTCTGGAAAACGAAAACCCGTATTCTTGGGAGATAATGGATCGATGGGCTGAGTTGACTCTCGGCATTTACCTGGAACCATCTTTCTACGAAGACATCCTCCTTTATGTAAATGAGCTGACCAACGCTCTTGAGGAAGTAAACCAAGATTTTTATTCAGACGGGCAGGGGAATTTTGCGTTTTTCTCTGAATACAAAATGTCTCCAGATGTTATGAAAGAGGAAGCTCGTGGACCATGGGAATCGTTGGCTGTGGCTTTGGAACATTTGATCAGAAACATGTTGGAGAACGAAATGAAATGTGCTAATGATATTGGATTGTCGTGTCAGCAACAGCAAACGTCTGTCAATATCGCTCAAAGCATCCTCAAAAAGGGATTTTTAATCTTGTCGAAAGGGCCCTCGGATGTAAATGCTTACGATTACCTGGACTATTTCCTGACTGGTTTGGTCAATGAGTTTGAAAGTGGATACTCCTTCATTTTAAGTTGCATTTTGAACTTTCAACCCATTATCGACACCTCAATAATGACGTATTCCAACGTGTTGGTGTTCGGAAACTATTTGCGTCATGGATGGCCTTCTTTAGAGCAATGTTCGAACGAATTTGAAGATTATTTTCATCCGCCTTCTTTTGTTGACATCGAGTCGCTCTCGAAAACCATTTGCTCTTTGAACGCCCTTATTAAAGGCGAGATCCAATGGGATTCAGGCATTGAAGACCCTTTAATAAGCACCATCGTCGACATGATCAATGGTGATGTGCCACGTTCTAACACGACTTCAGACGCTCTGGTGGAGAAAATTGAGCTCATTATTTCGGATGGGATTGGTTACCCCTGGCCCACTTGGAACTCGTTCGAGTTTGATGAGACAATTGAAGTGGCCGAGACGAACTTGCAAGATTTTACCTACTATGACATATTTGAATTGATTCAAGATCTTTTGAATTTTGCGGAGCGACTGATTGGAAATGACGAGTTTTTCAATTACACCAATGCAACCATCGCTACCCAAAATGAGGTCTTGAAATTAGCCATGGAATTTACCATTGATGCTCTGGAAAACATGGATCAAGAGTATATCTACATCTCTATATCCGACTTTCTTGTGGGATTTGACAGTTTGGACGAATTTGTGGAAAGCGTAGCTGAGACCATTCCTTACGTGGCCAATTCATGGTCGGATTTGCTCCTCAATGAACAATTTGAAAACATTACGAATGGTATTTATGCCCTCGTTTTGGACCCAGAATACAATATTTGTCAAGGCAACGCCAGTATTCGTGATGTTTGGGACTTTCCAATCGAGGCTGATCCTTTCATCGACAAGTTCGAGGCAGGCGTTTGCGATTTTGATGGGGTGCTCGATGAACTAAATAATTCGTCGTTCTTTCAAAAGCTGGACAATATTTGGTCCACGCCAAACTTTCAATTTGAATGGGCAGAAGCAGGAGAACGAAGTGAAATTTTTGTATCACAAGTGATTGAATTGGCGAATGCATCCGAGACACGAACGGTTGTGATAGATTACGTGGATGAGGACTTCAATCGGGCAGTCTTGAATTTTCTGGATAACGCTTGGGTGTATTGGAAAGGACTGTTCCATTCAGAGTTCGATATCCTGAGCGGCAATGTCAATTCTAATCAAGATGTGGTGCATTGTTTGACCATCGATTTGCCTTGCCTGAATAGCCAAATTGGAAGGTTTGCCAAGTTTGTATCGAGATTCATCAGCGATTTTGAGGCTCAAACGGGATTTTTGAGCACTTTGGAACAAGACGGAGGTTACCTTTACACTCGTATTGTGCCCCAAATTGTCATTGTTGGCTCCTTGAGGGATTTCCTCCAAACTGATCTCACTGAAGACGACTTGATTGTTCGTTTGATGGGCTCAGGTTTGTCTGAGGATGCTGCCTTGACAATGGCCAATAGCTATCTCAACTTGAATCGGGTGTATTTTGCCAGCCAAAACCCCGGCACATTGACCCAAAATACCATTGAAGATAACCAAAAGTTTGCCCGAGTCTTCAAATTTGGCAATGCCACAATTTATGAGAGAATTCATGCGGAAATTGAGCGTGCCAATGTCAGTCAAATCGCCTTAACACTTGAAGAATTCATCTCTGTGGATAAAGTAGTCACTTCGATTGAGACGATGCGAAGAGATCAATTTGAAGATTTCACTTGGATCAAGTCTTTCATCGGTCTTTCGAATGGAATCGTCAATGACGTCAAGGATTTGATCTTTCATGTCAGATACCTGATTAGAACACTTGCCAACGCCGAAATGGAGCAAGATTTAATTCGATCATTGCTTCAAATTCTCAAACAAGAATCTATTGGAGAGGTACTTGACAGCTTGTTTGGCTTAATCAACGGAGTGGAGTTGCTTATTGGCAATACCAAAGTCAGCAATCATATGGATCAAATACGAGAAGCTCTGGAGGGTCTAGAATTCATCCAGGCCTTGAGAGGTCTTCAATTCCAATTGGAAGTCCAAAGCTTGTTTGATCCATGGGCCATGGTTGAAGGCCACTTGGAGCAAGAATTAAACATAACAGCCCAGAGTGTCCAGTCCCTTGCCCAATCCTATCTGGACCTTCCAAAACTTGTCCTGGGGAACAGTGGGCATTCCAACAACACTATCATTGACTACGTGTGCGATTCAACTCTGTTGGACGATTATCTTGATCCTCCACTTGACTTTAGTGAAACGGATTGGGATATTACTGACGTGAGCACAGGCCTTTGTAACATGACAGCTGGCCAATCCCTGAATCTAACTATAATCATGATCGAGGAGATTGACATTGAAGATTTCTTGCGAGAGTTCCTATCGCTGTCCTTGAGCAACATCTTAGGGCCTCAAAACATCACAGAATCAATGGGCGAAAAGGCCTTGAATGCATTCTTCGAAGCCACGACCATCCTTTCCCGGGATTACATGGATGACATTAATCATTTGGTGGACTCTTTTGAAGATCTCTTCAATGACACAAGTATGTCGGCTCTAAACTTTGCCGGAGTCGTTGCTTGTGGCGACCCCTACAATTTCAATATCAATCTCGACATTCCTGGGTCGAATGGAGATGAAGATGAAGATGAAGGAAATGAATGGGTTCCTCCTCCAATTGCAGATCAATGCACCAACTTTCGAGAAGCGCTTTCCAAGATCAAAGGAGGATCGATCATTTGGACCACCATTGCCAGTTTCTTTCAGGGACAAATCATTTATACTCCTTCAAACTCGTTCACCGACAGTCTTGTCAAAGAGATGAACATTTCTTACTACAAAGAAATTGAGAAATTTCAAAAAAATGTCAGAGTCTTCCTAACAATTGCCGACAGAGCAGAAGAATTGTCCATGATCTCAGATGATTTTGCCGTCTATGACTTCCTCATCCACTCTCCCGTTTTCAAGGCTCTGATCAATAACTTATTTCCAACATTTGATTTAGAGCTGCTGGAAGATCTAAACTTGTTAGAAATCATTTCGAGCCTTGTGGATTATGAGAGCGTTTGGAAGACCGTCCAAACCGTGAGGTACGCTGTGGAATGTTATCATTTGGACCGCTTTGCTCCAGCCTCGTCTGAAGAAGAGTTGGAACGCATGGCACTGACTCAGAGCAATAACCAATCGTTTCAAGCGGGAATTGTATTTTTGGGACAAGCTGAGGACGAGGATGCCCTCTATACCGAGGCTGATGAGACGCCCAAGCATGTCAAATACAAAATCAGAATGAGCCGTAGCTCGGTTCCAACCACAAATCAAATCAAAGACACATTATGGGTCCCTGGGCCTGACGACAACTATTTCCTGGATTTGAAGTACTTGAGAGGGTTCATTCAACTTCAAGATCTTGTGGATCGAGCCATTCTTAGCGTCACATCCCAATTTACCAACTCAACCGATCTTTCAAACATGGGGGTGTACACTCAACAGTTTCCATATCCGTGCTTTGAACGAGACAATTATCTCAGTGGGGTATACACCGTTCAAGTTATGCAGATATGTTTCTTTTTGGGATATGCTATTACTTTGGCGTCAACTGTTCGTTTTCAAGTTTGGGAAAAGGAATCCCAAAATCTTGAGGTCATGCAAGTGATGGGAATGAGAAGGTCGATTCCTTGGATTGTTTGGCTCATCATTTCGGGAGCGACCATGATCTTGTCTAGTATTTTGCTCACCATCCTCCTGAAAGTCAGTGGAGTCCTTCCAAGAACAAATCCAATTTTGCTCTTTGCTCTTTTGCTTTTTTATGTCTTGTCTCTCCTTGGATATTGTTTGATTTTGGCTGTTATTTTGAATACTGCCTTGGTTGCTACTGTGGTGTCTGTGCTCCTTTACATTGCCTCATTTCTACCATTTGTGATTCTTCTTTTGGTGGAGACTAACGGTTTGTGGCTAAAAATAATCGCAAATCTGTTCATGTCCAGCAGCTTCAGTTATGGAGCGCTTTATCTTACACGATACGAGCAACAAGCCATCGGCCTTCATTGGGACAACATGTGGGCATCTCCATTGGGAGATAAAGATAACTGGAACTTTGGAATTGCTTGCTGTTTCGTGCTTTTGGATGCAATTCTGTACAGTGTTGTGGCCATCTTTATTCTTTGGTGGCAAAGAAAATCTTGGAACAACCAAAAGATTGAAAACGGAACACAAGGGTTCTCGGTTCCGGGAGAAAATCGTACCAATGAGCAAAGAGAAAGGAACAAGGTCAAAGACAAAATCATTGGGATCTCAATGAAAAACATTACGAAAGACTTTCCCATGCTCAAAAATAAAACGAAACGAGCTGTCAATAACTTGACCATGGATTTGTATGAAGGTGAAATCACCGTTCTCTTGGGGGACAATGGAGCAGGAAAAAGCACTACCATGAAACTTTTGACGGGCATGGAGAGCCTGACTTCGGGAAATATCGAAATCTGTGGGTACTCGTATCCCGATGAATGGGCCCAAATCCAGTCAAAAATCGGTTTTTGTCCCCAACAATCGGTTCTGTTTCCCGACTTGAGTACTCGGGAACATCTCCAGTTTTACGGCAAATTGAAAGGAATACATAATCCCGTGGAACTTGAGACTGCCGTTAATGACTTGATTGAGGCCATGAACATTGGAGCTGCCCAACATGAACCTGTAAGGAATTTGTCTGAGGGAAACCAGAGACGCGTGTGCATCAGTTTGGCCTTTATTGGTGGCTCGAAAGTGGTCATTTTGGATGAGCCAACATCTGGAGTGGACCCAATCGCCCGAAGATACATCTGGGATTTGATAACGCGCTTCAAACACGAGCGAACCATTCTTATGACGACTCATCATTTGGACGAGGCGGAGATCTTGTCGGATCGAATTGCTATAATTCATAAGGGGGAGTTGATGACTGATGGCTCGTTAAGCTCGCTCAAGCGGCAGTTTGGTGACGGCATGAAGTTAACCATCACGTGGAATAACAATCCCCGTCTTCAAGAAACCCGAAATCATCTATTGTCCAAGTTTTGGAGCATGCTTCCCAATGCCAAATACTATAATAATACCGTGGAATCGAATGAACAGTTTTTCATTCCTTATTCTCAACACATTATGCCCAAGGGTCTGTGCCAGTTCTTAGCAATCTTGGAGAAACAAATCGAGCAAAGGCAAGTTGTGTCTTTCAACTTGGAAGGCACCACTTTGGAGGATATTTTCCTCTCCTTTGTTATGGGATCGAGGGAGCACGTGAACAACCCACTCAACTCTAGTGATACGCCTTCCTCGAGACCAACCACATCCAGGTGGAGGCAAGGCCCTTTGTCGGCGCCCTCAACAACAGAAGCACCGAAGCACTTGGGCCTTCGAACTGGATGGCCTCTCGTTTTGGGTCAAGCACGTGGTTTGTTGTACAAACGCTTCAAACATTCCACACGGGATTGGCGGTTTCTGATGAGCTCCCTCGGCTTACCCACTCTGATGGTGATTTTGACCATGTTTTTAGCGCTTATGAGGCCATCCGGTGAGAGTCCGCCTTTATTGTTGACGCCCTCGATTTTTGGAGAAAACTCGAACTCTTTCGTCAGCTTCTCGGAAGAGTCTAATTTGAACAGCATTCTGCAGTCTTTGGTCTCTGATCCTGGTCTTGGCACAACATGTATGGCGGATGTTCCAGATCTGGGATTGTGGACCCCTTGTGACCCAATCATGGGCGAGGCAAACTTTTCAACGGAGTATGAGGGTCCTGGATCTAAATTGACGCAATGTTCCTGCCCTTCTTCCTATGAGTGGACTTGTCAATTGGATTCCAACTCATCCTTATCTCGAGCTCCACGCATGATCATGAATACGACGGATGTCATTTTTGACCTGACCGATCAACCCAGTCCCAGCCACTGGATTTTAAACTCCCACACTCAATTCATTGACAAGAGATTTGGTGGGTGGTCCATTGGCGAACCTAGTCCCTCATCCGAAGTGATTGGTGGCACCACAGAGAATCTGATTGTTTGGTACAACAACAAAGGAGTGCATGCAGTTCCTTCCTACTTGAACGCCATTCACAATGCCATGCTCAGAACCACGGTGGCCGAAAGCGAACAAGATCCAACCCAATTTGGAATCACAACTTACAACCATCCCCTCTTACTCAACGCAAAAGAAGCCACAACGGCTACAATCATTCAAAACGTGGCTGACATTGGGATCGCCCTCATGATGCTGACGTCGTTTTCATTCGTTCCGTCAAGTTTCATTTCTTACGTGATCACTGAGCGGATCCAACGCGAGAAGCAAGTTCAAATTGTGGCCGGAGTCTCTCCGCTGACATATTGGTGTTCCACATTCATTTGGGATTTAGGGGTCATTCTCATCTACACCATTGTGACTGGAATCGTCTTTCAAATCTTCAAGATCGGATCCTACACAGACAAATCCAATTTCTCAGCCGTCCTCGTCTTGATGTTTTTCTTCTGTACAGCCTCAGCCAGTATTGTTTATTGTATAGAGAAATGGTTTTCCGAGCCCAGTTTGGGTCAATTAACCGTGCTCTGTTCCAACGTTCTCTTGGGGATCTTGCTTCTCATGATCATCATTCTTCTAGACATGCTTTATGCGATTAAGGCTACAATGGGACTAGGTCGACTTCGTCGTTTCTTGAACGTGATCTTTCGATTTGCTCCAGCTTACTCTCTGGGTGGAGGATTATTAGCTTTGGCCACTAATTATATCACTGGCGAGGTCCTGGAATCTGCCATGTACGACGAGGACGGGTACAAACCTCCGTTTTCGTTCGACGTGATTGGGCTCAACATCCTTATTTTGGCTCTGGAAACCCTATTCTTCTTCGGGCTCAATCTGGTTGTGGAGTACAAACTCTTAGCCGATTGGTGCCCTTGGTGGACTTCCCCGTTGCCCCAAGTGGATTCGGAAATGACCCAAGAGGATAGTGACGTCAAAAGAGAGCGACAACGAGTGGAGAGTCAACAGCATCATCCGTTCTCTTATCCAAATGTGCAGAAGCCTGACCTCTTGAGAATCAAAAACTTATCCAAGGCTTTTGTTAATGGGAATCAGACCAAATTGGCGGTGAACAATGTGTCTGTGGGGATCAAATATGGAGAGTGTTTTGGTTGGATCGGATTGAATGGTGCAGGGAAAAGTACCACCTTCAAGGTACTAACTGGGGAAATACAACCCAGTGCGGGAGAGTATCAATTGAACCCACCTGACACACTTCGAGGATATTGCCCGCAAGAAAACGCTTTGGACCCACTCCTAACCGTACGAGAAACCCTTCAAGTCTATTGTCAAGTTAGAGGACTCAGTCCGCAAGACTCGAAAAAGGCAATCGATCATTCAATCGTTGATTTGGCCTTAGACACGCAAGGGAACACAATGTGTAAAGATTTGAGTGGAGGCACGAAGCGAAAAGTTTGCGCAGCCGTCGCATTTCTCGGTCAACCCAAGCTCATCCTGATGGATGAACCCACAAGCGGAATGGATGCGGGTACCAAGCGTCAAGTTTGGAAAATCATTCAGAGAGAGGTGGAAAAGGGAACAACTGTGATATTAACGACCCATTCCATGGAGGAGTGTGAGCAACTCTGCAGCAGACTAACCATCATGGCTGAAGGTCAATTGAAGTGCATTGGATCGCCACAACACGTGAAAAAAAAATTTGGTCAAGGTTATAAGGTCCAGTTGTCTTTCGAGTCCCTATGTCTCGCTCAAAAAGCTTTGAGGGTTTTACGTTCCATGTTCAATCGGATCACAAATGTATCTCAACATTACACCAACCTCAGCTTTAATATCCAGGATATGCCCCAAAGTGAGATTTTCGCTCGCATCGTCCCTCGACAAAAGGCACTTGGAATCACCAACCTGAATGTGGCTTCCACGACCTTGGATGAGGTGTTTGTAGGCTTCGCAACCAAACCTAAGAAACAATCTCACGAATGC**TAA**

>ABCB1

**ATG**GGAACCTCCAAAACACACGAAACCCTAAAGGTTCAGCCTTTCACCGACACTGCCAAGGATGGTTTTGAACCTGAGAAAGAAGCCAAGGACAATGATGACAAAGATAAGAAGAAGGAAGAGCCTTCGTTACCACCCGTGGCCATTCATCGGCTCTTTCGTTTTGCAAGTTCCCGAGACCTGTTCCTGATTTTCTTGGCAATTGTGGCGGCCATTATTGGAGGATGTTCCATGCCCGTGATGATTATTCTCTTTGGTGACTTGGCGAACACGTTTGTTCAAAATGATTTGAATGTTACACAAATATGTGTCGGGATTCCGCTTTGTTGCGACGACACTCCAGCAATCAATTTGGATTTGCCCAACTGCAATGTGACTGAGGAGGATCTTGGAAATTTTTTCATCAACATGAACTTTTTGGAACAAATTACTACTTTTGCCCAAGGCACCGCCTTAATTGGATTGGTCAACTTTATCATGAGTTACATCTTTGTGACGTGCCTAAATCACGCCGCAGAATGCCAAGTCTTCAAGATTCGAGGACTCTTTCTCAAGGCAATTTTGAGGCAAGACATTGGCTGGTATGATACCCATCAAACTGGAGATTTTGCTTCACGAATGACTGAAGATCTCAACAAGGTTCAGGAAGGAATTGGCGAAAAGATTGGCATGTTCATCTTCTTCGCCACAATATTTATCGCCAGTTTGATCAATGCCTTTGTCCACGGATGGGAACTGACCTTGGTGATCTTATCAGTGATGCCGGTTTTGGTGATTGCCACAGCTATAATTGCGGGTTCGCAGACTTACCTCACGGCCAGGGAGCTGAAGGCTTACGGCAAAGCTGGATCAGTTGCGGAAGAGGTCCTTTCGGCCGTGCGAACAGTGGTGGCTTTTGGGGGTCAAACCAAGGAAGTTGAGAGGTTTGAGAACAACTTGCATGATGCCAAGAAGGCCGGGATTATGCGGGGCCTCCTGACTGGAATTGGTGGAGGGTTCATGTGGCTCATTATCTATGCTTCGTACGCCCTAGCGTTTTGGTATGGCGTAAAGTTGATCATGGATGACACGGAAGACTGTTTCGAAGACATTCTCCATTGTGATCCTCGATACGATGCTTCCAGCCTTTTAGTGGTTTTCTTCTCAGTTCTCATGGGGGCCATGAATGTGGGCCAAGCCACTCCTTATGTGGAGGCTTTTTCTGTGGCTCGGGGAGCAGCTGCTCAAATTTTTGATATCATCGATCGTGTTCCGGAAATCGATTCTTCTAGTACTGCAGGCGAACATCCAGAGAAAGGAGCCGGGAACTTGACTTTTAGAGATGTGTTCTTCAACTATCCATCAAGAAAGGACGTCAAGATTTTGAAAGGAATGACCTTGGACATCAACAAAGGGGAGACTGTGGCATTAGTGGGAGCTTCAGGTTGCGGCAAGTCCACAGTAATCCAATTGGTCCAACGGTTTTATGATCCCTTGTCCGGCTCTATCATGCTCAATGGCAAAGATCTTCGTCAACTGAACCTTTCAGCCCTTCGAGAAAGGATCGGTATTGTTGGACAAGAGCCTGTGTTATTTGGTTGCACCATTGCTGAGAACATCCGATATGGCCGTGACGGTATCAACGACTCTGACATTGAACAGGCATGCAAGGATGCCAATGCGTACTCTTTCATCCAAAGCCTCCCTAAGAAATATGACACTCTGGTGGGAGAACGAGGAGCTCAACTTTCCGGGGGGCAGAAGCAAAGGATCGCCATTGCCAGAGCACTAGTTCGAAACCCCGATATTTTGCTCCTAGACGAGGCCACTTCAGCCTTGGATACTCAAAGTGAGGGCGTGGTTCAGGCAGCACTGGATAAAGCTCGACGTGGGCGGACCACCATAATGGTTGCACATAGATTGTCGACCATAAGGACGGCTGATAAAATAGTTGCATTTGAAGATGGCAGGGTGGCTGAAATAGGTACTCACGGCGAACTCATGAAAATGGAGGGGGTCTATTATGGTCTGGTCAGTGCACAAGGAATACAAGCGGTCGATGATGAGGATATGGAAGAAGAAGAAGACGATGTTACGGAGCTCGATATGGTTGAGCAAGACATTTTTGACAAAGGAAAAGGAAATAACCGTGTTCGAACGGAAAGTGAAAGGAAAATGAGTGTGGCCAGTAGCATTTTGAGCGACGATTCTGTCACAATGGAAGATATCGGACACGCCGTTGGGTCGGCAGTTGGATTTTCTAGAGTCCCATCCTTACAAGCTTCTTTTTATAAACGTCAAAAGGGATCGTTTACTGATACTCCATTGGAAAGCCCTGATGAAGATCTGCCTAAGGTATCCATGATCAGAATTCTCAAGGCTAATTCCAAAGAGTGGCCCTATATGCTCATTGGACTCCTAGCTTCAGTAATCATGGGAGCTTCCATGCCCGTGTATGCCATTCTATTTGGAGAGGTTCTTGGAGTGTTGTCCGAGGATCCGGTCTCGGCCAGAGACAATGTGTCCTATTATTGCATCCTGTTCCTCATCACTGGCATGGTTGTTGGAATAGCCATGTTTCTGCAAATCTCCATGTTCACTTTAGCTGGAGAGCACTTGACCTTGAGGATGAGGAAACTCGCGTTCGAGGCCATGTTGAGACAAGAAATGGCGTGGTTTGATCTCCCATCTAATAGTACAGGCGCCTTATGCACCCGGATCTCTTCAGATGCGTCTGCCATTCAAGGGGCTTCCGGTTCTCCGTTGGGCACTCTGTTCCAATCATTCTTCACTTTGACCATTTCCATTGGCTTGGCTATGTACTATCAATGGCAATTGGGTTTGGTGACATCGGTTTTCATCCCCTTCGTTCTGGTGGCTTTATACTTCCAAACTAAAATGATCATGGGCAGTGACTCAGTGCAAAAGGAGGCGTTTGCCAGTTCAGCCAAACTGGCCATCGAAGCTATTAGTAATATTCGAACCGTGGCTGGATTGGGTCGGGAAAAGACCTTTGAAGAGCTGTATTTGAACGCCCTTCGCCAACCTCATATGGATGCCAAGAAGCGGTCGCACGTTCGAGGGTTGATTTTCGGATTTGCGCAATCGGTGCCCTTCTTCGCTTACTCGGGATGCATGTTCTATGGCGGATGGCTCGTGGAAAACCAAGATCTGGACTACAAGAACGTGTTCAAAGTAGCCGAAGCCCTTATTTTGGGAACCATGATGGTAGGCCAAGCCACAGCCTTTGCTCCCAACTACAACAAGGCTCTTTTAGCGGCTGCCCGTGTGTTCAAACTCCTGGATCGAAAACCCAAGATCGACGCCAACGACGCCACCGGACTTCGCATAAATGATATTCAAGGGAACATAACGTTCAGCCAGGCCGGGTTTCATTATCCCACCCGGAAGGAAGTGCGTGTCCTTCGTGAGTTGAATTTGGCGGTGCAAGCTGGCCAAACCATCGCCTTGGTGGGCCCTTCTGGTTGTGGGAAGTCCACGTGCATTCAACTGTTGCAAAGGTTCTACGACCTTCACAAAGGAGTTCTCACTGTTGAAGGCCAGAATATCCAAAGTCTGAATGTACCCCAACTCCGATCCCGAATGGGGATCGTGTCACAAGAGCCAGTTCTCTTTGACCGAACTCTGGCCGAAAATATTGCCTATGGAGACAACAGTCGAACGGCCTCAATGGACGAGGTTGTGGACGCAGCTCGGCAAGCCAATATTCACTCATTCATCTCCTCTTTGCCATTGAAATACGACACCTTGGTAGGCGAAAAAGGTACACAACTGTCGGGAGGACAAAAGCAGCGCGTGGCAATCGCGCGGGCACTCATTCGCAACCCAGCTGTTTTACTTTTGGATGAGGCCACATCGGCTTTAGATACTGAATCGGAAAAGGTGGTTCAAGAGGCCCTGGATAAAGCGCAAAAAGGGCGGACTTCCATAACCATCGCTCATCGCCTGAGCACCATCCAAAATGTGAACCGAATTTTTGTCATCTCCAAAGGGCGTGTCGTTGAGGCGGGAACTCATAATGAACTCCTGGCAAGGAAAGAGGGCCTCTATGCCAAGCTATGGGGCAGCCAAACCCTTTCAAAG**TGA**

>ABCB6

**ATG**TATTATTGTGGCCCAAATGACTCCTTATCAAATGCTTGGGTCAATCATGGCTTTGGATTATGTTTCCTGCACACATTGGGGTCCAGCATCATTGGCCTTTGGATTCTTACCTTTGGGTGTATTCAACTTGGATTCTACAAGAAATATGCCACACGTCTCTCTGCAGCGGTCTTGCCTCAAAACAAGAAATTCAAAATCCAAGTGTTTAGTCATTATGCCCTTACCTCATTGGCCATTTTGAGTGTCATAGTCAGACACGCCGTCAGCCATGATCAACTCAACGGTTACGAAATCCTGGATTTGGTGGCCAATCTCATAATTTGGCCTTTGACTTTGACCGTGCTTTTAGTGGAGAGAAACTTTCAACTCCCATCGGCACCATCAAATAGTCATGGCGTGGTCTTGATCTCGACTTGGACTTTAGCCTTTCTTTGGCAGAATCTCAAGCTATTGAGTTTGAACAACGAACCTTTCTTCTACGATTTATCTCACTTGCGTGAACAGATTGAACTGACAGTTTTCTGTTTGGAGTATGCCTTTGTTTTCTCAGTTTTTTTCTTGGGAATGAAGGCACCTGGAATTTCACAAACCCATGACTACTTGTATGGAGAGCACTCAGATGTCGAGGCTATTTTGCCCGATGGATCTCCCAGGAACCAAGACATCCAACCATCTACCTGGTCTGGTTTATGGACCAAGTTAGCTGTGTTACTTCCGTACATGTGGCCCAAAAAGTCATTGGGTCTTCAATTCCGGGTCTTTCTGTGCTTTTTATTGCTTGCCGGAGTCAGGGTTGCCAACGTGTTTGTCCCTCTCTATTATAAAAAGATTGTTGATGCATTAACCCCTGTGGCTTTGGGAGCCGAAGTCAGATTTTGTTGGGATTTGATTGCCATATTTGTTACATTAAAACTTATTCAAGGAGGAGGAACTGGGGGGCAGGGCATTTTGAACAACATCCGGTCATTCTTGTGGATCCGTGTCCAACAATACACCACTCGAGAAATTCAAGTTGGCTTGTTTACGCATCTGCATCATTTGAGCTTACGATGGCATTTATCCAGAAAAACGGGAGAAGTGTTAAGAATCATGGATCGAGGTACTTCATCCATCAATGGACTTCTCTCTTATTTGGTCTTCAATATCTTGCCAACCATTGTGGACATCGTGATTGCCATCGTTTACTTTAGCTCGGCGTTCAATATCTGGTTTGGTCTAATTGTTCTGGTCACCATGATTATCTATCTGGCTGTGACGGTCGCCATGACTGAATGGCGAACCAAATTCCGAAGATCCATGAATCAAGCAGATAATGAGCAACGATCCAAAGGTGTGGACTCCTTACTTAATTTCGAAACAGTCAAATACTATGGTGCAGAACCTTATGAAATCGACCGTTATGAAAAGGCCATCTTGAGTTATCAGGCAGAGGAGTTCAAGTCTCAAACGTCTTTAACTTTCCTAAATGGGGCCCAGAGTTTGATCATTAATGGAGGACTCTTGGCTGGCTCGTTGTATTGTGCTTTGTTGGTGACCCAAAGATATCTAACCCCAGGAGACTATGTGTTGTTTGGAACATATATTTTACAACTCATGGTCCCATTGAACTTTTTGGGCACGCTGTACAGAGTGATTCAGGAGAGCTTTATCAACATGGAAAACATGCTTGAGTTGATGGATGAGCCCCAGGAGATCAGCGATCGTCCCAATGCAATGCCAATCATGGTCACCCAAGGGAAAATCGAGTTCAAAAATGTGAATTTTCACTATGGTCCGGATCGCCCTATTTTAAAGGATATCTCTTTCATAGCAAACCCAGGAGAGACAGTAGCTCTGGTTGGTGCCACTGGTTCAGGCAAGAGCACAATTGTCCGACTGCTCTTCAGATTCTACGACGTCCAATCTGGGTCCATTCTTATTGATGACCAAAACATCCAAACTGTCACTCAAGCATCGGTTCGGAAAGCCATTGGTGTGGTCCCTCAGGATACCGTTCTTTTCAACGACACCATAAGGTATAACATTCGATATGGCCGACCAGATGCCACGGATAACGAGGTCGAAGATGCCGCTCGACATGCGGATATCCATGATAAGATCATGGGTTTCCCCGATGGCTACGATACCAAAGTGGGTGAGAGAGGTCTCAAATTGTCAGGTGGAGAAAAGCAAAGGGTTGCAATTGCCAGAACCATCTTGAAATCCCCTAAAATTGTTCTCTTGGACGAGGCTACCTCTGCTTTGGACACCCAAACCGAGCGACACATTCAGAATGCTTTGCAAGAAGTCAGTGTGAATCGAACCACGATTGTGGTGGCTCATCGTCTTTCTACCATAACAAACGCTCATTGTATTTTGGTTGTCAATGAAGGCGAGATCGTTGAACGGGGAAGGCATGAAGAGTTATTGGGCATTCCGAATGGGAAGTACGCAGCCATGTGGAGCCAACAAAGCCAAAAAAAGGAGGCTGAAAAGGAT**TGA**

>ABCB7

**ATG**GCAGTTTTAGCGCGGGTGATTACGGGACAGTCCCCCCTCCTGTCCCGCCCCTCACATTGTCTCCCGCATCATTTCACGGCTCAACGAAGCGCCTTTCTGCCTAAAAAGGCCTTACGTCCCGCCCCCGCCCCTTTGGCTCCGCCTCGCGCTTTCGGCTTACTTCGTCAATGGGTGCGACAATGTTTCCACGGGACGGGTACGGGTCTCTCGGCCACGAATATACCCAAATCCTCGGCCAAAGTCTCGGGCATGGACATCTTGAAGGCTATGTACGCCAATGTCTGGCCCAAAGACCAAGTCGAAATCCGGAAGAGGGTGGTTTTGGCCTTGGGATTGTTGGTGGGAGCCAAGTTGTTGAATATCTCCGTGCCTTTTGTATTCAAACACGCCATTGATCACTTGAACGCGATCACCAATGGAGCCTTGAATCTGGACACGGCCGAAGGCACGATCACAACCGTGGCCTTGGCCTTGATTTTGGGCTATGGAATGGCTCGAGCGGGATCATCGTTCTGTAATGAGATGCGGAATGCGGTCTTTGCCAAAGTAGCGCAACATAGCATCCGCAAGATCGCGCAAAATGTGTTCAAACACTTGCACCGTTTGGACTTGCAATATCATTTGGGTCGTCGAACGGGAGCCTTGTCCAAGACCATTGATCGTGGATCTCGCGGAATAGCCAGTGTTCTAAACGCTATTGTGTTCAATATCTTTCCCACCATTCTCGAGTTGAGTCTCGTTTGCGGCATTTTGTCTTGGAAATGTGGCCCGCAATTTTCATTGGTGGCATTGGCTTCGGTGGGCATGTATGCGGTTTTTACCCTCAGTGTGACTTCGTGGCGCGCCCAATTCCGACTCAAAATGAATCGGACGGATAATGATGCGGGTAATCGGGCGATTGACTCCTTAATTAACTATGAGACGGTGAAATATTTCAATAATGAAGCCTATGAGGCCAAGGAGTACGACAAACATTTGGCCGTGTTTGAGAAGGCCTCGCTTAAAACCAGCGAAAGCTTGGCCATGCTCAATTTTGGCCAGAACCTCATCTTTAGTTCGGCCTTGACCACGATCATGTTGTTGGCGGCTCGCGAAATCACCGCCGGAAACATGACGGTCGGAGACTTGGTCATGGTCAATGGCCTCTTGTTCCAGCTTTCTGTCCCGTTGAATTTCCTAGGCTCCATGTATCGCGAAATGCGGTTGGCTTTGACCGATATGGAGGTCATGTTTGAGCTGCTCAAAATGGAGCCTAAAGTCCAGAATAAAGTGGGTGCTTCAGCGGTATCGATTACGGCTCAAAATGCGTCAATTACATTTGAAGACATTCAGTTTCAATATCAAGAGGGCAAGAATATCTTGAACGGACTCAGTTTGACCGTGGAACCAGGCAAACGAGTGGCCATTGTCGGTGGATCGGGTTCGGGAAAATCGACCATAATCCGTTTGTTGTACCGTTTCTTCGAGCCTAATCAGGGTCAGGTGTCAGTGGCTGGTCAAAATATCCAAGGCATGGACTTGGATAGTCTCAGACGAATCATCGCGGTGGTTCCGCAAGATTCGGTGCTTTTCCATAACACAATCCGACATAATATCGCTTACGGAAATCTGGACGCCAGTGACGATCAGGTGATGGAAGCGGCCAAGATGGCGGAAATTCACAATTCCATCCTGAATTGGCCCAATGGCTACGAAACCCAAGTGGGAGAGCGAGGTCTGAAGCTTTCCGGAGGTGAGAAACAAAGAGTGGCCATTGCCAGAGCCATTTTAAAGGACACGCCCATATTAGTGTTTGACGAAGCTACCTCATCTCTGGATTCAATCACAGAGAACAGTATATTGAAGGCATTGGATAAGGCCACCAAAGGTCGAACTTCGATTATCATCGCCCATCGGTTGTCGACCGTGGTAAATTGCGATGAGATATTTGTCCTTCATCAAGGTCGAGTTGTGGAAAGAGGAACGCATCGCTCACTTCTGAGTAAACCCAGCTCGATGTATCGAAATTTGTGGGATTCTCAACACGCGGTGCTAACGGACAACGAAAATATTATTCCAGATAGTAGACAAGGC**TAA**

>ABCB8

**ATG**GCCCTGATCCTTCGTTTAGCTTGTCCCACAAGGATCAACGGCCTTCGTCCTTGGTTTCCTCGAAGACTACCTTCGCTAAGGTCAACCGTTCCTACCCGAAAGGTCGCTCAATTTGGCACCACTCTCGGTCTCTTGGGACTGATTCCCGTCAGCCGTGCAAATACCTCCATTTCTCCCGAGAAAAATCGATTGATCGGGTCAAATCCTGACCTCCGACCTATCAATGACACTCGATTTCCATGGCGACAATTCCTGGCCATGCTTTGGCCGCATCTCAAGTATCTGTTAGCCGCCATCGGCAGTGCTCTAGCCGTAGCTTATCTCAACATTCAGATCCCACAGCAATTGGGTCAGGTGGTTAATGTGGTCTCATCGTTATTGAGTCAAGTGGGCGATGCTGAGGGCCAAGTTCGACGGTTCATGGACCAGATTCGGGAGCCGTGTGGCCGCATTATTCAGTTGTACTTGGGTCAAGCCGTGATGACTTGGGGTTATATCTATAGTTTAGCTTGTTTGGGTGAACGTGTGGCGGCAGATCTGCGCCAACAACTTTTTGCCTCGATTGTCGCGCAAGATATCGCTTTCTTTGATGAGCATAAAAGCGGCGAGATCATATCGCGTTTGACGACGGATGTCCAAGATTTCAAATCGGCCTTTAAATTGTGCATATCGCAAGGACTGAGAAGTACGGCTCAAACGGTGGGGTGTGTGGTGGCATTATACTCGATTTCGCCACCATTGACGGGGTTGATGTTGGCGGTGGTGCCCGTGGTCATCGGGATTGGAACGGCTTTAGGATCGGTCTTGAGAAAGATGTCCAAGGCCGCTCAAGCTCAGGTGGCTAAGGCCACGGCTGTGGGTGAGGAGTGCATCTCGAATATGCGCACGGTTCGAGCCTTTGCCATGGAAGATCAAGAACGAGCCATGTTCAATGAAGAAGTGGAGAATGCCCGACGACTCAATGAGAGATTAGGTTTGGGGATTGGGATTTTTCAAGGCGGAGCCAATCTTTTTCTGAATGGAATTGTTCTCACTACGTTGTACTATGGCGGGTACTTGTTATCCACGCAACAATTGACGGCGGGTGATCTGATGAGCTTCTTGGTGGCCACTCAAACTATCCAACGGTCTTTGGGTCAGATGTCGTTGCTCTTTGGGCAAATGGTCAAGGGTCTCTCGGCGGGCACAAGAATATTTGACTATGTCAATCTAGTACCCACCATTCGACTGGAGGGTGGACAAGAGATCCCCTATCACAGTTTAATCGGCGAAATCGAATTCCGCAACGTGGGTTTTCAATATCCCACCCGACCAGACCAACGGGTTTTGGATCAGTTCTCATTGAGAATTCCCGGAGGCAAAATGGTGGCCTTGGTGGGCTCCAGTGGAGGTGGAAAATCAACGGTGGCCTCTCTTTTGGAACGTTTCTACGATTGTAACGAAGGTGTCATCACAGTTGATGGCGTGGATATTCGAAAACTCAATCCCAAATGGCTTCGTGGACGTGCTATTGGCTACATCAACCAAGAGCCCGTTCTCTTTGCTACGTCAGTTATGGAAAATATCCGATATGGACGTCCTTCGGCTACCGATTTCGAGGTGATGGAAGCTGCTCGAGCGGCCAATGCTCACACCTTTATTCAAGGCTTTCCAGACAAATATGACACTGTCTTAGGGGAAAGAGGTGTAACCGTAAGTGGGGGTCAAAAGCAAAGGATTGCCATTGCACGAGCCCTACTCAAAAATCCGACCATTCTCGTCTTGGATGAGGCGACCAGCGCCTTGGATGCTGAATCCGAAAAAGTTGTTCAGGAAGCCATTGATCAGGTGGCTCAGGGTCGAACTGTTTTAGTGATTGCTCATCGATTGAGCACAATTCAAAAGGCTGATGCCATTGCGGTGGTTGATCAAGGTCGAATTGTGGAACTGGGCACCCACGAATCACTCAAGAAATTCGGGGGTATATATGCTAAGCTCATTCGACAACAAGAGCAGAGCAGTTCCCCGAATTCACGAGGCATGGCG**TAG**

>ABCB10

**ATG**ATGTCGTTGGTTCGACTGGGTCATGTTTGTTTGGGAGGAAGCACGCCACGCCTAGGATCCCTTCGCTCGTTTCACGTCTTGCTCCCCAGACTCATGACTCGACCCAATGGAGCTCACCGTTCCATGGGCGGGTTGCGCTTTCAATCTTCGACCACGTCTCAACCACGGCTGACGGAGTTGAAAAGATTGAAGAGCTTATTTGGTCCCGAGAAAGTCAGACTCACGGGGGCTTTGGGCTTATTATTGGTGTCAAGTGGCGTAACCATGTTGGTACCCTTTGCCCTGGGCAAAATAATTGATGTGATATCCAGTGAGACCGCACCCGGATCGACCGATCCTTCTAGTGGTTCCTCCGATCCAGGAAAGCTTCAAGAACGGCTCAAATCCTTGGCCGCCGGACTTTTAGTGGTGTTTACCATTGGTGCCTTGTGCAACTTTGGCCGCGTCTACCTAATGCGCATCTCTAGTCAAAACGTGGCTGCTCGACTTCGGCAGGATTTATTTGCGTCAATTATGCGCCAAGAGACAACCTTTTTTGACAAGAGTAAAACTGGAGAGTTGGTCAATCGACTTTCATCAGACACGCAACTCGTTAGTCAGACCATCACGCAACAAGTTTCCGATGGAACGCGCAGTCTGGTCATGACCTCCGCTGGAGTTGGAATGATGATGTTCATGTCACCTGAGTTGACAGTCGTGGGGTTGAGCGTGGTGCCTCCGGTGGCTTTATGGGCGGTTTGGATGGGCAAGAGGGTCAAATCCGCGAGTAAAGAGTTGCAACAACGTTTGGCCAACCTGACCGAGCAAGCCGAGGAAAAAATCTCAAACATTCGCACCGTATTGGCCTTCGCCAGTCAAAAGAAAGAGATTACCATATACAACGAAAGGCTGGAACAAGTAGTAGACCAAGCCCGACGTGAAGCCGAGATCCACGCCAAGTTTTACGGCTTGACTGGCTTTAGTGGCAACCTCATCATCCTAAGTGTGTTATCATATGGTGGCTCCTTAGTGGCCAACCACTCGTTAACCGTGGGGGAATTGGCCTCCTTTGTTCTTTATGCTGCCTACGTGGGAATTGGACTAAACGGTGTGTCCAATTGTTATGCTGAGGTCATGAAGGGCCTGGGTGCCAGTCAACGAATCTGGAATCTCATGGAGACCTCATCCGCATTGGAGGTTGTGACGCAGCAACCTCGTCCTAATTTGCCTCTAATGAGTTTACCCTCCCTTGAGAAGGCCATTGTGTTTTCGAACGTCAGTTTCGCTTTTCCTACTCGACCCGAAAGCCTAGTGCTCAACAATCTGAGCTTCACCATTCCCGCCAACCAAACCGTGGCTGTGGTCGGAACAAGTGGATCAGGCAAGACCACATTGATTTCTCTTTTGTTGAGACTCTACGATCCGGCCCAAGGCCAAATTTGCTTTGATGACAACGATATCAAAAGTTTTGATGTGGCGCAGCATCGTCGACGAATCGGTCTTGTGCCTCAGGAGCCCGTGTTATTCTCGACGTCCATTGCCGAAAATATCTTGTATGGACTGGACGTGCGGTCTGATTCCAATGAATGTCAACAGGAGAAGACCATGTCCTTGGTGCACGATGCTGCACGAGAAGTGAACGCTCATGAATTTATAATGCAATTCCCAGAGGGCTACAACACTCTCGTGGGCGAGAGGGGAATCTTTCTGTCAGGGGGACAAAAACAAAGAGTTGCCATTGCACGGGCCATCATCAAAGATCCGGATGTTTTGGTCTTGGATGAGGCCACGAGTGCATTGGATGCCAAAAATGAAAATATCATACGCGATACAATCAATCGAGTGCGACACAATCGAACAGTCCTGATCATCGCTCATCGATTGTCAACCATCAGAAACGCAGACAAGATTATTGTTCTACGCCAAGGCCAAGTTGTTGAAGAAGGATCCTTCCAGGAGTTGGCCAATTTGAAAGGACACTTCCACGAGATGGTGACGAAGCAGTCCATTAGT**TGA**

>ABCC1 isoform X1

**ATG**GGCTTCTGTAACGAGAGGTTTTGGGACCCCGAATTGATCACTCATGACGTCAGACCGAATTTTACCCCATGCTTTCATCAAACCGCATTGGTTTATGTTCCGTGCGGTTTCTTGTGGCTATTTGTGGGATATTACGCCAAATTATTCAGAGCTCACCGGAACAACAAACTTTGGTTGCCTTTGAAATCGCCGCTCTTTGCTTGTCGTGTGATTGTTGTGTTTGCACTGACTGTGCTTCAGATTGCCAATCTTCTGAACATTGGACAAGAAAGGGACCGACCTTCAAGTGAATGGCTGGCATCCCTGGTGTTTGTGGCCACTTTTGGTTTGCAAATGAGTGCCACTTTTCTTCACCATCAAGAGGGCCATGTCCGGTCTTTTATCCAGCTTTTCTTTTGGTTGATTCTGATGGTATTCCAATGCCCAACTTTTGTATCCGCCATTCAAGTGTTTCATTTTAAGGATCCTGGCCATGTTTATGAGTTGACACAAGCCGGAATCCGAATTGCCTTTTTCTTATTTATCWTAGTWGGGTGTGTACTTCAGTTTTTCTCGAATGTGTCAACTTCTGAAAATGTCTCAGAGAGTCCTGAAGCTAMAGCTTCCGCACCTTCACTACTACTTTTCACGTGGTTTGACAAACTGATTTGGAGCGGGTTCAAAACGCCATTGGTGAAGAGTTCTGTGCCTGAAGTAGGTAAAGAACTTCAAGTTAAGACAATTTCTGACAAATTCAAAAGACAATTCACAACAGATCGTGTCAATATTACAAATATTGGTCATTCAAATGACGCTTTTTGTTCAACCAATTCCGACAAAGTAAATGGGGAAGGTGCTGGGGTCCATTTGGAGATCAGTGCAGTGGTCAAAGAAAAAGTGCACATTGAAAGGGCTCTGATTAAAGCTTTTGGAAGAAGATTCTTCCAATCTGTTCTGCTAAAAGTGATCCAAGACATTCTGAAGTTCATCGCTCCACAAATATTGAAAAAACTCATCCGGTTTGCCCAATCTAGTGATCCAGACCAAGGAGTTTGGAAAGGCTACTTTTGGGCCTGTTTCTTGTTCTGTGTGAACCTTGTTCAAATCTTGGTGCTTCAGCAATACTGGAAACAGTGTTACCACGCAGGCATGGAGATGAAAACCGCAACGGTCAGTGCAATATACAAAAAGTCCTTGAGGCTCTCTAACTCTGGTCAGAGACAAACGGTTGGACAAATTGTGAATCTTATGGCATCGGATGCCTCCACGATTGAGGAAGTTCTACCTCGCGTTAATATGGTGTGGTCAATGCCATTCCAGATCGTTTTGGCGGTGTACTTCTTATATCAAGAACTGGGACCCGCAGTGTTTAGTGGGGTGGCCATATTGCTAGTTCTAATTCCATTCAATATGGTCACCAGCCGATATGATCGAAAATTTCAGTCCGCATATATGAAGGTCAAGGATCAAAGGATAAGGTCCATGTATGAGATCCTCAGCAATATCAAAATCATCAAGTTCAACACTTGGGAAGAGGCCTTTGCTGACAAAATATTGTCGCTTCGCGCTCAAGAGCTTAAATTCCTAGAAAAGAAGGCCGCTCTTCAAGCCTTCATCAATTTTGTGTTTGGCTCGGCTCCTGTTTTGGTGACCCTTGTCAGTTTTGCCACCTATGTATCAATCAGTTCAAGCAATCATTTGACTGCAGATAAAGTGTTTGTTTGCATTGCCCTCTTCAATCTGTTGAGACTGCCAATGCATCTTCTTCCTTGGTCTATCACGGAGACACTGAGACTTGTTGTAAGTATCAAGCGGATCAACAAGTTTTTCGAATCCAAGGAGCTTGATCCAACTCCCCTTAAGTTGGGAAGACAAAATACTCATTTGAAATATTCGGTGGAAATCAGGAATGGATCTTTCGGTTGGGAAGATCCGACAGTAGCCACACCCATCTTGAAGGACATAAATATCGCTATTCCCCAAGGAAGTTTAGTGGCGGTGATTGGCCAAGTTGGATCGGGCAAGTCCACGCTACTTTCAGCTATCTTGGGGGAGACTGAAAGGTTTGAAGGCGAGGTTTTGGTGAACGGAAACACAATCGCCTATGTCAGTCAAGAGCCATGGATTCAAAACATTTCGGTGAAGAACAACATTACCTTCTCCCAAGACAAACCAGATGAAAATTGGTACAAAAAGGTGGTTGAAGCTTGTTCCTTAGAACAAGATCTCAAACAACTGATGGATGGGGACCAAACTTTGATTGGAGAGAAAGGGATTAATCTGTCTGGAGGACAAAAGCAACGAGTTTCAATCGCACGAGCAGCTTACTCCAAGGCCGATCTTTACCTTATGGATGACCCTTTGTCGGCATTGGACTCTCAAACTGCCAACCATGTCTTTGAAGAGGTCATGAGTAATTCCGGAATCCTAAGACACTCGACAAGAATATTGACCACCCACAACACAAGTGTTCTAAACAAAGCCGATTTTATTGCTGTTTTAAAAGATGGCCAATTAGTCGAATTCGGCCCTCCCACAAAAGTCATGACGTCATCATCAGAGTTATCTCGCTTAGTCGAACATTTTGAGGAAAGAGAAGACGAATCAAAAACTGAAAACCCTGAAGAAAACACAAACAAGTTTGAAAAGCTCAGAAAACCTGTGGCCAAGGATAAGAAAGAAACAGCTCTGGCTCAAAGATTAAAAGACGAAGAGGTGCTCACTGGTAGAGTCAAAAGCCGAGCTTACCAAAAATACCTGGGAGCCTTGGGGGCTCCAATTTGCGTTCTCATTCTTGGTCTTTTCATGGCCAATCAAGCGATTTCTACAAGCTCAACAGTTTGGTTAGCTGCTTGGTCAGATCATAACTCACAACTTGCTAATCATTCTCAGACTAATTCCAATACATCCTTGACCGTCGAGAAGGATCCAGACAATGGCTACTACATTGGGGTATTGGCTGCTTTTGGAGTAGCTCAAGCAATCTTGAGCTTCTTGAGAAACCTGGTATTCTACCTTGCTTGTGCCAGAGGTAGCAAGATCATCCACAACTCATTGTTTGACGTCATAATTCGTGCCAAGACGAGATTTTTCGATACCACTCCAACGGGAAGTATCATCAATCGGTTTTCCGCTGACATGAATACCGTGGATCAACCTCTGCCAAATGCCCTATCAAGCTTCCTGTTCACCCTAATGGAGGTCACATCCGTCCTCATTGTCATCAGTATCTCTACACCTATCTTCATGGTGATCATTTTGCCATTGGTTATTGTTTACGTCTGTCTCATTCGAGTGTACATTCCAAGCTCGCGGCAACTCCAGAGGTTCGGTTCTAGCACCAAATCTCCAATTAATCTCCACTTTAGTGAAACCATTCAAGGAATTTCGACCATCAGAGCATTTCAACATGAAAATATGTTTACTGACGAGTTTGAATCTCGAGTCCAGGAAAACCTTCGTTTCCAATACGCCTCTGTAATGGCCACTCGTTGGTTGAGTTTGCGAAGTGAAGCATTGGGGAACATTGTGGTTTTGGTGGCTGCATTGGTTGCAGTCGGCCAAAGAAGTGCTTTGACGGCTGGATGGGTGGGATTGAGCATCACTTATGCATTGAGCATAACAGAAACATTCAACTGGGTCCTTCAGAACGGTTGTAAACTGGAGGAACAAGCAGTCAATCTGGAACGAATCCGCGAGACCGAAGAAAACGCCCCAGTTGAAAGGCCATGGAGAAATCCAGAAGTCGACCCAAAAGACCGACGATGGGTGAAGAGAGGGGACATTCAGTTCCATAACCTTACCCTGTCTTACGATGAAGATCTTGATCCAGTTCTCAAGGGTGTTAATCTGACCATAAAATCGGGGGAAAAGGTCGGGGTCTGCGGACGAACAGGAGCGGGGAAAAGCTCTTTGGCAGTGGCATTGCTGAATTTGGTAGATTCTTGGACTGGTCAAATGCTCTTGGATGGCGTTGAAGTCCGGGCATTGGGTTTGCATACACTCCGATCTCAAATCACTATGATTCCTCAAGACCCTGTCATGTTCAAAGGCACCATCCGGCAGAATATTGACCCTTTTAATAAATACAAGGACGACTTTATCTGGACCAGTCTGACCAACTGTAGGATGGATATTCATATCCGAAACTTTGCCGCAGGCTTGGAACATAAAGTCGAAGAAGGTGGGTCAAATTTCTCCGTGGGTCAAAAGCAGCTCATTTGCTTGGCAAGGGCCTTACTCAAAGACAACACGATCGTGTTTCTTGACGAGGCAACAGCTTCAATGGATGCCGAGACAGATCAGAGTATTCAAGAGACGCTTTTCCGCGAATTGGAGGGAAAAACCTTGATCACGATTGCTCACCGCTTGAACACAGTATTGAACTATGATAAAATATTAGTTCTTGATGAAGGCAAAGTTGTGGAGTTTGACACTCCACAAGCGCTTTTGGAGGATGAATGCTCTCAATTTTATTCCATGATCCATGAATCAAGT**TAG**

>ABCC1 isoform X2

**ATG**AGCTCTTTTGAGGAATTCTGTGGAGGTCCAATTTGGGACGTGAATGTCACTTGGAATACCAAAGATCCCGATTTCACCACTTGCTTTCATCAGACCATCCTTCTCTACATCCCAACCGGCTTCTTGGTCCTTTTCTCACCTCTGGAAGTGTACTTCCGCATCTATAGAGGGTCCAATAAGCCCGTTCCCTGGACCCTCCTCAACATTGCCAAGTTAATCCTCAACGGTATTTTAGTTATCCTTCCAGTTATTGACCTTGGCTATGCCATAAACGAAGATGCTAGAACAGTCCATGTGGTGGCCGCTATTGTGAAAATTGTCACCTATGTGTGTGCTCTCTGCTTGGCTGTGGCAAGTCAACGTCGAGGCGTGGTCACTTCGGGATTATTGGTCATTTTTTGGTTTTTGGCCGCAATTGGCGGGGCAATTACGTTTGCCTCTGTACTAAGCACACCTTACCTGAGTGGGGATCATTTTATGCTGCCATTTTTATCTTACACTCTCCAATTTCCTTTGATCCTTGTTCTATTTTTTCTCAATTGTTGGGCAGATGCCCCCTCCAAGTACAAAAAATTGGAAGAGGATGTTCCCAACTTAACACCGGAGAAACATGCGTCATTCTTTTCTCGAGCGATTTTTGCTTGGTTTGATCCGTTTGCTTGGAAAGGATGGAGACGAACTTTGGAATACACCGATTTATGGGACTTGAAGAAGAGTGAGAGGTGCTCTGGCATCGTTCCGGAATGGGATCGTCAATGGGAAAAGAATGTTATCAAAACGATTCAGAACTCGAAAGCTCCCTCACAAGTGTCGATCACGTCTACATTGATGAAAAGTTTTGGGCCTGCGTATGCAATTTCCGCACTTTATCAGCTTGGAACAAGCGTCTTACAATTTGCCAGTCCCCAAATCGTCAATTTGATCATCGATTTCGTCGAGTCTGATCAGCCTGTTTGGAAAGGATACCTTTATACTGGATTGATATGTTTGTCGACGTTGTTCAACACGATTTTGAATAACCAATGCTTCTATCAAGAGTACACGGTGGGACTCAGAATCAAAACAGCCCTGATTTCCGCCATATACAGAAAGTCCGTCAAACTTTCCAATTCGGGTCGAAAAGTAATGACAGTGGGAGAAACAACTAACCTCATGTCTATTGACACACAAAAGTTTATGGACCTAACTCTTTACTTGAACATGATTTGGGCCAGTCCCCTCCAAATTGCCTTAGCGATGTACTTTTTATGGGACATTTTGGGACCCTCGTCCTTAGCAGGTTTGGCGGTTATGGTGCTTATGATACCTCTGAATGCAGTGGTAGCTCAGCAAATGAAGAAATATCAACACGCACAGATGAAAGACAAAGATCGTCGAACTCGACTCATGGATGAGATTCTCAATGGCATCAAAGTTCTGAAACTTTACGCTTGGGAGCCTTCTTTTGAAAAACACGTGGTTGATATAAGAGCTCGAGAAATTTCTGCCTTGAAAAAGTCCGCTTACTTGAATTCTTTCACCACTTTCTTATGGACGTGTGCTCCAGTGTTGGTGGCTTTGGCATCGTTCGCCACATACGTTCTTTCCGATTCTGCCAATATTCTTGATGCCAACACTGCCTTCGTGTCCTTGACCTTGTTTAACCTCTTGCGCGTGCCACTGAACATGCTTCCGATGCTTCTGGTATTTTTGGTTCAATGCGAAGTCTCCCTGACAAGGATCAATACTTTCATGAATTCGGATGAACTTCGAGTCAATGCTGTGGACAGAAACCCCAATGCCAAGGGAGCAATAGTTGTGAAAAAGGCCACCTTCGCTTGGGACAAGGATGCAGAGCCAGTTTTACGGGACATTAATTTAACTGTAAAGGAAGGAGAGTTAGTGGCTGTGGTTGGCACCGTGGGTGCCGGAAAATCATCCCTTTTGTCCGCATTTTTGGGTGAAATGGAACGTTTAGGCGGATCCATCAAAACGAACGGCAAAGTTGCCTATGTTCCTCAACAAGCTTGGATGAAAAATGCCAGCCTTAAAGACAACATCTTGTTCAGCAAATCATATAAATCTAATTTATACGACCGAGTCCTTAACTCTTGCGCATTGGTCACTGATCTAGAGATGTTGCCAGGAGGTGATGAGACAGAAATCGGCGAAAAGGGCATCAATCTCTCCGGGGGACAAAAGCAACGAGTGAGCATTGCTCGCGCTGTTTACAGTAATCGAGACTTGTACCTTCTAGATGACCCTCTCTCTGCCGTGGACTCGCATGTAGGAAAGCATATTTTTGAAAATGTGATTGGGCCCCACGGGTTATTGAAAAAGAAGACCCGTGTATTAGTTACCCATGGGGTCTCTTTCCTCCCTCAAGTGGATAACATTCTAGTCATGAAGGACGGGCGAATTTCGGAGCGTGGGACTTATGAAGAACTGTTGAACAAACAAGGTGATTTCGCAAGTTTCCTGATCCAATATTTGACCGAAGAAGGGGATAAAGAAAAGGGCCTCGAAGAAGAGTCTGAATTGGAGGACTTGAAACAAGAACTGGAAAAAACCCTTGGAAAAGAAGCTGTCCAAAGACAATTGATGGCGAGCAAATCTGTACTGACATCGCTGACTGATTTGATATCAGAAAGTTCAGAAAAGGGTCAACATTTCAATCAGAAAGGGAAAAAACGGTCGTCATTAATGTCAACGTCGAGGATTCATCCTTTTTCGAGTAATAGCAATATTCCACCGTCGGAGCGGGGGCTTGATGAGATTAAGGTGGGACAGAACTTAATCGAGAAGGAAAAGGCCGAAATTGGAGGAGTGAAATGGTCCATCTACAGATATTATGCCAAATCAATTGGCACCAAATGGTCCTTGGTCACGATTCTCTTTTACGTCATTTACCAAGGGTTCCAATTGGGAGCGAATGTTTGGCTATCGGAATGGTCTACGGATCCTCGTGCCACAACTGACACTTCAGTGCGGGATATGTACTTAGGCGTCTATGGTGTTTTGGGATTGCTCCAGTCTCTAGCCATCATGACAGGGACCTGTTTTGTTATGGTTGGAACATTGAATGCCGCAGCCAAATTACATTCCACCATGTTACATCGCATTTTACGCTCGCCAATGTCTTTCTTCGACACAACACCACTTGGACGTATTCTGAACCGGTTCTCAAAAGACATCGACATCATTGACGTGACCATACCTATGAATATTCGCATGCTCCTGAATCAGTCCTTCAATGTTCTGGGAACACTGGTTGTGATTTGCTTTGCCAATCCCATCTTTATTGCCGTTGTTATTCCTATCATTCTGATGTACTACTTCCTTCAAAAGTTTTACGTCACGACAGCCCGACAAGTCAAACGAATGGAATCCATAACACGATCCCCTATCTATAGTCATTTCGGAGAAACAATCAGTGGAGCTCCGACAATTAGGGCATATGAAATGGTACAAGATTTCATTACGGAGAATGAGAAGAAGATCGATTACAACCAAAAGTGCTATTACCCAACTTATGTTTCAAGCCGATGGCTCTCGGTGAGATTGGAACTGATTGGGAATTTGGTCATTTTATTTGCATCGCTTTTCGCCGTTCTCTTTCGAGACACAATGGATCCGGGTAAAGTAGGATTGTCCTTGAGCTATGCCTTGAATATCACGGGCCCGATGAACATGTTGGTTCGAATGACGTCCGAAGTTGAAACAAATATGGTCAGTGTCGAGAGAATCAGCGAATATCAAGAAACACCTCAAGAGGCTCCTCTGGAAATACCGGAACAAGATCCACCCCCAGAATGGCCCCAGTATGGAGTGGTGAAATTTGACAACTATCAGACTCGATACCGAGAGGGCTTGGACTTGGTTCTTAAAGGAATATCCGTTCAAATTGAAAGCGGAGAGAAAATTGGCATTGTGGGTCGAACTGGGGCGGGCAAATCATCGTTGACTTTGGCTCTCTTCCGTATTATTGAGTCAGCAGGCGGTTCAATCTACATTGACAACGAAAACATTGGTCTACTTGGACTGTCAAAACTCCGATCAAGGCTGACCATTATTCCACAAGACCCAGTGCTCTTCTCTGGCTCCATGCGATTGAATTTGGATCCTTTCGAGCAATACAGCGATCGCGAAATTTGGCGAGCCTTGTCTCATGCTCATCTCAAATCCTACGTGTCGAATCTTCCCGGAGGACTTAGCTTTGATGTGAATGAAGGGGGTGAGAATTTGAGTGTGGGTCAGAGACAATTGGTTTGCCTGGCTAGAGCTCTTTTGAGGAAGACCAAAGTCCTGGTTTTGGATGAAGCTACAGCCGCAGTGGATTTGGAAACAGACGATTTGATTCAGACCACAATCAGAGCCGAATTTTCTGATTGTACCGTCCTAACTATAGCTCATCGATTGAATACAATCATGGACAGCTCGAAGGTATTGGTCTTGGATGCAGGACAGATCAAGGAATTCGATACGCCCGAAAACTTAATGGCCCAAAGGGATTCCATATTCTACGGAATGGCTAACGATGCTGGTTTGATCAACAACTTGGAT**TGA**

>ABCC1 isoform X3

**ATG**TTCTGCCCTGATCCCTTCTTCAATTCAAGCATCACATGGGACACGGATGATCCAAATCTCACTCAATGTATGAGAGACACCGTGTTAGTTGGTGTACCTTGTGGAGTGTTCCTTCTTTTGGTTCCTTTTTGGAACATCCACCAATTTCAATCCTATTCGAGACGGGATGTCTTTCCCAGAATCAGAGGCAACATCAGTGTTCGATTCGTGCTCAAAATCGTGTCTACTTTAGTTCTTATTGCCAATACCATCGTCAGAATACGGAATCTTGGTGGATCTCGAACAATATTTGGCTCTGAGATATTCCACTATGGATTACTATGTTTCACGCTCTTTGTGGTCCTTGTGCAAGAATTCATCGACAGAAGATTCCTTTGTGCCATTAAATCTCTACCTCTCTTTTCATTCTGGTTTGTTCTTTCATTAGCAAGCATTCCGGGCTTTAAATATGAGATTGATCAGGCTCTGGAATCTTCACAAGCTGAAGATTGGGCCCTCATACTCAGTTACTATCCTCTTGTGTTAGCCCAATTTCTGCTCAACTGTTGGTCTGATTTGGGAGGAATAACTGACGATGAGCTCGCTCCACCAGAGGATTCAGCCTCGTATCCATCCTTGCTGACCTTTTCGTGGCTTCATCGATTGATCTGGAAAGGATTTAAATCCCCCTTGACCCTCAAACAACTTCCAAAGCCTCCCAAAACCATTTTGGTAGCAACCAATGTCCTACGATTTTTGAACGCTTGGACCAAGAAGGTGGCCTCTATGGGAGTCAATTTTGAGACTGAAGGCACAAGATTTGTCAGTGTGTTCCCAGTCTTGTGGAAATGTCACTGGCCTCGGATTTTGCTTTGCATTGGTCTCAGTGCCAGTCATTACCTCATTGGGTTCATCAGTCCACAAATTCTCAAACTGCTAGTGAACCACGTGGCTTCGCAAACGGATCAAACTTGGAAAGGATATCTTTATGTGGTCGCATTGTTCCTGGAATCTTGCGTGTGGACCTTTTCATTTCACATGTATTGTCAAAACGCGTCGGTTCTTTCCATTCAAATGCGATCCAGTATGATGGGTATGGTGTTCCGAAAAGCTTTTCGTTTGTCGAACTCATCCAAGAAACATTACACTACTGGTGAGATCACCAATTACGTGTCTGTGGATGCCCAACGTTTGGTGGACACCATGCCTTATTTGAGCAATCTTTGGTGCGCACCTTTGACCATAGGTTTGGCTCAATACTTTCTCTATCAAGAGCTTGGACCAGTGAGCTTTGCTGGGATTGGAACTTTGTTCCTTCTGATGCCCATTAATCTGTTCACGGGGAAATGGGTGGAGCGTCTTCAAGCCGATCAATTGCGGAACAAGGACGAGAGGATCAAGTTGATGTCCGAGATTTTGGGAGGCATCAAAGTGTTGAAGCTCTATGCTTGGGAAAAGCCTTTCATGAAGCGTTTGCTCAATATACGCGACAATGAGATCAAGATCCTGCAATTCAGTGCCCGATTAAGAGCGTTGATTAATTTCACATTTTTCTGCAGTCCAATTCTGGTCACGATCTCCGTGTTTGGTTTGTACGTTTTAGTCGATTGCCAAAACACATTGACGGCAGAGAAGATTTTCGTTTCAATGTCTCTTTTAAACATGACTAGATATCCTCTGATTATGTTTCCTTGGGCCTTGATTGAGGGGATCAAATTGATAGTCTCGCTCAAACGGATCAACAAATTTCTCAACGCCAAGGAAATGAATTCATCTTTGGTCACCAAGGAAGTGATTGATCCCAATAATGCGGTGGAATTTACATCCGCATCGTTCACATGGAACGAGGGTTCGGATGCCATAATTCAAGATATAAATTTAAAGATCCCGTCAAGGAGTTTGGTGGCTGTAGTGGGTATGGTTGGATCAGGAAAGAGCTCACTCTTATCTGCTCTCCTGGGAGAAATGGAAAAAACTAGTGGCTCAATCGGCAGGGTCGGGTCCATAGCTTATGTGCCACAACAGGCTTGGATTCAAAATATGACCCTGAAGCAAAATATCTTGTTCGAAAACGATTATGAAGCGTCAAGATATCACCAGGTCTTGGATAAATGCCAACTTAAACAAGACTTGGAAATCTTGAAATCAGGCGACGAAACAGAAATTGGAGAGAACGGGATCAACTTGTCTGGAGGTCAAAAGCAACGCGTGAGCTTAGCACGTGCTGTGTATTCTGATGCAGATCTTTACTTATTGGACGATCCTCTTTCCGCTGTCGATGCTCTCGTCGGCAAAAACATCTTTGACGAGGTTCTTGCCAACGAAAACGGGTGCTTGAGAGACAAAACCAGAGTCCTTGTGACGCATAACTTGAATGTTCTCCCACGTGTGGATCATATCGTTGTGATGAGACATGGAAAGATTGCCGAATCAGGATCCTACGAAAAACTGAAAAAGGATGGAGGAGCCTTTGCAGAATTTCTCGAGAACTTCACGTTCGACAAGGTTCAAGAAGAGATTGTCAGTGCCTCGCCTTCTACTAGTGTTCCAATTCGACAGCAGAGCTCAGAGAGCAATGTGAGTCCTTTTGGTTCGCCCAAGTTCCAAGGGATTCCATATGTGAGGCACGACTCTTGGGAGGAAGCCAAGCAATTGATTAGACCGGGTGACAATGAAGAGAGATTGGATGAACGAGAGCCTTTGGTTCAGCCATTGGAATCGATCCACTCTGGCAATGAAACACTTTTGATGGAAGAGTCATCCTTCCAAGGATCTGTGAAATGGAGCGTGTTCCTGACGTATTTCAAGGATATTGGGTTTTCCAAGTTCCTTCTCATCGTTATATTGTTCGTGACAAGTCAAAGCTGTCATTCGTTTGCAAATTACTGGCTCTCAAGATGGGCTGACGTCAATCACAAATGTCCGGACAAATCGGCAGAAAATATCCTCACGTATCTTGGAACCTACAGCGCTTTAGGGTTGGTGGAGATTGGAACCGAGTTTGCTTACGAATTTCTGCACTTCCTCAGTTGTGCCTACGCATCCAAACTCATCCATGAACGCTTGCTTGAACGCGTTATGCACTCACCCATGTCTTTCTTCGACACCAATCCAATGGGACGGATTCTCAATCGGTTTTCCGTCGATGTGGACTCTTTAGATCAAAACATTCCGTCAATGATTCGGGACTTTTTATGGTGTATGTGCGATCTAGTGAGCATTATCGTCATCATCACTTACTCCACACCCTGGTTTTCTGTCACCATAATTCCTCTCGGTGTGGTCTTCATGATGTTCCAAAGGTATTATATAGCCACATCTCGCCAATTAAAGCGACTCGATGCGGTTAGCAAATCACCCGTTTTGGCCCATTTCTCGGAAACCATTACAGGTGCGGCGTCAATTCGAGCATACCGACAACAAGATAGGTTTATTGAAGAGTTCGAAACGCGATTGGAGACGAATATCAAGTGCTACTATCTCATGGTCTCTTGCAATCGATGGTTGGGTGTGCGTATGGAGCTCTTGGGGAACGTCATCGTGTTTTTCGCAGCCTTGTTCGTGATCCTCAAACGTTCGAGTTTGTCTCCAGGAGTGGCGGGACTATCAATATCATATTCCTTGAACATTATGGACGCCCTCACATGGATGGTGAGAATGGTTTGCGAGTTGGAAACAAATTTTGTGGCCTTGGAGAGGATCCTGGAATACACGGACAATCCTCAAGAAGCGCCTTGGGAAGTTCCAGCCATTGATGACAACCTTCCCCAAAATTGGCCCTCTGAAGGAAAAGTATGCATGAAGGCCTACAAAACTCGTTATCGTGAGGGCCTAGACTTGGCACTCAATGGTATTGATCTCGAAGTAAAAGCCCATCAAAGTATCGGCATTTGTGGCAGAACTGGAGCTGGCAAAAGTAGTCTGTCCCTGGCCCTCTTTCGCGTTATTGAAGCTTGTCATGGGACCATTTTCATTGATGACGTGGACATCTCAACATTGGGACTCCATCGACTTCGATCTTGCTTGACCATCATCCCGCAAGATCCCATCCTATTCACAGGGAGTGTCCGCTTCAATCTGGATCCAACCCAAACCAGTAGCGATTTTGATCTGTGGAACGCCTTGGGACATGTTCATTTGAAGCCAATTGTCGAGAATTTGAGCGAAGGTCTTGACCATCAAGTGTCTGAGGGAGGCATCAACTTCTCTTTAGGACAACGTCAACTGATCTGTTTAGCCAGAGCCCTTTTGAGGAAAACGACGATCCTCGTTTTGGACGAGGCCACAGCAGCCATTGATTTAGAGACAGATAACCTTATTCAAGCAACTATTCGCGAGGAATTCAAGTCTTGTACCGTTCTAACCATCGCTCATCGATTGAATACCATAATGGACAACGACAAAATTGTGGTCCTTTCGCAGGGACGAGTCTTGGAATATGACAGTCCAGCTGCCTTGTTGTCCGACGAAAACTCGACCTTCAGTGATATGGCTAAGGACGCCGGCCTT**TAA**

>ABCC1 isoform X4

**ATG**AAGTTTTGCAACACTCCATTTTGGGATATCAACCAAACATGGAACACAGAGGAACCAGATTTCCCTTTGTGCTTTCATCAAACCGTTTTGGTCTACGTACCATGTCTATTTTTATGGCTCCTCACACCAGTGGAACTCTGTTGGATCAACTCCAGTCAGAGCAGGAATGTTCTTTGGAATCCCATCAACATTGCCAAGTTCCTTTGTATTGGTGTTTGCTTGATACTGGACTTGATCAACTTGAGTTTTATCTTGGTGCAACTTGGGACATCGAGCGACATCGTGGCCATTTCTGATGTCTTGAGATCGTTGATATTGGTCTGTACTCTTGGTTTGGCTCTAACCTTAACCTTTCTGAATAAGCGAATGGGTTTGACCACTTCAGCCATCTTGTTCTTGTTCTGGTGGTTATTGGTTTTGTGCTTCAGTCCAACTTTTGCTTCAGTCATTCGCTTTGGAGCCATAGCTACGCCAAGGCCAATGTGGAACATGGAACAATCTGTAATATACACGTTCTATTACGCATTTTTGGTAGCCACCGCTTTTCTGAATTGTTGGGCAGATCCCCCACCCACATATTGGCAGATTGAAAAACAAGTTGAGACTCCATCGCCAGAACTATTTGCGTCTTTTCCAAGCAAGGTCTTGTTCAGTTGGATGAACGGACTATTCAAAATGGGGTGGAAGACTCCTCTGAATGGGAATAATATGTATGACCTGAAACCAGATTTGATGAGCCGATTCTTAACAAAAAGGTGGAACAGGTTGAAGAAGTTCAAAATGGACCCTGTGGCATATGAGCCTAAGGTCAGCATTTATAAAACGCTCGGTCGCTCATTTTGGCTGTCCTTCCTTTGGGCAACTTTCTTTCAAGTGCTCACTTTGATTTTAAATCAGTTGTCACCTCAAATTTTGAGCCTTTTGATCAGCTACACCACGAGTTCTGAACCAGAATGGAAAGGCTATCTTTACATGACGCTAATGGTGGTGGTGAACATGCTAAAAACCTTGACCCTCTCCCACAACTTTTTCATGTTCACCGTAATTGGCCAGAACGTTCGCACCATGCTTATTTCCGCCATCTACGAAAAGACATTTCTATTGAGTTCATCTTCCAGACGACAAAGATCAGTTGGGGAAACTGTGAATCTTATGTCGATAGACTCGGAGCGAGTCTCTACAATCATTGGTTCCTTGAACACAATTTGGTATAGTCCTACAGTGATCATCTTGTCCTTGGCTTTCCTATGGGAATACGTTGGACCATCTTGTTTGGCTGGTTTAGGGGTCATGATCATTCTAATCCCGGTGAATGCAATCCTGTCAGCCAAGATCAAAAAGTATCAAGTGATTAACATGAGGAAGAAGGACGCTCGATTAGAAGTGATGAATGAGATTCTGGATGGGATCAAAGTCATGAAACTCTATGCTTGGGAGCCTTCGTTTGCTCAAAAGGTCAGCAAGGTCCGCGATGAAGAAGTTGAAACTTTGAAGAAAATTTCGTATCTCGGCGCTGTTCAGACTCTTTTGTTCAATTCGGCTGTGTTTTTGGTCACTCTTGCCACTTTTGCAACCTATGTCTTGGTGGATCCTTCCAACATTTTGGATGCTCAGAAGGCTTTCGTGAGCATTTCGTACTTCAACTTGATGCGAGTGCCCTTGAATCAATTACCCATGTTAATCATTCAAGCCATTCAAGCCTTGGTTTCTCATAAACGTTTGGATTCTTTCCTCAACGCTCCCGAGATTGACGAAGATGCCATTACCAATGATCCTGACAACAAGGTAGCCATCCACATTCAAGATGGTTCCTTTGCTTGGGATCATGATTCAAGTCTTCAAAACATCAATCTGAAAGTCAAACGAGGCTCTTTAGTCGCTATTGTGGGACAAGTTGGGTCAGGAAAATCATCTCTAATCTCGGCTATATTGGGCGATATGCAGAAGATTTCAGGATCCGTGAACGTGGATGGAAGTGTAGCATATGTTCCACAGCAAGCTTGGATCCAAAATGCCTCCGTTCGGGATAACATTACTTTTGGCCGAGAGTACTTTGAAAACATCTACAAAGAGGCCGTGAAGGCTTGTGCTTTGGAAGAGGACTTTCTCATTTTGGACGATGGGGACCAAACTGAAATTGGGGAGAAAGGTATCAATTTGTCGGGCGGTCAGAAGCAACGTGTTAGTTTGGCTCGAGCTGTTTACAACAATCGGGACATCTATCTCTTTGACGATCCTTTGTCTGCTGTGGACGCACATGTTGGAAATCATATCTTTAGGGAGGTTATGAGTAGCAGGACAGGGGTTTTGAGGAACAAGACTCGAGTCCTTGTGACCCATAGTTTGTCTGTTCTTTCTGAGGTCGATATGATCATTGTCCTGAAAAATGGGACCATCACAGAAATGGGAACCTATGAAGAACTCGTTTCTTCTCAGGGTGCGTTCTCATTCTTCCTCGCGGAATATGGAACAACCTTGCCTCAAAGTTTAAACGAAGTGGAAGGAATGGAAACCTTTAAAGAAACAATTGACACCATTATGTTAAGGAACAAAAATGGTCCAAAACATTCACCGTTGACACATCAGGATGAGCCGACGCACGTTATTTTTGGTCAAAAAAAACGCCGCAACTCTGCATCGTCTGGAACCATGGGAAATGCGCTAAGTTTAAGGAAACGGAAAGTTAGCCATGATTCAAAAACTAAACCCTCAACAGAACGTCCCAAACGAAAGCCAAAGATATCAAGGAAGAAGAAATTGATCGTGGAGGAGAAATTGGAAACTAAAGCTGTGTCAATTGAAGTTTATCTTTACTATGCCAGGGCAATCGGAATCTGTGTGACATTCTCGATCCTGCTTCTGTATGCTGTTAATCAAGGCTTCTCAGTGGGAACAAATGTATGGCTGGCCAAATGGTCAGATGATCCAAATTCAGCCATTCCAAGTATTAGAAACTTGTATTTGGGTATTTATGGGCTCTTGGGTGGGGCTTCGGCGGTGACAATCATGGTCGTCTACTTGATCGTCACGATTGGAGGCTTGAATGCCTCCACCAAACTGCATAATAACATTTTGACCACGGTTTTGGCAGCACCAATGATGTTTTTCGACACCAATCCAAAAGGACGAGTTTTGAATCGCTTTAGCAAGGATGTGGATGCCGTGGATTCGAGTGTCCCTGGTAATTTCAATGGCTTCTTCAAGTTCCTTTTCAACACGTGCGGGACTATCGCCATCATTTGCTTTACTAATCCTATTTTCATTGCCATTTTTATACCCTTGTTCGTCTGTTACTATTTCATTCAGAAGGTGTACATCGCCACTTCAAGACAAATTAAGAGGCTCAATTCCATTGCCCGATCACCAATCTACAGTCTCTTTGGCGAGACTCTCTCTGGAGTGGTTACCATTAAAGCGTTCAAGCTCCAAGGCAATTTCATCGAGCAGAATCATGGCATGGTCGACAACTTTATGGCAAGCAACTATTTGAACTTTGCCGCTAATCGATGGCTATCTATTCGATTAGAAATGTTAGGCAATGTCGTGATCCTTTTTGCTTCCTTATTTGCGGTCTTGGGCAAGGATGTCATGAATCCGGGAGTGGTTGGACTATCGCTAAGTTATGCCATGCAAATCACAGGTTCCTTGAACTTTTTGATCCGATTAGTCTCTCAGATCGAGAATAACATGGTGAGTGTGGAACGAATTCAAGAGTACGAAATGGATGTGGATCAAGAGGCAAGTTATGCTCTCGTTACTGACCCTTCGGATGGCAAAACATGGCCTCCCCAAGGAGCAATCCGATTTCAAGACTATAAAACAAGATATCGACCCGGTTTGGATCTAGTTCTTAGAGGAGTCACTTGTGATATCGAAGCGGGAGAAAAAGTTGGAATCGTGGGACGTACTGGAGCTGGGAAATCCTCGTTAACCATGGCTTTGTTCCGCATGATCGAACCTGCTTCAGGCAGCATCTTCATCGATGGCGTCAATATCTGTTCTCTAGGTTTGGGTTTCCTCCGGTCTCATATCACCATAATCCCCCAAGATCCGGTCCTATTTTCGGGGTCCATGCGTCAGAATTTGGACCCTTTTGGACATTTCTCCGACCCTGACATTTGGAAGGCCTTGGAACTGAGCCATTTGGGACAGTATGTTTCGAGTCTGAAGATGGGATTAGATCACGAGATAGCTGAAGGAGGCTCTAATCTGAGTGTGGGCCAAAAGCAATTAGTCTGTTTAGCCCGAGCTTTGTTGCGAAAAACCCAAATCTTAATCTTGGATGAGGCTACCGCAGCTGTGGATTTAGAAACCGATGACCTCATCCAGACCACGATTCGAAAAGAGTTTTCGGAGTGCACCATTCTGACCATTGCCCATCGATTGAACACCATCATGGATTGTGACAAGATCATCGTGTTGAGTAATGGAATCATTGCTGAAATGGGCTCACCCTCGATACTGTTGCAAGATCAGAGGTCCATTTTCTACGATATGGCTCAGAACGCGGGATTAGTCTACACAAGTTTCACAAAT**TAG**

>ABCC1 isoform X5

**ATG**AGCTCTTTTGAGCAATTTTGTGGAGGTCCAATTTGGGACGTGAATGCCACGTGGAATACAAACGACCCCGATTTCACCACTTGTTTTCATAAAACTGCCCTGGCTTGGACACCGTGCTTCCTTTTGTTCATCATTGTTCCATTTGAAGTCAGCAAGTATGTGAAAAGTGTCAATCGTAACATTCCGTGGAATCTGTACAACCTCACCAAACTGTTTTTGACCCTGGCATTGGTGGTCATTTCCATTGTGGAATTAATCCTCCTCTTCGTGAAAACGCCCAATGTGTATGGGGTGGATTATGTGACAACCTCACTCTTCATTGTGACCTATCTTTGCTCTTTGGTGATGCTTCTTCTATCCATGCACTTTGGGGTTCACACGTCGCCGGCTCAATTTCTCTTCTATTTCTTGTCCACGGTGACGGGTGGCATCAGCCTTCGATCTCTTATTAAACGGCAATATGAAGCCGATGAGTATGGCGATGACCCTGGTACCATTGATCGCCTTAGTATCACCTTTGGAATCCAGTACAGTTGTGTGATCTTGTTGCTTATTCTCAACCTGTTTGCTGACGCCAAACCCCTTGTTTACGATCCAGATTTTGCGGCACTCACAAAACCTTGTCCGCAAATCCATGCATCTTACTTTGCCAAGCTAGTTTACAGTTGGTCAACTCCGTTATTGTGGAAAGGGTACAGAAACCCATTGGTCCCAGAGGATTTATGGCAAGTGAACCCGAAGCTTACCTCTAAAGGGGTGGTCCCAATTTTTGATCGCCATTTTAAGGATACGATCAAAAAAGCCCAAGCCAAAGGCAAGAAATTCTCCGTCTACCCTGCCCTTTTCTACACATTTGGCCCTACTTTCTTTTGGGGAACAGCCATCAAAACGGTCAATGATGTTCTGGCAATGGTTGCTCCTCAGATTATGAGTCTCATGATTGCGTTTGTGTCAAGTGACAATAACACACCCTGGAAAGGTTACTTTTATGGAGCCGTCTTACTTATCGTCACCATGTTTCAAAGTATCATCCTCTCGCAATATTTCGAGAAAATGTTTGTCGTGGGTATGAACCTGAGAACGGCCTTGATCTCGGTCATTTACCGGAAATCCTTAAGGTTGTCTGGGGCAGCCAAGAAAGAGAGTACTGTTGGAGAAATTGTGAACCTCATGTCTGTCGATGTTCAAAGGTTTATGGACTTGATCCCTTACCTCAATATGTTGTGGTCTGCCCCGTTTCAGATAGCCTTGTGTTGTTACTTCATTTACCGAGAATTGGGTGCTGCCATGTTTTCAGGATTGGCTGTCATGATTATTGGCATTCCATTCAATGCTATAGTGGCGTCATTCAGTAGGAAATACCAACTGGCACAGATGAAGAACAAAGATGACAGAGTCAAGTTGATGAATGAGATTTTGGGAGGTGTCAAGGTCTTAAAACTTTATGGATGGGAAGAGAGTTTCATGGGCCAAATTTTGGATATTCGTGACCGAGAAATTGCGGTTTTAAAAAAGGCTGCTTGGGTTGGAGCCATGATCAATTTTGTTTGGATCAGTGTACCATTTATTGTTGCGTTGGCTTCTTTCGCAACATACATTTTTATGGGTGGAGGTCAAATATTAGATCCTTCCAAAGCTTTTGTGACCCTTACGTACCTGAACATCTTACAGAAACCCATGTCTGTCTTGCCACTTTTGATCATTGGTTTGGTTCAAGTTGGGGTCAGTTTGGATCGTATCAACAAGTTTGTGAACAACGAGGAACTTGATGCAAATGCTGTTCAACATGATGAAAGCCACAAAGATCATCCGATTTTAGTAAAGGACGGTACCTTTGCATGGGGAAAGAGTGAGCCTTCTGTTCTTCGAAACATCAATTTTAAAATTCCTAAAGGCACATTGACGGCAGTGGTTGGAACTGTTGGATCGGGAAAATCATCGTTAATATCGGCTCTTTTGGGCGAAATGGAGAAAGAAAAGGGAAGTGTCAACGTAGTGGGAAGTGTGGCTTATGTACCACAACAAGCATGGATGCAAAATGCCACCCTGAAGAACAACATCCTGTTTGGCAAGGAGCTGGATGACAAGAAATACGCTCATATTGTGGATTCATGTGCTCTGAAATCAGATATTCAGATCTTGCCTGGAGGAGACACAACGGAAATTGGCGAAAAGGGTATCAACTTGTCAGGAGGTCAGAAGCAACGTGTCAGCTTAGCCAGAGCTTGCTACAGTGAATCGGATGTGTACCTTTTGGACGACCCTCTGTCAGCTGTCGATTCCCACGTGGGAAAACACATTTTTGAGAAGGTCATCAGCCATGATGGTCTTCTAGAGGGCAAGACTAGGGTTCTGGTCACTCATGGGATAACTTATCTCCCCAAAACGGACCACATAATTGTTCTCAAGGATGGAGAAGTGAGTGAAGAAGGATCATACAAACAACTGCTGAAGAACAAAGGAGCCTTTGCAGAGTTCTTGTTGGAGTACATGACAGAAGAGACCGAAGACGAGGACGTCCTTCAGGAGATTAAAATGGAATTGGAGGGAGTTTTAGGGACCGAAACGGTCAGGAAGGAAGTGACCAGACGCCAAAGTGTCCTGAGTAAAACAAGATCCGAGAGCATTTGTAGCGCTGGATCGGTCATGTCCAATGATTCCACACAAGTTGGTATCAGAAAGCGGAAAATCACAATGGAAGAAAGTAATAAAGCAGAGGTGCAAAAGCCGGATGATAAACATGAACCAAAAATTGGCACCAACCTCATTGAGAAAGAAGTTGCAGAAACTGGCAGTGTGGGTTTACAAGTCTACAAATACTACATGATCAACATCGGTCTCTTTGGAGTAGTTTCTGGCGTGGTCATTCAAATTTTTTATCAAGTCGCTTCAATTGGAACCAATTATTGGCTGAATGTGTGGACTGGTGAAGTTTTGGGGGATTCGTCCATTCCTAAGTACCGCGATCTTTATTTGGGTGTGTATGGTGGCCTTGGATTTGCAAGTGCATTGTCAACCTTATTCCTGAGTATCACTTTGGCTGTTTCGACGTTGAGAGCTAGTAAGGAAATGCACAAATCAATGTTGGGAAGGGTCATGCAAAGTCCAATGTCGTTTTTCGATACAACTCCATTGGGACGAATTGTGAATCGCTTTGCCAAAGATATTGATGTTTGCGACAACACCCTCCCCAACAATTTGCGCCAATGGCTCAACACCTTTGCAAGTTTCTTGGGAACAATCATTCTAATTATCACCGTCATTCCCATCTTTGCTGCCGTTATCGTTCCCGTGGCAGTCATTTTCTTCTTTATTCAATCGATTTACGTAAACACATCAAGACAACTGAAGCGACTGGAGTCCATCTCCCGATCTCCTATTTACTCGCATTTTGGTGAGACGATCACAGGAGCAAGTACCATTCGAGCCTTTGGCCTTCAGAAGAGGTTCATTCAAGAATCAAATGAGAAAGTGGACATTAACCAGATCTGCTACTATCCAAGTATCATTGCTAATCGATGGCTTGCAATTCGCTTGGAAACCATCGGTAACATCATCACATTTGCCGCAGCCATCTTTGCCATCATCAATCCAGAAAATATTGACCCCAGTCAAGTGGGTCTTATCATCTCCTATGCCCTAAATGTGACCCAGGTTTTGAATTGGTTGGTGAGGATGACCTCTGACGTTGAAACCAATATCGTTGCAGTAGAACGAGTCAAAGAATACACTCTTACCATTCCTCAGGAGGCTGAATGGGAGCTTCCTAACAAACCACCCAAGGAATGGCCTCAGGAGGGCGAAGTGACCTTTGAAGGATATGGCATGAGGTACCGAGAAGGTTTGGATTTGGTCATCAAAGACATCAGTTGTCATATCAAGGGCGGCCAAACCGTTGGAATTGTCGGACGAACTGGGGCCGGAAAATCATCTTTGACCGTGGCCTTGTTCAGACTAGTAGAACCCGCTGAAGGTGGAATCAAGATCGATGGATTGGACATCAGTCAAATGGGCCTACATGATCTCCGAAAGAAACTAACCATCATTCCCCAAGACCCAGTCTTGTTCTCTGGGTCCCTCCGCATTAATCTGGATCCCTTTGGAGTTCACTCAGACAGGGAAGTTTGGCAAGTGCTTGAATTGGCCCATTTGCGAACCTTTGTGTCATCCTTGGAAGATGGCCTTGAGCATCCAGTGTCAGAGGGTGGTGAGAACCTCAGTGTTGGCCAACGGCAACTGATTTGTTTGGCTCGTGCTTTGCTTCGAAAGACCAAGGTCTTGATCCTGGACGAAGCTACAGCAGCAGTGGATCTCGAGACCGATGATCTCATCCAAAGCACGATCCGGAAAGAATTTAAAGGATGTACCGTTCTTACCATTGCCCATCGATTGAATACGATTATGGACTATGACAGAATTATAGTGCTCGACAAGGGTCGAATTGAGGAGTTCGATTCTCCTCAGGCTCTTCTTGAAAATCCCGGATCTATTTTCTACAGCATGGCCAGGGATGCCAATCTTGTT**TGA**

>ABCC1 isoform X6

**ATG**CCAGTCGTTCGCATCGGAACAGAATTCAAAATGGGGGAAACTTTTTGCCGAGATCCATTTTTCGACGTCAACACAGTTTGGAACACCACGGATCCAAACTTGACGCAATGCATGCAAGACACACTTCTCATTGGCTTGCCATGTTGTGTACTTTTGATCACATCGGTGATTTGGAATCTATATCAATACGTGATTGAATTCAGGCATGTCACATCTCTCGAAGAACGTTCATTCGGAGCTCTCTTTTTCGCAAAGCTTGTGCTAAATTTGATCCTTATCGTCAATGCCGTGGGTCAGCTTTGGAACCTAGCTAACCTAAATGGAGGATGGGACCAACTGGTTCCGTCAGAAATCGTACACTATGCGTGTCTTTTAGGAATTCTGTTCGTGGCCAATGTGCAAGTTATCATCGATAAGTGGCTCCTTTCCCACACATCTCCACCTCTGTTCCTGTTCTGGTTGGTCCTGATCTTTTGTGAAGTGGCCAAGCTCAAAATTGACATTCAAACAATCGTGGAAGGTCATAGTACCTGGGAACATTGGGTGCTTCTCCTGAGCTATTTTCCAGTGATTTTTGTACAATTGGTGCTCAGTTGCATATCTGACATTGATAAAACCACGGCACTTTTTGTCTCACCTGAAGATAGGGCGTCATTTTTATCTCTATTGACATTTTCATGGTTCGACGGATTGATTTGGAAAGGTGCTCGCCAAATTTTGACTCAAGATATTTTACCCAAGTCGCCAAGGTCTATCAATGTTGGGCATAATGTGCAAGACTTCTTGAACTTGTGGCGCCAAAAAGTTGAATTTTTGGGAGTGAATTTTGCATCATTCCAAGAGCGACCATTCATTGGCATTTGGTCCATCTTTATTCGGACACAATGGGTCAGGGTGACATTTGCATTCTTGGTGGCAATAATTCATCTGGTTCTACCATTTGTCAATCCACAGATCCTTAAACTGCTCATAAATCATGTGGATAGTGATGAAGAGGCTTGGAAGGGATATTTCTACACCGTTGTGCTACTGATCAGTGCCACAGTTTCTACCATTACATTTCACGTTTATCAACAACATTTGAGCATATCCGCGATTCAAATGCGAAGTGCCATCATTGGGGCCATCTATCGGAAGAGTCTGAAGTTGTCAAATCACGCCCGAAGACAGTTCACAGTGGGAGAAATCACCAACTATATGTCGGTGGATGCTCAGAGGGTTGTCGATACGTTTCCTTACACAATGGCATTAATCATGGCACCCTTGACCATAATAGTGGCAATGTACTTCCTCTTCTTAGAAGTGGGAATCAGTAGCTTAGCAGGGTTAGCAATTCTGCTCATTCTCATTCCCATCAATGGAATTGGTGGCAAATGGGCCGAGAGACTCCAAGTCAATCAACTCAAGGCCAAAGATAAACGCCTCAAATTGATGAATGAGGTGCTTATGGGTATGCGTGTTCTGAAGTTGTATGCTTGGGAAAACCCCTTTATGGATCGCATAAAGAAGATTCGCGACTCAGAGGTTAAGATCCTGCGTTTGTGTGCACGTTTGTGGGCTTTCATGAATTTCACCTTCACTGGCGCACCCATTTTGGTCACGATTGCAGTGTTTGCTTTGTATGTGCTGATTGACCCTAGTCATGTTCTCACGGCAGATAAGATCTTTGTGTCGCTTTCTCTTTTCAACCTCATACGAATTCCATTGGTTATGTTTCCTTTCACTCTGGTGGAAACCATCAAATTGATTGTATCTCTGAAACGCATCAACACTTACCTCAATGCTAATGAGATCAATAGTGCCAATGTGTCTCACAATATGGATTGCGAAAGTGATGCTGTGGAGCTCAAAGATTTGACTCTTACTTGGGATGAGCCAGAATCTCCCAGTTTGATGAATATTTCAATCCAAATACCTAAAGGATCCTTGGTGGCAGTGGTTGGTGAGGTTGGATCAGGCAAAAGCTCCTTATTGAGTGCCATTTTAGGGGAGATGGAGGTTGTGTCTGGATCAGCCCATGCCGTTGGATCCATCGCCTATGTTCCACAACAAGCATGGATCCAAAACATGTCCTTGAAGGACAACATCCTGTTTGACAAAGCGTTTGTCAGGACAAAATATGACGAAGTGGTGCGATGTTGTCAACTTGAATCGGATCTGGCCATTCTCAAGAATGCAGATTCCACTGAGATTGGCGAAAATGGAATCAATTTGTCTGGAGGTCAAAAGCAAAGGGTTTCTTTGGCTCGAGCAGTGTATTCAGATGCCGATTTGTACTTGTTGGACGATCCTTTGTCAGCCGTGGATGCTCATGTGGGAAAGTCGATTTTCACACAAGTCTTCGATAATCAACAAGGTCTTTTTAAGGACAAAACGCGAATTCTAGTTACTCACAACATCAGTGTCCTTTCTAAAGTGGACAATATCATCGTGATGAAAGGAGGTAGAGTGGTTGAGATTGGATCATATCAAAAGCTTTTGGCCGATGGTGCAGCATTTTCGGAGTTCTTAAACATTTATGCTCAGGAAGCCACGGAGAAGCATGAACCTGTTGACGATGTGAATCAGTTTGAAAACAGCCACGTTGAGCAAATCTCAGAACAAAAAATCAATCAAATACAAGATTCCACATTGGCAGAGAGCGGAGATCTCATCAAAGATGAGAGAGCCATGGTCGGAAGGGTCAAATGGTCCGTCTATTGGCATTACGTTAATAGTAGTCGGATATCTAGATTCTTTCTGGTGGTCTGTCTGCATGCAATAGGATCGGCTCTATTAGCATCAAGTAATTATTGGCTCTCAATTTGGGCCGATGCCAATCAAAATGACCCAGACGAGGCCAATACCCAAATAGCTTACTTCCTTGGAATTTATGGAGCCTTTGGCGTTGTTCAACTCATTCTGGAGTTTTCAAGAGAGCTCATGTACTTTCTCGGTTGTGCCACAGCCTCTCAAATCATGCATGAAAAACTGCTTTATCACGTTTTCCGATCGCCCATGTCTTTTTTCGATACCAATCCCACAGGACGAATTATCAACCGGTTTTCCTCAGACGTGGATGTGGTTGATCAAAGAATACCTCAAGGACTCTCTGATACCATGTGGTCTATATTTGACTCTTTGAGCATCGTGGTGGTCATTTGTGTATCGACACCATCGTTCGGATATGTCATACTACCTCTAGTGTTGTTGTACTACTTCATTCAAAGGATGTTCATATCAACTTCGCGTCAAGTGAAGAGACTCGAATCACTCAGCAAATCTCCTATTTATGCCAACTTCTCTGAAACTATCACAGGAACTGCATCCATTCGGGCCTATGGTGAACAAATCCGATTCAATCAAGAATCTGAAAATCGAGTACAGAAATGTGCACAATGCACGTTTTTGATTGCATCAGCAAATCGTTGGTTGGGTACGCGAGTGGAGATTCTAGGCAATCTGATCGTTTTCTTTGCAGCTATTTTTGCAATTCTTGCCAGGGATACTTTATCTCCTGGAGTGGCTGGACTTTCCATCACTTACGCCTTGAACATCATGGATAACTTAAACTGGTTGATTCGAATGCTGTGTGAGTTAGAGACTAACTCTGTGGCCTTGGAAAGGATCATTGAGTACACTCACAATGACCAAGAAGCTGATTGGGAGTCTCCAAATGATTCCAAAGAACTCTCTCAAACTTGGCCAAACAGAGGCAAGGTGGAGTTTGACTCTTTGGCTATTCGATATCGAAAGGGATTGGATCTGGTCTTGAAAGGCATATCAATGGAGATTCAAGGACAAGAGAAGATTGGCATTTGTGGTCGAACTGGAGCAGGCAAGAGCAGTTTCACTTTGGCTCTATTTCGGATCTTGGAGGCAGACTCCGGTGAAATTCGAATCGATGGACAGGATATTTCCAAGATTGGTTTGCATGCCCTTCGATCCCGGATAACTATCATTCCTCAAGATCCGGTGCTCTTCACTGGAGACTTGAGATTCAACTTGGACCCGACTGGACTGCATTCTGACTCCAGTTTGTGGTCGGCTTTGGAACATGCTCATTTGAAGTCCCACATTTCTTCGTTCAAGGATGGTTTGGATCATGAAATCAATGAAGGGGGAGAGAATTTCTCGGTTGGCCAAAGACAACTCATTTGTTTGGCTAGAGCTCTCCTTCGAAAGACTAAAATTCTCATTATGGATGAAGCCACTGCCGCTGTTGATTTGGCCACTGATGATCTCATTCAGGCCACAATTCGACGAGAATTCAAGGACTGTACAGTTATCACCATCGCTCATAGGTTGAACACTATCATGGATTGTGACCGAATAGCTGTTTTCAACCAAGGTGAAGTTGCGGAATATGCCCCTCCAAGGGATCTTTTATCAATCGACAATTCAGCTCTGTCTTTGATGGCGAAAGATGCAGGTTTGCAGGCTAAA**TAA**

>ABCC1 isoform X7

**ATG**AATCCTCGTATTATTCCAGGTGTCAATGGTTCCACTGATTTCTATCTCACTATGGACTTTTGCAAGGATCCGTTCTACAACTCGTCATTGACGTGGAACACCACCAATCCCGACATAACACAATGCATGCAAGATACCCTTCTCACGGGTGTGCCATGCTTGTTCTTTCTCCTCACTGAACCCCTTTGGATCATCTACAAGATCTACTATTATTCGATCCAAAGTTTCCCCATCTCGCGGGACTCGAGGAACACATGGGGTTGGAAATTCTTGATCAAAGTGACGCTCAACCTCGTTTTGATCACAAACCTCATCCTCCAGATCTGGAACCAGAGCCACGATGAATTCTATGGCTCTGAGGTGTTCCGCTTCATGGCCATGATCGGCACAATCGTTCTCATGCTTGTGGCAATGGGAGTGGAGAAATGGAACAGAATCCATACCTCACCTCCCATGTTCCTGTTCTGGGGCTTGTTGCTTTTGGTCAAGATACCCACTGTCAAGGATCATGTGGAACATCTGACTAACGACCCGTCTGACGTGTTATACTGGATTTTGGCTTTCACCTATTATCCAGTGTTCCTCCTCCAGATCATATTCCACTGCTGGGCTGACCACTCTAATATGGATGCCTCAGATAAGCCGCCAGAGAGTGTGGCGTCATTTCCAGCTACAATTTTCTACACCTGGATCGATCCACTCATTTGGGCTGGGTTGAAAGCTCCCTTGACATTGGATCAAGTCCCACCTCAACCGCAGTACGTCACGGTCTCTTTTGTGATCCAGGATTTCCTTAATCAATGGTCGAAGAAAGTCTCGAAGATTGAGAAAACCTTTATCAAAGATCGGGATCCAGCTGGGGAGAAGAAGGTCAAAATTCTGGATGTACTCTTTGCCACCTACGGAAAACGGTTTTTTCTGATCTTTCTCTTGGGAACCGTCAACTTCACATTGATATTTGTTGGTCCAATCATGCTCAAGCTGCTTATTCAACACGTCCAAAGTGATGAGGAAGCATGGAAAGGGTTTGCTTATGTGATTCTTATCTTCGTCAATGCTTCCTTGCAGTCCTTGCTCAACCATCAGTTCCTCCAACAGACCGTCATCAGCGCTCAACAGATTCGTGCCAGCTTAAGTTCCATGATCTATCGAAAGACCTTGAAGCTATCCAACTTGGCGAGGAAAAAATTCACAATGGGAGAGATCACTAACTTCATCTCCACTGACATCCAAACTTTGGGCATGACCATACCTTACATGAACAACGTGTGGGTGGCACCCATGAAGATCATCATAGCGATGTTCTTCTTGTATGAGGAGCTCAAGTCTGTGGCTTTTATCGGAGTTGCAATCATGGCTGTTCTGCTTCCATTGAATTATTGGAGTTCCGTGATCATTAAGAAAATGCGCAAAAATCAGATGGAAGCCAAGGACGGTCGAATCAAGCTGATGAACGAAGTCTTGAAGGGCATGAGAGTCTTGAAGTTGTACGCTTGGGAGATCCCATTCATGAGCCGGATCAACGCCATTCGACTCACCGAGGTAAAACTAATGAAGTCTTTGGCCAAAGTTTATGCCATCTCAAATTTCACTTTAAATTCGGGACCCACTCTGATCACAATTGCTTGTTTTGGATTGTACACCGTGTTCCAACCAGGAGAGATCCTCACTGCTGATAAGATATTCGTCTCCATTGCCTTGTTTAACATCATTCGCATGTCCATGGTCATGTTACCTTGGGCCATTGTGGCTCTTTTGAAGGCTTTTGTGTCCTTGAAGCGCATTGCTGATTTCCTGAATGCCAACGAATTGGACCCTGAAGCTGTTGGCAAAGAGACGGAGCATGAGGAAAATGCCATTGAAATAACGAAAGGATCGTTCACTTGGACTGAAGAAGAAGAGCAGACTACACTTAAGGACATCAATGTTCAGATTCGCAAAGGCTCCTTAGTTGCCGTCGTGGGTGTGGTGGGAGCGGGCAAGAGCTCTCTTTTGAGCGCCATTTTGGGTGAGATGGAGAAAACCGATGGACAAATTAATCAAAGCGGCACTTTGGCATATGTTCCACAACAAGCGTGGATCCAGAACATGACCCTCAAGAACAACATTCTGTTTGAAAAACCTTTCGAAACAGGCAGATACAAGAGGACACTGGAAGCTTGCGCTCTCAAGGATGACCTGGATATTCTTATCAGCGGAGATGAGACCGAGATCGGTGAGAACGGGATCAACCTCTCCGGAGGCCAAAAGCAACGAGTCTCGGTGGCTCGAGCTGTTTATTCCGATTCGGATGTGTATCTTCTGGACGATCCACTCTCTGCAGTTGATGCTCACGTGGGCAAACACCTCTTTGATAAGGTTTTCAGCTCCAAGAGCGGAATTCTGAAAGATCGAACCCGAGTTCTAGTGACCCACAATGTGAGCTTTTTGGACCAAATGGATCTGATTTTGGTGATGCGAAATGGTACCATAGCTGAGCGCGGATCCTACTCACAACTGAAGGAGAGCAAAGGAGCTTTCAGCGAATTTTTAGAGCAATACGCCAATCAAGCAACAAAAGAGGAAGTTACTGAGGATCATGAAAAGAACAAACACCTTCAAGCGAAACCTATCAAAGCCCGACAAATTCCCCCAAGGACAAGACGAATGTCCCAGACTCTAAGTTGCTCACCCGCTTCGCCATTTATTAAATATTTGAGCACATCTGCAGATGCACCTCTTCCGGATGACATTTTGGCCGAAGAGGAAGAAGCAATTCTGGAAGAAACCATTGAAGAATCCGCTGTCTCATCACGACGTGGATCCAAGGTCAATGATCCCTTGGCAGGTCGTTTGGTCGATGATGAAGAGGCTATGATTGGCAAGGTCAATTGGAAGGTCTACTTGAGCTACATGAAAGATCTGGGAAATATCATCAGCGTTATCTCGTTTCTCCTCTACATGGTTGGCCAAGGCTTGGAATCCGGATCAAGCTATTGGTTGTCCTTTTGGGCCGATTCGAACGGAGCCGACCCTGCCACAGCTAATGACAAAACGCCCTTTTATCTCGGCGTCTACTCGGGCATTGGTGCTCTTCAAATCATTATCATGATTGTTCGCGAGATTGTCCTGTTCATTGCTTGCGCTGATGCTTCGAACCTTATTCATGCCCGATTGTTGTCCAAAGTGGTGAGCAGCCCGATGTCATTCTTCGATACCAATCCCATTGGGCGGATCGTCAACCGATTTTCAGCAGATATTGGCACAGTTGATGACGGGATTCCATTTCAAATATCGCTACTCTTCGAGTGCTTTACCAGTATCGCTACCGTTTTAGCCCTGATCTGCTACTCTCTTCCAATCTTTATGACTGTCATTTTACCTCTGGCATTCATGTACTATGGAATTCAACGATGTTACATCTCCACTACCCGTCAATTGCAAAGAATCGAATCCATTGTCATCTCACCCATCTTTGCTCATTTCGGTGAGTCCTTAAATGGAGTCCAATCCATCCGAGCTTACAAACAAGAAGAGCGATTCATCCTTGAATCGGAAAAGCGTGTGGATAAGGGCGTTCAACTGCACTACATGTGCGTCATGATGAATCGTTGGCTGGGAGTTCGGATCGAGTTCTTGGCTAACTTGGTCGTTTTCTTTGTGGCCTTGTTTGCAGTTTTGTCCCGAGGAACCATCTCACCTGGTCTCACGGGTTTGGCCATCACCTTTTCCTTGAATGTCACCGGCATGCTCAATTGGTTGATTCGAGCGGTTTGCGACATGGAAACTGATGCAGTGGCCTTGGAGCGGATCCTTGAGTACTCTGAAAATCCTCAAGAAGCAGATTGGTCTCTGCCTGAAGACGATAAGTTTTGCGATTTCCCCATGGAGGGCCAGATCGAGTTTTCCAAATATGAAACCAGATACCGAGAGGGATTGGATCTCGTGCTCAAAGACGTTTCCATGCGAATTCAGCCCAAGGAAAAGATTGGCATTTGTGGACGCACTGGTGCCGGAAAAAGCAGCCTCACACTGGCGCTCTTCAGAATCATTGAACCCGTCTCGGGCAAGATTCTCGTGGACGGAATTGATGTGAATACCCTTGGCCTTCACAAGCTCAGATCTGGCCTCACCATCATTCCGCAAGATCCCGTTCTTTTCACGGGTAATGTCCGATTCAATTTGGACCCCACCGAAGAAAACACGGATACTCAAATATGGCAAGCTTTGGAGCATGCCAATCTCAAGGCTCACATTAGTTCATTGACCAATGGACTAGACCACGAAGTATCAGAGGGTGGAGACAACTTTTCCGTGGGTCAACGCCAACTTCTGTGTTTGGCCCGGGCATTACTTCGCAAGACAAAGATTTTGATCCTTGACGAGGCCACCGCTGCCGTCGACCTCGAGACCGATGATCTCATTCAAGCCACAATCCGCAAGGAATTCGCTGATTGCACCGTTCTCACAATTGCTCACCGACTCAATACGATTTTGGATTCCGACCGAGTTGCCGTTTTTAGCGAAGGCAAATTGGTTGAACTAGATTCTCCACAAAGCCTTATGGACAAGCATGATTCTGTTTTTAGAGGTTTGGCCAAAAACGCAGGCCTTGTT**TAA**

>ABCC1 isoform X8

**ATG**TTGCAACTAGGCAATATGTCTTTTTGTACTGATCGGTTGTGGGATCCGGATATCACTTGGAACACTAATGATCCAGATTTGACAGAGTGCTTCCGGACCGTGATATTGTCCCCTTTACCAACTTTTGTCCTGATTGTCACTCTTCCATTTTGGCTGAGACAATTTCATCAAGCTAAAAAATCCATCTCTGTTTTTGGCACACTCACCAAAATATTTTGGATGAAATTGGGACTGACGGTGCTTTTGTCCATAAATGTGTTTTGTGATTTTGGAACACACCTCATCACTGACGCTAATTTGTTTCATTCTGACTTTCTGAGAATGATCATTTTGGAACTAGGGCTCATACTAATGGGGGGATTAACTTTGATCAGCCAAAGATATCATCTACAGACATCTGGAAGCCTATGGATATTTTGGTTTCTTACCTTGGTGGCTGAATTACCCACTTTTAAATATGAAGTTGATTACCTCACGATTAATTTCCAGCCGTTTAGAGCGGTTTTGGTGGCGACTTTTCTGCCTTTGGTTTTTATTCAAGTGATTTTGTACAGCATAACAGAAACGCAAGTGATTGGTTGTCCCGAAAGTCGAGCTTCGTTTATTTCTTATCTAACCATGAGTTGGCTTAATGGTTTGATACGTCAAGGCTTCCGAGCTCCTTTAACACAAGAGCAATTGCCCAAAGTGGATCCGGGATTAGATGCCAGTATCAATGAACAAGATTTCCTAAATAATTGGAACAAGGAAGTTGCTCGAAGTGGCGAAGTGAATATCTGGTGGGTTTTACTCCGCACCCATTGGAATGCTCTAGCCCTAGCAATGTCTTTGTACTTGATTCATCATGGCTTGCATTTTGTCAATCCCTTGATGCTCAAGCTCATCTTGAATCACTTGCAAACTCAGGACGAAGAGACGTGGAAAGGTGTCCTTTTTGCGTCAGTTACCTTTATTACAAGCATCATGTTTACTGTGCTATTCAATATTCCAAACCAACTTATGACCGGTTTAGCCGTGAAATTACGGAATGGTCTCGTGACAGCGATATACAAGAAGTCCCTAAAACTGTCAAATGAGGCCAGGCAAGAATTTTCATCGGGGGAGATCATTAACTTCTCATCAGTGGATAGTCAACTCATCTTGGAATGTGTTCCCTACGTTGAGCATCTTATAGCCGATCCATTACTCGTGATTGTTACAATGACATTTCTTTTCGTGGAATTGGGCCCGGCGGCGTTAGCTGGAGTCGCTTTTTTGTTCCTTCTGGTGCCATTTAACACCTATGGCAATAGAAAAATAGAAAAGTGCCAAGATATCTTGCTCAAGAACAAGGACGAGAGGATGAAAATCACCTCCGAGACTTTGAGCGGAATCCAGTTAATCAAGATGTATTGTTGGGAAATTCCTTTCATGGACAAAATCAAGGGATACCGAAACATTGAAGTTGATACCTTGCAAAGCACGGCCAAGCTTTACGCCTTATCCAATTGCACGTTCAGTAGCAGTCCAGTGTTTGCGTCTTTATTGATCTTTGTCTTGTATGTGGCCTTAGATCCTACAAACCACGTTCTCAACCCTGAAAAAATATTCGTGTCGATATCACTACTGAGCATTCTTAGAATTCCTCTGGAGTTGTTTCCGATTGTTCTCTTTGATACAATTCGTATTGGAGTGTCAATCCGAAGGATTGGAAAGTTCCTGAACGCAAAAGAGTTGGAAGAAGACGCTGTCGGTTGTCAAACTGAAGATCCCCAAAACGCCATCGAGATTAAAAATGGCTCTTTTTCATGGAACAAACATGGCGATTTTCTTCTTAATGTCGGGAACATATCCATCTCTAAAGGATCTCTCGTCGCAATTGTTGGAAAAGTTGGCTGTGGAAAGAGTTCCTTGCTCTCTTGTCTTCTCGGAGATATGGTGAAGAAACAAGGTTCAGTTAACATCGACGGTCAAGTAGCCTACATCCCCCAAGAGGGCTGGATTTTAAATACTACACTCAAAGAAAACATTCTTTTTGGAAAGCCCTTTCAAAGTCGAAACTATGAGCGAGTGATCGCTGCTAGCGCTTTGGAGCAAGATCTGATTACCATGGTTAATGGGGACCAAACAGAAATTGGCGAGAATGGCGTCAACCTATCTGGGGGTCAAAAGCAACGGGTATCAATCGGAAGAGCCGTTTTTGCCAATCGTGATGTTTACCTTTTGGATGATCCTTTATCAGCCGTGGACTCTCACGTTGGCGAGCACATATTTCATAACGTGATATCCTCCGCTCATGGACTTTTGAGAGACAAGACTAGGATCTTGGTCACCAATCAGATTCACTTTTTGGAAAAAATGGATCAAATCATCGTCATGGACCAAGGGGAAGTCAAAGAGCAAGGCACTTATCAAGAACTCGTGGCTAGTGGGAATCAATTTTCGGCCCTCATCCGCGACTTTGGTCATAAAAACACGAAAGAAAAGGTTCGGCAACGGCAAAGATTGCTCTCTCATATGTCAAATGGAGGTGGTGAAGACTTTCACGTGGAGGATGTCAATTCAAGGCAAGATACTGGAGGTAGGCTGATTCGCGACGAGGAAGCATTCCATGGCCAAGTGAGATGGTCCGTTTACTTGGATTACATGCGGATCATTGGACGGCTTCCTTCCACCAGCATACTGATTATGTTCGTTCTTGGCCAAGGACTACATATTAGCAGTACTGCTTGGCTTTCGTATTGGGCAGATATGAATGAGGAGTCCAATCATGCCCCGATTCTATTTCTGGGCGTGTACGGAGCCATTGGCATAGGCGAACTAATGGTTTGCTTTGGAAGGCAATACTCTCTCTACCGGGCATGTGCAAAGGCCTCAAGAGCCCTTCATCATAAGCTCTTGTTTCACATTATCAGAGCTCCAATGCACTTTTTTGACACCACGCCTTTAGGACGGATTTTAAACCGCTTCTCATCCGACCTCGACGTGGCCGATGAGACACTACCTCAAGAGATCACCGATTTCCTGTGGTGTTTCATGGACATTTCGTTCACCCTCATCCTGATTGCCATAGCAACTCCTTGGTTCATTATTGTTGCGATCGGCCTATTTGTTCTGTTTTGCTCCATTATAATTTATTACATCCGAACCTCTCGCCAACTGAAGCGGTTGGAATCCATCAGCAAGTCGCCGATCTTGAGCCACATCTCGGAGACTTTAAGAGGCGTAACCAGTTTGCGGGCTTATCATTGTGCAGACCAGTTCATTAGCAGGTGTCATGACAAGCTCAATGAAAATGTAGCTTGTCATTATCTCAATGCATGTTCAGTTTTCTGGCTTGGAGCCCGGACAGAATTCATCGGGGCCCTCATAATCTTCAGTGTGGCTTTATTAGTCGTGCTTGACCGAGGCTCAATAAGTGCTGGACTAGCTGGAATGGTCTTGACCTATTCTTTTGAGCTCTTGGAGGCATTCTCTTGGATGGTGAACATGGCTTGCAATCTTGAGGGCAATTCGGTGTGCTTGGAGCGGATTATGGAGTATTATCATGTGGATCAGGAGGACCAGTGGACCAAACCTGATCAAACTGCCGAATGCATGGGTGCAATCGAATTCCAAGATTATAGCACCCGTTATCGACAAGGTTTGGACTTGTGCTTACAAAATATCAGCCTCGAGATCCATCCACAAGAAAAATTTGGCGTGTGTGGAAGAACAGGTGCGGGAAAATCAAGTTTCACCAAAGCTCTCTTCCGTATTGTCGAGCCCGTTGAAGGCAAGATACTCATGGATGGCGTCGATATTACTGAGAAAGGGCTTCATGACCTTCGAAGTCAACTCACGATTATTCCTCAAGATCCAATTCTGTTTTCTGGGACATTGAGGTTCAACTTGGATCCCCTAGCTCAACATTCCGATCAAGAATTGTGGAGTGCATTGGAGCATTCTCATTTGGTCGAATATGTCAACACTTTGAAAGACGGACTTGAGCATCAAGTGCAGGAAAATGGAGATAATTTCTCAGTTGGTCAACGTCAATTAGTATGCTTGGCACGAGCTTTGCTCCGCAACACCAAGATCTTGATATTAGACGAAGCTACGGCTTCAGTAGACTTGGAAACGGATGCCCTGATCCAGAAGACTATTCGAGAGAAACTGAACCAAAGCACTATTGTGACAATAGCTCATCGAATCAATACGATATTAGACTCGGATCGAATTCTGGTTCTCTCCGATGGATCTGTGGCTGAGTTGGATACCCCACATTCTTTACGGAATCAGCCAAACTCTGTCTTCAAGTCCATGCTGAATGCAGCCAATCAATCG**TGA**

>ABCC1 isoform X9

**ATG**TCCGCCGAGGATGGGTCCATCACGGGCAATGTCAGTCAGATGGAGAATGTCGATCCGTTCTTGTGGAACGTGGATGAGGACGATGAGGCTCACGGGGTCATTTATTGGCCGGAATTGTGCGGTCCGGATCGACCGTTTTGGAACACGTCGCAGTCATGGCACACGGACACGCCCAGTTTGAGCGCTTGTTTCCGCACGGCCGTGTGGAGCGTTCCGCCCTTTGTGTACTTTTGGCTGCTCTTACCCTTCTATATTCGTTATGTACGGCGAGCCAAGGGGCGGGACATTCCTCTGTCCACCTTGGCCTTGGTCAAATTCATCGCGCTTCTGGCCTTAACGGGTGTGGCGGCCATGGACACGGCCTATTTCGCTCTGGGCGCCAATGTGGCTTTGGTGGATGTAATGGAGCCGGTTTTGCGATTGGCGTGTTTTGGGCCTCTCTTGGGCCTGCTCTTTCTGGAGCGACGCCACGGGATTCGCGTGTCCGGTTTTCAATGGTGCTTCTGGACTCTGTTCCTTCTCACATCGGGTCTGGTGTTCTACAGCCATCTGGAGCGTTTGTGGACCGAGACCCTCTCCCCAGGATTCTTTCCGTCCGTCACGTTTCTGTTGTCCGTGTTCTTGTTCGTCGTGTCTTTTGCCCTCCATTTCTTCGTTGAGCCCGAGCCCGCTTATCGAGGACCGCTTCAAATCGTCCAAGCTCGGCCTTCACCGCAGATCACGGCCAGCTTCCCGTCACAATTGGCCTTCTCTTGGTTTACGGGTTTGGCTTGGTCGGGTTTCAAGCGATCCTTGACCTTTGACGACCTCTGGGATTTGAACCCCATGCTGATGAGTCGCTTCGTGTTTCCCAGGTTCAATCGGTTTTGGGAACGCACCGTTAAGCAAATCCAATACGTCCATGCTAATGGACACCAAGCGACCAATGGTCACGGATTGGCCGATGCCTCGTTTAACCGAACCAACGAGGACGTGGATTTCAAGTCCACCAAAAAGAGCTCTTCCCATGCGGATCTATCGAGACTCAGCGTGTTCCCAGCCATGGCCAAGGCCTTCGGACCGGCCTTCTTGTACGGATCCTTGCTAAAGTGTGTCCACGATATCATGATTTTCATCTCGCCGTTTTTGTTAAAATTGATCATCGCCTTCACGGCCGACCCCGCAGAACCCTTATGGAAAGGCATCTTTTTCGCCGTTCTGATTTTTGTCATCTCGTGCGTTCAAACGTTGTCACTATCGCAGTACTTTTACAAGATGTACGTCATTGGTTTGTGGCTCAGAACGTCTTTGATATCCGCCATCTATCGCAAGAGTCTCCGCGTGTCCACCACGGCAAAGAAAGAAATTACCACCGGAGAAGTGGTCAATTTGATGTCCGTCGATGTCCAGCGAATTATGGAAATGACGCCTTACATCAACATGCTTTGGAGTGCTCCCCTACAAATTGCGGGTGCCATCTTCTTCTTATGGCAACAATTGGGCCCGAGCGTCTTGGCTGGTCTGTTGGTCATGATCTTATTGGTGCCTTTGAATGGCATGATCGCGGCCAAATCACGTCAATACCAAATCCTTCAAATGAAACACAAAGATCAACGAGTCAAGGTCATGAACGAGATTCTCCAGGGAATGAAGATCCTCAAGCTCTACGCGTGGGAACCGAGCTTTGAGAAGTCGGTCAATGATATTCGAGCACAAGAAATCAGTATCCTAACGCGTATGGCTTATCTACAATCCGGAAGCTCATTCATTTGGACTTGCGCACCCTATGTCGTGTCCCTAGTTACTTTCGCCACCTATGTGCTCGTCGATGAGGCTAATGTGCTGACGGCTGAAAGAGCTTTCGTTTCCTTGGCTTTATTCAACATCTTGCGCTTCCCTTTGAGCATGTTGCCCATGATGATCACGGGTTTGGTGCAGGCCAGTGTCTCAATCAAAAGAATCAACAACTACATGAGGGCAACTGAGTTGGAAGAGAAGAACGTCACCAAAGACACCAGTCAAGCAGGCAGAGAAGACAAATGCGCCGTAAAACTGAACAAGGCCTCGTTCACATGGAATGAAGAAGCCTCTGAGCCCACATTGAAAGAGATCGACGTCGAAATAAAAAAAGGATCCCTTGTCGCTATCGTGGGTACGGTGGGCTCTGGAAAATCTTCGCTTCTGGCAGCCATGTTAGGAGAAATCGACAAAACGGAAGGACAAGCTTTTATCAATGGTCGAGTGGCGTACGTGGCTCAACAGGCATGGATTCAAAACGAGACCATTCAAAATAACATTCTCTTCAATGAATCTCTGGATGACAAACGTTACCAAAAGGTTCTCGAGGCGTGTGCACTTCAAGCCGATTTAAGCATTCTGCCAGGTGGCGATCAAACCGAGATCGGTGAGAAAGGAATCAATCTCTCTGGTGGACAGAAACATCGGGTCAGTTTGGCCCGAGCCACTTATTGTAATGCTGACATCTATCTTATGGACGACCCATTGGCCGCTGTGGATTCCCATGTTGGCAAGCACATCTTCCAGGAGGTCATTGGACCAGAGGGACTCTTGGCCCAGAAAACGAGAGTCTTAGTGACCCACAGCATTGCCTTCCTGCCGAAAATGGACAACATCATCGTTTTGAAAGAAGGCAAAATTACGGAGAGTGGTAGTTACGACGAGCTTTTGGCTCGAAAAGGTGCCTTTGCCGAGTTTCTCGTTCAATTTTTGACGGAGGAAGAACAGACCGAGTCGGATGACGATCAAGTGCGCGAATTGAAACACACTTTGGAAGAGTGCATGGGCGCTGAAGAGCTCAACCGCCATCTCGAGCGTGCCCGTAAAGAGAGTGAATGCAAAGAGAGCTCCATCGAGCGATCCATGTCCGTGAGTAGTCGCAAATCCAGCGAAGGTCATCCGGTCAGATCGCCCAAATCTCGTCAAGGCTCGGACGCTCATAGCGATGCGGCTGCTGACAAGCAAGAACCAAGTCCAAAGAAACTTTTGACCCCGGAAAGAGACGGCACCCTCGAGTCTGTCACTAAAGACAAACCGACGCCTCCTCAAGCATCCAAAAATAAGCAATACCAAGAGGAAAAGACCGAAAGCGGCAAGGTTCAAATGGCGGTCTACATCTACTACGTCAAGAACATGGGCGTACTCTCGTTTGCTACCTCTGTGATATCGTTTTCAATCTATCAGATCTGCTCGGCAGCCTCGAGTATTTGGCTGTCCAAGTGGTCAGACGAGTCCTCTCGGGCAAACCAAACGGACACCCTCTCCCGAGATGTGTATTTGGGCGTGTATGGGGCGTTTGGAGTTGGTCAGGGTCTCACGGCAGTGTTTGGATCGTTGTTCTTGTACCTGGCCACTCTAAATGGTGCTCAACGATTGCACAATTTGGTGCTTGGCAATGTGTTAAAATCGCCCATGTCATTCTTTGATACCACTCCGCAAGGCCGAATTTTGAATCGATTTGGCAAAGACGTCGATGTCTTGGACACAACCATGCCAATGATTTTGAGAGGCTGGATCACCTGTCTGCTGGCCGTACTCTCGACGTTTGCCATTATTTCGTACACCACACCCATGTTCTTGCTACCAATTACCGTGGTTCTGGGTGCCTACTACATCGTTCAGAGGATTTATGTGGCTACTTCGCGCGAGTTGAAGCGGCTCGAATCGGTGAGTCGCTCACCGATCTACTCCCATTTTGGCGAGACCGTCAGTGGTGTTCAGACCATTAGAGCATACAACCTCCAAGAGCACTTCATTCGAGAATCCGAGAACCGTGTGGACTATAATCAAAGAGCTGGATACCCTAGCATCGTGTCTAATCGCTGGTTGGCTGTTCGGTTGGAGGTCGTTGGTAATATCATCATCTTTTGCTCCGCTTTATTTGCCGTCCTGGGCCGTGAGAATCTCTCACCCGGATTGGTGGGTTTGTCCGTGTCCTACGCCCTCTCTGTCACTCAAACTCTCAATTGGTTGGTACGAATGACCTCCGAGGTCGAGACCAATATTGTGGCAGCCGAAAGGCTGAAAGAGTACGCCGATTCCCCCACCGAAGCTGCCTGGACCAATCCTGACCACACACCCGAGGAATCGTGGCCCAATAACGGCTCGATTCACTTTGAGGATTACTGTACCCGCTATCGAGAAGGACTTCCATTGGTCCTCAAAGACCTCACTTTCTCCGTGAAGGGCGGAGAACGTGTCGGCATCGTGGGTCGAACTGGAGCTGGAAAGAGCTCGCTGACCTTAGCCTTGTTCCGATTGATTGAGAAAGCAAGTGGACGAATTCTTATCGATAACTTGGACATCTCAAACTTGGGACTCCACGATCTCCGCTCCAAGATAACCATCATCCCGCAAGATCCCGTGCTCTTCTCGGGAACTTTGCGAATGAATCTCGATCCTTTTAATCACCACAACGACGAAGAGATCTGGCAAGCATTGACTCATTCTAATCTCAAGACTTTTGTCAGCGACCAATTGACCGAAGGTCTGGACCACGTGATCTCCGAAGGAGGAGGCAATCTCTCTGTAGGTCAATGTCAACTCATTTGCTTGGCCCGTGCTCTTTTGCGGAAGACCAAGATTCTCGTCCTGGACGAGGCCACCGCCGCTATTGACATGGAGACCGACGAAATCATTCAAAAGACGATTCGAACCCAGTTCAATACTTGTACCATTATTACCATTGCCCACCGATTGAACACCATCATGGACTACGACAAGATCTTGGTGCTGAAGAATGGAAGTCGAGAGGAATTTGATTCTCCGCAAACACTCCTGAATTCCAAAGAATCCATCTTTTATAGTATGTGCAAAGAGGCTGGGCTGACGCAA**TAG**

>ABCC1 isoform X10

**ATG**AGAACAAGTTCAGTCTCAATTGAACTCAACGAGAGTCCAAACGAACCTACAATGGCGTCGTTTTGCGATGATCCTCTTTGGGATCTGAATGTGACTTGGTACACAGATAATCCCGATTTCACCACTTGTTTCCATCAAACCGTTCTCGTCTACGTTCCCTTGGGTCTTTTGCTCCTTTTCCTCCCCGTGGAATTCATTTATATTCGAAAATCCAAAGATCGAAACATTCCATTTACACTCCTGAATCTGACCAAGATTACATTGAATCTGATCCTCATCGTTCTACCCATTATTGACCTCTTCTACGTCATCAACCATGACGCTGCTTGGGTTCATTTGGTGGCTTCCATCGTTCGATTGATCACTTTCGTGATCACATTGGTGGTATTGTTCGTTTGCTTGCAACGCGGTCTGGTCACTTCGGGGGTATTGTTCTACCTTTGGACCTCACTTCTTGTGGCTGATGGTCTGACCTTCAGGTCTGTGATCATGTCTGGATTGGCCAAGGGCCCTAATGCAATGAGTCCTTTCATCACCGCTTTGATACAATACCCAATCATCGTGGCCATGTTCTTCTTGAATTGTTGGGCCGATGCCAGACCCAAACAGATCAACTTTGATGATAATGTGCCCAACATTGTTCCCGAAAGGTACTCTTCGCACATCTCCAAGATGCTCTTTGCGTGGGTGGATCCACTCATGGTGAAAGGTTTCAAGAAAACCATGTTGAAGCAGTCTGAGCTTTGGTCATTGAAGGCAAACAACAGATGTTCAGGGGTCATCCCCAGTTGGGACAAATCTTGGGAGAATCAAGTGAAAAATGCCAATGGCAGGATGATCTCCATCCTTCCTACCCTGATTCGAGCCTTTGGTCCAGCCTATTTCTTGTCTTCCATTCTCATGTTTGTCTTGAGCATTTTGCAATTTGCTAGCCCTCAAATTGTCGACTTGCTTATTGCATTTGTGAGCTCGGATGATCCAAACTGGAAGGGCTATTTCTATACGTTTCTAATTGTGGGTTCCACCTTGACCGTCTCAGTTTTGAACTCGCAGAGTTTCTATCAAGAATACTTGGTTGGACTTCGAGTTCGAACTGCCTTAATATCTGCGATCTACAGAAAATCATTGAGATTATCGAATTCGGCTCGAAAAGAAATGACTGTGGGAGAAACTACAAACCTTATGCAGATTGATACCCAGAAGTTTATGGATCTGACCCTGTACTTAAATTTGATTTGGTCGAGTCCGCTGCAAATTGGATTGTCTCTTTACTTCCTTTGGGGAACTCTAGGCCCTTCATCTCTCGCTGGTTTGGCGGTGATGATTTTGTTACTCCCGTTGAATGCAGTCATAGCCTCAAGGCTGAAGAAACTGCAGATCTCTCAAATGAGAAGCAAAGACAAGCGAACCAAACTCATGGACGAAATCTTAAACGGAGTCAAAATTTTGAAGCTCTATGCTTGGGAGACGTCATTTCAAAAGCAAATCTTAGGCATTCGAGATCAAGAAGTTAAAGCGCTGAAGGTGATTGCATACTGGAACGCAGGGATGACCTTTCTCTGGACGTGTGCGCCAGTGGTTATCGCTTTGGCATCGTTTGGAACCTTTGTCCTCGTTGATCCAAACAATGTTTTGGATGCAAACACAGCTTTCGTCTCGTTGACGCTCTTCAACCTGCTCAGAGTACCCATGAATTTACTACCCATGCTTATGGTTTATGTGGTTCAATGTCAGGTGTCACTGAAACGAGTCAATCGGTTCATGAACGCCGAAGAACTTGATCCCAACACCGTTTCCCATAGCAAACACGTCAAAGATGCCATTCACATTGATAATGCATCGTTCACTTGGGATCGAAACCAAGAACCCACGGTGAAGAACATTACTCTGAAGGTCAAAGAGGGATCTCTGGTAGCAATTGTGGGACAAGTTGGAGCGGGAAAATCTTCGCTGATTTCAGCCATGATTGGAGAAATGGAGCGAACCCAAGGGAGTATCAACACCAAAGGCAAGATTTCCTACGTGCCTCAACAAGCGTGGATCCAAAATGGAACAGTCCAGTACAACATTGCATTTGGAAACAAGTACAGCAAGAGTCTCTACAACAAGGTCATTGATGCTTGCGCTCTGCGTCCTGATCTGGAAATGCTTCCTGGAGGAGATCAGACAGAGATCGGCGAGAAAGGCATCAACTTGTCTGGAGGTCAGAAGCAACGTGTGAGCATGGCTCGTGCCACCTACAATGGAGGCCAACTATTTTTCTTGGACGATCCTTTGTCTGCGGTTGATTCGCACGTGGGAGCTCATATCTTCAAGCACGTGATCGGACCTGAGGGCATGCTGAAAAACAAGACTAGAATCCTGTCTACTCATAGTGCCAAATATCTGCCTCAATGTGACAACATTGTCGTGATGAAGAACGGAGCGATCAATGAGATTGGCACCTACAGGGAACTCCTACGAGACGATGGCGAGTTTGCCGAGTTTCTCATTCAATACTTGACGGAAGAAAGTGACAAAGACGAAGAGGCCGATGATGAGGTCTTGGAGAATCTTAAGCAAGAGTTGGAGGTTGTCATGGGAAAGGAGAAGTTCCAACGACAACTGAGCTATGCCCAGTCTCAGAAATCCGAGAGTAATCCATCCATGAAGAGTGGAATGACCGGGATCAGGAAAGAGCGAACCAGGCGAACGCGGAAAGAGTCGGTTATGACTGACATCGAGAGCATGAAAGAACACATCATCAAACCAGGCACCAATTTGATTGAAGATGAGAAGGCCGCCATCGGTGGAGTCAAATGGACCGTCTACGGATACTACTCGAGGTCAGTGGGCCCACTCATGCTGGCCATTTCACTTCTCTTGTATGGAGGATATCAAGGGTTCTCAGTTGGGAGCAGCATTTGGTTATCGAGGTGGTCAACAGACCCGTTGGCATCAACGGACACAACTGTCAGGGACATGTACCTAGGGGTCTACGGAGTTTTTGGGCTTTTGCAGGCTGCACTAATCATGGGTGCTACGGTTTTTCTCTCTATTGCTTGCTTAAATGCTGCCATCAAGCTCCACGCCACCATGCTGCAACGAATTCTCAGGTCACCAATGTCGTTCTTTGACACCACGCCTTTGGGCCGGATCTTGAATCGGTTTTCAAAAGACATCGATATTGTCGACATCACCATTCCCCAAATCCTTCGCTCGTTAATGGTTCAAATTTTGACAGTACTGGGGACCATTTTTGTGATCTGCTTCGCCAATCCCCTGTTCATAGCCGTGATCATACCAGTTTTCATTATCTACTACTTTGTTCAGAAGTTTTATGTAGCCACTGCTCGACAGGTGAAGCGCATGGAATCCATTACTCGATCCCCTATCTACAGTCATTTCGGAGAGACTATCAGCGGAGCCTCCACAATCCGAGCATATGGAGTCAAAGACAAATTTATCGAGGAGAATGAGTCCAAAATTGATATCAACCAGCAATGTTACTACCCGACGTTTGTCTCGAATCGATGGCTGTCCATTAGATTAGAATGTATCGGAAATTTGATCATCCTCTTTGCCGCCTTGTTCACCGTCATTTCTCGGGACAAACTTGATCCTGGCTTGGTGGGATTAACTCTAAGCTATGCGTTAACCATTACCAACTCCATGAGCTTTCTGGTTTCCATGACATCGGAAATTGAGACCAATATGGTTGGTGTTGAGCGCATTATGGAGTATCAAGAGATAGCCGAAGAGGCTCCACTGGAAATCCCAGAGCAAGATCCGCCAGCTGATTGGCCATTGTTTGGAGTGGTCAAGTTCGACAACTATCAAACCCGGTATCGTGAGGGACTGGACCTTGTTTTGAAAGGAATCGATTGTCATATCCAGAGTGGTGAAAAGATCGGAATTGTCGGTCGAACTGGAGCCGGTAAATCCAGTTTAACCCTGGCCCTGTTCAGAATCATTGAAGGGGCTGGAGGAGCTATTCTGATTGATGACCAAAACATTGCTCTCATGGGGCTTCACCGACTTCGATCCCGATTGACCATCATTCCACAAGATCCCGTCCTCTTCTCGGGGTCACTGCGCATGAATTTGGATCCCTTTGATCAATATAGCGACAAAGAGATTTGGCAAGCCTTAGAACATGCCCATTTGAAGGCCTATGTTTCTAACTTACCCAAGACACTTCTGTTTGAAGTGAACGAAGGCGGCGAGAACTTGAGCGTAGGTCAAAGACAATTGGTGTGTCTGGCTCGAGCTCTCTTGAGAAAGACCAAGGTTCTGATCTTGGACGAAGCCACGGCTGCCGTGGACTTGGAAACGGACGACCTTATTCAGGCCACGATTAGATCCGAATTTTCCGATTGCACCGTGCTGACCATTGCTCATCGATTGAACACCATCATGGACAGTTCCAAGGTCATAGTTTTGGATGCTGGCCGAGTGATCGAGTTTGATAGTCCGGCCAACTTATTGAAAAACACGGCCAGCGTCTTTTACGGAATGGCCGACGAATCTGGATTGGTCCCCAAGCCTGATCTCATGAAATTTGAGGATCCTGACGTTCGCAGAGAA**TGA**

>ABCC-like

**ATG**CCAACTGAAGTCATCAAGACCACAACATCTCCCTCTTCCTCATCTTCGTTTTGGCATGAAGCAGACTTCATTACCAATGGAGAAGGAGGGGCCACTTCAAAAAAGACCGCACTTGATTACGGACCATCCGGGTTCCCCCACGAGGAAAACGATTTGATCCAGGCTCCACCTCTGCCCTCTTTGTCCCTTTGGGGACGGTTGTTTTTCAACCACTTCCAGGTCTTTCTCCCCAAGGAACGAACTGCCGGCTTGTCCTCAGACGAAGTCCCACAACTTGCCGAGGAATATACGGCCTCAGTTTTGACTTCCATTTGGATCCAAAATCACAAATACCTCGGACCCAAGTCTTGGTTCAAGAGCACCACTCTCCGTTTGATCCTGAAATTGAATCAAGGCTGTCTAGTCCTCAGTGGAGCCCTGGAACTAGGACGGATCATCTTCTCGTTACTGGTCCCCATGTCATTGCAACAAGTGATCCATTACATCGATGTGGCTGACCTCGAGGATCAAGGGTTCCCGTTCCACGGCATTTTTTACGCCTTGGTCCTCGTTGTCGTTAGCTTTCTGGGATCACTTTGTGAGACTCATGCCTTCTTCCAACTCAACATGGCGGGCATGAACAGTAAAACGGCTTTGGCCTCGGCAGTGATTCGAAAATCATTGCGACTCAAATGTCCCAAGGATAGTGACGTGGTAAATCTGATCTCAGTCGACACCGTGTTCCTTCAAAAGGCTCTCCGTTTCATTCACCTCCCTTGGGCTTGTCCACTGCAGATGTTATTGTCAGTTCTTTACCTTTATTCTATCCTCGGAGCCAGTATTTTCCCAGGGTTGGCGGGGTTGTGCCTGATTCTCTTGGTGAGCTTGTTCCTCTCCCTACGAATGAAAGAATGTCAATGCAATCAACTCAAGCGGAAGGATGACAGACTCAAAAAAGTGGTGGAGGTCTTGAACCACTTAAAAGTCATTAAGTTCCAAGTCTGGGAGAGAACCTTTGAACGATCAGTTCAGGGCTTGAGGGAGCATGAACTGGAAGCGCTGCGAGAGTTTCTTTTGATCCAAGCATTTCAAAGCTTCATGTGGAATTGTGCGATCTTCCTGGTGGCATTCCTGTCCTTTGCCAGTTATGCCCTCATGAATTCTGGCTCCTCGGAGAACAAGCTCACGGCCACCACGGCCTTTGTTTCATTGGCCATTTTCAACAACATGCGAAATCAGTTCCGCTTGTTTCCCAGTGGAATTGCTTCCTGGTCCCAAGGCTTCGTGTCCTTGGATAGAATGGACGAGTTCCTCGAGACCGATGAATTACCGGCGTTGGATCAACCGGATGGGATCAAGGACGACCGCAACGGGAACATCAAGATCCAACCCGGAGAATTGGCCATCCTGGCTCGCAAGTGCTCTTTTGCTCGGACAGGAGCCAAGGATCCTAATCTTTGTCTGGAAAACATAAACCTGTCCATCATGAAAGGGGAACTCATCGGAGTGATTGGGCCGATGGGGTGCGGAAAGAGCACACTTATGGAGGCCCTCTCTGGAGAGCTTTACAAAGCCCATGGACAATTAGAGATGAGTGAGCGAACCCTTCTGGTAGCTAATTCACCGTGGTTGCGGAGCGGAACCATTAGGGACAATATCCTGTTGGGCTCGCATTACGTCAAGCACAATTATGACAAGATCGTCGAGGCATGTGGGCTCACTCGAGATTTCCAAGGCGTTCCAGATGGTGACCTCACCTGGGTCCATGGAAATGGGGCTGTGGATCCGAAATTAAGAAAAGCCATTTTCAAAGACGTCATCAGTAATGAAGGACTTTTGAGACGGAAAACCAGGGTCATGATCGTGAATGATTACGCTATGGTGGCGAAAATGGACAAACTTATCCTAATGCAAAATGGCACCATTGTCGACTTTGGCCGATTAGACATTTTGGAACAACGACACAATCTGGATATGGAGGTTTGGCAATCATTGAAATCTATAGATCCTTTAGAAGCTGAAGAGGACAAAGATGCCATCAAACCCAAACCAAAGCTAATGAAAAGACAAGACACAATCAGCATCTTGAGATCGGTTCAAACCAGCGGCTTTGACAATCTGGACGACATGTGTTACGAGAGTGTCTCTAAAGACAACTACCTCTTCTACTGGAAGAATATGGGTTACGTCGGATCTCTTGTGGGTATTACTTTCTACATAGCTTGCCAAATTTTCGAGATCAGTACCAAATTGTGGTTAGCCCGATGGACTTCCGAGGAGCAAGCTAACCTTCGTGCCGAAACATCTGAAACTAGAGAGGATAATTTCATTCTCGTCTACGGATCATTAGGTCTTTTACAAGCCTTTAGTTATCTTGGCGCAGTGCTCCAAGTCAATAACACAACAATAGGGGCATCCTCGAAGATCCACAACTCGGTGTTTCACAAGGTGCTATACAGTACCATCGATTTTATTTGGTCCACCCAAGTGGGTGCTGTGGCCAATCGGTTCTCTCGGGATCTGAATGAGCTCGATGTGATCCTGCCTAGTACCCTCAAAAGTTTTATATTCCAATGCATGCGGATATGTGGAACCGTCTTCATAGTTCTCTTCACGTTTCCTAGCAACATCATCTCTACGGTCCCGCTTATTGTGGGCATCTTGTGCATCTTCAAGTTCTACATCCAGATCTCAAGGGTCTTTCGACGTTTGTCCTCCGCCACAATGGCCAGCTTGAACGGGTTCGTCACGGAAAACGTGAGTGGTGTGGCATCGATCCGGACCTTCCGCAATCAGCGTCAGGTTTTGGACCTGAGTTTGAACGAGATTGACGCTCATCAGAAGAACTGCTATATGGAAATGGTCTCGGATTGTTGGCTCTTTGTCCGACTCCAAGTAATCACCACGGTCTTCATCGCTTCTCTGTCTCTCGTCATTGTCTGGAATCGTCATCATATCACTTCTCATATGGCTGGATTGTGCCTCACTTCGGCCATCATGATCATGTCCGACGTTTTTCTTTTTACAAGGTATGCAGCCGCCTTAGAAAAATCTATGGTGAGTGTGGAGAGGCTCCAAGAGTATCAGGATATCGAGCAAGAGTACGATGGTCTCTACTCGGACAAGAGTTTCACGATAGCTCCGAAAAACGATAAGGCTGGTGGACATCTCACTTTCCACCGACTTTCGGGGCGATATCCCGGTGAAGGTCAAGTTTGTATTAAAGACGTGTCCGTGGATATTAGACCCGGTCAGAAAGTTGGGATAGTGGGACGGACTGGAGCAGGAAAATCAAGCATTGCCCTACTTTTATACCGAATGATTGAAAAGCTAGATGGATCAATTCTAATCGATCACTGTGACATAGCCAAAATGGACTTAGCTCATCATCGCTCTCGAATAACTATCATACCCCAGGATTATGCTCTCTTCAGTGGAACGCTGCGATTCAATCTGGATCCGGAGAACAAACACTCAGACTTTCACTTGTGGAATACAATCAGAAGCACCGAGATGTTGGATTTTATTCAAAGCTTCCCCAACAGTCTCGATTTTATTATTGATTCAGATGGTGTAGGGTTAAGCGTGGGCGAGCGACAGTTAATCTGCACTCTCCGTGGGCTCCTCACTGAAAACAAAGTGGTGATTTTGGATGAGGCCACGTCGGCATTGTCAAACTCAATCGAGAATCGCATTCTCAAATCTGTGAACGATCATTTTGCCACTTCGACTATCCTCACCATCACTCACCGAGTTCACAAGGTCTTGAAGTGCGACAAAATTCTGGTCATGGATTCCGGCCGAGTCGCTGAGTTCGACTCTCCTGCTCGGCTTCTCAATAATCGACAATCAATGTTGTATAGGATGCTCAATAATCCATCCCGAGATAGTTTAACTTCAATT**TAA**

>ABCC2 isoform X1

**ATG**ACACAACCGCCATGGATACCAAGTGAACCGCCTTCTTTCTGTCCAGACCAATTTTGGAACAGTTCTTTATCATGGAACACGGATTCTCCACGGCTCAGCTTATGTTTACAAGACACCCTGTTAACCGTGATTCCCTGCGTCATCCTATTTACCTTGGAGCCGTTCTGGCTGTGGTTCACGAAGAGTCGACAAAATTCACATCACGTATTTCCAACACCCAGGCAATTTGGCAGAGTATTCTCTCTCCGCATTCTCCTTACCCTTCTTCTCACGGCTAGTTGCATCACTCAAGTAATTTTGAATTCAAATCGGAGAGGTGCTTTTGGCTCTGAAGTATTTCATTACACTCTCCTTATTGGCACATACCTTGGTGTGATTGTTTTTGAGTGCTTGGATCAATGGAACAACACGGCCAACTCGCCCCCTATATTTCTTTTCTGGCTTTCCATGGGAATTTGTCAACTTCCCGTGTTACAGACGCAAGTGACATACTTACTGACTCATCCCAATGACGGGATTTTTTGGGTGGTGTCTCTCGCCCATTACCCAATCATCATTCTGCAATTGATAGTCCACACTGTGTCTTCGTCAAAGCCACAAGTTGGGGATTGGTCCTTGGAAGCGAGTGCCTCATTTCCATCCCTCCTTTGCTTCACATGGATGAACAGATTCATTTGCAAAGGCATATTTTCTCCAATAACCGATCACTTCCAGATTCCCAAAGCTCCTTCCATGTTGGATCCAAGTCGATTGCGGCAAAAATTTGCCTCTCATGATCAGGGTGGCAAATTGATCTGGCAAATCATTCGTACCATTTGGAAGCGATTGGTACTGGCCTTTACTCTCGGACTTTCCGCCATCATACTCTCGTTCCTTCCCACCCTGTTTTTGAGGCTTCTAATTTTGCACTGTCAAAACCCAGAGCAATCTTGGAAAGGATACTTTTTCGCCATCACTTTATTTTTGGGAGTCACCCTGGAGTCAGTGGTTCTTAACCAGTTTTTCAAGCAAGCTACCGTTTTAGCTCAGCATGTAACAAGTGGTTTGGCGGCCTTGATATTTCATAAAACGCTCAAGATTCCTTCTCGAGTGAGACAAAATCTGGAAATTGGCCAAATCGCCAATTTGATTAGCAATGATATTGCCGCCTTGGGAAACAACGCTTGCATTATCACGAATGTTTTCACAGCACCCATTATCATCGTCATTGCCATGATATTACTCTGTTTGGAGTTAGGATTCTTGGCATTTGTGGGTGTGGCGATATTTTTCCTTCTCATGCCAATCACACAAATGGCGTCAATTCGTCTCCGGAAGCACCAGAATCAGTACTTGGAATTGAAAGACCAACGATTGGCTTTCTTGGCTAATTTTTTGCAAAACATGTCCGTGCTGAAGGTAAACTCATTGGAACCAGCGATGCTGAGAACAGTGAATGGGATCAAGGATCGAGAAGTGGCAATTATGAAGAGATTGGGCAGAACCTTAGTGTTGGCCAAAATACCCTTTTCGTTGGGCCCAACGATTTTAGTGCTCTCTTCATTTCTCATAGTTACCCTTGTGATTCCTACTAGTGTCCTTAAGGCCGAAAAAATATTTGTGTCAGTGGCTCTCTTCAGCATTCTCAAAAGGTCAGCCGAGCAATTTCCTATTGGTTTAGGGTGTCTTGTGAAGGCTTTGGTGTCATTTGAAAGGATTTCTAAATTCTTGGAGATCCCCGAGTCCACTGAAGACGCCATACAAATTTGTAATGCCCAAGAGAACTCCAGCGAGGACATTGCAGTGGAAATCATTGGACCTGACTTTTGCGAAAAACTAGGTCAGGGTGAATCCATTGCCATTTATTCTCACCCAAACACTTCTTGTCAGTTACCAGCAACATTTGGTGGCAATGGAGAGCAATCAAATCTTCAACTGAAAGTATACCGACCAATGTCCTTTGCAGCCACTCAACCCTGGATCCAAAACGATACAATGAAGAGTAATATCCTTTTTGGAAGACCATTGAAGGAGGAAAAATACAAGGCTGTGTTGGAATTGTGTCACCTAGATCAAGATTTGGAACTTCTACCTCAAGGAGATGAAACCGACCTTGGGCCTTCAGGGATCAATTTGAATGAAGACCAGAAGCAGAGGGTATCTCTAGCCAGAGCGTTATATTTGCATAGGGATGTTTGTGTTCTGCAAAGCATTCCGTCGAATGTGGACCCTCAAGAAATTGTCACTTTTCTTCAAGATGCGACTGTTATCCTTTTTACTCAAGATAAAAGAGTTTTGTCGCTGGTAGACAAAATTATATCCTTAGACGAAGATCACTCTCCCAAGATTGTAACTGTAGAAGAACTTGATTCAGGCCAATCGCACTTTCATCAAAAATCTTGTGCATCTTCACCCAGCACCACCAAAGTGGAACTTGTTGCAATTGACAATGAATCTTGTTCTCTTGGATCAGTGTCATGGCGAGTGTATTTGTCTTACATCGAAGCTTTTGGCCCTCGCTTTGTCCCTTTTGTGTTAACCTTGTTCCTTGTGGCTCATGGCCTTGACCTGGCTTGTAAATATTGGCTTTCCCTTTGGGCCAAGGCCAACAACGACGAAGTGTCTTCTAACGCAACCCCTTTATATTATTTGTGTATTTATGCAGCGATCGGCGCCGTTCAAGGTGCCTTCATAATCACCAAAGACATCTGTTTATTCATGGGATGTGCCAGAGCGTCCCGCAAAATTCACAAGGACCTCGTTTCTACTACGCTGGCGTTTCCTCTGTCATTTTTCAAGACTAACTTGTTTGGAAACATTCTGAACCGATTCTCTATCGATATTAGTGGGATTGATGACGTGATCCCCATTCAACTAGCCGTGTTCTTGAATTGCGTATGCGCTTTAATCATGATTTTTATAGTCATTGGATCTACCATACCCATCTTCATAGTTTTTATCTTCCCTTTGGCCATTTTCTATTATTCCATTCACATCATTTACCTAACCTCATATCGTCAACTTCAAAGACTTAACGTCTTAACCAGATCTGAGCTTCTCAACTTCATCTCTGAGATCACACTTGGAGCGGAGACCATTCGCATGTTTGACCAAGAACACAGAATTCAAAACCTTTCTGAATTCAAGGTCAATAAAGCTGTGCAGTCCTCATACACTATGGAGATGATGCGTCGATGGTTGGCCCTTCGTTTGGAATTTTTGGGTAATCTAATTCTTTTTGGAACGGCTCTTCTGTCTGTGTGGAACAATGATTCGATCTCGCCAAGTTTGGTTGGATTGATTTTATCGTTCTCTTTGGAAGTGACACATGTATTGAACTGGCTGATGCGAGCAATCAGTGACTTGGACTCACAATTCGTGGCTTTGGAACGGATTCGGAGATATCAGGATGTTCCAAGGGAGGACATCTGGACCACTGAGGGAGATCATGGATCGATTTCACCAATCGAAGAGCTCTCGGTAAACATTGGATCAACTTTGAAGTTCTCTGTCAAGACTGGGGAGAAGCTGGTCGTTGTGGATCCATCTGGAGGAGCTGCTCGTGATTTTATGAGTCAAATGTTCCGTTTGAACGATTCAGACAATGGAATCATATTATTAGATGGTCAAGAAATTCACAACTTTGAACAAACCCATCTTCGAAAACAGATCCGATTTGTTTTAAAAGATCCAATTATTTTTCCGGGTGCTGTGAGGTCCATTCTAGATCCAAATCAGACTCACGAGGATGATGACATGAGGCATGTTCTCAGCAGATTGGGAATGCCTGATCTTGATCTTGATGTGGAACTCAACTCTTACGAGGACCCCGTTTCTTCATCCAATTTGTGGCTTCTTGTTGTTGCAAGATTTGTCCTGGAGCCACCGGCAATCTTATTCCTTGAATTGCCTTTTCTGAAGGATGATGAGAACGGGATCATAGGCAAAATTAACCCATTACTAGATGACGAGCTCCGATCAGTCTCTGTGATCTTTTTTTCACAAAAACCCCTTCTTTTGGACGCTCAATCAACACAGATTGTTCAAGTGGAGATA**TGA**

>ABCC2 isoform X2

**ATG**GAGCTTTGTTCTGTTCCCATATTTGATCTAAATCAGACTTGGTTCGTTTCCAAACCTCAACTTTCACCATGCATGATCCATGGAGTTCTTCCTGCAGTCCCAGTTGCATTCTTGTGGCTATCATTCGTCCCTTCGTTATGGTACTCTTGGCGCAATCGGCATGGTCAGGAAATCCGAAGCAGGTCCTCACTCAACCTATTAAAGCGGTTCTTAAATATCTTGGTAGTTGTGAACTTGGGGTTCCAACTAACGGCAAGCAATCGGACCTTTGTGGAAGATTTGCATGTGGCTTTTTTGATTCCCACACTTATCTTGGGCTTTTTGGTATTAGTGGTTGAATCTTACTGTCGACGGATTTCATCACCAGTTTTGACCATCTTTTGGATCACCCTGGCTTTTGTATCGATACCAGTTTTTGTGGAACAATTTGGAGAGGTGCTTACTCAATTCAGTTTTCGTGGTCTCTTTGTTCTGGTCACCTTTGAACCGGTTGTTATTTTAACCGCGATCTTGAATTGTTTCGCGGAAAAGACAAACTTTCCTAGAGATCATTGGTCGAAAGAAGAGACTTCCTCTTGGTTTTCCAATGTGTTCTTCACGTGGGTCAATGCCGTGGTTTGGGATGGATTTAGACGAAATTGGTTTTTGACAAAGTTACCCACTAGCCTCCCTTTTAGACTTCAATCTATACCCAATGGGCTCAAATTGCGAAACAACTTGAACCATCAGTATGGGTTTTGGAGAGCCTTGATCAGACCCTACTATGGAACTCTCATTCTTCCAACCATTCTGGCCTTGATTTACTACACGTTGTTGTTTGCTCAACCCCAGATCTTGAAGTTATTGATCCTTCATTTTCAAGACGAATCGGATTATGAATGGCATGGATTGATTTTAGCCGTAGTGTTGTTCCTTTTGGCCTTCGCGAGTTCCTTAATCAAGGAACATATCAACGATTTGATGATCAAAGTGGGCATGCAGATGAAAAGTGCTCTTCTTGATCAGATATATCATAAGGCAATCACATCAGTGGGTGTCAAGGACACGGAGCAATTTTCCATCGGAGATATATCAAGTTTTCTCACCGTGGACGCGGAACGTATTTACACGTTCATGCCTTACTGTCAAATGGTTTGGAGTGTTCCATACCAGGTTCTTGGTGCAAGCACATTTCTTTATTTTGAAATGGGACCGGCTGCGTTTGCTGGTTTAGCGACACTGATTCTCATGATCCCAATCAGTGGATTAGCCAGTAAGAAGTTGAAGGATTTGCAAGAGTTGGAAGCTCGGGTTCGGGACAAGAGGATCAGAACGACTGTGGAGACCATTTCTGGGATTAAGGTAATCAAGATGAATGTTTGGGAACGACCTTACGTCCATAGAATTCAAGCGTCAAGACTGCAAGAGATTAATCTAATGAAACAGACGGCGTTTCCCAAAGCCATCTCGAGTGCGGCTTTGATCCTGGGTCCACATTTGTTCATCATGGTCGTGTTCGGCATCTATGTATCGTCTGCCGATGCTCAATTGACCTACGATAGAGTGTTTGTCTCCTTGGCTTTGTTGGGTCTCATGAAATTTCCAATCATGATGTACAAGATGGTACTTTCTGAAGGAGTGAACCTTTGGGTGTCCTTGCAGAGGATCAAGGATTATCTGGACGTAGATGAAAATCTGAATAATCCAACCCCTTTGGTGGCACCCATTGAGGATGGCATTGCAATGTGTCTCAACAATGCTTCTATTTACTCCAATGCTGGGAAGGCATTTTTGAAGAATGTGGATATCAGCTTTCCTCAAGGGTCTCTAACCATGATTCATGGGTCAACGGGCTCTGGGAAATCGTTACTCTTGGCGTGTTTAGCTCGAGTCAAATCCATTAATCGTGGATACCTTCAACAAAAAGGGACCCTTGCTTTTGTTCCACAAATTCCTTGGATCCAAGACAAGTCTTTCAAGGAAAATATTGTTTTTGGAGCACCCTTAAACAACAGATATTTTCAGCAAGTTTTGGACGGATGTGCTCTCCTTAAAGATTTAGATTCTTTCGATGACTACGACGACTCAATTGTGGGAGAAAACGGCAGTCGACTGTCTGGGGGCCAAAAACAACGCCTGGCTTTGGCCAGGGCAGTATATCAGAAAAGTGATATCTATCTAGTGGACGACCCTCTCTCATCCTTGGACGAATTGGTTCGAAGTCAAGTTTTCTTGAAGGTTTTCAGTTCAGACTTTGGACTTTTGCAAAACTCAACGCGGATTATGGTGACAAACCAGATGGATTTGCTCCAATACGCTGATCAAGTCATTCTTTTGGAAAACGGAAGTGTTCACTCAAGATGGAATAAACGAGCCAAGTTGAGCCCCTCAGTTCAATATACTCAACTCTACAAGAAACCTACAGGAAAGGATGTTCAAAACACAGAGACCGATTCCCAAGAGCTCGCTGTTTTGGGACCGGTAAAAATGAGAAACTATTGGCAATATCTCCGACTTTTTGGCAAACTCAACTTCGCTGTTTGTCTCTTGCTTTTTACAGCAATTCAATTTGGACAAACTGGAGGCTCGATATGGTTGGCCAAATGGACGGAAATGGCCGGAAATCAGGGATTGAGTCAGCACATGCTATATTTGGGGATCTTCGCGGCCTTTGGCATTGTTATGATTCTGGCCAGTATTCTGAAGGATCTACTCCTTATCAACAGTTGTGCAAACGCATCCATGCACTTGCATACAAATATGATTGAGGGTGTTTGCTTAAGTCCAATGCGGTTTTTTGAGAATAATCCAATTGGAAGGGTTGTCAACCGATTTACACAAGACATGGCCATCACTGACCAAGCCGTGCCAACTTTAGGCGCTCATCTATTTTGGGCCACCAGCCAAGTTGTGGCAGTCCTCATTGTGATTAGTTTGGCTAATCCGTACTTAGCTTTGGCCAGTGTTCCTTTCGGATTGGTTTACTACATGGTCCAAAGAGTATACATCGCTTCGTCTCGCCAACTCAAACGACTAGAAGCCTTGAGCAAATCCCAAGTATTGTCCCATTTCAATGAGAGTTGGACGGGGAAAAGCACAATTGTCACCTTTCATCGGAACATGGCATTCGAAACGGATTTCCAAGAAAAAGTTGATTCCAACGCCCTTTGTTATTATCTCAGTCAAGCCATCAACAGATGGTTGGGATTGGTTATGGCCAACATTGGGAGTGTTTCAACCTTATTGATATCTTTGGTTGCAGTGATGGAACGTGGGATAATCTCTGTGGGATTAGCCTCTTTGGCCATCACCTTCTCATTTCAGCTGTCGGAAAACCTGATCTTCATGGTTCGAATGATCTGTGAATTGGAGAAAAACTTTGTGTCGGTCGAACGAGCTCTGGAATATTGCGATTTACCCATGGAACCAAATGGCCCAGAAAATAATCACTCTAGCTCCTTGTGGATCCAACGTGGAACAATTTCCTTCGAATCCGTCGGTCTAAAGTACATCTCCAAATCGGATTTCTCGCTGAAGAACCTTTCCTTCAACGTTCGAAGCCAAGAGAAATTCGGGATTATCGGAAGGACAGGAGCGGGGAAGAGTTCAATAATATCTGTTCTCTTCCGCTTAGAGAACCCTACGGTCGGTCGAGTGTTATTGGATGGCCAAGATATCATGACCAAGCCTTTGCCGGAACTACGTCGGCAAATGGCCATCATTCCTCAAGATCCACTAATCTTCTCGGGCTCGATCAGGTTCAATCTAGACCCTTTCCAGGTTTATTCAGATATTGATTTATGGAAAGCCTTGGAAGCTGCGGAAATTCATAATCTTGTTCGAAGACAACCAGGAGGATTGGACGCGGTGGTTACGGGGAGACCTGATCAGTTTTCGGCCGGACAAAGACAACTCTTTTGTGTGGCTCGAGCCATCTTGAAACGGTCCAAAATTGTTATCCTAGATGAGGTTACAAGTTTGGTGGATCCCAAAACGGAGGAGATTATCCGGAGAACGATTCAGACCCACTTCAAAGACTGTACAACTATTAGTATCAGTCACAAGAGGGAGGAATGGACCAATTTTGATCGCATTATGACCTTAGATGAGGGCGTAATCCAGACGATAACTATGCCATCTGGTCATTTCTTGTCTCTT**TGA**

>ABCC4

**ATG**GACGCAGAAGAAGCCTTCCAGCAGAAACCAAATCCCCGAGAAAATGCCAACATTTTGTCGATCGCATTCTTCTGGTGGCTGAATCCTTTGATGTGGCTGGGTTACAAGAAGAACCTTGAGATTCAAGACCTCTACAGAGCCTTGAAGATCGATCAATCAGCAGACTTGAACTCGAGAATAGAGAAACATTGGCAAAGAGAATTGGATAAGCAAGCACAATCAAACAACAAACACAAACCGTCCCTATTTCGAGCTGTGATCCGCACTTTCTATGGTGAATTTTGCTTCTTGGGATTGTTCACATTTTTCGAGGAATGTTTGATCAGAATTGCACAACCAGTATTCCTAAGTTGGTTTGTGGCATATTTCACCCCTGGTCAGACTGCGATCTCAAGGACAGAGGCCTATATCTATGGATTTGCAGTTGTTCTCATGTCAGCCTTGTACACCTTCACTCATCATCCGTATTTCTTTGGTGTGATGAAAGTTGGTATGCGGGTACGAATAACCCTCTCGGCCCTGGTTTACAGAAAGGCCTTGAAATTGAGCTCGGCTGCTTTGGGCCAAAGTACCGTGGGTCAAATGGTCAATCTTCTGGCCAACGATGTCAATCGATTTGATGGGGCCTGTTTATTCATTCATTACCTTTGGGCTGGTCCTCTTCAACTTTTAATAGTGGTCTACTTGACATGGAACGAAATGGGTGTGTCAACAATGGCAGGAGCTGGAATTATTCTCATATCCGTGCCACTGCAAAGTTGGATCGGACGAATGTTTTCCAAATTGAGGTTGGAAACTGCCAAGAAAACTGATACGCGGATTCGAATCATGAATGAAATTATCAATGGGATCAAAGTGATCAAGATGTACGCTTGGGAGTTTTCATTCATGAAACTCATTGAATCCGCTAGAGAATCTGAAATGGATGTGATTCGGCGGACAGCCTACTTCCGCGGATTTAACTTTAGCTTCTTCTTCGTTGCTTCCAAAGTGATTTTGTTGGCCATCCTCATTCCATACGTTTTGACTGGTCAAATCATTAATGCTGAAAAGGTGTTTCTCACCTTATCCTTGTACAATACTGTTCGACTTTCGATGACCCTATTCGTTCCTTTTGCCATTTCTATGGGATCAGAGGGTTTGGTCTCCATCGGTAGAATTCAGAAATTCTTGATGATGGAGGAACGAGATAAAACTTGTTTAGCATGTGTCCACGAAGAATCCTCTTCAAAGGAGAAACCGATTTCTTTAGATATTTGTGGATTGACTGCCTCTTGGACAGACGCAACTGCCGACCCCACGTTAAATAACGTATCGTTTTCAGCCAGAGCAGGGGAGATCGTGGCCATTGTAGGAACTGTGGGAAGTGGAAAGACATCCATGTTGCAAGCTGTCCTTGGAGAAATTCCGAGTAAGGAAGGCAAGATCAACATTCGAGGCCGAGTGAGCTATGCTGCCCAGGAACCTTGGGTGTTTTCTGGTTCTGTCCGCCACAACATCCTATTTGGCGAGAACTATGATGAAAAACGCTACATGAAGACTCTCGAAGTGTGTGCTCTAGAACATGATCTAGAACAATGGGAATTTGGAGACAAGACTTTGGTGGGAGAACGTGGGGTAGCCTTGTCTGGAGGACAAAAGGCCAGAGTGAGCTTGGCTAGAGCCATCTACCGTGATGCTGATATCTATCTCCTGGATGACCCATTATCGGCTGTGGATGCCCACGTGGGCAAATACTTGTTTGAGAATTGCATTCAGTCGCATTTGAAATCAAAACTAGTGATATTGGTTACCCATCAGATTCAGTTTCTGAAAGATGCCGACATGATCTTAGTGTTGAAGAAAGGCTCGATCCAAGGACGAGGAACGTATGCTGAACTGGAATCATTGGGCACTGATTTCACGGAGTTTATTTCGGAAACCAAGGAGGATCAAGAGAATGAGGAAGAAGAGGAAGACGCAACGTTGATAGATGGGGAAATCCCGAGCCCAAAAGAACACAGGAGCTTGTCAATTCGATCCTGTCTTCAACGGAGAAGCCGTGGTTATTCTTTGGGTGGTGACTCAATCATGTCAATGATCTCAGGTGTGAGTGAAGACATTCTGGAGTATGACCAAGTGATGGCCGAAGAAAATAACACAGAAGAAAAGAAGAAACCCAAAGTAGAAGCTGAATCGGTTGTATTGGGTGCCGTGTCCTTGGCCGCATATTGGTCGTATTTTCGTGCTGGAGCAGGTGTTTTAGTGTTGATATGCGTACTCAGCCTCAACATTGGCGTTCAAGTCATTTTCACGGGAGCAGACGTTTGGCTGGGAATATGGACCAATCAAGAAGAGCAAGAGATGCAACAGTCTTCCATATCAGCGAATAACTCCACCAATACCACAAAAATTTTCACTATGGAAGATATCAGCCAATCCAATACAAACAATCTCTATATTTTCGGGGCCATGACTCTGTCGTTGGTGGTGGCTTCGTTGATCCGAACCATTCAGTTTCTTCTTGTGTGTAAGAATGCCTCTACTCAACTTCATCATTCCATGTTCAAGCGAATCATCCGAGCACCGTCCAGATTTTTTGACATCAATCCAGTTGGCAGAGTTCTTAATCGGTTTTCAAAGGACATGGGATCAATTGATGAGTTATTGCCTCCTGTTTTCTTGGACGTTATAACGATCTTCTTGACCATGGCTGGAATAGTATCCGTGATTGTCTGGTATCAACCAATTGTGTTTCTTCCGACCTGCATCATGGCTGTGCTCTTCACCTTTCTCCGCAAATTCTACCTCTCTTCGTCCAGAGCCATTAAAAGAGCTGAAGGTGTGGCTAAATCGCCTATTTTCTCTCAACTGGCTTCTACATTAGGTGGATTAACATCAATTCGTGCATATCGAGCTGAGCACATTTTAGTGAAAGAATTTGATCGCATTCAGGATGTCCATACGTCAGCGTGGTACTCTTTTCTGGCAACGACGCGTTGGTTTGGGCTTTGGTTGGATTGGTTGGTGGTGGTATACTTGGCATGTTGCGTCTGCAGTTTCCTCTTCATGAACGGATCCTCTCAAAGTGGCGACGTGGGCGTGGTTTTGAGCTCGTGTATCATGTTGACCGGCATGCTTCAATGGGGTATGCGACAAACAGCTGAAATGGAGAACCTCATGACCTCTACTGAAAGAGTGCTCGAGTACGGCAAGATCCAATCCGAGGCTGAGCTCAAGACTGATCATCCGCCCCCTGGTTATGATGAAGAGGCGTGGCCTGGGACTGGAGTCCTTGAATGCAACGATGTTTGTCTCCAATATGGAGAGGGCGAAAAGATGGTCTTGAAAGGCGTGAACTTTCTCACGAAGCCCCGGGAAAAGGTCGGGATCGTGGGAAGGACAGGCGCGGGAAAATCCTCACTCATTGGAGCCCTCTTCCGTTTAACCGAACCAACTGGACAAATCATTCTCGACGGAGTCAATATTCAACAACTTGGCCTTCATGCCCTCAGGAAAAAGATCTCAATCATTCCGCAAGACCCTCTGGTTTTCACTGGCTCTTTAAGAAAGAACTTGGATCCTTTTGACGACTATCAGGATCATCAGATCTGGCAAGCATTGGAACAGGTTCATTTGGACCAAGCTGTGAAGCACATGTCTCATGGTTTGGAAACTGAAATGAGCGAGGGTGGCTCCAATCTTTCAGCCGGCCAACGACAACTCATTTGTTTGGCTCGCGCTATTTTGAAACATAACAAAATTCTGGTTTTGGATGAGGCCACGGCCAATGTTGATCCAAGAACGGATATGCTCATTCAGGAGACAATACGTACTCGATTTGCGGACTGCACTGTTTTAACTATCGCTCATCGACTTCATACCGTTATGGATTCCGACAGAATTTTGGTCATGTCAGACGGAAAAGTTGCGGAATTTAATACTCCTTATGAGCTACTTCAAGATCCACGAAGTGTGCTGTCCGAACTTGTTGCTAATGTTAGTTTGGCATCTCAATTAAAACTGAGAAGAATCGCCCGAGAAGCATTTGTGAGACAAAAGAACTTAGATTTGGCTGAGCCCAAGATCACAAATAATATACGGAACAACTCTTTTGTGGAAAATTCTAAGGTTCAAATAATGGTCGAGGACGACGAGAAAATTGTCACTAAGCTT**TGA**

>ABCC5

**ATG**GGGGACCGACCAATCATCCGACCGGAATCAAATATTCAAGTCCTACCCATCACTAAAAACAAGAAATCTCCTGCTAATGGCCAACCAATCGAGAACATTAACGGAGACATTTACGAAAATGGACTTGGAGGATTTGTACCGAGCTGTCCAGAGACTCGGTGGAATAAGAAATATAACACGGCACTCAAAACCCTGATTCCCTATCGACCTCAGCCAAAAGAGGGACGTTATTTGCCTATGGAAAAAGTGGGATGTTGGAGCTACCTGACCCTATCATGGTACACTGGAACCATGTGGAAGGCCTTTCGTACTGGTTTGTCTCGATTAGATCTTTTAGAAGTGGCTGAACGAGACCAAGCCCATGTCACAGCACAACGCCTTCAACGGATATGGGAACAAGAAGTGGAACGAGCTCACAAACAAAGTGATCCCAAGAAACGAACACCTTCTATGACCAAGGCTGTTATGAAATTCTGCGCCACTCGAGTCTGTATTGCAGGGATAATGCTGATGATTTCCATTGTTCTTCAGTTCTTGGGACCAAGTTTGGTCTTGAAATTGCTATTGGACTACATCAACCAACCACTTGAAGACCTTAACCACGGGTGGATTCTGGTTTTAATATTACTACTGACTCAATTCTTCCGAACAGTCACATTTTCTCTCAATTTCAATATCGGGGTACACACTGCCCAGAAAGTTGTTGGTGCTCTTCAATATCTGGGTTATTCAAAGCTTCTTCGGTTAGCCTCTCCTAATGAGGCTGCTCTGGGACAACTAATGACCTTTTTCACGGGAGATGAGGAAAGAATCATCGAAGGTGTCATCGTGGCAACTCTATTCTTAGGAACCCCTATCCTCTTCATTCTGTCTGGCGTGTACTCTACTTATCTGGTTGGACCGGTGTCCTTGGTGGGCTTCCTCATCATTCTATTGTTTTATCCTGTCATGGGTGGTATTGCAGCAGCGATGAGCAAAATGCGATTGAAAATTGTACATGTTACTGACAAGCGAACTACACAGATGAACGAGATCTTAAACTCAATCCGTTTAATCAAGATCTACGGTTGGGAGAAATCGTTTGAGGAGAGAATTCACAAAATTCGGCGACAAGAGATCAAGGAATTGAGAAAGGCAGCATTTCTACAATCTGCTTTTACGTCCATCACTCCATCAGTTACTATTATCGCCACAATTGTTACGTTTTTTGCCCTTACTGCAAGTGGATATCGATTGACAACTCCCGAGGCTTTCACCGTGTTTTCCGTGTTTGTATCACTCCAATTTACGGTTGGAACATTGCCCTATGCATTGAAGTGCCTCGCCGAAGCAAATGTGTCCTTTCAACGGCTTCAAAAATTCATGGAACTACCAGAATACGCCAATCCCTCATCCATCCATTTTGATGGCGAAGAAGCCAAGAACTTGAGACTTCGAATGAAAAACGCCACATTGGCCTGGACGAAACCTCCCGAATGGATTTCCCCCGAGGATGAAAAGAAAAAAGACAAGAAAAATAAAATGTCCACCCACGCTAATGGTCTTGGAACCGCTAAAGAAACCGAACCTCTCAAGGACCAAGACGATGATCAACAACCCTACACTTCCTGTCTTTTTGACATCGATCTTGAGGTCGAAGAGGGCACTCTCTTGGGTATTGCAGGCCCAATTGGTTCTGGGAAATCATCGCTCTTGTCTGCTATTATGGGCGAAATGAAACAATTGACCGGAGACGTGAAAATCCGAGGAAGTCTGGCCTTAGTTTCTCAGCAAGCATGGATCTTCAATGGAACCCTCAGGGACAATATTCTCATGGGATCTACTTACGAAAAGGACCGCTACAAATCTATTCTTCGGGCCTGTACACTCGAAACCGATCTTGAATTACTTCCCAATGGCGATCAAACTGAGATTGGTGAACGAGGCGTCAATCTGAGTGGAGGGCAAAAGCAACGGGTCAATCTAGCTCGTGCCCTCTATGCCGATTTGGATATTTATCTCTTAGATGATCCACTATCAGCCGTGGATGCCCGAGTTGCTAAAAGGATCTTCCACGGATGTGTTTTGAGGGAGTTGAAGGCCAAAGGCAAAACCGTTCTTCTGGTGACTCATGGGATGCAGTTCTTGGAAAAATGCGATAAGGTCGTTTACATGTCCAACGGAACTATTCTGGAACACGGAACGCATTCTGAGCTCATGAAAAGAGACGATGGTCATTATCTTCACATGTCTACTTTTGATCAAAGTCAAAAAGAGGGCAGAAACCGAGGGGCCTCAGAGTGCTTATTGGATGATGCCAAACCCGTCAACGAGTCCGGGAGCGACAAAAAAAGAAATGGTTCCATTGTATCGGAAACCCTCGACGAGACCGATGCCGCCAAAGGACAGTTCGTGTCTGATGAAGTCGATGTTCTGTATTCTGGATGGGGTGTCCTGTTGAAATATTTCCAAGCGTGCGGTGGTCTGGTTATAATGTTTCTGCTCTTTTTGGCCATTTTGGCCTTCTCATTGGCCCGACTATTTACCTCAATTTGGCTTCAAATTTGGCTGGATCATGGAGATGGATTGCAAGAAGAGCGGATGCAAAATGCCACTCTTTTTAACGAAACATTCTCCGACATCGAGCTCAAAGGATATGTAACCGACAACCCTCGTCTGTGGTTTTACCAAACGGTCTACTTCCTCAATTTAGGAGTTATGGTCTTCATTGGTTTGGTCAAAGGTATACTTTTGGCCTTCCAATTTCTCAAAGGATCAGCTAGACTTCACGATGCCATGTTACATCGAGTCATGAGAAGTCCTATGAGCTTTTTTGATGCCACACCCTCTGGTCGGATTCTCAACCGTTTCTCGAAGGATTTGGATGAGTTGGATGTTAGGATGCCATTCTATACGGAATTCGTCTGCCAAGCTGTCCTGTTTTGTGTTACTCAAATCTCCGTGGTGTGTTTCGTTTACCCCTACTTTGTGCCTCCATTCACCCTCATCATTTTGGTGTTTGTTCTCTTGGATGTTATCATGAATCGTGGAATTTTGGAGACCAAGAAGCTGGAAAATATCACAAAATCTCCAGTGATCCATCATCTTTCTTCTGCCATGGCAGGAATCTCTATCATTAGGGGCTATCAGAAGCAATCCGTTTTCCAAAAGAAGTTTGAGCGCGATTTGAACATACATCTTAGTGCTTCGGCGTTGTTTCGATTTGCGAATCGTTGGTTCGCATTCCGCATGGATTTGATTGGAATGGTGACAATCGTTTTGACGGGAATTTTCACGGTTCTTTTCAAAGGCTCCGTAACACCAGCCATTGCAGGTTTGGCCTTGGCGAATGTGTTTCAAACGTGCACGTTCATTCCATTCGTAATGCGAATGAAAGCAGACTTTCGGGCTCGTTTCAATTCAGTGGAGCGAGTGGCAGAATACGCCAACGATTTGCCTCAAGAGGCCCCAGAGCATATTCCCGAGAAGAAACCTGCGGAATCGTGGCCAGAGAAAGGTGGCATCATTTTCAAAGACGTCTCGTTACGGTACAGACCAGATTTACCTTGGGTTCTTTCAAAAATATCAATCGAAATCAATGGAGGCGAGAAAATCGGAATTGTCGGTCGAACTGGAGCTGGAAAGACTTCCTTGATCACCACCCTACTCAGATTGACGGAAATTGAGAACGGCTCAATCACAATGGACGGTGTCAACATTTCGGAGATTGGCTTGCACGACCTCCGGTCAACGATTGCTGTGATTCCACAAGATCCAGTGCTTTTCCAAGGGACTTTGAGGTATAACGTGGACCCCTTCAACCAATATTCTGATAAGGAAATTTGGATTGCTCTAGAGAAGTCTCATTTGAAGGAGAAAATCAGTGGAGCTCAAAATCAACTCTCAATGACGGTGGATTCTGAAGGGGACAACTTTTCAGTGGGGGAAAAGCAATTGATTTGCTTGGCCAGAGCCTTACTCAGGAAGAACAAAGTGTTGCTATTAGATGAGGCCACAGCTTCTGTGGACGTCAAAACCGACTATCTCATTCAGGCAACCATTAAAGAAGCATTTGTTGATTGCACCGTTCTCACTGTTGCACATCGACTTCACACTGTTGTAAATTATGATCGGATCATGGTCATGCAAGCTGGAGAGGTTGTGGAGTTTGGTCCTTCGAGAGAGCTCTTATCCAACCCCGATGGAGTGTTCTCGTCCATGATCAAATCCGTCCAAATGACCAGTCCTTCTCACCCAACCCTCTCCGAAACTCCCATAGCTGAG**TGA**

>ABCC7

**ATG**GCGGACCTCGCTTTCAACCGCACCCGGTCTCCGCCTCTGGGTCTATGGAGTCGCCGCCTGGACTTCAATCAAAGCGCATTCTGCGGCCTTCAAAGTTGGCAGTTTTGGGACCCGGTTCATCAGAGTTTGGGCGCCTGTTTCACCCGCATCGTCTTTATCTGTCCCATGCATCTGCTCTTGGCCGTGTTCTCGACTTACTACGTCGGTTACCGGGTTCGGAATCTGGGTGGGCGGGCCTCGCGGTTGGCGCGGGTGTGTCTTTGGGCTCGCGTGCTGGCCGCTCTGGCCTTGATGCTCACGCCTTTTATCGGTTATGTCCTACTGTGGGGTTTGGATGGCGATCCCTTGGCCCAAACGGGCTGGTCTCAGGTCATCCAGACCTGTCTCAAAACCGGGGCTTGGCTGGCCAATCTCTTGTACACGTTGGCCCTCTTCAACCGCATTAGTTGGTCCGTCCGTGGCCATCGCAGCCTTCTGTTGATCTTTTCGTTGTGCGCCATGGTCGACTTGATCCAATATCATAGTATTTTCGCGGAATACCCGCCGGGTAAAAGCCGTCAATCTGATGTCATCTTCGGTGCCGCTCTGATTGAACTTCTGGGACTGATCGTGTACTTTGCCTCTTTGCTCCCGGGACCTTGGACTGACGATCCCAGTCAACCCGTGTTGTTGCCGGCGGATGAGCTCGAAGATCCGCAGTATCGACACGTACGCCACGCCGAGTATTTGGGCGTGGCTAAAGAGCACACCCATTGGTTATCCCGAGTGTCTCTGCAATGGGTTCAACCGCTGATTCGAAAAGGTAAATGCGGTCAACTGCGCACGGCCGAAGACGTGTTCAATGTGCCGATTGACATGTCCACGCCCGTGAGTAGCCAACGATTTCAAATGACCCTCAACGAACTCATGTCACCGCAAGGAGAAGTGAGTTTGTTTCGGGCGCTGTTTAAATCGTTTGCCCTCCGGTTTTTCTCCATCGGTATCTTAAAGTTTGTGGCTGATTGTGCGGGCTTTGCTAGTCCGATTCTGCTCAACCGCTTGGTCTCGTTTATGGAAGACGCCGATGAAGACATTCGATGGGGTTATCTGTACGCGGGTGGACTGACCGTGTCCACTTTTGTGGTCGCCATGTGTAATACCCACTTCAACATGTTCATGAGTGAGCTGGGTCTCAAAATTCGGGCCTCCATCATCACGGCCGTTTATCAACACACCTTGAACGTAACCTCAGCCAATCTGAGTCGATTTAGTATCGGCGAGGTGATCAACTTCATGAGCACGGACACGGATCGCATCGTGAATTTTTCGCCCAGTCTGCATGCGGCCTGGAGTTTGCCATTTCAATTCACCATTACGATGGTGTTGCTGTATCAGCAAGTTGGATTGGCATCCTTGGCTGGCGTAGGTTTCACGGTGCTCATGATCCCTTTGAATAAAGTAATTGCGGACACCATCGGTCGACTCAGCACCAAGATGATGATGGCCAAAGATCAGCGAGTCAAAACCATGTCGGAAGTGCTCTATGGCATTCGTGTAATCAAGTTCTTCGCATGGGAAGGCTTTTTCACCCATCGAGTTAACGGTCACCGTAAAGAAGAACTGAAACATTTGAAAGGACGAAAGTATCTGGACGCCGTGTGTGTCTATTTATGGGCCACCACGCCTGTCATTATCTCGGTGTTGACTTTTACCACCTACGTGCTTATGGGCAACACATTGACCGCGGCCAAGGTATTCACTTCGGTGGCCTTATTCGCCATGCTCACCGGGCCTTTGAATGCCTTTCCATGGGTCCTCAATGGCGTTGTTGAAGCCACTGTCTCAATCAAGCGAATCACGGCCTATTTAGGTCTCCAGGAAATCAATCGGAGTAGCTACTTTTCCGAAGCCTCCGACATAGTGGAGGAGGAATTAGTGCCCGACATGGACATTGTCATGAGTAGAGCCAGTTTTGACCTTTCTGCTACCTCCAACGCTGAATTCAACTTCAAATTACAGGGCCTCGATCTGCGAGTCGAGAAAGGAGAATTTGTGGGGATCGTGGGTAGAGTGGGTAGTGGAAAGAGCTCCCTTCTGACTGCGTTGCTCGGTGAATTGTCTAGAAGTCGAGGTAGCATATACGTCAGAGATCCCCGTGAAGGTGTAGCTTATGTCCAACAAGAGCCGTGGCTCCAACAAGGCACCGTTCGCGAGAATATCCTTTTTGGCAAACCATACGAGCTGGAATGGTACCAAAAGGTGGTGGATGCCTGCGCCTTGAAGGAAGACTTCAAGCAACTGGCCTCAGGTGATGACACGAATGTGGGCGAGGCTGGGGTGATGCTCAGTGGAGGCCAAAAGGCCCGAGTGGCTCTGGCCCGAGCAGTCTACCAAAATAAGGAGATCTACGTGGTGGACGACATTTTTTCGGCCGTGGACGTGCCAGTGGGAAGTCACATCTATCGGAACTGTCTCATGGGTTTGTTGAAAAATAAGACGCGAATCTTGTGCACGCATCATCCCCGATTTTTGGCCGGCGCTTCCAAAGCGATTTTGATGGAGAATGGAGAAATCGTCGATGAAGGATCACCGAAAGATATTTTGACTAAGGTGGATTTTGAACGAGATGCGGTTGAGAAACGGGATCGGAAAAACACAGAAAAAGACGAAGATCCGAAAGAAGGTTGTCAACTAGAGTTGGACTTAATCGACGACGAGACACAGAGCGCTGGCCATGTGAAACTGACAATTTACAAGAACTACTGGAGGGCTGTGGGCAACTTTCTCTCGCTTGCGATTATCCTTGCAATGGCCTTAATGCAAGCTTCCAAGAACTCCACCGACATTTGGCTAGCTCAATGGGTCTCCGAAGACTCGAACCAAAACGAATCCCATTCGCCGATCTTATCTGGCATGGCATGGCTTTCACCCCTTCTTGAAGGTGCCTCTGACGAGAGGTATCACTTGGTTGTGTACGGTTCGATTGCCGTGTTCAACACCTTCATGAGCTTGGCTCGAGCATTCCTCTTTGCCTTTGGGGGCATTTGTGCCGCCAAGGCCATACATGAGAAACTCCTCAATGTCATTATGCGGGCTCGCACGCAATTCTTTGATGGAACACCCGCGGGTCGAATCCTGAACCGATTCTCTTCGGATATGTACACAGTCGACGACAGCCTCCCGTTCATCTTGAACATCTTCTTAGACCAAGTTTTCGGTGTGTTTGGACCCATCATTGTGTGTGCCTATTCTGTCCCCTGGATCATGTTGATCCTCGTCCCTTTGGCCTTCCTCTATTACGACATTCAGAAGAATTATCGACCCGCTTCGCGCGATCTCAAACGAATGGGCTCCGTATCATTGTCCCCAATCTACGCCCACTTTTCCGAAACGCTGAGTGGCCTGCCCACCATTCGAGCCATGAAAGCTGTCAAACGATTCAATTCGGAGAATGAGGATAGACTCGAGGCCAATCAGAAAGCCCAATACGCGGGCATTGCGGCTGCTCAATGGTTAGAATTACGCTTGCAATTGATCGGTTGTGCAGTCGTCACCGGCATTGCGTTGATCGCCGTGGTCGAACATCATACCGATAGCGTAAATCCGGGCTATGTGGGCCTAGCTATAAGCTATGCGCTGGGAATCACGGGAAAACTCTCGGATTTGGTGAAGAGCTTCACGGAGACGGAGAAACAACTCGTGGCTGTTGAGAGGTGTTACCAATATATTGACGAGGTCGAGCCTGAACCCAGCGAAGGCCAAAGTTCTTGTCCAGCCCATTGGCCTGACCAAGGGGTGCTCGAATTCCGCAATGTGTCCATGCGATATCGAGACAACCTGCCTTGGGCTCTCCAAGGGCTCAATCTCAAGACTCAGCCCCATGAAAAAGTGGGTATCGTTGGTCGAACAGGTTCAGGGAAGAGCTCCATATTCCAAAGCCTCTTTCGAATGGTGAACATTGCCCAAGGCGAGATCTTCTTGGATAATGTGAACATCCACCACCTGAACTTGTCTGACGTTCGCCGCAATCTGGCCATCATTCCGCAAGAGCCTTTTCTCTTCAGTGGCACAGTGCGTGAGAATCTGGATCCTCTGGGTATGAACTGCGATTCAGAGTTGTTGTCCAGTTTGGAGAAGTCGCATTTGGCACAAGCGGTGAGTCGGTTGGGCGGACTTGAAGGGAAAGTCGACGAGCGTGGACGGTCCCTCTCCATGGGTCAACGGCAATTATTCTGCTTGGCTCGAGCCATCCTCTCGCCTTCTAAGCTGGTTTGCGTGGATGAGGCTACGGCTAATGTGGATCTAGAGACGGACACTTTAGTTCAGGAAGTATTGCGAAGCGCACTCAAAGACCGAACTGTGATATCTATCGCTCACCGCATCGATTCGGTTTTGGGAACGGATCGAGTCATTGTTATGGCTGCGGGCCAAGCTCTAGAAAGTGGATCTCCACAAACGTTGTTACAAGACAAAAGTTCGGCTTTCGCTCGACACGTTTCGCAGAAC**TAA**

>ABCC9

**ATG**CCGACTGTTACTGAAATGTGCCTCTGTCAAAGCGGCTTCAATACCACGATTGAAGACTCTGACCCTCCGGATCGTGATTGCCGCATCGAATTTGCCATGATCACATTGAACAGTCTGTTTATTTACGTGACACTGATGCTACATTTGCATGTGAAGTTCTGTTCGCCGAAGAAGCGACCTGCCAATCGAAGAACAACCATGAGCCAACCCCATCCGAGAAAAGCCCGTCCCTCCAAGAAACGGATCGTTGGTCATAACTTGATTTGGATCCTCATGCTCGTTCAAGTGTTGTGCAGCCTCTTTGCCTTGGTCGAGATCCTCCTGATCTTAGCCAAACGAGACGTGTTATTCCTAGTGTCGTTGATCGAGACTTTGATTGCATTTATCACGTCCATTCTCATCCCAATCTACTTTGATGCCGTTGAGCTCCGAGGTGGAACGATTCCGAGGATAGTGCAACAGTTCTTTCCGCTCGTCTGTCAGTTTTTTGGGTCTGTTATCATTCTGGGACTTCGAGCCAGTCGATTCTCGAGCAATACTAGTGGAACGGTGGAGTCCTTTGGCGCCAGACATCTTATATTCAACCTCACCTTGCTTCAACTCGTTGTAAACACTTCACTTTTCCTCATTTTGATGTTTGCTTTGGCAAAGAAGGTGCTCCTTCATGATCGAAGAGTGGGCAAGAAACGACCTCCACACCCTGGGAAACCCCAATACCCAAACGACAAGCCAATGACACCAGATCACGAACCGGATATGGATACGACCTTCGTGTATCAATACAACCACGTGTCTTTCTTTTCCAAGATGTGCTTCACATGGCTCTTGCCCCTTCTTCAACGTGGCTATCGAAACCCCCTGGAAATGAGAGATTTGCATAAGTTGCCGGCGGAGGAAAAAGCCCGGAGGCAATATGAGCATCTCAAAGCCTGTGTCAAAGAGCCGGAGAAACGCGTGCTAAAAGCCTGCCTTATCATGAACTGGAAACTAATTCTCCTGGGGGGTTTCTTCAGATTTTGGTCGGACGTCTTTGGTCTGGTTGGAGCGATGAGCATCAAGTTTATTGTCGACTCAATCTCGGAGGACTTTGAACATATTTTGGCTGAAGAGAGCAACAACCATACGTCCCAAGGGAATGGGTCCTCGTCATTGATTACGAACTCGGGTTATGACTCCCACTCGATTTTCAGCAACGTCTCAAATGAGGACCACTCTTTGACCCCATTGACTTTGGAAGAGTTCTTTTCGGATAGCTTCGTCATTGCCATTATTATATTCCTGGCAGCCTTGGGCCAAGGAGCCTTCTCACAAACGTCAAGTCATCTCCTGACCATTGCTGGAGTCCGATCCAAGAATGCCTTGCATGTTTTGCTTTATGAAAAAGCTTTGCGACTTCCGGTGGGGACCGTCCAACACACTCCCGTTATCAAAGAGGCTCTCTGCATATCGAATACTTGCTCCACAGATGAGGCCGAGGAGGCCAATGAGGGCAACATTGACATTGGTTTCATCACCAATCTAGCCTCGGAAGATATCTTGAACATCCGGGAGTTGATCTGGAATATTCATTACCTGTGGGCATTACCTCTCAAGGTCATTGTTATTATTGGTCTCCTGTATCTGAAAATGGGGATAAGTGGCGCCACAGGCGTTGTCATTGGAACTTTGATCATTGTCCCACTCCAATTTCTGACGGGAAAACTCATGTCAGACAACAATAAGCGGATATTTCAAGCTCAAGACATCCGCTTGTATAAATCCACGGAAACTATGCAAGGCATGAAAACCGTCAAATTGGGATGTTTAGAGGAGGCCATGTTTGCCAAAATTGATGATGCTCGGGATCGTGAACTTCGCTTTTTGCGCCGGGATTCCTTCTTCTGGTCCATCATGGCGTTTTTGGCAAGTGTTTCGACAATTATTGTGAGCACAGTCACCGTGGGGCTCTACGTGGCCTTGGAAGATACCAATTTTAGCGCAGCCAACATTTTCTCCGCTCTTGCTCTCTTGGGACAGCTCACAGTGTGTCTCTCCGTGTTCCCAGTGACAATTCCCATCTTCATCAAAGGCATTGTGAGCAGGTCACGACTGCTCGAGTTCTTTGCCCGTTCTGAAGTAAACATCTACAAAGATTACTCTGGCAACAAAAAACCCTCACATACTTGCGGTAGGACAGTGACAGCATCAACGGAAATAGGAACACTAGGTGAGGTCCATTTGGAACAAGGGCAAGAAGATTATGAAGACGAAGACGAAGAGGAGGAAGAAGAAGACGAAGAAGAGGGCGATGGCCTATCAAATATTGAGGAAGAGAAAGAATTCTCTGATAGCCAATCCAAATCTGCACCTCAAGACTCCAGGAAAAATAGCTGTCAAAACAACAACATTCATAGTTTTCCGAGCATTGCATTTTCCATCCAAAATGGCACCTTCTCATGGCCCAAAACCAACGCCAATGTTTTGCGTGACATAAATCTTGAGGTGAAAACGGGAGGTCTCACGATTGTCATTGGACCCTCAGGTAGTGGTAAAACTGCCCTCATATCTGCTCTCACGGAGGAAATGGAACGACTTCGAGGGAGCATCAAGTGGCATTTGCCCCCCGCTGTGGCTCTCACGGGACAAAGACCTTGGCTCTTGAATGCCTCGATACGTGACAACATTCTACTGGGACGGCCTTTTAAAGAGAAACGCTACGAAAAAGTTCTTCGAGCTTGCGATTTAAAGGCCGATATTGAACTCTTACCTGAGGGTGATGAGACGGAGGTGGGAGAACGAGGCGTCCTATTGAGTGGAGGGCAGAGGCATCGGATCGCTATTGCGAGGTGTATTTACTCGAAAGCCCCTTGCACGTTCTTGGACTCGCCTTTCAGCTCATTAGATTCGAACATCACCACTCACATCTTTCATGAGGGAATTCTCAGGATCCTGCTCAAACGGCGAAGAACTGTATTTATGGCCACTGATCGAATCGATTTCTTACAAAAAGCTGATTGGGTTATCTACATGAGCCAAGGCACAATCAAGGCCCAGGGTTCCGTAGACGAAATTATGAAGCACTATCCCGAATTACGTATCAATATCAAGCACATTATCTCAAGAACCTCGACCCTGAGCTCCGATGATGGATTGGTCGAGGGAAAAACTGCTCAAGAGAGATGGACTCTATTGAAAAACGTGACCATGTTCATGAAACAAATGCAAAGAGCGCGACTTGTCAAAGACCCCTCCAAGAGCAATAAGCCTCAATTTAGTCGGAATATAGGCTTGCTCAAGAAAAACAATTCCACGAACAAACTGGCCCGAACTCAAATCCGCATGGACAGTTCGTCGCAATTGACCTTATGCCACGACATCCTGCTCCCATCAGATGAATGTGTGGGTTCAAATAGCATCTTTGAACAAGGATCCGGTCTGACAAGGACAGACTCTCGTCGAGGTCAGTATGGAAGTCTACGGAAGCATCGGAAGTTCCGCACAAGCAGTGGATCAAGCGTCGGAAGTCACAAGCTCAAGTCAATTTCGCGAGCTTCCTCTTGGAGTACCAGCTTCAATAACGGTGTTCCTGTCACATCCCTTAAACATGGTCCTGTCATGAACCCGGCCAATCGGCGTCTAGCAGGAGTGGGAAGTTCCAATAGTTCCATTGGCCTGGTTCGACAAAGTGCCATATGTCAAGCTCCATTACCTTCCACGGGATCGTTTGGAACCCCTCGAACGGTAAAACATCAGGGCGAGTTTCAACTAATGCGATCGTGCCACCACATGCTGGCATTTAAAAACCGATTGGTCAACTCAGGGAGTAGTGCTTCGCTACCCAAACATAGCTCCTCAGAGCCTGAAGGGTTTTCTTTCGAAAGTAACAATATGGCCTCGTTTCGTTCTCGCTCAACCAGCCGACAATCTTCGTACCAAGATTCAAATGCTTCCTCCGTGGTTCTCCATAAACCTACTTATCCTCTGCATTGTCGTGTAATGAGGATGACGTCAAACGCCTCGGCCATCTCACAAATATCGGGGTTTAGTGATGATTTCCACGATGATGACGAGGACGATGGATTGATCGTCACCCGCGAATCGTCTAGCCAAGAGAAAAGGGAATATGGCCAAATTGGAGTTCATGTCTACATGGATTATTTCACGGCCGGAGGGCTCCACTTTGCAGCCATGTTCCTGGTCCTGTCTGTTGCCCTCCAAAGCATCAAAGTCTACATGGATTTCCTATTACGAGACTGGTCTCTGGAGGCCGGGAATGTTCATGACTCGGTCTCAATGTCCTATTTCACTTCGTACAGCACATTCTCCGTTCTCGTGTTAGTGTTCTCGTGTTCGGCTAATCTAATTGGTCAGTTAATTGGGGCCAGAGCTCGACGGACGCTTCACTTTCGGATGCTCACCAACCTGCTACGGTGTCCGCTCGACCTTTTTGAAGCCCATCCGATTGGACGCATCATCAACAGATTCTCCTATGACATGTTCGTGGTGGACCAGAAACTCCCTGCCTGTGTCCAACGCCTGGTTTTGGTGTCCTTGATTTGCATCTCGGCCCTGGTGGTCAATTCGATTCAGTCGCCCATTTTCATTGTGTTTGCCGTGCCGATGATTGCCATTTATTGGTGGCTTCAGCATTACTATCGATGCACATCGCGAGAGCTTCAAAGGCTCGATAGTATCAGTCGGGCACCCGTTCTATCGCATTTCTCTGATACATTGGGAGGGCTGAAAACAATCCGTGCCTTTCGAGAACAAAACCGATTCATCAACCAGCTGTGTGAGAAAATTGATGCCAATACCACGTCTTTCCTCATCCTTCAAAGTGGTTGCAGGTGGTTGGGAGTCAGTCTAGATTGCACAGGTGCAGTCATGGTTTTCGTGTCGATCATCATCAATCTGTTTGTGTCCTATCAGTATCCTGGGGAGCGGTCTTCGGCCACCATTGGCCTTTCGATGAATTACAGCCTTTTGGTACCCATCTACTTGGCTTGGGTAGTTAAGTTTTTGGCTGACATTGAAAACTACATGAACGCCGTGGAAAGGATCCTAGAGTACACGGATTTATGCGTTGAAGAGGAGTTCCAAACGGAGAACTGCGCGGACCAGCTCAGTAATGATGGGACAATTCGATTTGATAACGTTGGCTTGACCCACAGCCTTGATCAGAGGGCCATTATTTCATCGCTCAATCTGGAAATCCCCACCAAACAGAAGGTCGGGATTTGTGGTCGAAGTGGAGCCGGAAAGTCGACCTTGGTAATGAGCTTGTCCCGAGTGACGAATGTCCTTCACGGGAAAATCACTATTAACGATGTTGACATCGCAAATATTCCTTTGAAAAACCTTCGTCGTTTTATTTGGACCGTTCCTCAAGACGTTACCCTTTTCAGTGGAACCCTCCGCAGCAACCTCGATCCCGAAAACCAGTTTTCTGACGCCGAGATCTGGCAGAGCTTGGAACACATCGGACTCAAGGACATGGTCCAAAATCTTGCCAATGGCTTGGACGCAGAAGTGATTGAAAATGGAGACAACTTCAGTTTGGGTCAGAAACAAGAATTGGGCTTAGCCAGAGCCATTTTGCTCAAGCCTCCTATTTTGGTTTTAGATGAGGCCACGAGTGCTTTAGATCCGGCACGTGAAATCCAATTGCACAAGTGCCTCCTGAAGGCCTTCGCTGATTCCACTATAATCGCAGTGGCCCATCGATTGGCCAATATAGTCCCTTACGATCGTGTGTTAGTCATGGGCGATGGTCGAATCTTGGAAGACGGAAACCCAAGGGAAATGCTGAAGAAACCAATGGGCTTTTTTTCGTCTCTCTGGAGAGCAGCTGGTGAAAAGCCTCTT**TAG**

>ABCD2

**ATG**GCCGTGATATCCAGGTTCCTGGATGAATCTGGCAAGAAATTGGGTTTACCAGTTTTGGGTATTACTCGTGGTGTGTACGCTGCATGTGTGTTGAGCTACGTTATCAAAGTTGGCTATCCTAAATGGGTTCAACGAAAAAGGACCGCCTCCAAGGATCAGGATGACGAAGAGACGACAATGGCCACCAATATCGTTTCGGCTCGACAAAAGCACGTCAAGAAAGGCCCGGCAGTTAATAAGGAATTCATAGAAAGGCTTCAACGACTCTTGAAGATCATGATCCCAGGCCTTTGGACCAAAGAATTCGGCTTGCTCACCTTGCACACGTCGATTCTGATTTGTCGCACCTTTCTCTCTATTTATGTGGCTGGATTAGAGGGACGGATGGTCAGGTTCATCGTGCAAAAAGACGTGCAAAATTTTGGTTGGATGATGATCAAATGGTTTGGTGTGGCCATTCCGGCTACCTTCATAAACTCGTTGATTCGATACGTAGAAAGTCAATTAGCTTTGGCTTTTCGAACTCGTTTGGTTGGCTACGCATACAAACTCTATTTCAAGAACCAAACCTATTATCGAGTGTCTAATTTGGATTCTCGCTTGGAGAATGCGGATCATTGTCTCACGGATGACATCAGTGCTTTCTCGTCAACAGTGGCCCACTTATATTCGCACATCTCCAAACCAATGTTGGATTCGGCTTTGATTTTGTTCCAATTGTTCAGGCTTTCGAAGATGCAAAAGGCCAATAGCCTGGCCACTCCCATTATCACCACATTGGTGACCACGTTGACAGGCGAAGTGCTTCGTTGGGCCTCGCCCAAATTTGGAAAACTTGTCTCGGAGGAAGCCAATCGTAAGGGCTATCTCCGCTATGTCCATTCCAGGATTATCACCAACGCTGAAGAGATTGCCTTTTATGGTGGACAAGAGGTGGAATTGAATGCCCTTCAAAAGGCATATCGATCGATGGTCAACCAGAGCCAGAAGATCTTCAATCAACGCCTTTGGTATGTCATGTTGGAACAGTTCCTCATGAAGTACGTCTGGGCTGGAACTGGAATGGTCGTCATCGCCATACCCATTTTGACCAGTCAGCAATCCAAAATGGGCGGAGAAGGAGCCGTTTCCGATCGGACAGAGTATTATGCCACTGCCAAGAACTTACTGTCTTCAGGTGCAGATGCTATGGAAAGGCTCATGACGGCTTACAAGGAAATTGTGGAGTTGGCCGGGTACACGGAACGAGTGAGTACGTTGGTCAAAGTCTTTGAAGACTGCTCTGTAGGCAAATACCAACGAACTGCCGTTTCCACCGTGCAAAAGTCCAAAGGCAAACTTGAGCGAGGACTTTCTGATATTCTCCAATTCCATGATGGAGTTCCACTCATTCGAGGGAAAATCGTTCATTCTGCCGACAAGAGCACCATTATGTTGGAAAACGTTCCCATTGTGACCCCCAATTGTGATATTGTGGTTCCTAGTCTCACGTTGGAGGTGAAGTCAGGGATGCACTTGCTGATCAGTGGTCCCAATGGATGCGGAAAGAGCTCTTTGTTCCGTATTATCTCGGGATTGTGGCCGGTGTACGCTGGAACCCTAAGGAAACCTCCTCTCTCGTCCATGTTCTACATCCCACAGCGCCCATACATGTCCGTGGGATCTTTGCGCGATCAAGTTATTTACCCAGACACCCTGTCAGATATGAAAGCCAAGGGCTTAAATGACAAAGACTTGGAATCCATTTTGGACATTGTTCACTTGAAGCACATTGTCACGCGAGAAGGTGGATGGAGCAACATCAAAGATTGGAAGGATATTTTATCAGGAGGGGAAAAGCAGCGAATGGGTATGGCTCGCCTATTCTATCACAAACCCCAATTTGCCCTTTTGGATGAATGCACCTCTGCTGTGAGCATTGACGTTGAAGGCCGGATGTATGAAGCGGCCAAGGAGTCCAATATCACCCTTCTAACCATAACACACCGACCTTCACTTTGGAAGTACCATACGCACATTCTGAAGTTTGACGGCGAAGGTGGATGGAACTTGGACATTCTGGATTCTGGCGCCATGTTGAGTCTCAACGATGAGAAACTAAAATTAGAGCGGGAACTGGCCAGTGTTGCCAAAATGGAAAGCCGCTACAAGGAAGTGTGCGCTCTCATTGGCACGGAGGATCAAGTGGAAGAAGTGGATGATGAA**TAA**

>ABCD4 isoform X1

**ATG**AAAAGTCGGGCTGATAGTGTTGTGAAATATTACGGGTTCAATTGGCAGTTCTTGGTTCGAGTGACAAAACTTCACAAGTTTTTGTTCCCATCATGGACATCGATACCAGTGTTTCTGTTCGTGTTTATTCTCCTGGTTCGATGTTTGGAGGAATATGTAGGCTATTACGTGGGCCTGATTTCCTCAGACTATTATCAAGTTTTAGGAGACAAAGACTTTGATGCGTTTGTTCAAGTCACATTGAAGAGCTTAGGCATGATTGTGCTCATAGCCTTTATCAAGACTACTCGAATATGGTTCCAGGAAATGATTGTGGTCTCTTGGAGATTGGCCATGACAACGACTTTAAACCAAAAATATTTCCAGCATCACTCATTTTACCACATCAACATTTTAGGAAGGGGCTTTTTGGGCAATGCTGATCAGAGGATCACAGCGGATGTGTCCTCGTTTTGTTCCACGTACGGGTTCATCATTGCGGATATTTTGGTCTCGCCATTGACCATTGGTTACTACACTTACGATGCGTACACTCGTGCTGGGTGGATCGGTCCCTTGGGAATGTTCCTCTTTTTCGTGATCTCAACCGTGATTAACAAACTTTTGATGTCACCTGTGGTGAATGCTTCGGCTGAACAAGACAAACAAGAGGGTGAATTTAGATTTCAGCATGTTTCGACACGGATCCATTCCGAGTCTCTCGCGTTCCAAGATGGATCTGAAGTGAATCTAGAAAAGATCCAAGGGTCATTAAGGCGATTGTGCCGAAGTCAGCGAAGGGTTTTCAACCTGCAGTTCTTCCTCAACTTAGCCACAAACCTTTTTCAATACGTCAGCAGCATCGCTTCTTTCCTCGTCATCGCTGTTCCATTATTCAGTGGAGCTTACGACGACCTAACGGGCTCTGAATTGAGTTCCTTGATCTCAGAGAATGCTTTTGTGTGCATCAACTTGATCTATCAATTCTCGAAGCTTGTCAACATTAGCCCATATGTGAGCGAAATGGGTGGAGCCGCTCATCGCATTGCCGAGCTCATCGAGACCTTTCAAAGCCTGGAAATGGACCACGCCAACAAGAACAGAGATGGAATAGGAATGAGTCCATATTTGGATCCAAAAGTGGCGTTTCGATTGACCAATGTGGACGTACGTTCACCGGGTATGGATCGACCTTTGATTAAAGATCTCTCCCTGGACATCGAATTGGGCCAAAACCTTCTGGTTATGGGCCCAAGTTCTTGCGGGAAGTCCAGTCTTCTCAGAGTAATTAAAGGATTATGGCCCATTTCAAATGGAGCCATTGAGGTCAATCCTGACCTTAAGGCCATTTACTTGCCCCAAAGACCATACATGATGAGTGGATCTTTGAAAGAGCTCGTCCTTCATCCCATTGACACTCTTCAATGCTCTTTAACCATTGAGGACGAGCAAAGAATCAAGGACCTTGTCACTGAATTCGGTCTCGACCATATTTTGGAACGATGTGGAGGCAATCTCGACTATGATCCCCAATGGAACTGGTCTGATGTGATGTCCCCCGGTGAGCTCCAACGTTTGGCCTTCATCCGTCTCTTCTATCACAAGCCTGCCTTGGCCTTTCTGGACGAATCCACTTCAGCCTTGTCCTTGTCCGTTGAAGAGAAACTTTATGAAAAGTGCTCCAAGATGGGCATCACTTGTGTCAGTGTGGGGCATAGAGAAACTTTGAAAAAGTATCACACCAAGATCCTTGAGATCAAGGAAGAGAGCGATCACAATGCGAGCACCCATGTGAATACTAATGCACTCTGGACCCTTCGGGATCTA**TAA**

>ABCD4 isoform X2

**ATG**GCGGACAACCATTACGGATTTAATACGCTCTATTTGCGGCGGTTTTACCGTTTACACGGATTGCTCTTCCCTGGATGGCGATCGCTCAATGCGGCTCTCATGGTCTTGTTGGTCATTCTATCGGGCTTGGAGCAATGGTTGGCCTTGTACGTGGGTCTGATCTCGGGTGGTTACTACAAAGTGTTGGGCGATCAAGATTGGAGTGGTTTCAAAACCACCACTTTGGATGCCACGTTGAAAATCATGGCCATGGTTATTACCAAATCGGTCCGAGTGTACGTGACCAATGTCATGACTGTGGGATGGCGACGATCCTTGACCATGGCCTTGCATCAAATGTATTTTGCTCGCATACGCTATTACCGCCTTAATGTTTTAGACGAAAGCGGTTTGGACAATCCGGATCAACGTATCACGGCCGATGTATCGTTACTGTGCCAATCGTATGGAAGCATTATCGCCGATCTCATTGTGGTTCCGTTTACCATCAGTTACTACACCTATTCGGCTTATACTAGGGCGGGTCCAGCTGGACCCACGGGCATGTTCTTGTTCTTTCTGGCCTCCACCATTATTAACAAACTACTTATGTCGCCCGTGGTGCGATTAACGGTTGAGCAAGAGCGTCGAGAAGGCGATTTCCGCTTTAAACACGTGACAGTTCGGACGCATGCGGAATCATTGGCTTTCCACAAATCGGCTCGAGTTGAGTCGATCAAGTCCGATGAAAAGCTCCTAAGCTTGTGTCAAACCCAACAGAGCTTGTTCAATCGTCAATTGGCCTTGGATTTGGCCACCAATGTGTTCAGTTATATGGGCAGTATCGCTAGTTTCCTCGTGATTGCCATCCCGATCTTTTCGGGAGTCTATGATAACTTGAGTCCGAGTCAATTGGCTCAAGCCGTGTCGGAAAATGCATTCGTTTGCATGTACTTGGTGTTTCAGTTCACCAAATTAGTGCAAATGGCCTCGACCGTGAGTCGTTTGGCGGGTGTGACGCATAGAATCGCTGAAATGACTGAAGTGCTCATCAACATGAATCGGTCTTTTCATTGCCAAGGTCGAAAATCGTCGAATAATTCTGAGGATGAAGATCAGGATAAGCTGGTTGCGGTGGTGCCTGATCCGAAACCTTCCGAGAGTCCTCTGAAGAATCATTCGTTGATCTTGGAAAACGTTGATTTGATCACCCCCAAATCCAGGAAAAAATTAATTCGAAACCTAAATCTAGCCTTGGATCTAGGATCCAATCTTCTAATTGTGGGACAGAGCTCGGCTGGAAAATCTAGTCTTTTGAGAGTCATTGCGGGCTTGTGGCCTTTGGACCGAGGTGTGATTCAACGCAACTTGTCAGATTTGGATGTGTTCTTTCTACCTCAACAGCCTTTTTTCACGGATGGAACTTTGAGAGAACAAATCGTGTATCCACTGCAGGTTCTGACCTCGCAGATCGATCCTCAAGAGTCTGTGGATCTCCTCAACATCTTGGAAGAGGTGGGTTTGGGCGATTTGCCCAAACGATGTGGAGGCTCTTTGGATACGGATCCCAAATGGTCGTGGTACGATAAGTTATCACCGGGAGAAATGCAACGCCTGGCTTTCATTCGGTTGTTCTACCATTGTCCCAAAATGGCCTTTTTGGATGAATCCACATCAGCCTTATCCTTGGATTTAGAGGATTTGTTATACAAAAAGTGTCTGGAGAAGAATATTGCTCTCGTGAGCGTTGGTCATCGAGAAACACTTCGACAATACCACGACCAACTTCTGACCATCGGTTTGTCTGATGGCCAATGGACCCTGACACCAATCTCTCGTCAT**TGA**

>ABCE1

**ATG**CCGCCCAAAAGTAAGCGAAAAGAGGCGGATGAGGGCGATACTTTGACTCGAATCGCCATCGTGAACAATGATCGATGCAAACCCAAACGATGTCGGCAAGAATGCAAGAAATCTTGTCCCGTGGTTCGGATGGGCAAATTGTGTATCGAGGTCACGCCCAATTCCAAGATCGCCAACATTTCCGAGGAGTTGTGCATCGGTTGCGGCATTTGCGTCAAGAAATGTCCTTTCGACGCCATCAATATCATCAATTTGCCCTCGAATCTGGAGAAAGAGACCACGCATCGTTATTCGGCCAACTCGTTCAAGCTGCATCGACTGCCCGTGCCACGCCCGGGGGTGGTGTTAGGCCTGGTCGGCACCAACGGCATCGGCAAATCGACGGCCCTCAAAATTTTGGCCGGCAAACAGAAACCGAATTTGGGTCAGTACAGTGATCCGCCGGATTGGACCGAGATTTTGGCCTATTTCCGGGGATCTGAGCTGCAGAATTACTTCACCAAGTTGCTCGAGGACGACTTGAAACCCGTGCTCAAGCCGCAGTACGTGGATCAAATCCCGCGAGCCGTCAAAGGCTCGGTTCAAAGTCTTTTGGACAAGAAGGATGAGATGAAGAATCAGGACGAGATTTGCCAAGTCTTGGATCTGACCCGGGTCAAGACTCGGAATGTGGACGAGTTGTCGGGTGGCGAGTTGCAGCGCTTCGCCATCGCCTTGGTGTGCATTCAAAAGGCCGATATCTTCATGTTCGACGAGCCCTCGTCGTACTTGGATGTGAAACAGCGGCTAAACGCCGCCCGCGCCATTCGCAACCTGATGTTGCCGCAAACGTATGTGATTGTGGTCGAGCACGATTTGGCCGTGTTGGACTACTTGTCCGATTACGTGTGCTGTTTGTACGGCGTCCCGGGTGCGTATGGTGTGGTCACGCTCCCATCGTCGGTGCGGGAAGGCATCAATATCTTCTTGGATGGGTTCATACCCACGGAGAATCTGCGATTTCGCGTCGAGTCGCTCGTCTTCAAGGTATCGGAAACGGCCACCGGGGAAGATGTCAAGCGTATGACTCGCTACGATTATCCTCTCATGATCAAGACCATGGGTAGTTTCAAGTTAGAAGTGGAGGCCGGTCAGTTCTCGGACTCTGAGATTCTGGTCATGTTGGGCGAAAATGGCACGGGCAAGACCACGTTCATTCGTATGTTGGCCGGCAAACTCGAGCCCGATTCTGGATCCGGCGAAGTGCCCCAATTGAATATTAGTTACAAGCCCCAGAAGATTTCGCCCAAGTCCGAGGGCACCGTTCGACAGCTTATGCACAAGAAAATCAACGAGGCCTACATTCACCCCCAATTCGTGTCGGACGTGATGAAACCCATGAGGATCGATGAAATCTTTGATCAGGAGGTGCAGAACTTGTCCGGCGGTGAATTACAACGTGTGGCCATGGCTCTCTGTTTGGGAAAACCGGCGGATGTTTACCTAATTGACGAACCCTCTGCTTATTTGGACTCGGAACAACGTCTCGTGGCGGCCAAGGTTATCAAGCGATTTATTCTGCACGCTAAAAAGACAGGTTTTATCGTGGAGCACGACTTCATCATGGCCACGTATCTGGCCGATCGTGTGATTGTGTTCGAAGGTGTGCCCTCGGTGAACACCAAAGCGTGTTCGCCGCAGTCTCTCCTCACTGGCATGAATAAGTTCTTGCAACAGTTGGAAATTACATTCAGAAGAGACCCCAACAACTACCGGCCGAGGATCAACAAGCTGAACTCTCAGAATGACACCATGCAAAAATCAAGCGGAAACTACTTCTTCTTGGAAGAT**TAA**

>ABCF1

**ATG**GCACCCAAGAAAGGCAAAAAAGGCAAGAAACAAGACGACGAATGGCCCGACGACGAGCAAGACAAGAAAATAGAGGAGAAAATGAAGAATCTCATGGTGGCAGACGACCCGGAAGTGTCCCAAGATCAAGAAGGGATCGCCTCCAATCCCGTAAAGGCTAATAAGGGCAAGAATAAGAAAAAGAAGGGATTTGTCATGCCCGAGAGTGAAGATGAAGACCAAGACCACAGTCCAGATCAAGAGCAGGACCATGAAGGCAAGAGCAAAGAGAGCAAAGAGAGCAAAGCCAAGAAAAAGGACAAGAAAGGCAAGAAATCCAAAGATAAAGATGAAGAAGAAGACTCGGAAAACGACGACACGGAGGATGAGGACAAGGTCAAAAAGGACAAGAATAAGCACAAGAAACATGACCAATCCGAAGACGATGATGATCAAGAAGATCAGGATGAGAAGAATCGCCAGAAAAAAAAGTCCAAGGTCAAGAATAAGAAAGGAGGAGGAAAGAAGAAAAAGGAAGAGGAGGAAGAGGAAAAGAAGGATGAATCCAAGGATTCCCAAGACTCCAATGAGGATGAAGACGAGGACGACCAAGATTCGGATCAGCCTAAGGACAAGAAAGCCGCCAAAAAGGTGGTCTTGACGCATAAAGAGAAGAAAGAGCTGAAAAAGAAGCAGAAACTGGATGCGGAAATGGCTCGAATCACCAAAAAAGGTGGTGAAGGTCACTCGGAGTTGGGCGACAATTTCACTGTGGCCCAAGCTCTCAAAACCGGCGGAGCCTTGGCCCAAATGGAAACGGCTGTGGACATCAAGATCGACAAGTTCAGCATCGCAGCCAAGGGCAAAGATCTCTTCCGAGACGCTAGTTTGCTCATCGCCCAAGGCCGAAGGTATGGTTTGGTGGGCCCCAATGGGCATGGTAAAACCACGTTACTTCGACATATTGGAAATCGAGCCCTTCAGATTCCGCCCAACATTGACGTGCTCTATTGTGAGCAAGAGGTCATGGCCGATGACCGGTCTGCCCTTGAGACCGTTCTTGCCGCTGATGTAAAGCGCACAGAATTGATGAAGACCTGTGCTGACCTTGAGAAGCAGCAGGACAAGGGCAAGGATGTAACTGACCAACTCACCGAGGTCTACGATGAACTGCGAGCCATTGGAGCTGATCAGGCCGAACCCAAAGCCCGGCGATTGCTGGCAGGTCTTGGATTTACGCGAGAGATGCAAGACCGACCCACGAACAAGTTTTCGGGTGGATGGCGGATGCGTGTGTCCTTGGCCAGAGCCTTGTTTATTGAGCCCACCCTGTTGATGTTGGATGAGCCCACCAATCATTTGGACTTGAATGCCGTGATCTGGTTGGATAACTACTTGCAGAATTGGAAGAAAACCCTGTTGATCGTTTCTCACGACCAGTCGTTCTTGGATAACGTCTGTACCGATATCATTCATCTCGATCAGTGCAAGCTTTGGTACTACAAAGGAAACTACTCCATGTTCAAGAAGATGGTGGTGCAAAAGCGTCGCGAGTTGAACAAAGAGTACGAGAAACAAGAGAGACGAATCAAGGAGCTCAAAGCCTCGGGTTCATCCAAGAAAAAGGCCGAGGCCAAAACGAAGGAGGTGCTCACGAGAAAACAGGAGAAGAATCGCACCAAGCTTCAGAAGAATGACGATGATCAAGGGCCCACGGAGCTCTTGGAAAAACCCCGCGAGTACATGGTCAAATTCAGATTCCCCGAGACCTCTCAACTTCAACCACCCATTTTGGGATTGTTCAATGTGAGCTTTAAGTACCCGGATCAACCATATTTATTCAAGAACGTGGACTTTGGCATCGACATGGAATCCAGGGTTGCTATCGTAGGTCCCAATGGTGTGGGCAAATCTACTTTCCTGAAGCTCCTCATGAACGACTTGACACCTACTCAAGGTGAAGCTAGACAGAATTTGAGGCTCAAAATTGGCCGGTATGATCAACATTCCGGAGAGCATTTGACCGCCGATGAATCGCCCACAGAATATCTAATGCGCTTGTTCAACTTGCCCGTCGAGAAGGCCCGTAAACAATTGGGATCCTTTGGTCTTCAATCCCATGCCCATGTGATTAAGATGAAAGACTTGTCTGGAGGACAAAAGGCTCGTGTGGCGTTAGCCGAGCTCACATTATCGGCCCCGGATGTGGTCATTCTGGACGAGCCCACCAATAACCTGGATATCGAGTCCATTGATGCATTAGCCGAGGCCATCACAGAATACGAAGGTGGTGTGATCATCGTTTCTCACGACGAGCGTTTAATCCGTGACACTGGTTGCAACCTTTGGGTGATCGAAGAGCAAACCATCAATGAAATTGAAGGTGGCTTTGATGATTACAGGAAGGAGGTCCTGGAAGCTTTGGGTGAGGAGGTCAACAATCCATCCTTGATTGCCAATCAAGCAGTCCTCCAAAGC**TGA**

>ABCF2

**ATG**CCGTCCGATTACGCGAAGAAGAAGGCGGCCAAAAAGAAGGAGCTCTCCAAGGGCAAAGGCGGCAAGAAGGGCACTAATAGTGCCACGGACACGCCCGAGGACACGCCCACCACCACCAACGGCACCAACACGCCTACTCATGAGAATGGCAAAGGCGTCACGGAAGAAGAGGAGCTGTGTAGCTTGCTGGAAGAGCAGGCTCGTTTGGCTGCCGATGCCCGAGCTTGCACCGGTGTTTTGGGCATTCATCCCATGTCCAGGGATATCAAGATCGACAACTTCTCCATCACTTTCCATGGAGCCGAGCTCTTGCTGGACACCAAATTGGAATTGTCTTGCGGTCAACGTTATGGACTGATTGGTGCCAATGGCTCAGGCAAGTCCTCCCTGTTGGCAGTGTTGGGCAACCGTGAGGTGCCGATTCAAGATCATATTGACATTTACTATTTGTCCAGAGAGATGCCCGCCTCAGAAACAACGGCTTTGGAAGCTGTCATGAAAGCAGATTCCGAGCGAATCAAACTCGAGAAATTGGCCGAGGAACTGGCTTTGCTTGAGGATGACGAATCTCAGGATTATCTCATGGAGGTATACGAGCGCTTGGATGAATTGGGTGCTGATACTGCCGAGGCCAAAGCTTGTGGTCTTCTTTTGGGTCTGGGTTTCACCAATGCTATGATGCATAAGAAGTGCAAAGACTTTTCGGGAGGATGGAGAATGCGTGTGGCTTTAGCTCGAGCTCTCTTCATCAAACCCCATCTCCTTCTGTTGGATGAGCCGACAAATCATTTGGATTTGGAGGCTTGTGTCTGGTTGGAAGAAGAACTCAAGAACTACAAACAGATCTTGGTCATGATTTCTCATTCACAGGATTTCATGAACGGCGTTTGTAGCAACATCATTCATCTGGACAAAAAGGTGCTGACCAGTTACGGTGGTAACTACGACGCGTTCGTCCGAACTCGATTGGAATTGTTGGAGAACCAAGCCAAGCGATATCAATGGGAGCAGGATCAAATTGCTCACATGAAGAACTACATTGCTCGTTTTGGTCACGGTTCGGCCAAATTGGCCCGACAAGCCCAATCCAAAGAGAAGACTCTGGCCAAAATGGTGGCCGGAGGATTGACCGAGAAGGTCGGAGCCGACAAGAATATTAGCTTCTATTTCTACTCATGTGGCGAGATCCCACCTCCAGTCATTATGGTTCAGAATGTCAGTTTCCGATATAGCGAAGATACCGAATGGATTTACCGTAATTTGGAATTTGGTCTGGACTTGGACACCCGGTTAGCTTTGGTGGGTCCCAACGGAGCTGGAAAATCCACACTTCTCAAACTCATTTTTGGTGATTTAATCCCCACGGAAGGCATGATTCGACGACACAACCACTTGAAATTCGGCCGATACCATCAACATTTGCACGAGCTCTTGGACATGGACATCAGTGCGATCGACTACATGATGAAGAACTTTCCCGAGTTAAAAGAACGCGATGACGTCCGAAGAATCCTTGGTCGATATGGCATCACGGGCAAACAACAGACCTGTGCTATCAAGCAGTTATCCGATGGTCAACGGTGTCGAGTGGTTTTTGCTTGGTTGGCTTGGCAAACCCCACACATGCTTCTTTTGGACGAACCGACCAATCATTTGGATATCGAGACGATTGACGCTTTAGCTGAGGCCATCAACAACTTTGAAGGCGGACTTGTCCTCGTTTCTCACGACTTCCGACTCATCAATCAGGTGGCTGACGAGATTTGGATCTGTGAGAAGCAAACGGTGACGAAATGGGAGACGGATATTCTCGAGTACAAGGAACATCTGAAGACCAAAGTTTTGGCCTCGATATACAAGGAAACCAAGAAGAGTTCGAGGCCCGTGTCCAAATCCAACGGATCCAGTTCGAGAGAGGCATGG**TAA**

>ABCF3

**ATG**GAGGCCAAAACTGAGGCCTGTGCTAAGCTTATCCGCGGCCAATTTCCCGTGATCGACTCCGACCTCTTTCAGTACATACAAGATGTGATCCAAATGTCAGGGGATGATTTTGAGCAAGATGAGGATGTGTTTGAAGCCGTGGGCGGATTTCTCCAGGAATGCGATGTCTCCAAATCCGAAGAAGATATCAAATCGTTATGTAACGGGATATTCGGGCTGTTGCATGGTGAATCCGGTAACAAATCCAATGGCAATTCCGGTCAACGCATCTTAGATGCTCCCGTTCAAATGAAGGACATTGGTGGAAATTCGGCCAATGGTCAAGAAGAACCCGCTGGAAGTATTTGGATCCAAAAAATTGAGGACAACTTGAGTGTGGACAAGAAAAAGTTGGAAAAGGCAGAGCAAATGTTGCTCAAGAAGATGGATCGCAAGGAGAAGGTCGAAAAACCCGCTGCCAATCGCTACAAGAGTCACGAAGCCACAGCCTCACAAGTGATCAGTAAAAAAGATGCCAAAGCCGAAGCCTCGAATCTCCAAACCAAGGACATTCGAATCGAGAATTTCGACATTTCCTTTGGCGATCACGTGCTTCTTCGCGGTGCCAATCTCGTTCTCACTTTTGGTCGACGATATGGAATGGTGGGTCGAAATGGAATGGGCAAGTCCACGCTTTTGAGAATGATCTCTAGTGGTCAATTGGTGATTCCATCGCATATATCCATACTACACGTGGAACAAGAAGTGACTGGAGACGACACCATAGCCCTTCAATCTGTTCTAGAAAGTTTAGAGAAGCGCGAATCTTTGCTTCGAGAAGAGAAAGAGATTAACCAAACCATCAATGACGGAGGCGATCCGGCCCTGTCATCCCGTTTGACGGAAATCTACGCCGAACTTGAAGCCATGGAATCGGACAAAGCTCCTTCCAAAGCGTCCGTGATTCTGGCGGGTTTGGGCTTCAACGCCGAGATGCAAGCACGGCCTACCAAGACCTTTTCTGGAGGCTGGAGAATGCGATTGGCCTTGGCCAGAGCCCTCTTTTGCAAGCCTGATCTGTTACTCTTGGACGAACCCACAAACATGTTGGATATGCAGGCCATTATCTGGTTAGAACGCTATCTTCAAACTTGGGGTACCACGTTACTAGTCGTCTCTCACGATCGGAATTTCTTGGACGAGGTCCCCACAGACATGCTCCATCTTCATTCCCAAAGAATTGACACTTACAGAGGCAACTACACAGAATTTAACAATACCATGACCGAGCGACTGAAGTCCCAACAACGCGAATATGAAGCCCAAATGGATTACCGGAAACACGTTCAAGAGTTCATTGACAAGTTCCGATTCAATGCCAAACGCGCGTCTTTGGTTCAATCGCGAATCAAACAATTGGAGAGGCTCCCTGAATTGAAACCCATAGAAAAAGAAACTGATGTGGTACTGAAGTTCCCGGATGTGGAGAAATTGTCACCCCCGATTCTCATGTTGAGTTCGGTTACATTTAATTATGACAAGAATCGGACCATTTTTTCGAACGTGGACCTCTCGGCCACCATGGAATCTAGGATATGTATCGTGGGAGAGAATGGAGCAGGAAAAACCACGCTGCTCAAGATCCTCATGGATCAGTTGACGCCCACTTCTGGAGATCGGACAGCCCATCGGAACCTCAAATTCGGCTACTTCTCCCAACATCACGTGGACCAATTGGACATGAGCATATGCTCTGTGGAACTCATGCAGAAAGAGTTTCCCGGTTTCAAGGTCGAAGAATACCGCCGAATGCTCGGAAGTTTTGGTGTGACTGGAGAAATGGCCTTACAACAGATCTCCAGTTTATCGGGAGGTCAGAAATCACGCGTGGCGTTTGCCATTTTATGCGCCCACAAACCGAATTTCCTGATTCTGGACGAGCCTACCAATCACTTGGACATCCAAACCATCGAAGCTTTGGGCAATGGGCTTCTCAAATATAAGGGCGGAGTTATTTTGGTCAGTCACGACGAACGCTTGATTCGGATGGTGTGCCAAGAGTTGTGGGTGTGTTCCAAAGGGCGTGTTTACTCGTTGGAGGGTGGATTCGACGAATACAGAGCCATCATCGAGAAGGAATTGGTTTTGGCC**TAA**

>ABCG 125057

**ATG**GAAAACAAGGGCTTCAGCGAAGTTCATTATGGGGGCAATGATGACGTCGTGGTCAAAATTAATCGTTCGTCCTCCATCGCAACGGCTGAAGAAGATCAGATCACATTCACTTGGGATGACCTCTCCGTGAGTGCTGTTTTTCCTGGATCAAAGATCCCATTCAAAAAGAGCGAAAAGAAAATCAAGCAGATCGTAAAGCAAGCCAGTGGCTATGTCAAACCAGGAGAATTGGTCGCAATCATGGGCGCTTCAGGGGCCGGAAAATCCACCCTTCTGAATGCCTTGACGTTCCGCAATCTCGATGGGCTCGATCTTACTGGTACACGTTACGCCAATGGTGTTCCCATTTCTCCCAATTCTTTGACCTCCGTGTCGGCGTACATTCAACAAGATGACCTTTTTATCGGCACACTCACTCCTCGTGAACATTTGGAGTTCCAAGCTTTGGTTCGGATGGATCGAGAAATCCCTTACAAAGCTCGAATGGCTCGTGTGGATAGTGTCCTACACGAATTAGGCCTTACCAAATGTGCCAAAACTGTGATCGGCGTTCCCGGACGAATCAAGGGGATTTCGGGCGGAGAGATGAAGCGATTGGCATTTGCCTCCGAGGTCTTGACCAATCCCGCTTTGCTATTCTGTGACGAGCCGACTTCAGGATTAGATTCTTATATGGCTCAAAATGTGGTCGAGGTTCTCCAACAATTGGCGAACCAAGGCAAATCCATCATTTGCACTATTCATCAGCCAAGTTCTCAGGTCTTTTCCATGTTTGATCGTGTCCTACTCATGGCTGAAGGCCAAGTGACTTACTTGGGAGGAGTTCGAAGGGCCTTCGAGTTCTTTTCAGACGTTGGTCATCCTTGCCCAGAACACTTCAACCCGGCAGATCATTATGTACAAGTTTTGGCAGTAACTCCTGGCGATGAAGAATCTTGTCGTCAACGAATTAAACGAATTGCAGACGCTTTTGAGACATCACCTGAAGGTCGAAGCGTGGTGAAAGAGGTGAACTATCAAAAGCAAAACAAGCACGCTTCCAATCAGATGGTTGAGGCTTCCAAGAAGTCCTCTCCTTACAAAGCATCATGGATTGAACAATTCAGGGCTCTTCTATGGCGAAGTTTTCTCTCGGTGATGAAAGAACCCATGATTGTCAAAGTGAACCTCATTCAAACAATCGTGATTTCGTTGATTTTGGGTATTATCTACATGAACCAAGAATATGACCAGAAAGGCGCTTCCAACATAAATGGTGCCTTATTCATCATTATCACAAACTTGAACTTTTCGAATGTCTTTGGTGTTGTCAATGTGTTCTGCTTGGAACTACCCATCTTTATGAGAGAGCATTTCAATGGCAGTTATCGAGTGGACACCTACTTCTTGACCAAACAATTGGCCGAATTGCCAGTCTACATAATCATGCCTATTGTTTTTGTCTCCATCTTTTATTGGCTTGTGGGGCTGAATCCGGATGGTGGAAGATTTGTCATTGCAATTGTTATTGCAATTTGTTTAGTTCAAGCCGTCATCAGCTTTGGATACTGCGTGTCTTGTTGGGCCTCTTCGGTGCAGATGGCTTTGGCCATTGCTCCACCAGTCATGATTCCAATGCTTCTCTTCGGCGGTTTCTTCTTGAATAGCGATACGGTGCCAGTTTGGTTGGATTGGATTCAATACATATCGTGGTTCAAATATGCCAACGAGGCCTTTGTGGTGAACCAATGGGTTGGAGTGGAACTTTCTTGTCCTCCCGATGAAATTGTGGAGCTTCCAGACACGTTTAATATCAGTTGTAATGCCCTGGGAGGCAATATTTCCAACTTTTGCAATAATTTCGCAGACAATGGTATCATCAGTATTCCTTTAAACGCATCGGCTACGATTGACGTTCCTTCGCCTTGTTTCTTTGACAGTGGCGAAGCTGTGATTGAAAATCTTGGTTTCGATAAGGACTCCAAAGTCTTTAACATTATTATGTTGGTTGTCCTCGCCATTGCTTTCCGTATCATTGGATTTATTGGTCTTTTGGTCAGGACCAGAAGGAAAACAAAA**TAA**

>ABCG 90506

**ATG**GAGAATTATGCCTTTGACCAAAATGAGATTCAAGATCACGAACCTCATGTACAACATAAAGCACTACCTCAGACTGCAACACCCCTGGCAATAAATCGAGGCAAAAAAGGTCGTACCACCTCATTAACTGTTCAAAACGAGCAAATTACATTTTCTTGGGAAGGCATCACCGTTCAAACTTCGAGTTCAAATAATCGGTGCTTTGGTTTTAAACGGCGACTTGTTTCAGAACCCAAGAAAATATTGAAAAATGTGAGCGGTTTTGTTAAACCTGGTCAGTTAGTAGCCATTATGGGAGCCTCAGGAGCCGGAAAATCAACCTTAATCAACTCTTTGACTTATCGCAATTTGAATGGATTGGAGATAATATCTGGAACTAGATATGCTAATGGGGTTGTAGTGAACCCAAACTCATTGACCGCTGTTTCTGGATATGTTCAACAAGATGATCTTTTCATTGGAACGTTATCAGTTCGAGAACAATTGGAGTTCCAAGCTTTGGTTCGAATGGAAAGTCAAATCCCCAAATCAACCAGGATGGAAATGGTTGATGCTGTAATCCAAGAACTAGGTCTAGAGAAGTGTCAGAATCAAATCATTGGAGTACCTGGAAAATTGGATGGAATCTCTGGGGGAGAAATGAAGCGATTGGCTTTTGCATGTGAAGTGTTGACGAATCCATCTCTGCTTTTTTGTGATGAACCCACAACTGGACTGGACTCATATATGGCCCAAAACGTGGTGCAAGTTCTCAGAGCTTTGGCTGCCAAAGGCAAAACAATTGTTTGCACCATTCATCAACCAAGTTCACAAGTATTTGCCTTATTTGATCGGATCCTTCTTATGGCAGAAGGACGTTTGGCCTACATTGGGGATTTGGCCAAGGCAGCCATGTTCTTCGACAGGATCGGTCTGCCTTGTCCAGATATGTACAATCCTGCCGATCATTACATTCACGTTTTGGCAGTAACTCCAGGAAAAGAGGATGAATGTCGGACAAAGGTCAAAGAGATTTGTGATCTATTTGAGAATTCACTTGAGGGGAAACATGTTTTCAACGAGGTTCAAAATCAAAAGAACAATGTTGGCTCAATTCCAAACCTTGGAAACGGTAATGAATCTGGAAAAGTCAGTCCATATCGAGCCTCATGGATGGAACAATTCAAGGCACTTCTGTGGAGAAGCTGGTTATCTGTTATCAAGAATCCCAGTGTGACCAAAGTTCGATTCGTTCAAACTTTACTGATCAGTTTGGTTTTGGGTTTGATTTATTTAAATCAAGAGTTGAGCCAAGAAGGAGTGATGAACATCAATGGAGCATTGTTCCTTCTTTTGACCAACATGACTTTTATGAACTTATTCACTGTTATGAACGTGTTTTCCATGGAGTTGCCAATATTCTTGAGGGAGCATTTCAATGGAATGTACAGAGTGGATGTTTATTACATTTGTAAACAATTGGCCGAACTTCCTGTATTTCTGGTCTTTCCCATATTGTTTGTCAGTATCTATTATTGGATGGTGGGACTCAATCCTTTGGCAGGACGATTTTTCGTAACCATGGCAATTGCTACACTTTTGGCTCAAGTGGTTATCAGTTTTGGATATTTCATCTCCTGTGTAACTCCAAGTTTGTCTGTTGCCCTTGCTTTGGCGCCTCCACTCACTATTCCAATTATGCTCTTTGGAGGGTTCTTCTTGAACTTTGAGGCTATTCCTAAATGGTTGGGATGGCTTCAATATTTATCGTGGTTCAAATACGGAAACGAGATATTGGTCATTAACCAGTGGAAAGGAGTCGCTCTGAATTGCACTGAACGTTGCAACTTCGAGACAGGTCAAGATGTAATCGATTTTTACAACTTTAACGAGGACAATTATGGATTTGATATCGCCATGTTGGTTGTCATGACAATCCTGTTTAGGATTCTGGGCTTTTTGGCTCTTTTAGCCCGGACCTACCAAAAACAAATA**TAA**

>ABCG 75152

**ATG**GATGAAGTCAATTTTTCATGGAAAGATCTCACCGTGATCACGGACTCGGGATTAATGTGTCGGAGGCGAGCTCGACCGGTCCTCAAAAATGTGTCTGGCTATGCCAACAAAGGGGAGCTTTTGGCCATTATGGGGCCATCTTCAGCCGGAAAGAGTACCCTTCTCAATGCCCTAGCCTTCCAGAACCTGAACGGATTGCAGATCACGTCAGGAGAGCGATACGCCAATGGCATGCTCATGAATCCCACGGGAATGACCTCTATCTCGGGATACATTCCTCAGTTTGATCTCTTCATTGGGACACTCACTGTCAGAGAACACCTCACCTTTTTGTCAAACTTAAGAATTGGTGGCCAAGTAAGTCAAGAGGACAAACGTATCAGAGTGGAAACGGTTATGAACGAATTAGGACTGACAAAATGTGCTGATGTACTGATTGGTGTCCGAGGTCGGGTCAAGGGCATTTCGGGTGGTGAAATGCGCCGATTGGCATTTGCCTCCGAGGTCTTGGCTGATACACCTCTTCTTCTTTGTGATGAGCCCACATCAGGATTAGATTCTTGGATGGCCGAAAACGTGGTCGAGCAAATGAGGATCATGTGCGACAAGGGCAAGACGATCATTTGCGTGATTCATCAACCCAGTTCTGACGTCTTCACCATGTTTGACCGGGTCTTGTTAATGGTGGAGGGTCGAAACGCTTTTATGGGAACGATCAAGGAGGCCAACGACTTCTTTGACAAAATTGGTTATCCATGTCCTTCGAATTTCAATCCGGCTGACCACTTTATCTACAAGTTGTCCATGCAGCCCAAACTTGAGGATGAGTATTTGGGAAGAGTACAGTACATTTGTGATGAGTATGAGAACTCGAGATACGGAAAGTTTGTCTCGGCTGAGATTGAAAACCATATCGAAAACAGAGAAGAGCTCTTTCATAACGTTGGGTTCAATTTTTCCGAGCTTAAACCGTACAAATCCCCTTGGGACCAACAATTTCGAGCGGTTTTGTGGAGATCCTGGCATTCGGTTACTAAAGAACCTCTCATTGCTAAGATCCGTATCCTGGAAGTTATTGTTTTGGCGCTCATTGTGGGAGCCATATTCTTTGGACAAGAAAATAATCAAGAAGGAGTGATCAGTATCAATGGCGCTCTGTTTTGGGTCACCATGAACCAGACATTTTCCAATTATTCAAGTGTGCTTAATGTGTTCTGTAATGAAATACCTGTTTTCATACGAGAACATTTCTCCGGAATGTATAGAACTGATGTGTACTTTTTAGCAAAGCAAATAGCTGATTTGCCATTATTCGTTATCACCCCTGTACTCTTCATGGCCATCTACTACTACATGGTCAATTTGAACAATGAGATTGACCGATTTCTCATGGCGATTCTCATCAACGTGATGATTGTTCAGACTGCAAGTGCTATTGGGATCTTCATGTCTTGCATCTGTCCAAATTTGCCTGTCGCATTAGCAATTGGACCCACCATTTTTATTCCCTTGATGAACTTCGGCGGATTTTTCCAAAATGAAGGAACAATCCCAAGTTATCTGATTTGGATACGATACATCAGTTTGTATTATTATGGCAATGAGGCATTCAACATTAATCAATGGTCAGGAGTAACGAACATCACTTGCGATTCCGATCGACACGAAAGTCGACTTTTTAAACCGTTGACGGCCATTATTGATCCCTATCACGCTATTCGAAGAGATCCAGCCGCTTGCGCAGAGACTGGAGAAGAAGTTTTGGACTTTTACCATTTCCACAAAAACAATCTCTGGTTTGACATCATAATGCTGGCAATTTTATCGTTGAGCTTTCGAATCTTGGCCTTTTTGGCGTTGCTGAACAAGGCACGAAGACGAACGAAA**TAA**

>ABCG 105304

**ATG**GCCATGAATCCTTCCGAAGCCCAACCCTTCATGGCCCCGCCTCCCCCTACGGGCTTAACCTACTCATGGCAAAATCTCCGGGCTCAAGTTCCAATCAGTCGGCAATCCGGATTCGCGAGTTGTTTTACGCAATGTCCGCCTCGCTGCGGCCGGAATAGAGAGCCCATTGTGGATCAAGGAAGCGAGGCCTTGAACGGTGGCCTCGTTAGAGACGACCAAGCGTCCAAGGTGGTCTTGGATGATGTGAGCGGGATTGTGGAGAAGGGCGATTTGTGCGCCATCTTGGGGGCTTCGGGGGCGGGCAAATCCACCCTGCTCAATGCGCTCACCATGAGAAATGTTGATGCTTTAGAGATCAGCGGTGAACGATACTGCAATTCCGAGTTAGTCACGCCAGAGTCATTGACGGCGCAATCGGCTTACGTTCAACAAGACGACATGTTCATCGGTACCTTGACCGTTCGGGAACATCTCCAGTTCCAAGCGCGAGTTCGCATGGATCGACATTTCAATGCCAAAGAGAGAATGGATCGCGTCCAAGAAGTCATTGCTCATTTTGGCTTGGATAATTGTGCTGATGTTCTCATCGGAGTACAAGGAAGAATCAAGGGGATCTCCGGAGGCGAATCCAAACGGTTAGCCTTGGCTTCTGAAGTTTTAACTGATCCTCTATTGATGTTCTGTGATGAGCCCACCTCCGGATTGGACTCGTACATGGCGTTGACCGTGGTCGAGACCCTAAAGAACATGGCCACGGAAGGCAAAACTGTGATAATCACCATTCATCAACCGTCATCACAAATCTTTGAAATGTTCAACAAGATTCTTCTGCTAGCCAATGGACGAGTGGCTTTTCATGGGAGTCGCGAGGATGCCGAAGGATTCTTCCGATCCATGGACATGATTGTTCCTCAGTATTTCAATCCAGCCGATTTCTACGTCCAAAAATTGGCCATGGTGGCGGGTAAACGAGATGAGTCTCAGTCTCAAATTCAAAAGATCTGCGATACGTTCCAATCAAGCATGTGGGGCTCCCAATTACGCGATAAACTGGCTAAGAATGCTAGTCCAGAAGATTCCGCTTCGACCAAAGTCTCGATCAGAGCTTCCGTTTCCAAGTACAAAGTCAGTTGGTGGCGACAGTTTTCGGCCCTCATGACCCGAACCTACTTGGCCACTATCAAAGAGCCCTTAATTGCCAAAGTCAAAATCATTTCGACCCTCGTCGTCTCCCTTGTTTTGGGTTTCATCTACTATGGGCAAAAGCATGACCAAGCCGGAATCATGAACATTAACGGTGCCATCTTCATCATCATCACGAATCTCTCATTCGAAAACATCTTCGCCGTCATCATGGTGTTCTGCAATGAACTGCCCATCTTTCTTCGTGAGCACGCCAATGGAATGTATCGAACCGATGTCTACCTCATTTGCAAGCAACTCGTTGAAACTCCCGTCTTCATCATCACCACGACTATCACGGTGGTAATTCTCTATTTGATGACTGGGATGAATCCGGATCCGCTTCGCTTTCTCGGTGCTCTGGGAATTGCCCTCCTTCTGACTCAAGTTATCGTATCGTTTGGGTACATGATCTCTTGCATCGTTCCCAATGCTCAAGTGGGCAATGACATCTCTCCCGTGCTGATTTTGCCGTTTATGTTGGTGGGAGGGTTCTTCCTCAATAATCTCTCTATCCCCACATGGCTCATCTGGCTCAAGTACATCTCTTGGTTCAAATATGCCAACGAGTGTCTAGTCATCAACCAATGGAGTGGTGTGGAAGAGATCGATTGTGATGAGGGCGTGGATTGGGGATGTATTCACACGGGAGACGAAGTGATTGCCAGATTGGGATATGACAAGGCCAATATGACCTTTAACCTCGTTATGATGGTAGTATTTTCTTTGGTCTATCGACTCATTGGATTTGTCGTGCTGTGTATCAAAAGCCGACGTCGCAAC**TGA**

>ABCG1

**ATG**TCGGGAGGAAATCATCCAAACTTGGCAACAACACCGCAGCCTCCAGCAGGCCAACACAGTGGAGAGGACCTTCAAAAAGATGTTGTTCACCCTCACCAAGAACATGACGGGCATCTACCGCTTGCCTCTGATCACGATGAGCCCGGCTCGTCGCCTGCAAAAACTCTCGGAATGGTCATGATCAGTGAGACAAACTTGAAAGAGAAACAGCTCAGTTCTGAAACTAGCACTCAACCAAAGAGCGTTCATGAAGTTAATGGGGTCGTTGCATACAACTCCGAAGGTGTAATTCTAACTGGAGGAGGGTCGAGATCACGGTCTAATCAAAATCCGGAGCGCAAGATGATGACCAATCTGGCCAAACGTCCCCCCGTGGACATCGAGTTCAAGGATTTGGTCTATTCGGTACCGGAGAAACACGGGTTGATCATGAAGACCTTTGCTGGGCGGAAGACAATCCTCAAAGGCGTGAGTGGCAAGTTGAGATCAGGCGAGTTGATTGCCATCATGGGACCTTCAGGAGCTGGTAAGAGTACTCTCATGAATATCATGGCCGGATATCGGACCTCCAATGTGTCGGGTGACATATCTATCAACGGGAAAACCCGGAATTTGAGGAAGTTTCGGAAAATGTCTTGCTACATAATGCAGGATGATTGTCTGTCTCCACATTTGACTGTCAAAGAAGCCATGGAAATTGCTACAAATCTGAAGCTAGGCGAGGAAACCCCGCTTGAAACAAAGGCTCTTGTTATCAAGGAAATAACTGACAACCTTGGTCTCACGGAGTGCATGGAAACAAGGACAAATATGATCAGCGGAGGTCAAAGAAAACGACTCGCCATTGGTCTCGAATTGGTCAACAATCCACCTGTGATGTTTCTCGATGAACCCACTTCAGGCTTGGACTCGGCCTCTTGTTCTCAATGCATATCACTACTTAAACAATTAGCACGGGAGGGTCGCACCATCATTTGCACCATTCATCAACCATCAGCCAGAATCTTTGAAATGTTTGATAAACTCTACATTCTTGCGGAAGGTCAGAATATCTACAAGGGAACCGTGAAAGGATTGGTTCCATTTCTTTCCTCCATGGGGCTAGATTGTCCCAGCTATCACAATCCCGCTGATTTTGTCATGGAAATAGCCACGGGTGAATATGGGAACTACACACAAACACTAGTGGAAGCCGTGAATGCGGGGAAATGTACAAACTTGGGTGATGATTCGCCTTCGCCAAGCGCCAAATCTAGTCAGGATATTGGTGAAGGTGCTCGGAATGGGAAAGTGAAATCCATTCCGGAAAACCTCCCAACCAAATCATCTTCAGGAACGCTTCAGATATCCATGCCAGAAGACGAACCAAACACCACCAACGGAACCGTTGGCAATGGCGAACTCCAAGCTTCACTTTTGGGTGATTCGGACGAGAGCATGGGCAAACATGCTCATACATTTCCAACCACTGCTTGGACTCAATTCCGGATTTTAGTGGTTCGGACTTTCAAGTGCATCATTCGGGACGAGACGCTGACTAAGCTGCGATTCATATCCCACGTGGCCATTGGAATTCTCTTAGGACTGCTTTACTGGGATATCGGAAACGAGGCCAGTAAGGTCTATAACAATAGTGCCATGCTGTTCTTCTGCATGTTGTTCAGTATGTTCACCGCCATGATGCCCACTGTGATGACATTTCCTCTCGAGATGGCTACCTTCAAAAGAGAACACTTGAACTACTGGTACAGCATGAAGTCTTACTACCTGGCCAAGCTCGCCGCAGACTTGCCGTTTCAGATTGCATTTCCTTCGATCTTTCTTCTCATTGTCTACTTCATGACCTCACAGCCTCTGGAGCTCACCAGGTTAGGCATGCTGCTCCTCATGTGCATACTCACATCTCTGGTGGCTCAGAGCTTAGGTCTAGTGATTGGATGTGCGGTGGACATGCAATCTGCCGTTTATCTGGGACCAATCACCACCATTCCCATACTCTTGTTTTCTGGGTTCTTCGTCTCCTTGAATAACATTCCCATCTTCCTACAATGGTTGGCCTACGTGGCCTATGTTCGTTATGGTTTTGAAGGCACGATCTTGGCCATCTACGGGTTCCAACGGGAAACTCTTAAATGCTCGATTCCTTATTGCCACTTCAAGTATCCGAAAAAGTTCCTGGAGGAACTTGCCATGGATGAAGGAGTATTCTGGATAGACGTCGTTAGTTTGTTGGCCTTCTTTGTACTTCTTCGAGTCTTGGGATACTTTGTACTCAAGTTCCGGATTCAATTGGAGAAG**TAG**

>ABCG5 isoform X1

**ATG**AAAACCATGGCGAGTGATTATGTTTTAGAGGTGGCAAATGTGTTTCACTCGGGTCAAGTTGAAAAAGGAACCCAAATCCAAAAGCTTATCGGTGCTATCCGAACGGGGGTAATCTTAAAAGATGTTTCAATGTCCTCATTTGGTGGAGAATTATCGGCGGTTCTGGGATCGAAAGGAAGTGGAAAACGAGCGTTTCTAGATGTGATTGCAAGAAGAGCACAAGGCCCAACCAGGGGACAAATCATGCTGAATGGCGTGCCCATGTCTATGCGTCTTTTTCAAGATAGTTGCGGTTTTGTCGGAAAGTCGCCGGATCTTCTTTATGGGCTCACAGTCAGGCAAACGCTTTTATTTGCAATTGAACTTTCGCTGGGATCAAAGATTTCAAAGGACGTGAAGTCCTCCAGGGTTAAACAAATCCTGGCAGACTTTGCATTAACGGATTTACGGAATAGACAAGTTGAAGATCTGAATAAAAGCGAATCTCGACGTCTGGCAATCGCCAAAAACCTTGTGCGAGATCCTGTACTACTTCTTTTAGATGAGCCAACGAACGATCTTAATCCTTTGGATGCCTATTTTGTCATTTCAATACTGGCCAATCATGCCAAGCAAAACAATCGAATCATCATATTGACCATTGAGAAGCCAAGATCTGACATATTCCCCTTTTTGGACAGAGCAACCTACCTTTGTCTTGGCGATGTTGTTTACACCGGTCCCACCAGAATGATGCTGGAATATTTCCGCTCTATCGGGTTTCCGTGTCCCGAGCTGGAAAATCCTCTAATGTATTATTTATGTCTGTCGACGGTTGATAGACGCTCAAGGGAGAGGTTTGTAGAATCGAACAATCAAATTGCTTCACTTGTCGAAAAATTTAAAGTAGAAGGAGTCCATTACCAACATCTCTCAAACACCGCAACCACAGATATAAAAACAAAAATTCCTTTGACTGCCTTTGGAAGACCATCCGGCCCTGTGAAAATCTGGGTTTTGTTAAGGAGGTCGCTAAGTGGATTTTTCCGTGGTGGAGCCAAAGGAATAATCTGGACTCTATTTCATCTCCTTTTTTTGAACGTACTTTTTTTCTTCATCTGGGGGATGTATTACAACTCAATGACCAGACCAGTGCAATCACAAACAATCTTTTCAACCAAACGTGGCCTTATTTTCAATACATGCTTGGCAACACATTTGGCGGCCACTGTAACGTCTGTCCTCCAAGTTGCCCCAGCAAGAACTGCCTATTATACCGAATATAGACAAGGTCTTTACAACGGACCTTCCTTTGTTGTTGCTCAAACGCTTCGAAGTATTCCGCTATCCATCTTTGTCACATTTACAGGATCTCTCTTACTCTATAAAGGGTTACGACATGAGGATTGTCTTACATCAAATTGTACCCCCGTGTCTGGATACCAGTATTTCTTCCCTTATTGGTTTACCATTTGGGTCTGCTATATGTTTTCCGAGCAACAGGTTCTAGGTCTTTTACTCGCTGTGAAATCGCAAGTTGTTACTGCAGTTATCGCCTTGTTCATGTCAACGACAGCATTGGCAGTTTGGAGTGGCACAACAAGGTCTTTTTTCGGCCTTCCAAATATATTGACTTATATGTCGTATGGATCCCACGCCCGATACGTTGGAATTACGCTCAACAGCATTGAATTTGTCCACTCGTTAAATCTGAACAATGTTGGTTGGCAAGATGATGAGGGACGAATGTTTCCTTGTAATGGCAACACATTTGGATTTGGTTGCAGATATATCAATGGAACACATTATCTTCAAGAAACTTATGGATTGTTAACGGATAATTTGGACGATTTGTTGAATGTGTGGAATAATGCACTCATATCTGCATCATTTTTCTTGGGAATGTTTGGCCTCAATTTGCTACTTTATGTACTTCCAATTCCATTATTCATAAAATTGAAATTTCGAGAGGAAACAGCAGAGGCT**TAA**

>ABCG5 isoform X2

**ATG**AGTTCTAGAAAAGAACAAATAAAAAAGATCCCGAGGGAAAGCAGTGATGGCGGAATCAAAACATCGGAAGACCTTCACGCTTGGTCCATCTACAGACAGAACTTGAATTCTGACTTCACAGATTCGGCATTAGGTTCATGTGAGAAATCCCCAATGCCGTACGGTAATTTCCACTTAAGAGAAACGACAATGCAAAGTATACTCAGTAATCCCAAATATGGGCCCAAGTCTGAACTTGGATCGAATGTCTACACGTACCTCAAATTTGGGTTGCCCAGAGTGCTTCCCCCCAATGTGTATCCTGTCCAAGATAATTCAAGTGGCTATGATTCCACGGACGAAGAAAGAGACAGAAGGGCCATCCATGTTCGAGGATCTCACAGTGTGGATAATCTTAATAGTGTTTCAAAGTCAAAATTTGACGCTTTCTACCCATCGAATCAGTTAGGTGACTTTAAGCAATCAAGATTAGCGAAAAGTGAACTGAATTTGGCTCGCCAAAAATCAAAATACTGGATCCCAGAGGTTGCGTCTCATGGAAGTGACACTCCCTTAACAAAAAGCAAACAAGACAGGAAAACTTCTGAAATGGGCATGAATTCGATTCCAACAGCGAGTATTGAGGCTGAATTGGCCCAATACCCTCATCTGCAACTCAGAGATTGCATGTTGGTCGAAAAAGCTTCGAAAGTTCGTTCTTTAGAAGGCCTTACTTTGGAGGCTCATGGTGGAGAACTGGTTGGAGTTTTAGCAACAAATGCTGAAAATGGGTCTTTGTTTTGTGATCTTATAACTGGAACCTACAACTCAAAGAAGTATTACAGCGAGGGCCAAATTTTGCTAAATGGACATCGAATCACCAGGTCGCGACTCAAATCAAGGGTGTCATACGCTAGGTCCAGTCATTGCTTTCCAGAAAACCTTTCAGTTCGTCAAGTTATGCTCTTCCGAGCTTATTTACAAGAATCTGAAGACAATGTGCGGAACAAAGACGTGAAGGGCCGGATTGAAGCTTTGATTGAAGACCTTGGGTTGGCTCAAGTAAAGCATGCCAGGATAAAGAACATCACAGCCTCAGAAAAGCGGCGTTTAAATTTAGCTGTACACTTGCTTCTGGACGCGGATATCACAGTTATTGATCAACCCACCAACGGAATGGACATCTTCGACACCTTTTTCCTTGTTGAATATCTTCGACAATGGGCATCCAGGGGTCGCTTAATCATAATAACTATCCATCCACCAACTTACGAAATATTGACCATGATTTCAAAAGTTGTGTTGGTCTCCATGGGTCGAATTATATACTGTGGCAAAAGACGAGAGATGCTCCCCTATTTTGCCTTCATTGACTATCCTTGCCCAGCTTATAAGAATCCCTCTGACTATTATCTTGATCTAGTTACCCTCGATGACTTGTCTCAAGATGCTATTTTGGAGTCAAGACAAAGAATAGTGCATTTGGCAACGACCTTTAACTTGAAGGCCGAACCCTTGTCAGATCCTGGACCACCTGGAACGTTGCCCCCAAAGTTCAAAACAGCTCAATGGATCATTCAAGTCATGGCGTTGATTGTGCGTGACGTTGGGTATTTATATCCATACAATATAATATTTTGGGCAAAGACTTGGCTTCTCGCTGGAACAGTCTCCTTATTGTTCGGATTATTTTTCATTGGTATTCGTTGGCAGTATTGGAACTCCTCATGGGTCGAGGATCCTTCATTTGAACAAGACAACATAGTCAACAGATTTGGATTGATCCACATTTTACTCTGTTTTGCTCCGTGGCCGGTTCTTTGCTCAATAGTTCAAATTCAGGGAAATGAAAGAGCTGTTTTACAAAGCGAATTACATGAGCGACTCTACGCAAATATTTGTTTCCATCTGGTTAAGAACCTTTTGGAATTCTTACCAGTTTGTTTGACTTATGCTGCGTACATTATTCCTGGTCTTGCATTGGTTGGTGTTCATCAGGATATTGATTATCATGTGCTGGGGATTATTATTCTTCATCTGGAATGCTGGAGACAGTTTCTGGAGTTTTTGCTTATTGCCTTCGACAATAAGATCTTCGTATTAATATCGTCATCCGTCGTGGGATTCCTCGCCAATTTGACCACAACGTTTTCAATTCATCCCAAAGATTCCCCAAAATGGGCACAAGGCCTTAGTCTTGGGTTTCCAAACTTTTGGGCATTTAGATCAATAATAAGAACTGAATTCCAATCAATTGACGTTCTGAGATGTTCCACCAATCCAGTCATTACAGAAAACTCCATTATTAAGCAAGTGCCTTGTGGGTTACCTGATGGCCAAGGAGTCATCAAATATTTTGAGCTCAACGAAGACAGTACATCTGGTTTACCACTTTCGGATTCCATATTAAGTCTTTGTATTATGTATTTTGTGTTAAAAATGCTAAACGTTTTAGGATCTATCCTCTTTCAAACACATCGGAAA**TGA**

>ABCH 139282

**ATG**CAAATCACCACGGGACAGAATGGCAACGAGATCAAAATCGAAAGTTCAATCGTGAAGGTTGTGAACGCGCAGAAATCTTACGGAAAGGGCATCCAAAAGAAGGTTGTATTGGACAACCTTTGTCTCACCGTGCCTAAAGGGTCCATATATGGACTGCTGGGGGCCAGTGGGTGTGGCAAAACCACTCTATTGTCGTGCATTGTCGGTCGCAAAAATCTGGATCATGGACGTCTTAGTGTTTTTGGAGGTGTGCCTGGAGATCGACGAACTGGAATTCCCGGAAAAAGAGTGGGATACATGCCACAGGAATTGGCCCTTTACATCGAATTCACCATCAAGGAGATGCTCGAGTATTTCGGACGCATTTACGGCATGGACAGACACCAACTCCAGGAAAAGATAGATTTTCTCGTGCAATTCTTGGACTTGCCACATTTTGATCGTGTCATTAGAACTCTGAGTGGTGGCCAACAACGTCGGGTTTCCTTTGCCGTCTCCTTATTACATGACCCAGAGCTGCTTATACTCGATGAACCCACAGTGGGAGTGGATCCGATGCTGAGACAAAGCATCTGGAATCACTTAATTCGCTTGGCCAAAGAAGAAGGGAAAACCATTATCATCACCACTCACTATATCGAAGAAGCTCGACAAGCTTCCATGGTGGGTTTGATGAGAAATGGGCATCTCCTGGCTGAAGCTCCACCTTCAACCCTCCTCGAGCAATTTAACCTTCCATCTTTGGAGGACGTTTTCCTCAAGTTGTGTGTGAAACACGAGATCAATGACAGCCATGATATTCCCACCAAGATCCCAACCGGGACTAGGTCGCTTTCCGGCACATTGAGAAAGTTGCCTCTGGTCTCAGATCTTACTTTGACCAACACAGCCAAGAGACAGAAAAGGGGCGGAAGTCTAGTTTATCCGGGGAGTACGGCGATGGCCGAGGACTACAGCATTCAAGCCATCTCTTACATGAAAGCCCCGGAGGAGACGGAATTCCAGAAGATTAAGCGCAGACTTTCAATGAAGAAGAAGCCTAATTCTGGTTACAACGACCAAGTCTCTCCAGGATGTTGCCAGATGACGGTCCCTTCTCCGTCGAAGATCTGGGCGTTGATCATGAAAAACCTTATTAAGATGTGGAGGAACATGGCGCAACTTTTGTTTTTGTTCTTGTTGCCCGCTGTTCAAGTGATTTTCTTCTGCGTGGCCATTGGACAAGATCCTATCGACCTCAAATTTGCAGTGGTCAATCATGAAGTCGCAAACCTGACCTCAAATTGCACGGTGACCACAGGCTGTGATTTCACCACCATATCATGTCGATTACTCGAGTCAATATTCACTAACTCGTCCACACTCGTTATGGTGCCTTTCGAAACCGATGAGTTGGCGCGAGAAGCCGTTGCCAAAGGAGAAGTCTGGGGAATGGCTATCGTTCCCGAGACCTTCACCAAATACTTCCTTAAACGATTGTGGTCGTCATTGGATGTGGACAATGAAACTTTGGTCCAAAGTTCGATTCAGGTCAATTTGGACATGTCTAATCAACAAGTGGCTTTCCAACTTCAAAAGGCTCTTCACGAAGGCTATCAAAGCTTTTTCCGACAATTGCTCACCGATTGCGAATTTCCTCCCGAAATGGGAGACCTACCTCTAGTCTTCAAGAACCCCGTATACGGGGATCTGCATCCCAATTTCACCGAATTCATGGCTCCTGGAATCATCATCTTGATCATCTTCTTCTTGGCCTTATCCCTCACTGGTGATATATTTGTCACCGAAAGACGTGACGGACTTTTGGATCGGTCTTGGATTGCTGGAGTTCTTCCTTCGGAAATCCTGATTTCCCATGTCTTCACGCAGTTTCTCGTATTGGCCGGTCAAACTGCCATCACTCTTGTCTTTATCTTCTTGGTCTTCAATATCAAATGTGAAGGACCTTTAGGCTGGATTGTGGGGCTAACTCTCCTTCAAGGAACGGCAGGAATGTGTTACGGCCTTCTTTTGTCGACGATATTCAACGACGTTGCCGCGACCATTCAGTTCTCCATTGGAAGTTTCTACCCATGTCTTCTTTTAAGTGGTATTTTGTGGCCATTGGAAGGGATGCCTTGGTACCTGAAGACCATTTCGTGGTACTTACCCTGCACAGCGGCCTGTCAAGCCATGAGAGATGTCATGTCCAGAGGATGGGGCATCATGCATCCTTCCGTGTATTTGGGATATGTTTCCACTTTGTCATGGATTTCTGTTTTCATTATTCTCAGTTGGATTGTGATCAAAGTGAAATCTTCG**TAG**

>ABCH 88708

**ATG**CCCTCTGATAAGAAGCGCGATAATATCGAGGGTAATTGCTTCTCTGAGGCACACCAGCATACTCAAAACTCTTTGACTCACGATCAAAAGATCGAAGAAGAGACATGCTTGGTGGAAAAGAGCCCTCAAGTGATCTCACCTAAGCGAAAAAGTCCAAAGAAGACCAAGATGGTGTCTGATCCAAACACAGCCATCTCCTTCGAGAATGCCTACAAACAATACGGGAGAGGCTCAAGAAAAGTGCCCGTTCTCTGCGGTCTCAACATGACCATTCCAAAAGGTGCCATCTACGGGTTATTGGGTCCAAGTGGATGTGGCAAGACCACTCTGCTTTCTTGCATTGTGGGCAAGCAACGATTAAAATCAGGGTCAGTGACGGTGTTTGGCGGTAAACCGGGGTCAGCTGAATCTGGTGTGCCCGGAACACGTGCTGGGTACATGCCTCAAGAACTAGCTCTCTATGGGGAGTTTACGATTGCCGAAACAATGCAATACTTTGGGCGGGTCTATGATCTCACCGCTGCCAAGATTCGCGAGCGGACAGAGTTCCTCTTGAATTTTCTGGACTTGCCCAAAGAGAGACGCTTGATCATGAATCTATCAGGAGGTCAACAAAGACGTGCCTCTCTGGCTGCAGCTTTACTTCACGAACCCGAGCTCCTGATCTTGGACGAGCCCACCGTGGGTGTGGATCCACTTCTTCGTCAATGTATTTGGACCCATCTCTTGGAGATTTCCAAAGCTAGTCACTTACAAACCACCATAGTCATAACCACTCACTACATAGAAGAAGCCCGACAAGCTAACGTAGTAGGGATGATGCGGTTCGGAAAGCTCTTGGCTGAAGGATCCCCCGCCAAGTTGTTGGAACTTTATCACATGCCAAATTTAGAAGATGTCTTCCTTCATTTATGTCTAGGGGACGAGGAACAACGCGAGACCGAAAATGCAAACAAACTTCAGGATATCGAATCCCCCAAGAGTGTGGCCAGTCCGGTCAAAGTCGACAACGATTTCCATGTTCCCGAAGTTCAGGTGGAAACGGAAGGAAGGGAACCAAGACAAGAGCGTGGTCAAACAAACAATTGGTGCACATTCCTGACCCCTCATCGAATGCGAGCTCTCTTAGCCAAGAACTTCATTCGTATGTGGAGAAACATTGGCTTCCTTATCTTCCAATTTATCATACCCACAGTTCAAGTGGCCCTCTTTTGTCTAGCCATTGGAAGGGACCCACGTGATCTCACTCTAGCTGTTGTCAACGAAGATGTACCTTCCATGAGGTGTCCACGATTTACAACAGGTTGTATCCTAGGTGAAAAAGACGACTTTTTGGGAGCGTACGACTTCAATATGGAACACAGAGCTAACTTGAGTTGTCGTTATTTGTCCTATATCGACGAGGCAATCATACGACCAAAGTTCTTTGACTCCTTGGAAGAGGCCGAACTTGCAGCCAAGAGAGGTCAACATTGGGGTGTAATGCACTTTAAACAAAACTACACACTAGCTCTCTTCGAAAGGTTATTTGGCCTTGCTCTTGCCAATCCTGAAAAGTTGACCAACGAGACCTTGGATCTCAGTGAAATCCACGTCAGTCTGGATATGACGAACCAACAAGTGGGGTATACCATCCAATTGAGATTAAGTGAAGCATACGAGAAGTTCGCGCAGAGCCTCCTCAACTCTTGCAACTTTCCAAAAGAACTGGCATCGTTGCCAATCGCCTTTGAAGAGCCTATTTATGGGTCCAATGAACCCACTTTCACCGAATTTATGGCCCCTGGTGTGATCCTCAGCATTACTTATTTCATGGCAGTGGGACTGACGGCGTTATCATTCATCATTGAGCGAAAAGAGGGCCTTTTAGATCGAAGTTGGGTGGCTGGTGTCACGGCCACGGAAGTGATGTTGGCCCACGTGGTGGCCCAGTTTGTCGTGATGGTCGTCCAAGTGGCCTTGGTACTTGTCTTCATGATCTACGTGTTTAACGTTCCGGCTGAAGGACCTTTAATATGGATCATTCTTTTGACCATCTTCCAAGGGATTTGCGGCATGTCCTTCGGTTTGGTCATCAGTGCAGTTTGCGACAACGAACAGGATGCCATTCAACTTGCATTGGGATCATTTTATCCCAATTTGTTACTGAGTGGAATCATTTGGCCTTTGGAAGGTATGCCCGAAGGACTCAGGTATTTGAGCTACATTTTACCCCAAACTTATGCATGCGAGGCCATGCGGGGTATCTTGTCCCGAGGCTGGGGTATCGAATGGATGCAAGTATATCGAGGTTACTTGGTCACGTTTGCGTGGACAATTTTCATGCTCATTCTGAGTGCTGTACTATTAAAGATCCGACGA**TGA**

>ABCH 103004

**ATG**GAGGACCAAACGGAACAAGAACAAGCTTTAGTGAATGGAAACAGTGTTGCGACAAGTGCGAACGAATCAAAACTACCCAATGGCCAAACCAAAGAAGGCCCAGTGGCCATCAAGATCTCAAATGCATATAAACATTACACCTCTGGCCAGAAACGCACACCTGTCCTTTTGGGATTGGACATGGAAGTTAAGAAGGGGCAAATCTACGGACTTTTGGGTCCATCAGGGTGTGGGAAGACCACCGTGTTAAAATGTATTGTGGGCAAATTGACCGTGGAAACGGGGTCGGTGCGAGTTTTTGGGGAGCCTCCAGGGACGCCAGCCTCCGGGGTCCCTGGACCTCGTGTTGGTTACATGCCTCAAGAATTGGCCATCTTTCTCGAGTTCAACATCGAAGAGACACTTACCTATTTCCGACGTATTTTTGGCATGACGCGGAAGCAATACGAGGATCGGCTCCAATTCCTCATGAAATTCCTGGACCTGCCTTCAAAAACACGATCTTTAATTAATATGAGCGGCGGTCAACAGAGACGGGCATCCCTGGCCATTGCATTGCTCCACGAACCGGAATTACTTATTCTTGACGAACCAACGGTTGGTGTAGATCCCTTGCTGAGAAGTGCAATATGGAACCATTTAACCGAGATGACGGATTGTTTTGGGAAACATTGCACAATTGTGATCACGACGCATTATATTGAAGAAGCTCGACAAGCCAATCGTGTTGGAATGATGCGTTTTGGTAAGCTTTTGGCTGAGGATTCTCCTGAAGAGCTATTACGCAGATTCAGCAAGCCCAATCTCGAGGATGTCTTTCTCGACCTCTGTCTCCAGGATGGGGATCTTGAGACGGCCAAAGTTCGAGAAGAGCGTACTCAACGCCGATCCATCTTGAAATTCTCTAAGAAAAAGTCAGAGGCCGCCAAACCTGATCTGGAGAACGGACAGGGTTGCCAAATGGTCGTCACGAATGGCAAGGGCCCAAAACCCAAGGAAAAAGTGTCCGCCACAAACAGTACGGCCAGTGCCCGCTCCGTTTCGTCCACGGTCTCGAAGTACATTGATCAAAAGAAATCAAATCTGACCTCGCAAAGAGTCACGGCGTGTATGATCAAAACTTTCAATCGGATGAAGAAGCGCGTCGGACTCCTTGTTTACCAATTCATTTTACCCGCGCTTCAAGTGTCGCTGTTTTGTCTGGCTATCGGCCAGGACCCCAAAGAACTGCCAGTGGCCGTAGTGAACCTAGAAAACCAGGGCCAGCCCTGTGCCGGTCACCCAACTGAGTGTCCGTTTGACATGACATTTATTGGCCCAGAAATTAATGACAATTTACAGAATCTATCCTGCCGCTACCTTAGCTTCATGGACGCGTCCATAGCCAGACCAGTGTATTATGAGAACTATGAGGATGCTCACCAGGCCGTGAAGAATGGTGAGGCATGGGGCGTGATAAGTATGGGTCAGAATTTCTCGAGGAATCTGTATGATCGAATCTTCGAATCCGTGGCCAGCGAGGACTTGACCAAGGTCAATTTGGACAAATTCAATGCCAGTCAGATCCAAGTTGAGATGGATATCACAAATCAACACATTGCATTCACACTCCAGTTGAAGTTTGCCGAGGCATTTCAACAATTCATGGAACAATTGGTGACTTCTTGCGGCATCAATAAGGCCGTGGCCAACATTCCACTCACCTTTCTCGATCCAATTTATGGAACACCCGAGTTAACCTTTACAGATTTCATGGCTCCAGGCATTATTCTGACCATTGTGCACAGTATGGCATTGGGTTACAGTGCCATGATTATCATATTAGAGCGCAATGAGGGATTGCTAGATCGAACTTGGATCGCAGGGGTCACTCCAGGCGAGTTTGCTTTGGCTCATTTCCTTGTGTCTTTGGTCGTCAACTTCCTGCAAGTGGTGGTGACCCTCACCTTCATGATCCTCGTGTTTCAGGTTCCAAATGAAGGCTCGTTGTGTTTGGTGTTCCTCCTGACCATGTTGCAAGGTGTGGCAGGAACCACCATTGGCTTGATCATATCGGCCGTGTGCAAGACTCAACAAGATGCCACTCAAATCGCATTGGGCATCTTTTATCCCAATCTGATTCTTAGCGGAATCATCTGGCCCGTTGAAAGCATGCCATTAGCTTTAAGATACTTTGCTTACACTTTGCCTCAAACGTTTGCTTGTGAAGCCATGCGTGGTATTCTGCTCAGAGGTTGGGGAATGGCCTTCATGCCCGTTTGGAGAGGATTTGTGGTCACATTGGCGTGGATCATTGGAACCTATATCATCTTTAAGCGCTTGGTGACCAAG**TGA**

>ABCH 55580

**ATG**GAGCCCAAAAACGCCATTGCAATCAAAAGTGCTTACAAACATTATGGGCGAGGTGATAAGAGGTTGGATGTCCTTCGGGATCTGAACATGACGGTGCCAAAAGGAGCCATTTATGGACTCCTCGGACCCAGTGGGTGTGGCAAGACAACACTTCTTTCAAGTGTCATTGGTCAAAGCAAATTATCTCATGGAACGGTGCAAATTTTTGATTCAAATCCGGAATATTCGATTTCCCGGATTCCAGGACCCCGAGTGGGCTACATGCCTCAAGAGACGGCCTTGTGTCAAGAACTCAGCATCATGGAAACTTTGCAGTATTTTGGCAGACTCAATCTCATGAATGAAGCCAGTATTCGCATTCGCTGTCTCTTCTTGATCAGCCTCTTGGACTTGCCGGAACCAAAAAGAGTGGTGGCCAATCTTTCCGGAGGCCAGAAGCGACGAGTGTCTTTGGCAGCTGCTCTTATCCATGAACCAGAACTGTTGATACTGGATGAACCCACTGTCGGAGTTGATCCATTGCTAAGAGAATGTATTTGGAAACATCTTTTGGAGTTGTCCAACGAATGCCAAACCACCATTGTCATCACCACACATTTTATTGAGGAAACTCGACAAGCCCACATTGTGGGTATGATGAGATTAGGCAAGATTTTGGTCGAAGGTAAACCCAGCGAGATCTTGGAACGGTATTCCAAGGCCTCCTTGGACGAAGTATTCCTTCATGTGTGCTTGGGTCAAGATGACCTTGTTTCAATTAACCGATCCAACCGCTCAAGGGCTACCATTGAAAGTGGTCTTAATCACCCAGAGCAGGTGTCTCTTAAAGAGAATGGCCAAAACCCAAAAGATGTCCCAATGACCTTTCAATCAAAACGCCAAAGAAAGACAAGATGCTGTCAATCAGGCATGAAATTCAAAAGAGTCAAGGCCCTTACGATCAAGAACTTCATCAAGATGTGGAGAAATATGGGCTCGGTCATTTTTCAGTTGCTAATTCCTGCATTCCAAGTGGCACTATTTTGTTTGGCCATCGGAAACATCCCCAGAAACCTTGATATCGCCATAATTAACCAAGAGATATCGGATTCCACGTGCGATTACTATTCGACTGCTTGTGTTTTGGGAGAACGATCCGATTTCATCGGCGGCTACGATTTTCAGGCTCAAAATCGGGCGAACCTAAGTTGCAGATTTTTGTCTCACCTTGATCAAAGTGTGATTCGCTTCAAACCATTTGACTCATTTGAATCAGGCCTGGAATCAACACGAACGGGCGATACTTGGGGCTTGATTCACTTCAAAGATAATTTCTCGGAACGGTTCTTTGATAGATTCTTGGCCTTGATCGAATCTCGTAGCTTGAATGCAAGTCAGCTCGCCGAAAGTGATATCCAAATCAAACTGGACATGACCAATCAGCAAGTTGGAATTACCCTTCAAATGGAACTTGTCGAAGCTTTTAAACGATTTTCCGAGGACGTGATCAGTTCATGCAACATCTCCACCAAATTGGCCTCTATTCCTCTACGCTTCAGCGATCCCGTATATGGAAAATACAAGCAAACTCTGACCGACTTTGCTGTCCCGGGAATCATCCTCTTGGTGACGTACTTCATGGCGTTGGGGTTAACGTCGTTGGCCTTTATAGTGGAACGAAACGAAGGGTTGCTGCATCGCAACTGGGTGGCCGGCGTCACGCCCACCGAGCTCATGCTAGCCCATGTTCTAGCTCAGATCTCCGTGATGGCCGTTCAGACAATTCTCATGTTGGGCTATGTCATTTTCATCTATCAACTGCCCACTGCAGGATCATTAGCTTGCATCTCAGGGCTCACTTTGCTCCAAGGACTTTGTGGCATGGCCAATGGTCTTTTGGTCAGCGCAATTAGCAACACTGAGGAGATGGGAATCTATCTCTGCCTAGCAACATTCTTCCCCAACATGACCTTGAGCGGGATATTTTGGCCTTTGGAAGGTATGCCCGCATGGCTTCATCAAATAGCTGTTGTTTTGCCGCAAACATACGCGTGTGAGGCACTTCGACACGTGTTCTTCAAAGGATGGGGCATGGAGTGGGTTCAGGTGTACCGTGCCTTTGCATTGTCTGTTGCTTGGACCTTGGCCTTTCTTATCTCATCAGTATTCATTTTTAAAGTACGACGT**TGA**

>ABCH 68644

**ATG**GATATTGTGTTTGATAATGTGTGTCTGTCTTATGGGCGTGGTAAGAAAAGATTCTCAGCTTTGAAAGGTGTCTCACTAAATGTTGATCAAGGGACAATCTACGGTTTGGTTGGACCTTCTGGCAGTGGAAAGACTTCTATTTTGTCATGTTGCTTGGATCTTCAGCAACCAGATTCCGGGAGCGTCCGCATTGCCGGGCAAAATCCGGGCGATAAACACCTAGGGATTCCCGGACCCACTGTTGGATACATGCCACAGGAGTTGTCAGTCCACGCTTTTTTCAATCCAAGAGAGATTCTTCATTACTATGGAAAGATCTTCAACGTTCAGGATTTGGAACAACGAGTAACAACCGTTTTGGAACTCCTCGATCTGAATCAAAATGGCTTGGATAAACGTCAGATAGGTTTTATGAGCGGAGGCCAGAAACGACGCGTGTCCTTGGCTTGTGCCATGGTTCATAATCCTCGGTTATTGATTCTCGATGAGCCAACTGTTGGTGTGGATCCACTACTAAGACGAGCGATCTGGACCGAGTTGAATGTTTTGGCATCAAGTGGAACCACTGTCCTCATCACCACACACTATTTGCAAGAAGTCGCATCCGCTTCAAAGGTGGGATTTTTGAGGGATGGGCGAATCCTACTGGAAGACGATCCCACGCAATTAATGGCCCAACGTCATGAAACATCTCTAGAAAAGATATTTCTGGACCTTTGCTTGGATGAGATTCCGCATTCATCGTCAGAAAGACTTTCCATTGAGAGTGGCAGTGCTTTTGAACTCGAGCCCTTGTCGCATGGTTCAACCTTAGCTTCATCCAAAGAAATTGAACCAAGTGAAGAACGGAATGAGCCCGTTGCTAATCCCTCATTCAGAAGTGTATTGAATCCTCTCATTCTCAAGAATATGAAGATTCTCCAAAGGCACACGGTTCTGTTGTCATTTCAAGCGATTTTACCACTTCTGACTTATCTCATGTTTCTTGTTTGCATCGGAAGACCTTTGCATGGTCTAGACTTAGGAATCGTGAACGAAGAAAGTGCATGTTGCCCGCAACAAAGAACTAATCTGACGTGTCTCGCCATGAGCCTTCAAATGGTCTTGCGAAGAGAGTTCACCTTGGGTGAAGATGTTTCGTGCCATGTTATAGATAATCTGGATCCTCATATCTTCAGTTTAAAGTATTTCAAATCCTTTGAAGAGGCCAAGGAATCGGTCGTTCTTGGCCAAACCCATGGCTTTGTCAGATTTCCATCCGACTTTTCTGTAGGACTTGAACAGAGAATTGCCACTCTTGTCGACACGGACAACACCCTTGAAGATGATATCTTAAGTCATACCAGGATGTTTGTTCACACGGACGCTTCAAATGTCAAAGTATTTGCCTCATTACGTGAGAACCTTCTATTTGGTCTTCAAAAGTTTTTCATCCAATACGGCGAATCGTGTCACTTGGAGCAACTTGTAAAGACCATCACCTCGCCCGGAATTGCGATTCATTCCCTCAATCCTGAGTTTAGACACCAAGACTATGACCTTTCAGAGCTAATGTTTCCCGGTATGATGACCTTATTGCTGAATCTTCTTCCTATGGCTTTAACAGCTGATCAGCTGATGACGGATAAGGAAGCAGGGCTGCTGCTCCGAGATTTTGTGTGCAGGGTGCCCTTCTCGGCTGTCATTTTCACTCAATTGTTTTGCCAACTCGTTGTGGTCATGGTTCAATTGGTCTCATCGTTGATATTGGTTCTCGTGTTGTTTCCAACGACTTCAATAGGAGTGCTAGTCTTCGCGGGTTTTCTGCTCTTCCTTCAAGCTTGTTGCGGAATGGCTATGGGCGTGTTTATTTGCTCGTTATGTAGTTCCAGGGACCAAGTGATCCAGGTGGGAATGGCGGTGGTGATGCCCGCATTTATAGTCTCCGGGATTCTATGGCCAAGATCAACAATGCCTGAAGTGCTTCAAGTGATATCAGCCATGATCCCCACCACATTTGCTTGTGACATCATGAAGAACATCATTCTTAACAACACAATATTTGTCAATGGCCTGTGGAAAGCCATACTTTTGCCAACGGCTGGGAGTGCCATCTTTTTCTTTGGATGCCTTTTTATGGTTTCTAGAGTGGGCCTAAAA**TGA**
