# Supplementary material for: Genome-wide identification of whole ATP-binding cassette (ABC) transporters in the intertidal copepod Tigriopus japonicus
Source: BMC Genomics. 2014 Aug 5;15(1):651. doi: 10.1186/1471-2164-15-651 (PMC4247197; doi:10.1186/1471-2164-15-651)
Supplement: Supplementary file 2 — Additional file 2: Phylogenetic analysis of T. japonicus ABCA subfamily with those of other species using Bayesian method. Numbers at branch nodes represent the confidence level of posterior probability. (PPTX 85 KB) [file 12864_2014_6676_MOESM2_ESM.pptx]

## Slide 1
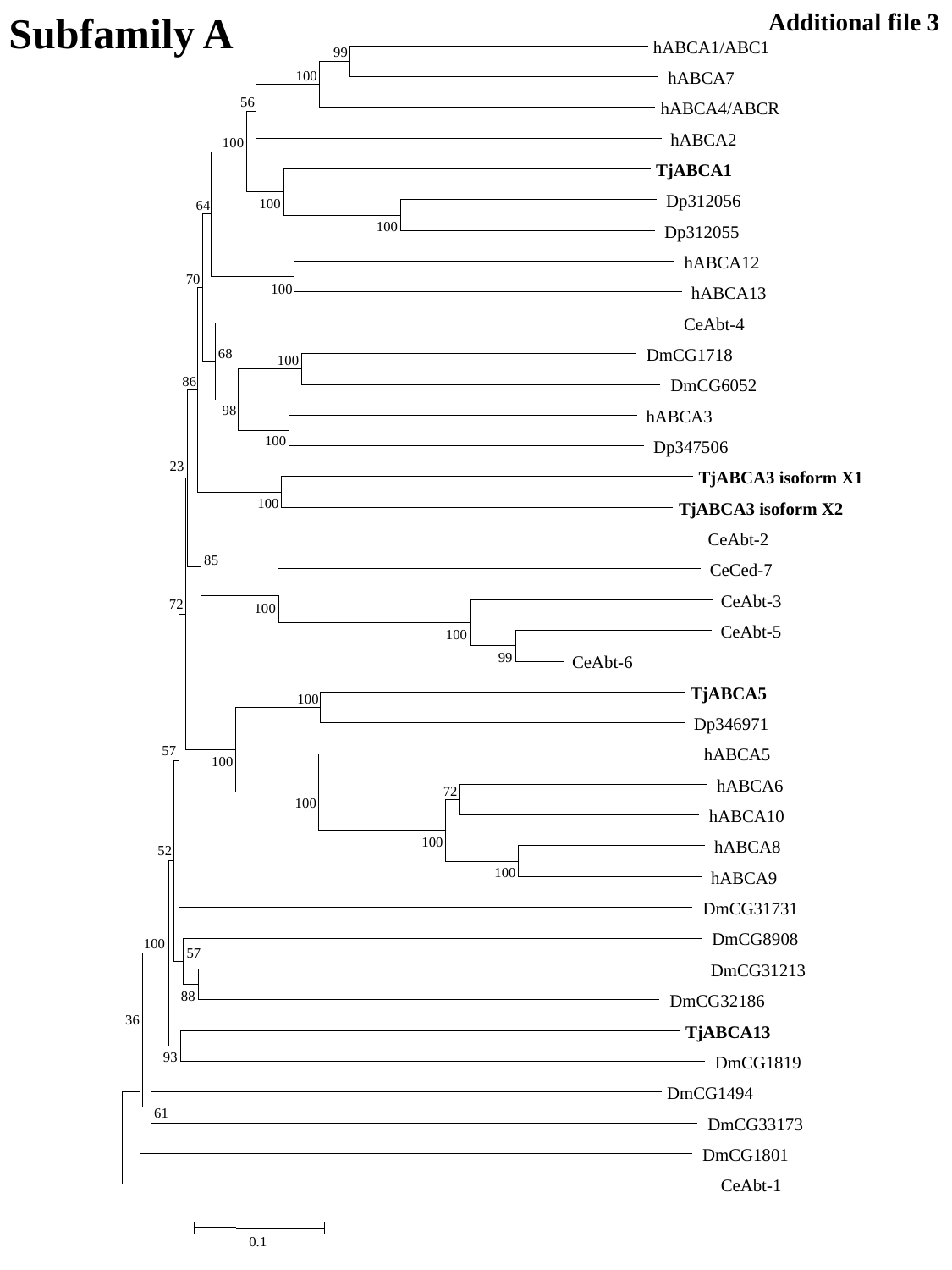

Subfamily A
Additional file 3
 hABCA1/ABC1
99
100
 hABCA7
56
 hABCA4/ABCR
 hABCA2
100
 TjABCA1
 Dp312056
100
64
100
 Dp312055
 hABCA12
70
100
 hABCA13
 CeAbt-4
 DmCG1718
68
100
86
 DmCG6052
98
 hABCA3
100
 Dp347506
23
 TjABCA3 isoform X1
100
 TjABCA3 isoform X2
 CeAbt-2
85
 CeCed-7
 CeAbt-3
72
100
 CeAbt-5
100
99
 CeAbt-6
 TjABCA5
100
 Dp346971
57
 hABCA5
100
 hABCA6
72
100
 hABCA10
100
 hABCA8
52
100
 hABCA9
 DmCG31731
 DmCG8908
100
57
 DmCG31213
88
 DmCG32186
36
 TjABCA13
93
 DmCG1819
 DmCG1494
61
 DmCG33173
 DmCG1801
 CeAbt-1
0.1
