# Supplementary material for: Genome-wide identification of whole ATP-binding cassette (ABC) transporters in the intertidal copepod Tigriopus japonicus
Source: BMC Genomics. 2014 Aug 5;15(1):651. doi: 10.1186/1471-2164-15-651 (PMC4247197; doi:10.1186/1471-2164-15-651)
Supplement: Supplementary file 3 — Additional file 3: Phylogenetic analysis of T. japonicus ABCB subfamily with those of other species using Bayesian method. Numbers at branch nodes represent the confidence level of posterior probability. (PPTX 95 KB) [file 12864_2014_6676_MOESM3_ESM.pptx]

## Slide 1
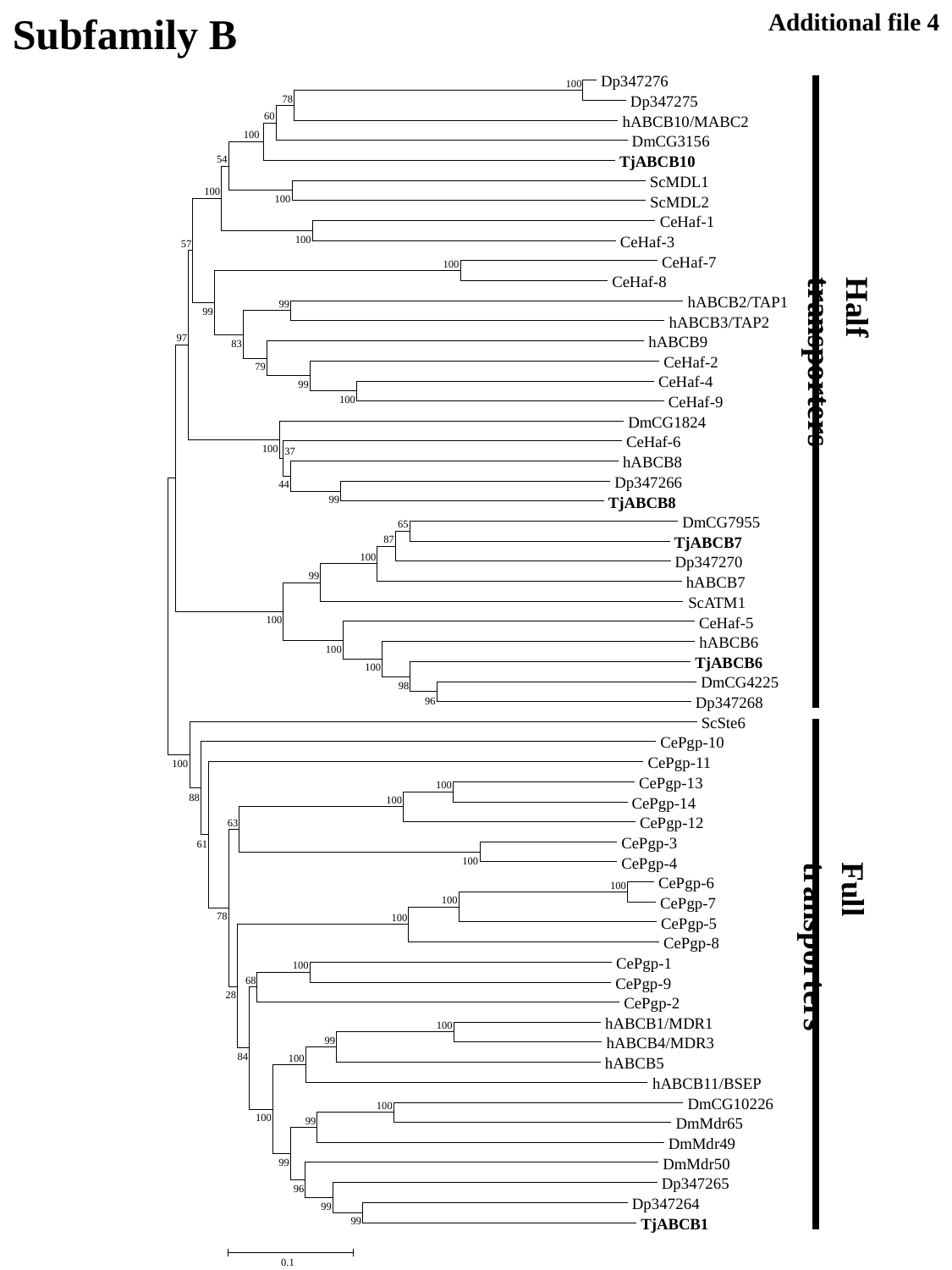

Additional file 4
Subfamily B
 Dp347276
100
 Dp347275
 hABCB10/MABC2
 DmCG3156
 TjABCB10
 ScMDL1
 ScMDL2
100
 CeHaf-1
 CeHaf-3
100
 CeHaf-7
100
 CeHaf-8
 hABCB2/TAP1
99
 hABCB3/TAP2
 hABCB9
 CeHaf-2
 CeHaf-4
99
 CeHaf-9
100
 DmCG1824
 CeHaf-6
 hABCB8
 Dp347266
44
 TjABCB8
99
 DmCG7955
65
 TjABCB7
87
100
 Dp347270
99
 hABCB7
 ScATM1
 CeHaf-5
100
 hABCB6
100
 TjABCB6
100
 DmCG4225
98
 Dp347268
96
 ScSte6
 CePgp-10
 CePgp-11
 CePgp-13
100
 CePgp-14
100
 CePgp-12
 CePgp-3
 CePgp-4
100
 CePgp-6
100
 CePgp-7
100
100
 CePgp-5
 CePgp-8
 CePgp-1
 CePgp-9
 CePgp-2
 hABCB1/MDR1
100
 hABCB4/MDR3
99
 hABCB5
 hABCB11/BSEP
 DmCG10226
100
 DmMdr65
 DmMdr49
 DmMdr50
 Dp347265
 Dp347264
 TjABCB1
99
78
60
100
54
100
57
99
97
83
79
100
37
100
88
63
61
78
100
68
28
84
100
100
99
99
96
99
0.1
Half transporters
Full transporters
