# Supplementary material for: Genome-wide identification of whole ATP-binding cassette (ABC) transporters in the intertidal copepod Tigriopus japonicus
Source: BMC Genomics. 2014 Aug 5;15(1):651. doi: 10.1186/1471-2164-15-651 (PMC4247197; doi:10.1186/1471-2164-15-651)
Supplement: Supplementary file 4 — Additional file 4: Phylogenetic analysis of T. japonicus ABCC subfamily with those of other species using Bayesian method. Numbers at branch nodes represent the confidence level of posterior probability. (PPTX 93 KB) [file 12864_2014_6676_MOESM4_ESM.pptx]

## Slide 1
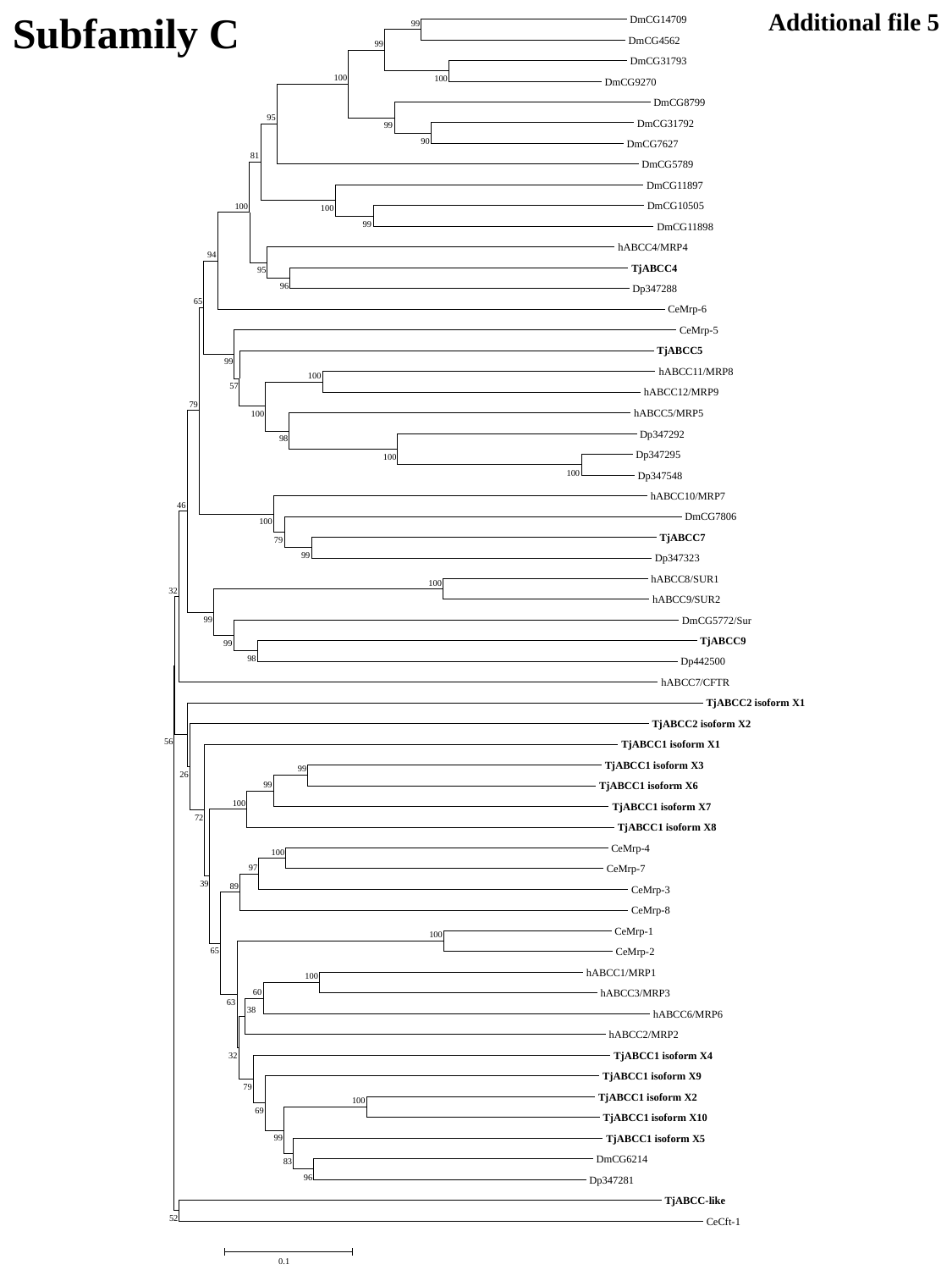

Additional file 5
Subfamily C
 DmCG14709
99
 DmCG4562
99
 DmCG31793
100
100
 DmCG9270
 DmCG8799
95
 DmCG31792
99
90
 DmCG7627
 DmCG5789
 DmCG11897
 DmCG10505
100
99
 DmCG11898
 hABCC4/MRP4
 TjABCC4
96
 Dp347288
 CeMrp-6
 CeMrp-5
 TjABCC5
 hABCC11/MRP8
100
 hABCC12/MRP9
 hABCC5/MRP5
 Dp347292
98
 Dp347295
100
100
 Dp347548
 hABCC10/MRP7
 DmCG7806
100
 TjABCC7
79
99
 Dp347323
 hABCC8/SUR1
100
 hABCC9/SUR2
 DmCG5772/Sur
 TjABCC9
98
 Dp442500
 hABCC7/CFTR
 TjABCC2 isoform X1
 TjABCC2 isoform X2
 TjABCC1 isoform X1
 TjABCC1 isoform X3
99
 TjABCC1 isoform X6
 TjABCC1 isoform X7
 TjABCC1 isoform X8
 CeMrp-4
100
 CeMrp-7
 CeMrp-3
 CeMrp-8
 CeMrp-1
100
 CeMrp-2
 hABCC1/MRP1
100
 hABCC3/MRP3
 hABCC6/MRP6
 hABCC2/MRP2
 TjABCC1 isoform X4
 TjABCC1 isoform X9
 TjABCC1 isoform X2
100
 TjABCC1 isoform X10
 TjABCC1 isoform X5
 DmCG6214
96
 Dp347281
 TjABCC-like
 CeCft-1
81
100
94
95
65
99
57
79
100
46
32
99
99
56
26
99
100
72
97
39
89
65
60
63
38
32
79
69
99
83
52
0.1
