# Supplementary material for: Genome-wide identification of whole ATP-binding cassette (ABC) transporters in the intertidal copepod Tigriopus japonicus
Source: BMC Genomics. 2014 Aug 5;15(1):651. doi: 10.1186/1471-2164-15-651 (PMC4247197; doi:10.1186/1471-2164-15-651)
Supplement: Supplementary file 5 — Additional file 5: Phylogenetic analysis of T. japonicus ABCD and ABCE subfamilies with those of other species using Bayesian method. Numbers at branch nodes represent the confidence level of posterior probability. (PPTX 66 KB) [file 12864_2014_6676_MOESM5_ESM.pptx]

## Slide 1
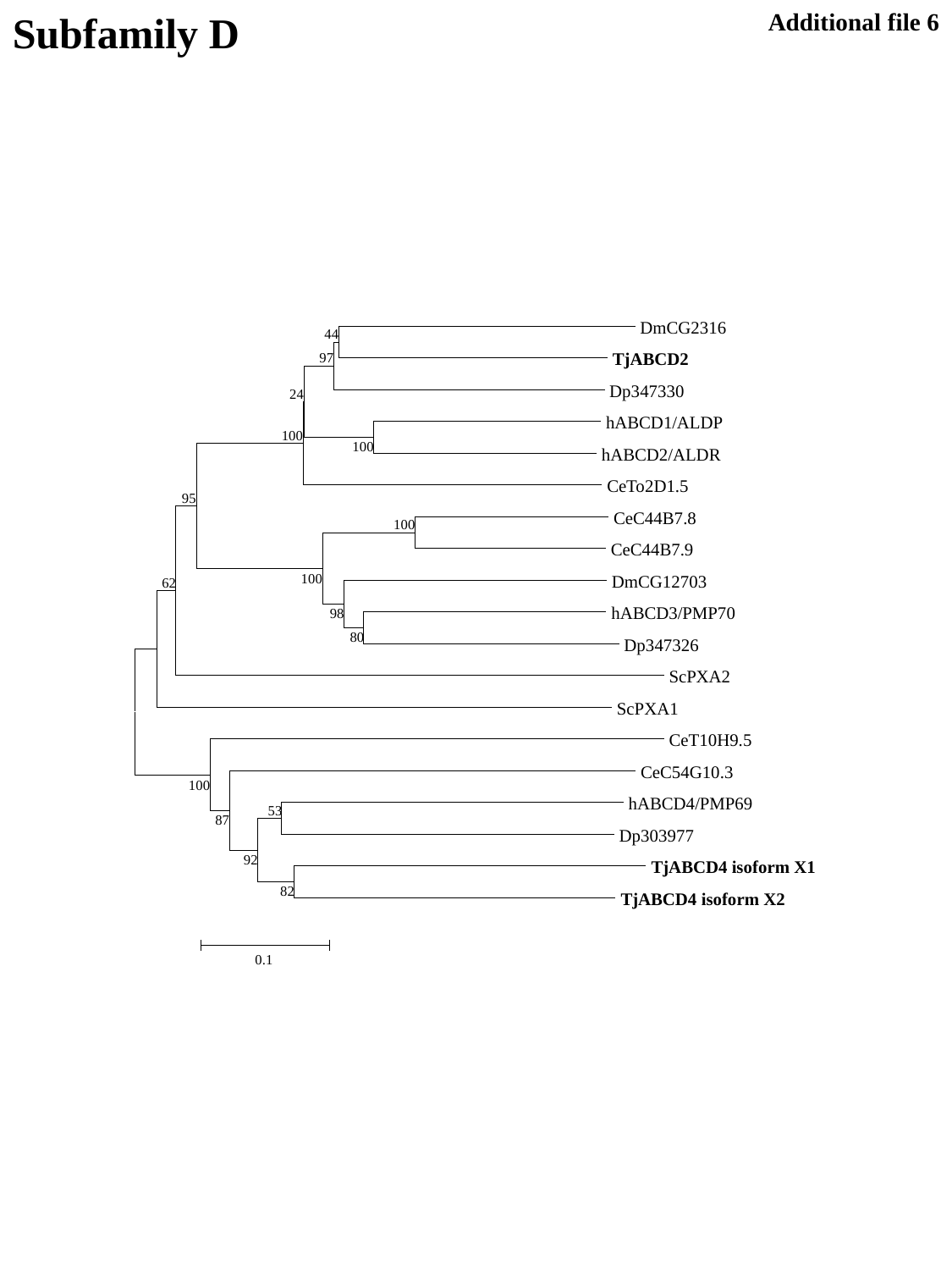

Additional file 6
Subfamily D
 DmCG2316
44
 TjABCD2
97
 Dp347330
24
 hABCD1/ALDP
100
100
 hABCD2/ALDR
 CeTo2D1.5
95
 CeC44B7.8
100
 CeC44B7.9
100
 DmCG12703
62
 hABCD3/PMP70
98
80
 Dp347326
 ScPXA2
 ScPXA1
 CeT10H9.5
 CeC54G10.3
100
 hABCD4/PMP69
53
87
 Dp303977
92
 TjABCD4 isoform X1
82
 TjABCD4 isoform X2
0.1
