# Supplementary material for: Genome-wide identification of whole ATP-binding cassette (ABC) transporters in the intertidal copepod Tigriopus japonicus
Source: BMC Genomics. 2014 Aug 5;15(1):651. doi: 10.1186/1471-2164-15-651 (PMC4247197; doi:10.1186/1471-2164-15-651)
Supplement: Supplementary file 6 — Additional file 6: Phylogenetic analysis of T. japonicus ABCF subfamilies with those of other species using Bayesian method. Numbers at branch nodes represent the confidence level of posterior probability. (PPTX 82 KB) [file 12864_2014_6676_MOESM6_ESM.pptx]

## Slide 1
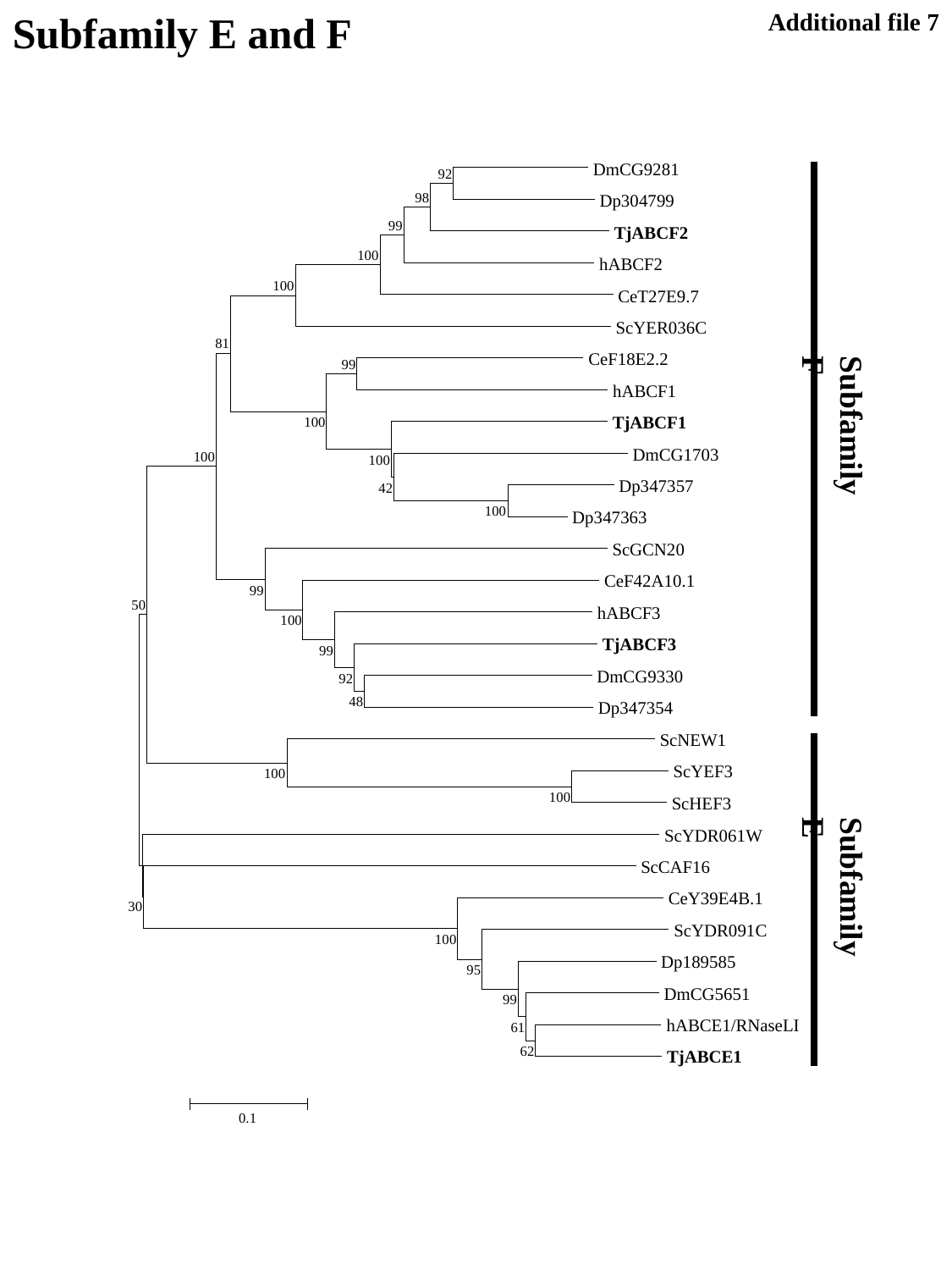

Additional file 7
Subfamily E and F
 DmCG9281
92
98
 Dp304799
99
 TjABCF2
100
 hABCF2
100
 CeT27E9.7
 ScYER036C
81
 CeF18E2.2
99
 hABCF1
 TjABCF1
100
 DmCG1703
100
100
 Dp347357
42
100
 Dp347363
 ScGCN20
 CeF42A10.1
99
50
 hABCF3
100
 TjABCF3
99
 DmCG9330
92
48
 Dp347354
 ScNEW1
 ScYEF3
100
100
 ScHEF3
 ScYDR061W
 ScCAF16
 CeY39E4B.1
30
 ScYDR091C
100
 Dp189585
95
 DmCG5651
99
 hABCE1/RNaseLI
61
62
 TjABCE1
0.1
Subfamily F
Subfamily E
