# Supplementary material for: Genome-wide identification of whole ATP-binding cassette (ABC) transporters in the intertidal copepod Tigriopus japonicus
Source: BMC Genomics. 2014 Aug 5;15(1):651. doi: 10.1186/1471-2164-15-651 (PMC4247197; doi:10.1186/1471-2164-15-651)
Supplement: Supplementary file 7 — Additional file 7: Phylogenetic analysis of T. japonicus ABCG subfamilies with those of other species using Bayesian method. Numbers at branch nodes represent the confidence level of posterior probability. (PPTX 101 KB) [file 12864_2014_6676_MOESM7_ESM.pptx]

## Slide 1
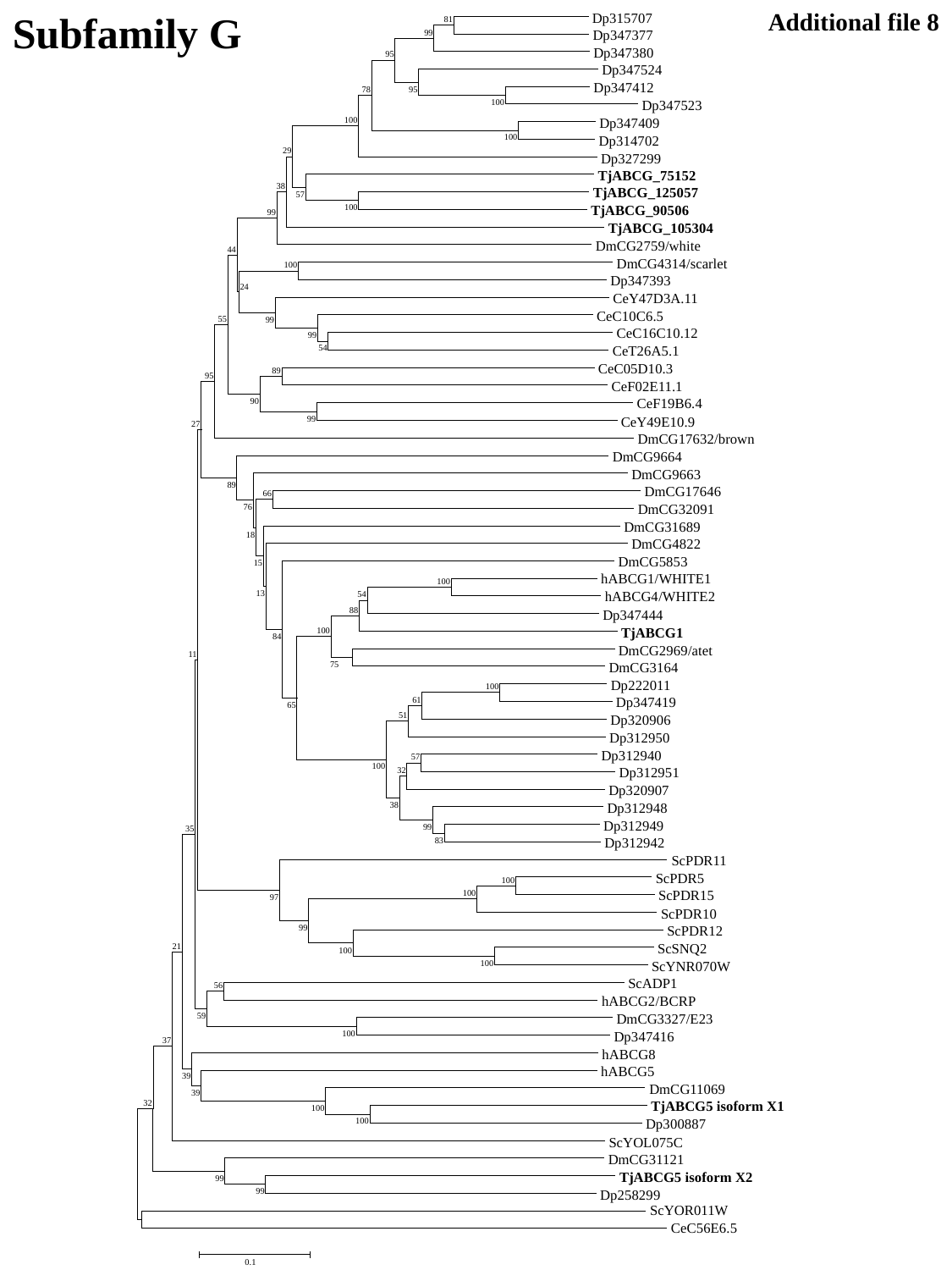

Additional file 8
Subfamily G
 Dp315707
 Dp347377
 Dp347380
 Dp347524
 Dp347412
 Dp347523
 Dp347409
 Dp314702
 Dp327299
 TjABCG_75152
 TjABCG_125057
 TjABCG_90506
 TjABCG_105304
 DmCG2759/white
 DmCG4314/scarlet
 Dp347393
 CeY47D3A.11
 CeC10C6.5
 CeC16C10.12
 CeT26A5.1
 CeC05D10.3
 CeF02E11.1
 CeF19B6.4
 CeY49E10.9
 DmCG17632/brown
 DmCG9664
 DmCG9663
 DmCG17646
 DmCG32091
 DmCG31689
 DmCG4822
 DmCG5853
 hABCG1/WHITE1
 hABCG4/WHITE2
 Dp347444
 TjABCG1
 DmCG2969/atet
 DmCG3164
 Dp222011
 Dp347419
 Dp320906
 Dp312950
 Dp312940
 Dp312951
 Dp320907
 Dp312948
 Dp312949
 Dp312942
 ScPDR11
 ScPDR5
 ScPDR15
 ScPDR10
 ScPDR12
 ScSNQ2
 ScYNR070W
 ScADP1
 hABCG2/BCRP
 DmCG3327/E23
 Dp347416
 hABCG8
 hABCG5
 DmCG11069
 TjABCG5 isoform X1
 Dp300887
 ScYOL075C
 DmCG31121
 TjABCG5 isoform X2
 Dp258299
81
99
95
78
95
100
100
100
29
38
57
100
99
44
100
24
55
99
99
54
89
95
90
99
27
89
66
76
18
15
100
13
54
88
100
84
11
75
100
61
65
51
57
100
32
38
99
35
83
100
100
97
99
21
100
100
56
59
100
37
39
39
32
100
100
99
99
 ScYOR011W
 CeC56E6.5
0.1
