# Supplementary material for: Genome-wide identification of whole ATP-binding cassette (ABC) transporters in the intertidal copepod Tigriopus japonicus
Source: BMC Genomics. 2014 Aug 5;15(1):651. doi: 10.1186/1471-2164-15-651 (PMC4247197; doi:10.1186/1471-2164-15-651)
Supplement: Supplementary file 8 — Additional file 8: Phylogenetic analysis of T. japonicus ABCH subfamilies with those of other species using Bayesian method. Numbers at branch nodes represent the confidence level of posterior probability. (PPTX 67 KB) [file 12864_2014_6676_MOESM8_ESM.pptx]

## Slide 1
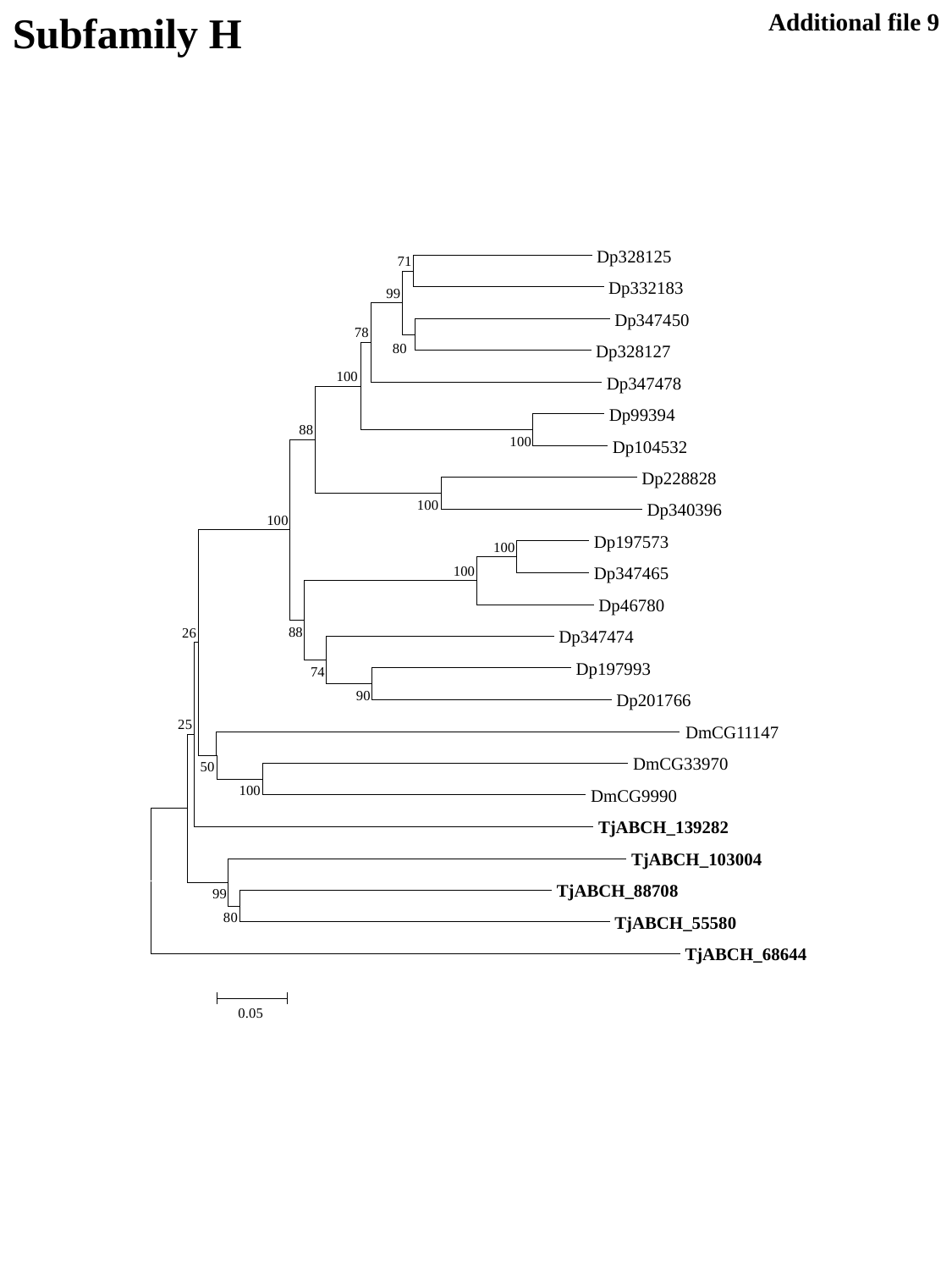

Additional file 9
Subfamily H
 Dp328125
71
 Dp332183
99
 Dp347450
78
80
 Dp328127
100
 Dp347478
 Dp99394
88
100
 Dp104532
 Dp228828
100
 Dp340396
100
 Dp197573
100
100
 Dp347465
 Dp46780
88
26
 Dp347474
 Dp197993
74
90
 Dp201766
25
 DmCG11147
 DmCG33970
50
100
 DmCG9990
 TjABCH_139282
 TjABCH_103004
 TjABCH_88708
99
80
 TjABCH_55580
 TjABCH_68644
0.05
