# Supplementary figures and images for: Genome-wide identification of whole ATP-binding cassette (ABC) transporters in the intertidal copepod Tigriopus japonicus
Source: BMC Genomics. 2014 Aug 5;15(1):651. doi: 10.1186/1471-2164-15-651 (PMC4247197; doi:10.1186/1471-2164-15-651)

## Slide 1
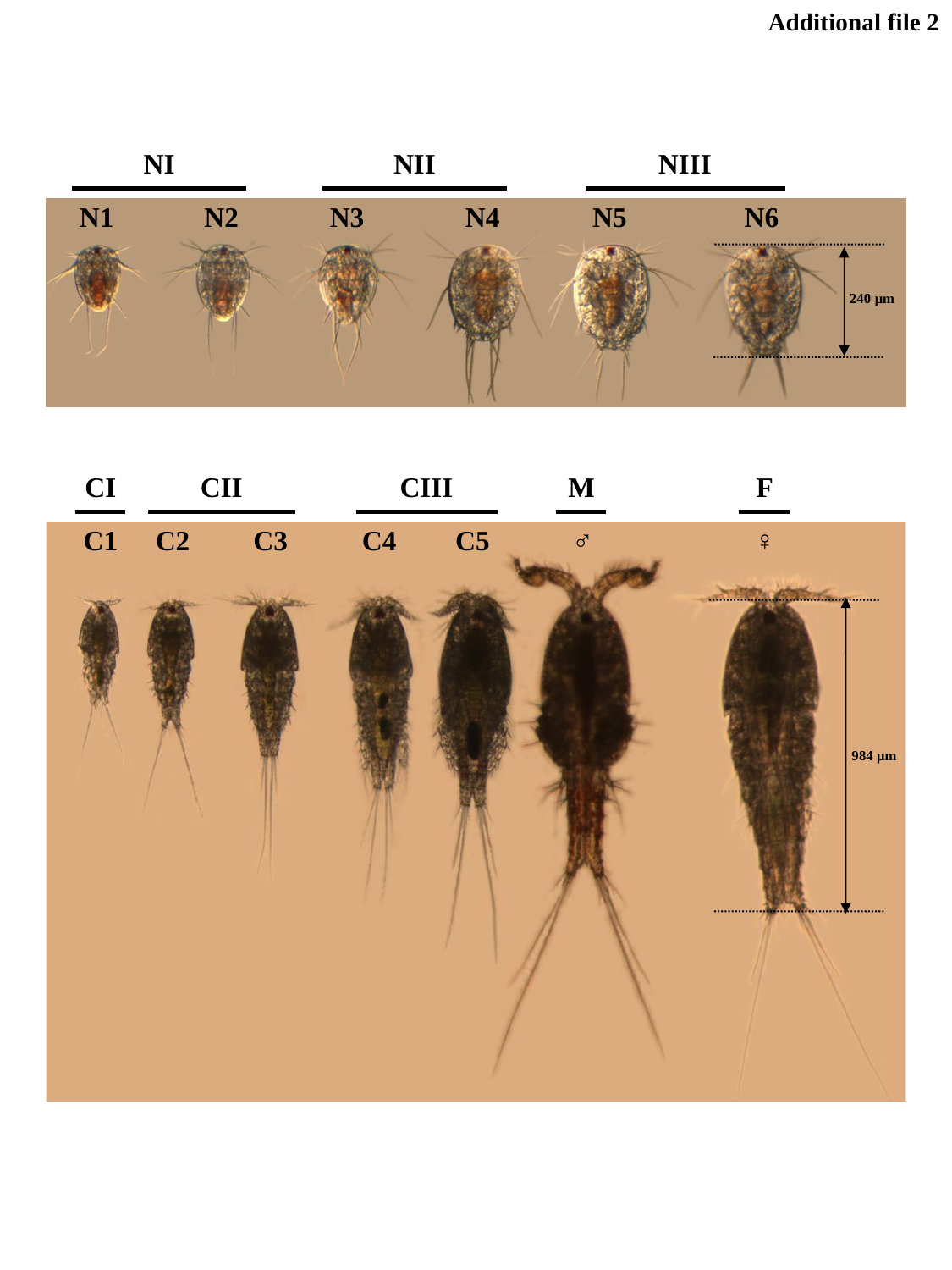

Additional file 2
NI
NII
NIII
N1
N2
N3
N4
N5
N6
240 μm
CI
CII
CIII
M
F
C1
C2
C3
C4
C5
♂
♀
984 μm

Supplement: Supplementary file 9 — Additional file 9: Developmental stages of the intertidal hapacticoid copepod, T. japonicus . Stages 1–6 are nauplius (N) stages and five stages in the second row represent copepodite (C) stages. Figure was modified from our previous publication (Seo et al., 2006). (PPTX 997 KB) [file 12864_2014_6676_MOESM9_ESM.pptx]
