# Supplementary material for: Genome-wide identification of whole ATP-binding cassette (ABC) transporters in the intertidal copepod Tigriopus japonicus
Source: BMC Genomics. 2014 Aug 5;15(1):651. doi: 10.1186/1471-2164-15-651 (PMC4247197; doi:10.1186/1471-2164-15-651)
Supplement: Supplementary file 10 — Additional file 10: Result of gene-specific hierarchical clustering analysis with temporal transcriptional expressions of T. japonicus 46 ABC transporters in different developmental stages (N, nauplius; C, copepodid; M, male; F, female). (PPTX 193 KB) [file 12864_2014_6676_MOESM10_ESM.pptx]

## Slide 1
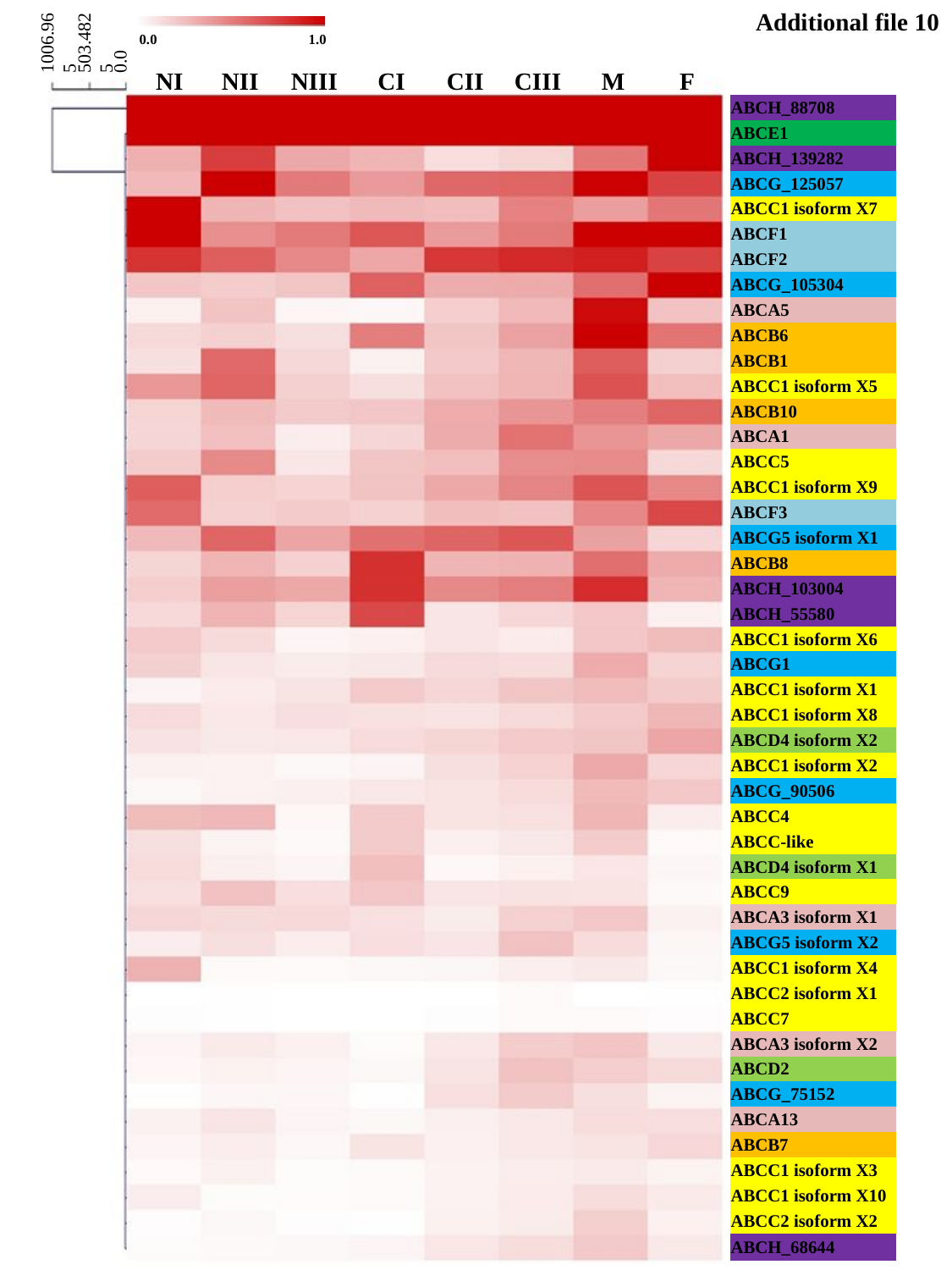

Additional file 10
1006.965
503.4825
0.0
0.0
1.0
NI
NII
NIII
CI
CII
CIII
M
F
| ABCH\_88708 |
| --- |
| ABCE1 |
| ABCH\_139282 |
| ABCG\_125057 |
| ABCC1 isoform X7 |
| ABCF1 |
| ABCF2 |
| ABCG\_105304 |
| ABCA5 |
| ABCB6 |
| ABCB1 |
| ABCC1 isoform X5 |
| ABCB10 |
| ABCA1 |
| ABCC5 |
| ABCC1 isoform X9 |
| ABCF3 |
| ABCG5 isoform X1 |
| ABCB8 |
| ABCH\_103004 |
| ABCH\_55580 |
| ABCC1 isoform X6 |
| ABCG1 |
| ABCC1 isoform X1 |
| ABCC1 isoform X8 |
| ABCD4 isoform X2 |
| ABCC1 isoform X2 |
| ABCG\_90506 |
| ABCC4 |
| ABCC-like |
| ABCD4 isoform X1 |
| ABCC9 |
| ABCA3 isoform X1 |
| ABCG5 isoform X2 |
| ABCC1 isoform X4 |
| ABCC2 isoform X1 |
| ABCC7 |
| ABCA3 isoform X2 |
| ABCD2 |
| ABCG\_75152 |
| ABCA13 |
| ABCB7 |
| ABCC1 isoform X3 |
| ABCC1 isoform X10 |
| ABCC2 isoform X2 |
| ABCH\_68644 |
